# Comparative Analysis of the Complete Chloroplast Genomes of Nine *Paphiopedilum* Species

**DOI:** 10.3389/fgene.2021.772415

**Published:** 2022-02-04

**Authors:** Yin Sun, Peishan Zou, Nannan Jiang, Yifu Fang, Guofeng Liu

**Affiliations:** ^1^ Shandong Provincial Academy of Forestry, Jinan, China; ^2^ Department of Botany, Guangzhou Institute of Forestry and Landscape Architecture, Guangzhou, China

**Keywords:** paphiopedilum, plastid genome, comparative genomics, divergence, simple sequence repeats, phylogeny

## Abstract

*Paphiopedilum* is known as “lady’s or Venus” slipper orchids due to its prominent shoe-shaped labellum, with high ornamental value. Phylogenetic relationships among some species in *Paphiopedilum* genus cannot be effectively determined by morphological features alone or through the analysis of nuclear or chloroplast DNA fragments. In order to provide aid in understanding the evolutionary and phylogenetic relationship in *Paphiopedilum* at chloroplast (cp) genome-scale level, the complete cp genomes of six *Paphiopedilum* species were newly sequenced in this study, and three other published cp genome sequences of *Paphiopedilum* were included in the comparative analyses. The cp genomes of the six *Paphiopedilum* species ranged from 154,908 bp (*P. hirsutissimum*) to 161,300 bp (*P. victoria-mariae*) in size, all constituting four-part annular structures. Analyses of the nucleotide substitutions, insertions/deletions, and simple sequence repeats in the cp genomes were conducted. Ten highly variable regions that could serve as potential DNA barcodes or phylogenetic markers for this diverse genus were identified. Sequence variations in the non-coding regions were greater than that in the conserved protein-coding regions, as well as in the large single copy (LSC) and small single copy (SSC) regions than in the inverted repeat (IR) regions. Phylogenetic analysis revealed that all *Paphiopedilum* species clustered in one monophyletic clade in the Cypripedioideae subfamily and then subdivided into seven smaller branches corresponding to different subgenus or sections of the genus, with high bootstrap supports, indicate that cp genome sequencing can be an effective means in resolving the complex relationship in *Paphiopedilum*.

## Introduction

The genus *Paphiopedilum*, first described by Ernst Hugo Heinrich Pfitzer in 1886 and commonly referred to as “lady’s or Venus” slipper orchids, belongs to the subfamily Cypripedioideae of the flowering plant family Orchidaceae, along with *Cypripedium*, *Mexipedium*, *Phragmipedium*, and *Selenipedium* genera (Koopowitz, 2008). *Paphiopedilum* is one of the most widely cultivated and horticulturally important orchid genera due to its beautiful and long-lasting flowers, which is characterized by their shoe-shaped labellum and synsepalum, a structure that is formed by the fusion of two lateral sepals. The genus is native to tropical and subtropical regions, mainly distributed in Southeast Asia, southern China, northern India, and New Guinea, with over 80 original species and several thousand hybrids/cultivars distributed or cultivated worldwide (Chung et al., 2006).

**FIGURE 1:**
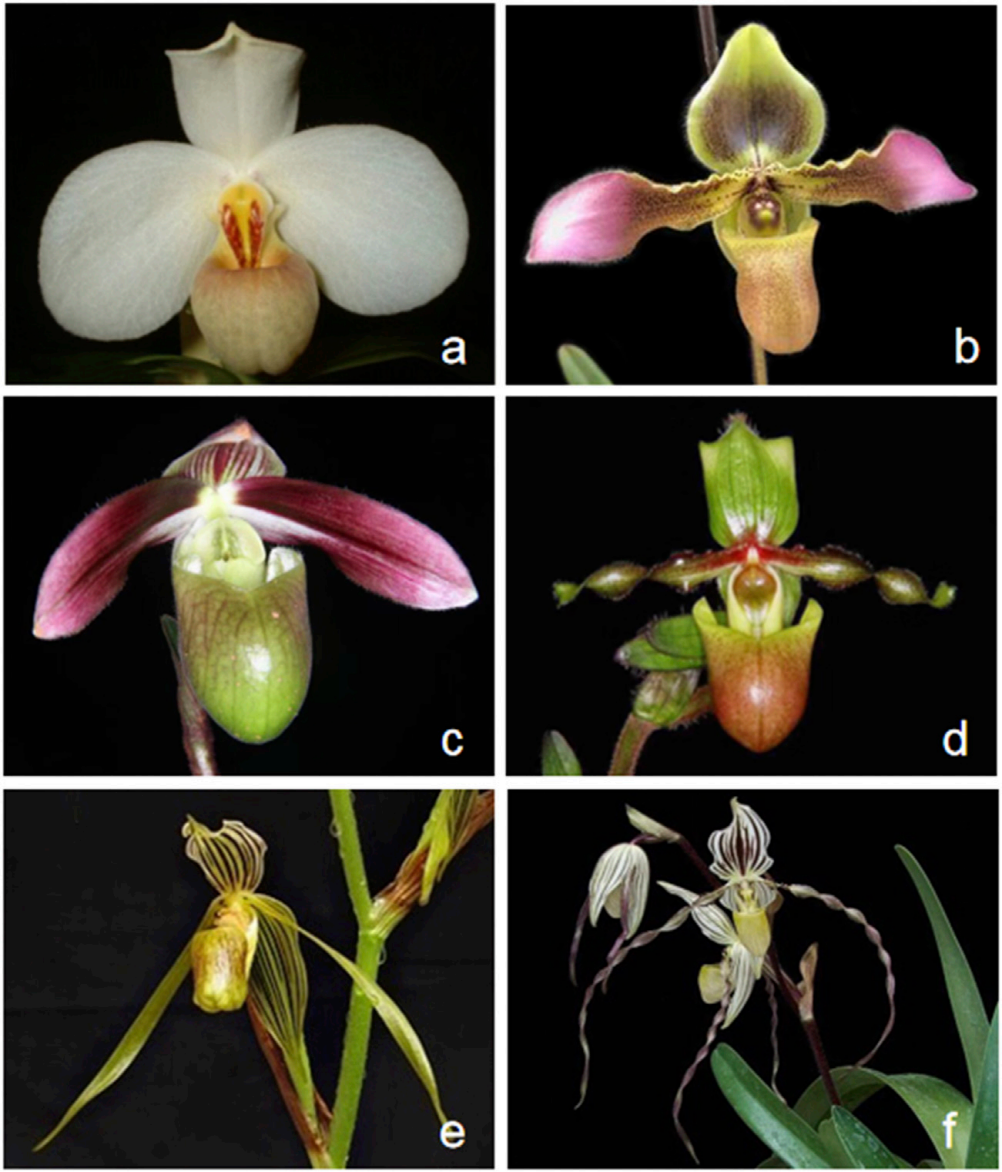
Flower morphological characteristics of six *Paphiopedilum* species. **(A)** *Paphiopedilum tranlienianum*; **(B)** *Paphiopedilum hirsutissimum*; **(C)** *Paphiopedilum violascens*; **(D)** *Paphiopedilum victoria-mariae*; **(E)** *Paphiopedilum kolopakingii*; **(F)** *Paphiopedilum philippinense*.

According to the classification of (Cribb, 1997), the genus *Paphiopedilum* was divided into three subgenera: *Parvisepalum*, *Brachypetalum*, and *Paphiopedilum*, based on the morphological, cytological, and molecular data (Cox et al., 1997; Cribb, 1997). Furthermore, the subgenus *Paphiopedilum* can be morphologically and phylogenetically subdivided into five sections: *Paphiopedilum*, *Barbata*, *Cochlopetalum*, *Coryopedilum*, and *Pardalopetalum* (Cribb, 1997). Species identification of *Paphiopedilum* during flowering is relatively easy on account of a diverse labellum pattern (Figure 1); nevertheless, it would be problematic while not blooming, simply by other phenotypic traits like leaves, stems, and roots, especially for young seedlings (Guo et al., 2016). In addition, the genetic diversity and relationship among species within *Paphiopedilum* subgenera and sections are not so clarified. Over the past decades, various molecular markers including randomly amplified polymorphic DNA (RAPD); simple sequence repeats (SSRs) ; sequence-related amplified polymorphism (SRAP); sequence-characterized amplified regions (SCARs); and sequence-based markers such as sequences of nuclear ribosomal internal transcribed spacer (ITS), low-copy nuclear genes, or chloroplast DNA fragments had been used for species identification and genetic diversity analysis of *Paphiopedilum* (Cox et al., 1997; Chung et al., 2006; Sun et al., 2011; Chochai et al., 2012; Gorniak et al., 2014; Guo et al., 2015; Xu et al., 2018; Vu et al., 2019; Tsai et al., 2020), which also provides important clues and evidences for taxonomic placement of some problematic novel species (Gorniak et al., 2014; Lee et al., 2017), while there are still some unresolved phylogenetic questions in *Paphiopedilum.* For instance, recent phylogenetic studies indicated that widespread reticulate evolution existed in the genus, and previous markers cannot solve the deep phylogenetic relationship (Chochai et al., 2012; Guo et al., 2015; Tsai et al., 2020). Currently, limited molecular and phylogenetic studies of *Paphiopedilum* have greatly hampered more profound understanding and exploitation of this valuable genus.

Chloroplast (cp) is a crucial multifunctional organelle in plants, which is involved in photosynthesis, synthesis of starch, fatty acids, amino acids and pigments, carbon cycle, and other life links. Chloroplast is a kind of semi-autonomous genetic organelle, corresponding to matrilineal inheritance with an independent transcription and transport system. The cp genomes of most angiosperms are topologically circular, ranging from 120 to 160 kb in length, with highly conserved structure, gene order, and content (Wicke et al., 2011). In general, a large single-copy (LSC) region, a small single-copy (SSC) region, and two copies of inverted repeats (IRa and IRb) constitute a typical four-part annular chloroplast genome (Curci et al., 2015), whereas gene expansion, contraction, or loss in the IR regions makes up the most structural divergence (Ma et al., 2013; Zhang et al., 2014).

Due to the low mutation rate, lack of recombination, and uniparental inheritance, cp DNA sequences are a versatile tool for plant identification or barcoding and untangling genetic relationships among plant species. However, no single locus in chloroplast genome that can distinguish all plant species from each other has been sought out. To evaluate the species identification power of frequently used plant DNA barcodes within *Paphiopedilum*, markers like *accD*, *matK*, *rbcL*, *rpoC2*, *ycf1*, *atpF-atpH*, *atpI-atpH*, and ITS were investigated, which indicated that the core plant barcodes *rbcL* and *matK* (Group, 2009) showed low resolution (18.86%), while the efficacy of a multi-locus combination of chloroplast fragment *matK* + *atpF-atpH* can reach 28.97%, but still not so ideal (Guo et al., 2016).

The whole cp genome sequencing is an informative approach for studying plant speciation and classification, which has been widely used in current comparative genomics, population genetics, and phylogenetic studies (Kress et al., 2005; Celiński et al., 2017; Park et al., 2017). In this study, the complete cp genome sequences of six *Paphiopedilum* species were reported, and their comparative analyses with another three cp genomes of *Paphiopedilum* derived from the National Center for Biotechnology Information (NCBI) database (https://www.ncbi.nlm.nih.gov) were performed. According to the recent classification, these nine taxa stand for species of all the three subgenera (*Parvisepalum*, *Brachypetalum*, and *Paphiopedilum*) and the five sections of *Paphiopedilum* subgenus (*Paphiopedilum*, *Barbata*, *Cochlopetalum*, *Coryopedilum*, and *Pardalopetalum*)*.* Through this study, we are dedicated to: 1) characterize the cp genome of representatives of different subgenera and sections in *Paphiopedilum*; 2) profile the chloroplast genetic diversity and identify the hotspots with high-divergence across the cp genome of the genus; 3) systematically reidentify the infrageneric relationship of *Paphiopedilum* and the backbone phylogeny of Orchidaceae on cp genomic level; 4) provide molecular markers for identifying and/or distinguishing the genetic germplasms, which would be beneficial to novel cultivar breeding with important economic values.

## Materials and methods

### Plant materials and DNA extraction

Six taxa of the *Paphiopedilum* subgenus, including *P. tranlienianum* and *P. hirsutissimum* from sect. *Paphiopedilum*, *P. violascens* from sect. *Barbata*, *P. victoria-mariae* from sect. *Cochlopetalum*, and *P. kolopakingii* and *P. philippinense* from sect. *Coryopedilum* (Figure 1), were obtained from Wuhan Qilan Biotechnology Co., Ltd. (Wuhan, China) and grown in the greenhouse of Shandong Provincial Academy of Forestry (Jinan, China). Genomic DNA was extracted from fresh leaves through the modified cetyltrimethyl ammonium bromide (CTAB) method and purified (Doyle and Doyle, 1987). Voucher specimens were deposited in the herbarium at Shandong Provincial Academy of Forestry (specimen code SPAF-Bore-2020-01-10.1 to SPAF-Bore-2020-01-10.6, under the charge of Yin Sun).

### Chloroplast genome sequencing, assembling, and annotation

Purified DNA samples were randomly fragmented into ∼400 bp using an ultrasonicator, followed by DNA library construction and paired-end sequencing (2 × 150 bp) on an Illumina MiSeq 2000 (Illumina Inc., San Diego, CA, USA) platform by Shandong Huabo Genetic Engineering Co., Ltd. (Jinan, China). Approximately 6–10 gb of raw data for each sample was generated and fed into the NGSQCToolkit v2.3.3 (Patel and Jain, 2012) to conduct sequencing quality controlling. After trimming and filtering, the clean data was assembled and stitched into a synthetic loop using SPAdes 3.9.0 (Bankevich et al., 2012) with the optimized kmer selected by VelvetOptimiser, with candidate values of 93, 95, 97, 103, 105, 107, and 115 (Zerbino and Birney, 2008).

Cp genome annotation was performed by PGA (Qu et al., 2019). Local sequence comparison retrieval (BlastN) database was constructed from the near-source chloroplast genome sequences published in the National Center for Biological Information (NCBI). Exonerate v2.4 (Slater and Birney, 2005) was applied for adjustment and confirmation referred to the chloroplast data and protein-coding gene sequences of close related species, where the parameter setting was 1e^−10^ for the comparison threshold e-value and 70% for protein similarity threshold. Genes, introns, and the boundaries of coding regions were compared with *P. tranlienianum* in *Paphiopedilum*, as reference sequence. The chloroplast genome outline map was visualized using Organellar Genome DRAW v1.2 (Lohse et al., 2007) based on GenBank annotation file and then manually corrected. GC content was analyzed using MEGA v7.0.14 (Kumar et al., 2016).

### Simple sequence repeat analysis

Nine *Paphiopedilum* cp genomes including six species we sequenced and three more deposited in NCBI, i.e. *P. armeniacum* (accession no. KT388109.1) in subgenus *Parvisepalum* (Kim et al., 2015), *P. niveum* (accession no. KJ524105.1) in subgenus *Brachypetalum* (Lin et al., 2015), and *P. dianthum* (accession no. MF983795.1) in sect. *Pardalopetalum* of subgenus *Paphiopedilum* (Hou et al., 2018), which together covered all the subgenera and sections of *Paphiopedilum* genus, were chosen to conduct simple sequence repeat (SSR) analysis and the subsequent genome comparison and sequence divergence analysis. The type and number of SSRs were discriminated by Perl script MISA (Thiel et al., 2003) with critical parameters set as 8, 4, 4, 3, 3, and 3 for mono-, di-, tetra-, penta-, and hexa-nucleotides, respectively. Completely repetitive SSRs were searched to remove redundant results and cyclically arranged or inversely complementary SSRs were treated as the same type.

### Genome comparison and sequence divergence analysis

Multiple sequence alignment of nine *Paphiopedilum* cp genomes was conducted with MAFFT v7.427 (Katoh and Standley, 2013). Subsequently, the aligned sequences were fed into the online tool mVISTA (Frazer et al., 2004) to visualize the percentage of identity. DnaSP v6.12.3 (Rozas et al., 2017) was used to scan nucleotide insertions/deletions (indels) and substitution as well as to calculate nucleotide diversity (Pi) with 600 bp sliding window and 200 bp step length.

The boundaries of four regions (LSC, SSC, and two IRs) of cp genomes were compared using IRscope (Amiryousefi et al., 2018). The divergence of boundary between inverted repeats and single copy regions (IR/SC) among the nine species were analyzed by extracting IR boundary gene information.

After removing the genes that did not exist in some species and the pseudogenes from the protein-coding genes, the remaining genes were termination-codon-eliminated and concatenated. Variable and parsimony-informative sites of the nine cp genomes were calculated using MEGA v7.0.14 (Kumar et al., 2016). To estimate selection pressures, non-synonymous (dN) and synonymous (dS) substitution rates were calculated by the yn00 program in PAML4 (Yang, 2007) after format conversion by DAMBE v7.0.58 (Xia and Xie, 2001). Extreme values (dN > 0.5, dS > 5, and dS < 0.0005) were removed before estimation to avoid biases from saturation of the synonymous rate between related species and the misassignment of orthologous groups.

### Phylogenetic analysis

Twenty-four cp genomes from 20 *Paphiopedilum* taxa, including the six cp genomes obtained in this study and all currently accessible cp genomes of *Paphiopedilum* in the NCBI (http://www.ncbi.nlm.nih.gov/), together with those of other 42 species in five subfamilies (Apostasioideae, Cypripedioideae, Epidendroideae, Orchidoideae, and Vanilloideae) of Orchidaceae (Supplementary Table S1) were aligned by MAFFT v7.427 to construct the phylogenetic tree. The cp genomes of two Liliaceae species, *Lilium regale* (GenBank accession no. MK493302.1) and *Tulipa altaica* (GenBank accession no. MK673755.1), were used as outgroups. The GTR+γ model was adopted to construct the ML tree using RAxML v8 (Stamatakis, 2014). The cp genome sequences of all other species were downloaded from the GenBank database in NCBI under accession nos. listed in Figure 8.

In addition, the complete chloroplast genomes, coding regions, non-coding regions, and 10 hypervariable regions selected by the percentage of identity using mVISTA mentioned above of the nine cp genomes, with *Cypripedium macranthos* (GenBank accession no. NC_024421) as outgroup, were extracted for further exploring genetic relationship. IQTREE software (Kalyaanamoorthy et al., 2017) and ModelTest-NG (Darriba et al., 2020) were utilized to select tree models. Maximum likelihood (ML), maximum parsimony (MP), and Bayesian inference (BI) phylogenetic trees were constructed based on these four different regions using the programs RAxML v8 (Stamatakis, 2014), MEGA v7.0.14 (Kumar et al., 2016), and MrBayes v3.2.7a (Ronquist et al., 2012), respectively. The ML tree, MP tree, and BI tree were merged together to get the final result.

### Divergence time estimation

Divergence times were estimated with BEAST v1.8.0 (Drummond and Rambaut, 2007) based on cp genomes of nine *Paphiopedilum*, four *Cypripedium*, and one *Phragmipedium* species. The GTR + F + G4 model was used based on the best BIC value (1335854.5481) in ModelFinder (Kalyaanamoorthy et al., 2017). BEAST input file was made by BEAUti, a module in BEAST package, with empirical base frequencies, strict clock, and a yule speciation process. We conducted 200,000,000 generations of MCMC simulations and sampled every 20,000 generations, with a burn-in of 1,000 (10%) trees. TRACER v1.5 was used to ensure that the effective sample sizes (ESS) of all the parameters were above 200. The remaining trees were annotated with TreeAnnotator v1.8.0. The phylogenetic chronogram was displayed by FIGTREE v1.4.0 (http://tree.bio.ed.ac.uk/software/figtree/). The calibration age was set to 53 Ma at the divergent time of the most recent common ancestor of *Cypripedium* and *Phragmipedium* (mean = 1, SD = 0.5) with a lognormal distribution, which took into account the ages of the groups estimated in (Ramírez et al., 2007). The lognormal priors consider the errors in the original estimation and thus are appropriate for the calibration point (Ho and Phillips, 2009).

## Results

### Structure and content of *Paphiopedilum* chloroplast genomes

**FIGURE 2:**
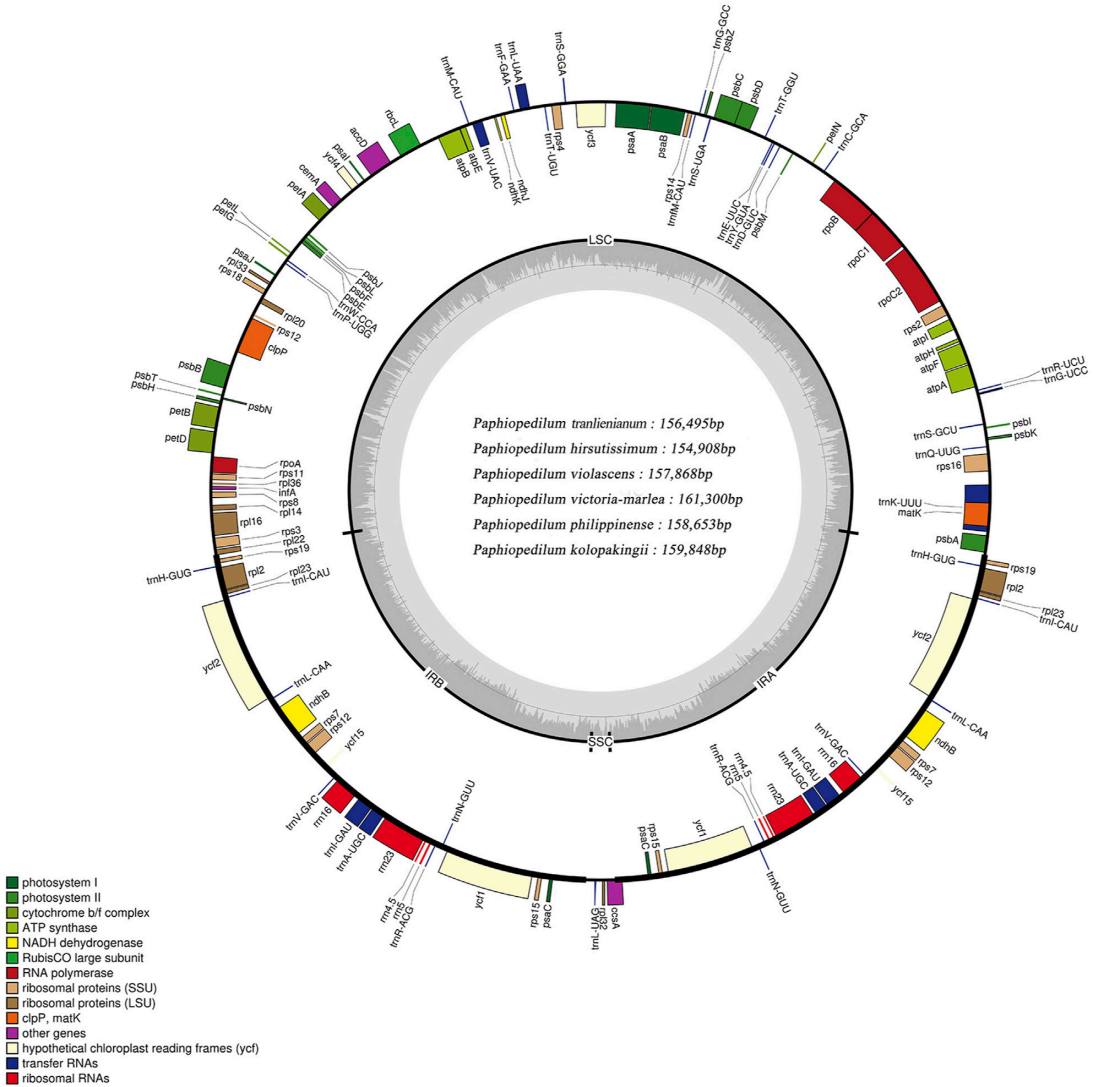
Structure map of the *Paphiopedilum* chloroplast genomes with *P. tranlienianum* as reference. Genes drawn inside the circle are transcribed clockwise, and those outside are transcribed counter-clockwise. Different colored bars indicate genes belonging to different functional groups. Large single copy (LSC), small single copy (SSC), and inverted repeats (IRA, IRB) are indicated. Area dashed darker gray in the inner circle indicates GC content, while the lighter gray area shows AT content of the genome. The intermediate gray line is 50% threshold line.

The cp genomes of six *Paphiopedilum* species were newly sequenced and assembled, and their full sequences have been deposited in GenBank under accession nos. MW794129-MW79134 for *P. tranlienianum*, *P. hirsutissimum*, *P. philippinense*, *P. kolopakingii*, *P. victoria-mariae,* and *P. violascens* in turn. The mean coverage of the six *Paphiopedilum* cp genomes varied from 244.8 × (*P. hirsutissimum*) to 682.1 × (*P. victoria-mariae*). The genome size ranged from 154,908 bp in *P. hirsutissimum* to 161,300 bp in *P. victoria-mariae.* Consistent with that in most higher plants, the six cp genomes all exhibited a typical quadripartite structure, with an LSC region and an SSC region separating two copies of IR regions (Figure 2). The length of the LSC and IR regions ranged from 85,060 bp (*P. hirsutissimum*) to 89,363 bp (*P. victoria-mariae*) and 34,080 bp (*P. violascens*) to 34,856 bp (*P. victoria-mariae*), respectively, while the SSC regions were relatively short, only 524 bp (*P. hirsutissimum*) to 2,225 bp (*P. victoria-mariae*) in length (Table 1).

**TABLE 1:** General characteristics of the six *Paphiopedilum* chloroplast genomes

Species	*P. tranlienianum*	*P. hirsutissimum*	*P. violascens*	*P. victoria-mariae*	*P. kolopakingii*	*P. philippinense*
Genome size (bp)	156,495	154,908	157,868	161,300	159,848	158,653
LSC size (bp)	86,459	85,060	87,659	89,363	88,158	86,837
SSC size (bp)	1,834	524	2,049	2,225	2,054	2,146
IR size (bp)	34,101	34,659	34,080	34,856	34,818	34,835
GC (%)	36.1	36.3	35.5	35.5	35.5	35.7
GC in LSC (%)	33.5	33.8	32.8	32.8	32.8	33.1
GC in SSC (%)	23.9	23.5	21.9	21.7	21.8	21.4
GC in IR (%)	39.5	39.4	39.4	39.3	39.3	39.4
Total number of genes	130	132	129	130	131	131
Protein-coding genes	84	86	83	84	85	85
rRNA genes	8	8	8	8	8	8
tRNA genes	38	38	38	38	38	38
JLB (LSC/IRb)	86460	85061	87660	89364	88159	86838
JSB (IRb/SSC)	120,560	119,719	121,739	124,219	122,976	121,672
JSA (SSC/IRa)	122,395	120,244	123,789	126,445	125,031	123,819
JLA (IRa/LSC)	156,495	154,908	157,868	161,300	159,848	158,653
Chloroplast coverage	366.9×	244.8×	380.4×	682.1×	592.9×	324.2×

The total GC contents of the complete cp genomes were 35.5%–36.3%, which was nearly identical for the six *Paphiopedilum* species. However, there existed an obvious unbalance in different regions (Table 1): the GC content in the IR regions (39.3%–39.5%) was higher than that in the LSC regions (32.8%–33.8%) and SSC regions (21.4%–23.9%). The gene contents and their arrangement of the six cp genomes were relatively conservative, with a little divergence (Figure 2; Table 1). Varied from species, 129 to 132 genes were annotated, including 83 to 86 protein-coding genes, 8 rRNAs, and 38 tRNAs (Table 1). Thirteen genes including five tRNAs (*trnA-UGC*, *trnI-GAU*, *trnK-UUU*, *trnL-UAA*, and *trnV-UAC*) and eight protein-coding genes (*ropC1*, *rps12*, *rps16*, *rpl2*, *rpl16*, *atpF*, *petB*, and *petD*) contained one intron and two genes (*ycf3* and *clpP*) possessed two introns (Supplementary Table S2), which was similar to the situation in *P. dianthum* and other orchid species (Dong et al., 2018; Hou et al., 2018).

Twenty-four genes including four rRNAs (*rrn16*, *rrn23*, *rrn4.5*, and *rrn5*), eight tRNAs (*trnA-UGC, trnH-GUG, trnI-CAU, trnI-GAU, trnL-CAA, trnN-GUU, trnR-ACG,* and *trnV-GAC*), and 12 protein-coding genes (*psaC, ndhB, ndhD, rpl2, rpl23, rpl32, rps12, rps19, rps7, ccsA, ycf1,* and *ycf2*) with two copies were all distributed within the IR regions. In addition, some genes were taxa-specific, e.g. *trnP-GGG* was merely found in *P. hirsutissimum*; *ycf15* only occurred in *P. tranlienianum* and *P. hirsutissimum*; while *ndhC* (pseudo) was particular in *P. niveum*, *P. kolopakingii*, and *P. philippinense*. Conversely, *ndhD* was missing in *P. tranlienianum*, *cemA* was not found in *P. violascens*, and no *infA* appeared in *P. niveum* (Supplementary Table S2). Moreover, *ndhB*, *ndhC*, *ndhD*, *ndhJ*, *ndhK*, and *cemA* genes were annotated as pseudogenes in all the cp genomes we sequenced if any, which was in accordance with that of *P. dianthum*, a species also in *Paphiopedilum*, with the only distinction of *ndhD* located in the IR region instead of SSC region (Hou et al., 2018).

### SSR analysis of *Paphiopedilum* cp genomes

**FIGURE 3:**
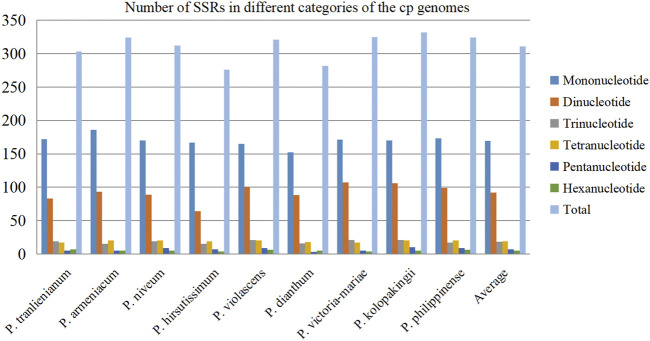
Distribution of different categories of simple sequence repeats (SSRs) in the nine cp genomes.

In recent years, SSRs have been used as important genetic markers to study genetic diversity and evolutionary relationship of species due to their co-dominant inheritance, good stability, high allelic polymorphism, and favorable reproducibility (Kaur et al., 2015; Suo et al., 2016). After scanning SSRs among the nine *Paphiopedilum* cp genomes, a total of 2,799 SSR loci were detected. This batch of SSRs can be categorized into mononucleotide, dinucleotide, trinucleotide, tetranucleotide, pentanucleotide, and hexanucleotide repeats with length ranging from 8 to 132 bp (Supplementary Table S3). The amounts of different types of SSRs varied greatly. Mononucleotide repeats were the most abundant, accounting for over half of all SSRs (54.52%, average 169 for each species), followed by dinucleotide repeats (29.62%, average 92) and tetranucleotide repeats (6.11%, average 19), while pentanucleotide and hexanucleotide repeats owned the least proportion, 2.22% and 1.68%, respectively (Figure 3, Supplementary Table S4). The base composition of the SSRs from mononucleotide to pentanucleotide repeats had A-T preference, e.g. A(8) and T(8) repeats were the most common mononucleotide repeats, and AT(4) plus TA(4) occupied over a half of the dinucleotide repeats (Supplementary Table S3).

There were no significant differences in SSR numbers among the nine *Paphiopedilum* cp genomes, varying from 276 in *P. hirsutissimum* to 332 in *P. kolopakingii* (Figure 3, Supplementary Table S4). However, the quantity of SSRs varied greatly across different structural and functional regions of the cp genomes. Most SSRs were found in the LSC regions and intergenic areas, with an average number of 215 and 175, respectively. The SSC regions and intron areas contained the fewest SSRs, with an average number of 50 and 17, respectively (Table 2). In addition, we found that a certain number of SSRs were located in the gene areas, approaching 30% of the entirety or even more in some taxa, which was obviously higher than that in the intron areas, indicating that SSRs could also participate in coding proteins in *Paphiopedilum* (Table 2, Supplementary Table S5).

**TABLE 2:** Number of SSRs (percentage in the total) in different regions of the cp genomes

	Intergenic	Gene	Intron	LSC	SSC	IRa	IRb	Total
*P. tranlienianum*	169 (55.78%)	93 (30.69%)	41 (13.53%)	207 (68.32%)	6 (1.98%)	45 (14.85%)	45 (14.85%)	303
*P. armeniacum*	189 (58.33%)	91 (28.09%)	44 (13.58%)	221 (68.21%)	11 (3.40%)	46 (14.20%)	46 (14.20%)	324
*P. niveum*	171 (54.81%)	83 (26.60%)	58 (18.59%)	227 (72.76%)	55 (17.63%)	15 (4.81%)	15 (4.81%)	312
*P. hirsutissimum*	136 (49.28%)	92 (33.33%)	48 (17.39%)	184 (66.67%)	40 (14.49%)	26 (9.42%)	26 (9.42%)	276
*P. violascens*	182 (56.70%)	82 (25.55%)	57 (17.76%)	232 (72.27%)	7 (2.18%)	41 (12.77%)	41 (12.77%)	321
*P. dianthum*	160 (56.74%)	79 (28.01%)	43 (15.25%)	190 (67.38%)	4 (1.42%)	44 (15.60%)	44 (15.60%)	282
*P. victoria-mariae*	190 (58.46%)	87 (26.77%)	48 (14.77%)	226 (69.54%)	13 (4.00%)	43 (13.23%)	43 (13.23%)	325
*P. kolopakingii*	200 (60.24%)	85 (25.60%)	47 (14.16%)	223 (67.17%)	7 (2.11%)	51 (15.36%)	51 (15.36%)	332
*P. philippinense*	176 (54.32%)	87 (26.85%)	61 (18.83%)	226 (69.75%)	8 (2.47%)	45 (13.89%)	45 (13.89%)	324
Average	174.8	86.6	49.7	215.1	16.8	39.6	39.6	311
Min.–Max.	136–200	79–93	41–61	184–232	4–55	15–51	15–51	276–332
Total	1573 (56.20%)	779 (27.83%)	447 (15.97%)	1936 (69.17%)	151 (5.39%)	356 (12.72%)	356 (12.72%)	2,799

On the other hand, SSRs were unevenly distributed across different structural parts of the cp genomes. In the SSC region, the density of SSRs was the highest (9.31 SSRs per kb), which was much higher than that in the LSC (2.46 per kb) and IR (1.14 per kb) regions. So, although the SSC regions were incredibly short (1,805 bp on average with the shortest of 524 bp in *P. hirsutissimum*), it could be an important area for designing SSR primers in *Paphiopedilum.*

### Comparative analysis of chloroplast genomes

Comparison of cp genomes showed that 177,090 sites were aligned in nine *Paphiopedilum* taxa, and 136,995 sites remained after excluding alignment gaps; 8,699 variable sites (4.91%) were detected across the cp genomes, among which 4,521 were polymorphic (segregating) sites, and 1,890 (21.73% of the variable sites) were parsimony informative sites (Table 3). The genome-wide nucleotide diversity *pi* and *theta* (*θ*) were 0.00896 and 0.01257, respectively, by using the sliding window method with 600-bp window length and 200-bp step size. A common feature of these *Paphiopedilum* cp genomes was that the IR regions were substantially more conserved than the LSC and SSC regions, with the LSC and SSC regions containing 1,533 (23.18%) and 208 (27.84%) informative sites, respectively, with only 116 (13.81%) in the IR regions. A large proportion of information sites lead to the highest nucleotide diversity (0.02524) in the SSC region (Table 3), which could be used as an important basis for the research of the interspecific genetic relationship among *Paphiopedilum* plants.

**TABLE 3:** Variable and informative site analyses in the nine *Paphiopedilum* cp genomes

	Number of sites	Number of variable sites	Number of informative sites	Nucleotide diversity
Large single copy region	99697	6614 (6.63%)	1533 (23.18%)	0.01237
Small single copy region	41573	747 (1.80%)	208 (27.84%)	0.02524
Inverted repeat region	36689	840 (2.29%)	116 (13.81%)	0.00325
Complete cp genome	177,090	8699 (4.91%)	1890 (21.73%)	0.00896

**FIGURE 4:**
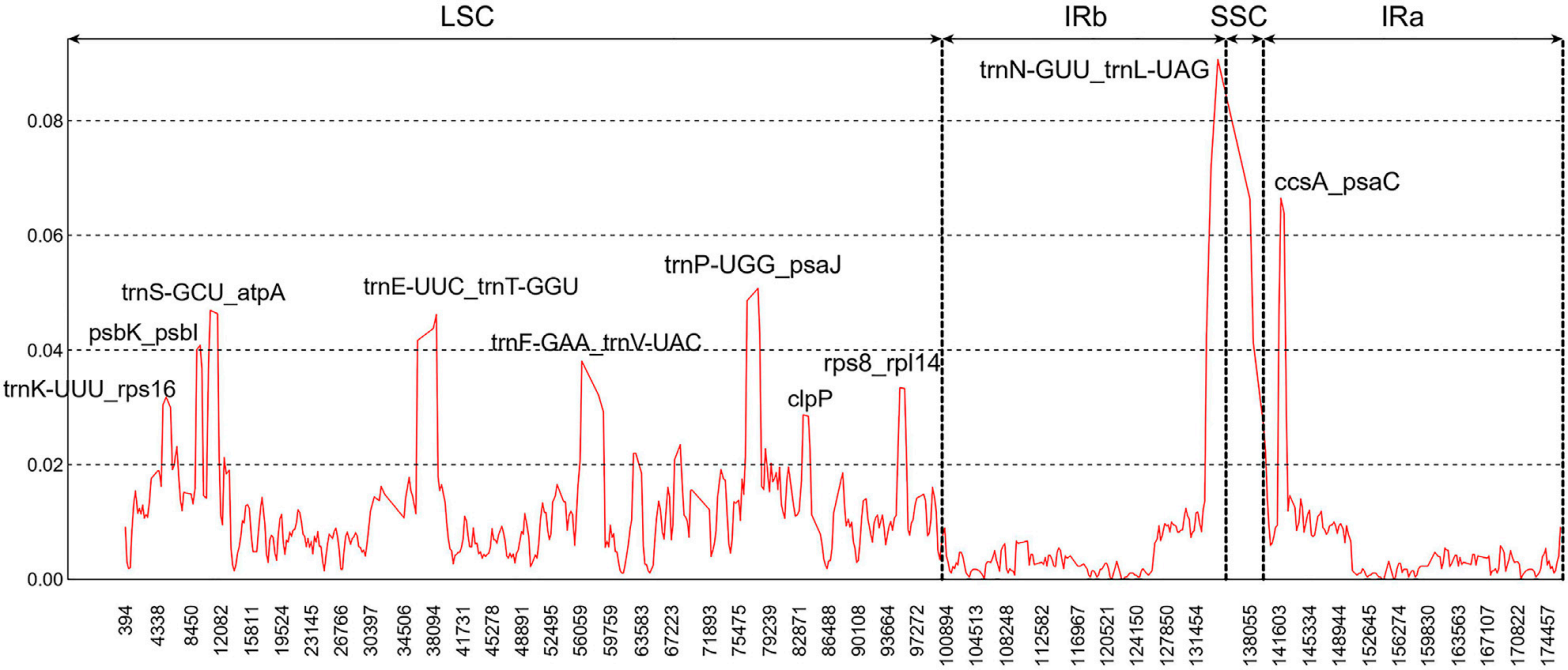
Sliding window analysis of the nine *Paphiopedilum* chloroplast genomes (window length: 600 bp, step size: 200 bp). x-Axis, the window midpoint; y-axis, nucleotide diversity (Pi).

In addition, ten hypervariable regions were uncovered by sliding window analysis, including one gene region (*clpP*) and nine intergenic regions (*trnK-UUU-rps16*, *psbK-psbI*, *trnS-GCU-atpA*, *trnE-UUC-trnT-GGU*, *trnF-GAA-trnV-UAC*, *trnP-UGG-psaJ*, *rps8-rpl14*, *trnN-GUU-trnL-UAG*, and *ccsA-psaC*). Most of them existed in the LSC regions (Figure 4). These regions could act as a batch of potential candidate markers for further *Paphiopedilum* infrageneric phylogenetic analysis and affinis identification.

Based on the MAFFT multiple sequence alignment results, Perl scripts were used to count nucleic acid insertions/deletions (indels) and substitutions. Data analysis showed that the number of indels and nucleotide substitutions ranged from 400 to 889 and 702 to 3,092, respectively. Among these taxa, *P. kolopakingii* and *P. philippinense* had the tiniest differentiated degree with 400 indels and 702 nucleotide substitutions between them (Table 4), which was identical to the fact that they belonged to the same section, *Coryopedilum and P. armeniacum* showed the highest interspecific variability, which had 889 indels when compared to *P. violascens* or *P. philippinense* and 3,092 nucleotide substitutions when compared to *P. dianthum*.

**TABLE 4:** Number of insertions/deletions (indels) and nucleotide substitutions in the nine *Paphiopedilum* chloroplast genomes

	*P. victoria-mariae*	*P. armeniacum*	*P. dianthum*	*P. tranlienianum*	*P. violascens*	*P. niveum*	*P. kolopakingii*	*P. hirsutissimum*	*P. philippinense*
*P. victoria-mariae*	—	824	641	550	572	662	604	597	620
*P. armeniacum*	2,768	—	866	854	889	622	887	870	889
*P. dianthum*	1,680	3,092	—	711	746	753	564	692	563
*P. tranlienianum*	1,270	2,620	1841	—	594	731	688	564	712
*P. violascens*	1,472	2,864	1939	1,283		765	745	600	749
*P. niveum*	2045	2,719	2,530	2,123	2,193	—	756	730	762
*P. kolopakingii*	1,338	2,750	1,472	1,371	1,548	2091	—	707	400
*P. hirsutissimum*	1,521	2,583	1780	1,401	1,409	2052	1,631	—	713
*P. philippinense*	1,436	2,803	1,600	1,394	1,629	2,190	702	1,668	—

The upper triangle represents the number of Indels, and the lower triangle represents the number of nucleic acid substitutions.

**FIGURE 5:**
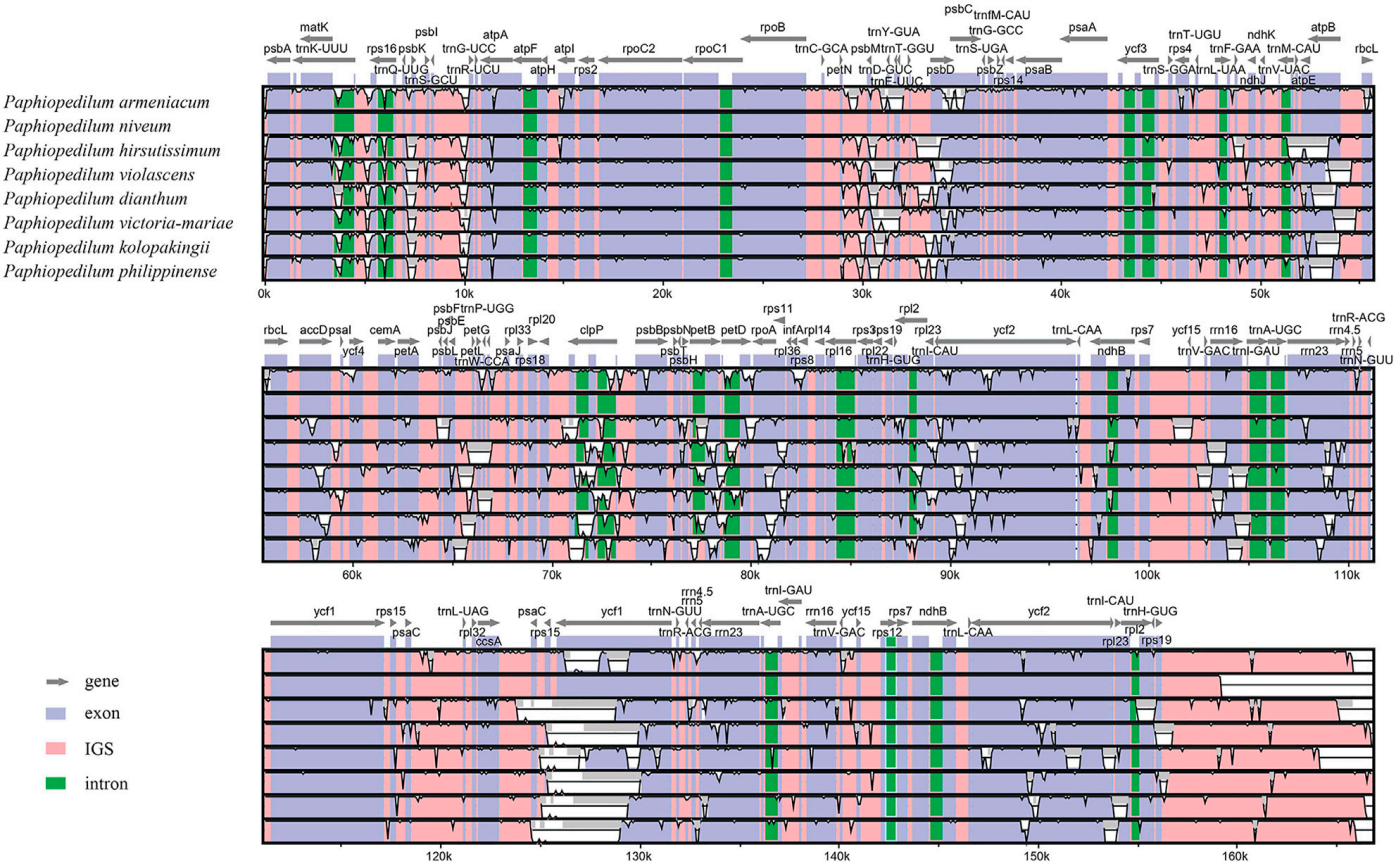
Comparison of nine *Paphiopedilum* chloroplast genomes using *P. tranlienianum* as a reference sequence with a 50% identity cutoff. Gray arrows show the position and direction of each gene. The colored areas indicate the exon, intron, and intergenic spacer (IGS) sequences. The vertical axis indicates the sequence identity, ranging from 50% to 100%.

**FIGURE 6:**
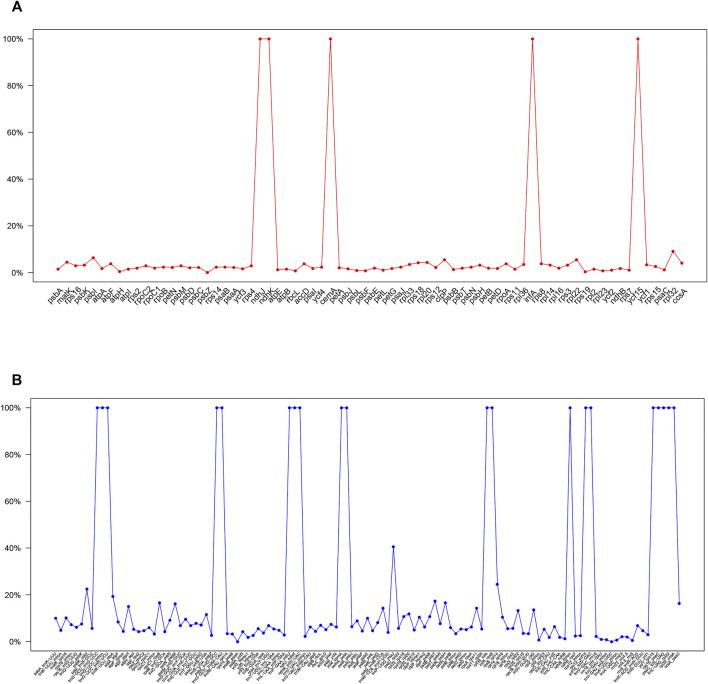
Percentages of variable sites in homologous regions across the 9 *Paphiopedilum* species with complete chloroplast genomes. **(A)** protein coding sequences (CDS). **(B)** non-coding sequences.

The divergent regions of the nine *Paphiopedilum* cp genomes were analyzed by mVISTA (Figure 5), which showed that there were less than 15% variations between most of them. The proportions of variable sites in the non-coding region (introns and intergenic spacers) were 6.69% on the whole, more than twice as high as in the coding regions (2.38%). Among them, five coding regions (*ndhJ, ndhK*, *cemA*, *infA*, and *ycf15*) and 20 non-coding regions (*trnS-GCU-trnG-UCC*, *trnG-UCC* intron, *trnG-UCC-trnR-UCU*, *psbZ-trnG-GCC*, *trnG-GCC-trnfM-CAU*, *trnF-GAA-ndhJ*, *ndhJ-ndhK*, *ndhK-trnV-UAC*, *ycf4-cemA*, *cemA-petA*, *rpl36-infA*, *infA-rps8*, *ndhB* intron, *rps12-ycf15*, *ycf15-trnV-GAC*, *ycf1-rps15*, *rps15-psaC*, *psaC-trnL-UAG*, *trnL-UAG-rpl32*, and *rpl32-ccsA*) showed the highest variation (reached 100%) due to fragment indel in some species, followed by *trnP-UGG-psaJ* (40.62%) (Figure 6, Supplementary Tables S6, 7).

Eliminating the genes that did not exist in some species and the pseudogenes from protein-coding genes, the remaining gene sequences were concatenated after the termination codon was removed. MEGA v7.0.14 and the Codon mode of the Muscle algorithm were used for comparison. After format conversion, non-synonymous replacement rate (dN) and synonymous replacement rate (dS) were calculated with yn00 subprogram of PAML. The minimum value of dN appeared between *P. victoria-mariae* and *P. kolopakingii* (0.0006), the minimum value of dS occurred between *P. victoria-mariae* and *P. tranlienianum* (0.0036), and the maximum value of dN and dS both happened between *P. armeniacum* and *P. niveum* with 0.076 and 0.0217, respectively, indicating that there was a conspicuous variation between them at nucleotide level (Table 5). Overall, dN was lower than dS, with the ratio (dN/dS) of 0.1268–0.6081 appearing between *P. victoria-mariae* and *P. kolopakingii*, *P. emersonii* and *P. niveum*, respectively, implying that the whole cp genomes in *Paphiopedilum* were probably in a state of purifying selection (Table 5).

**TABLE 5:** Pairwise substitution rates (dN/dS) between the *Paphiopedilum* cp genomes based on protein-coding gene sequences

	*P. victoria-mariae*	*P. armeniacum*	*P. dianthum*	*P. tranlienianum*	*P. violascens*	*P. niveum*	*P. kolopakingii*	*P. hirsutissimum*
*P. victoria-mariae*	—	—	—	—	—	—	—	—
*P. armeniacum*	0.1855 (0.0034/0.0181)	—	—	—	—	—	—	—
*P. dianthum*	0.2683 (0.0014/0.0052)	0.2109 (0.0041/0.0196)	—	—	—	—	—	—
*P. tranlienianum*	0.4234 (0.0015/0.0036)	0.2389 (0.0044/0.0184)	0.4443 (0.0022/0.0050)	—	—	—	—	—
*P. violascens*	0.2050 (0.0014/0.0068)	0.1851 (0.0040/0.0216)	0.2471 (0.0020/0.0082)	0.3269 (0.0021/0.0064)	—	—	—	—
*P. niveum*	0.5117 (0.0061/0.0118)	0.3478 (0.0076/0.0217)	0.5210 (0.0069/0.0131)	0.6081 (0.0069/0.0114)	0.4536 (0.0066/0.0145)	—	—	—
*P. kolopakingii*	0.1268 (0.0006/0.0050)	0.1702 (0.0034/0.0198)	0.2417 (0.0010/0.0042)	0.3173 (0.0015/0.0048)	0.1583 (0.0013/0.0080)	0.4675 (0.0060/0.0127)	—	—
*P. hirsutissimum*	0.2467 (0.0013/0.0054)	0.1919 (0.0039/0.0202)	0.2516 (0.0017/0.0068)	0.3936 (0.0020/0.0050)	0.2008 (0.0016/0.0082)	0.4955 (0.0066/0.0132)	0.1921 (0.0013/0.0066)	—
*P. philippinense*	0.2080 (0.0013/0.0064)	0.1856 (0.0039/0.0212)	0.3057 (0.0017/0.0056)	0.3580 (0.0022/0.0062)	0.2087 (0.0020/0.0094)	0.4701 (0.0067/0.0142)	0.1964 (0.0008/0.0042)	0.2455 (0.0020/0.0080)

### Contraction and expansion of inverted repeats

**FIGURE 7:**
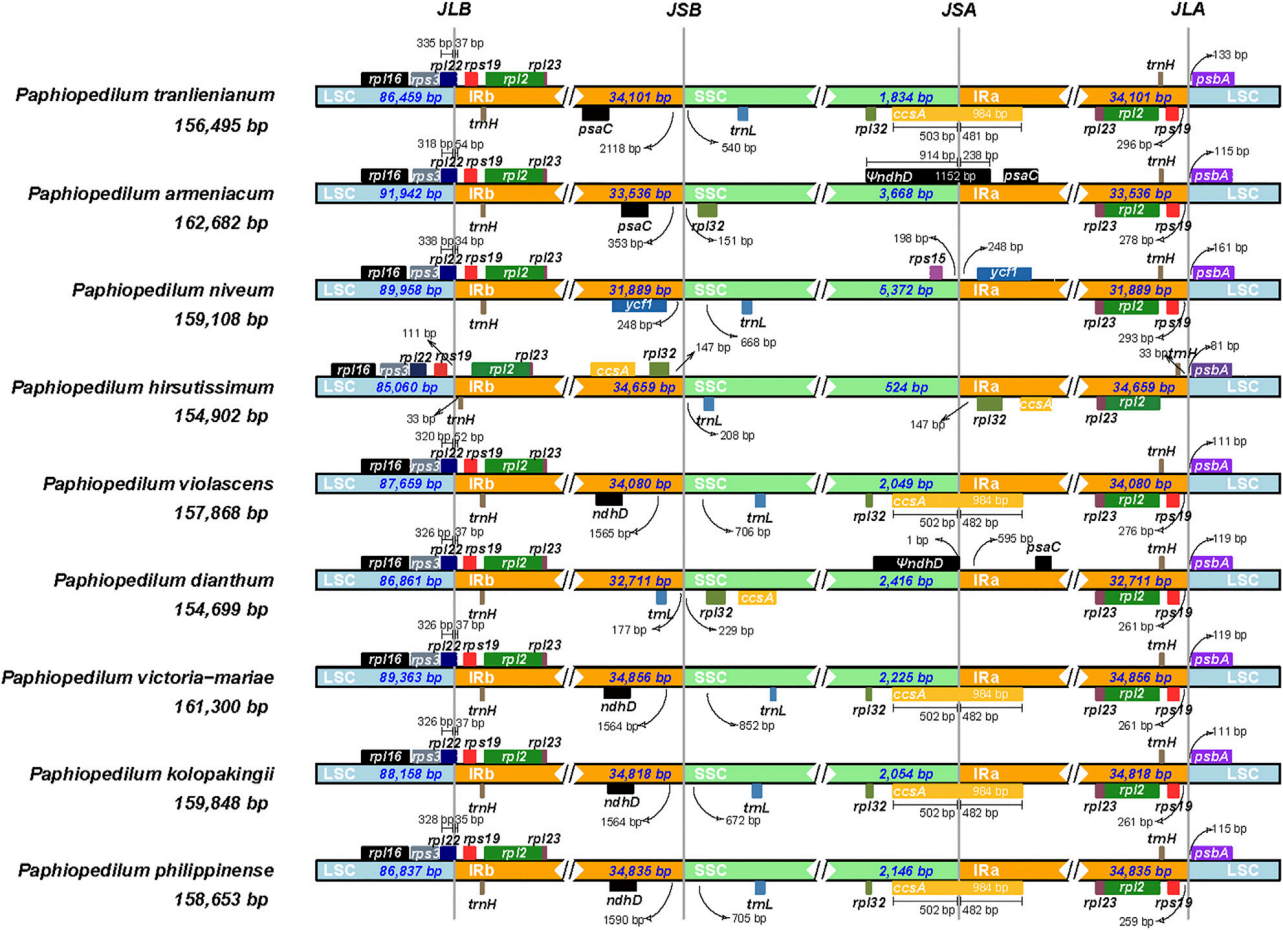
Comparison of junctions between single-copy and inverted repeat regions in the chloroplast genomes of nine *Paphiopedilum* species. The genes transcribed from right to left are depicted on the top of their corresponding locus; genes transcribed from left to right are depicted below. The arrows indicate the distance between the start or end of a given gene and the corresponding junction site. JLB (LSC/IRb), JSB (IRb/SSC), JSA (SSC/IRa), and JLA (IRa/LSC) denote four junctions between the two single-copy regions (LSC and SSC) and the two IRs (IRa and IRb).

The contraction and expansion of inverted repeat regions had been proved to be the major reason resulting in the size variation of the chloroplast genome and to play vital roles in evolution (Goulding et al., 1996). To clarify the mechanism of the cp genome variation in *Paphiopedilum*, a comprehensive comparison of four junctions (JLB, JSB, JSA, and JLA) between the two single-copy regions and the two IR regions of nine representative *Paphiopedilum* species was performed by analyzing IR border positions and adjacent genes (Figure 7). Overall, the LSC/IR boundaries including JLB (LSC/IRb) and JLA (IRa/LSC) were relatively stable in the genus *Paphiopedilum*, while the SSC/IR boundaries, i.e. JSB (IRb/SSC) and JSA (SSC/IRa), varied drastically among species. For instance, except for *P. hirsutissimum* in which the JLBs were located between *rps19* and *trnH*, all other *Paphiopedilum* species located their JLBs on the *rpl22* gene, with the IRb region including 34 to 54 bp of the gene. Likewise, the JLA of all *Paphiopedilum* species were located between *rps19* and *psbA* (259–296 bp after the end of *rps19*), except for *P. hirsutissimum* in which the JLAs were located between *trnH* and *psbA* due to shifting of the *rps19* gene from the IR regions into the LSC region (Figure 7). As for the JSB and JSA, six types could be categorized among the nine taxa: in *P. violascens*, *P. victoria-mariae, P. kolopakingii*, and *P. philippinense*, the JSB was located between the *ndhD* and *trnL* gene (1,564–1,590 bp away from the end of *ndhD*) and the JSA was located within the *ccsA* gene, with 502 bp of the gene included in the SSC region; in *P. tranlienianum*, the JSB was located between *psaC* and *trnL* (2,118 bp after the end of *psaC*), and the JSA was located in *ccsA*; in *P. armeniacum*, the JSB was located between *psaC* and *rpl32* (353 bp after the end of *psaC*), and the JSA was located in the pseudo *ndhD* gene; in *P. niveum*, the JSB was located between *ycf1* and *trnL*, and the JSA was located between *rps15* and *ycf1*; in *P. dianthum,* the JSB was located between *trnL* and *rpl32*, and the JSA was located before the pseudo *ndhD* gene; and in *P. hirsutissimum*, the SSC region was extremely short (524 bp), containing only one gene, *trnL*.

### Phylogenetic relationship and divergent time estimate

**FIGURE 8:**
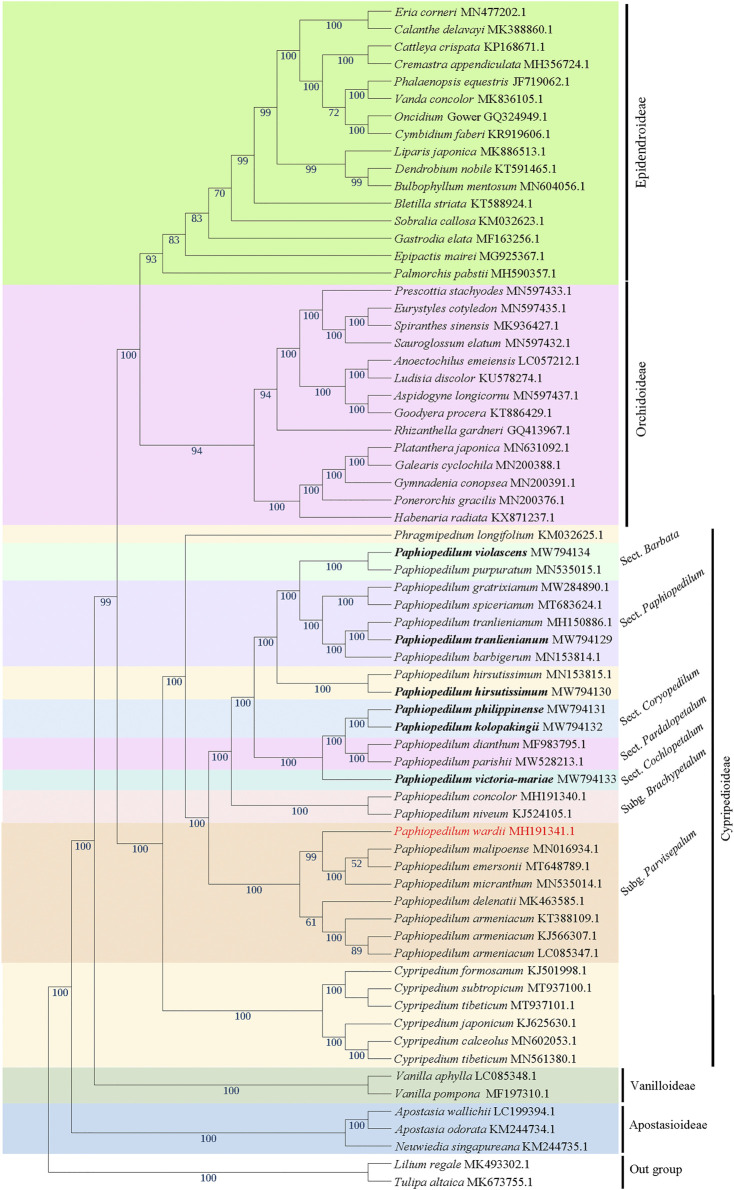
The ML phylogenetic tree based on 66 complete chloroplast genome sequences from Orchidaceae, with *Lilium regale* and *Tulipa altaica* as outgroup. The six taxa we sampled in this study are highlighted in bold. Bootstrap values with 1,000 replicates are indicated at nodes. The GenBank accession numbers of the cp genomes are listed behind the Latin names of the species. The species that may be mistaken in name is marked in red.

The phylogenetic tree based on 66 cp genomes of the Orchidaceae species, including the six *Paphiopedilum* cp genomes we sequenced, all currently accessible cp genomes from subfamily Cypripedioideae, and those from representative species of other subfamilies in NCBI, was constructed with maximum likelihood (ML) method, with two Liliaceae species as outgroup. The result indicated that all Orchidaceae species were grouped into five monophyletic clades (Figure 8), corresponding to the five subfamilies of Orchidaceae (Apostasioideae, Vanilloideae, Cypripedioideae, Orchidoideae, and Epidendroideae), which was consistent with the backbone of Orchidaceae in the current APG IV taxonomy (The Angiosperm Phylogeny Group et al., 2016). In the subfamily Cypripedioideae, two subgroups were separated, corresponding to the tribe Cypripedieae and Paphiopedileae (including *Phragmipedium* and *Paphiopedilum*), respectively. The six species we sampled were all clustered into the *Paphiopedilum* genus with 100% bootstrap support values (Figure 8). According to the branches in *Paphiopedilum*, three subclades representing the subgenus *Parvisepalum*, *Brachypetalum*, and *Paphiopedilum*, respectively, could be distinguished as monophyly, and five sections in the subgenus *Paphiopedilum* were clustered separately in their own lineages: *P. violascens* and *P. purpuratum* were clustered in sect. *Barbata*; *P. tranlienianum* together with *P. barbigerum*, *P. gratrixianum,* and *P. spicerianum* in sect. *Paphiopedilum*; *P. kolopakingii* and *P. philippinense* in sect. *Coryopedilum*; *P. dianthum* and *P. parisii* in sect. *Pardalopetalum*; and *P. victoria-mariae* alone in sect. *Cochlopetalum* (Figure 8). Unexpectedly, the two cp genomes of *P. hirsutissimum* that were categorized in sect. *Paphiopedilum* by morphology were clustered as a paraphyly to sect. *Paphiopedilum* plus sect. *Barbata* with high bootstrap value (100%); in addition, another species that should belong to sect. *Barbata* in *Paphiopedilum* subgenus, *P. wardii*, was clustered in subgenus *Parvisepalum* (Figure 8).

**FIGURE 9:**
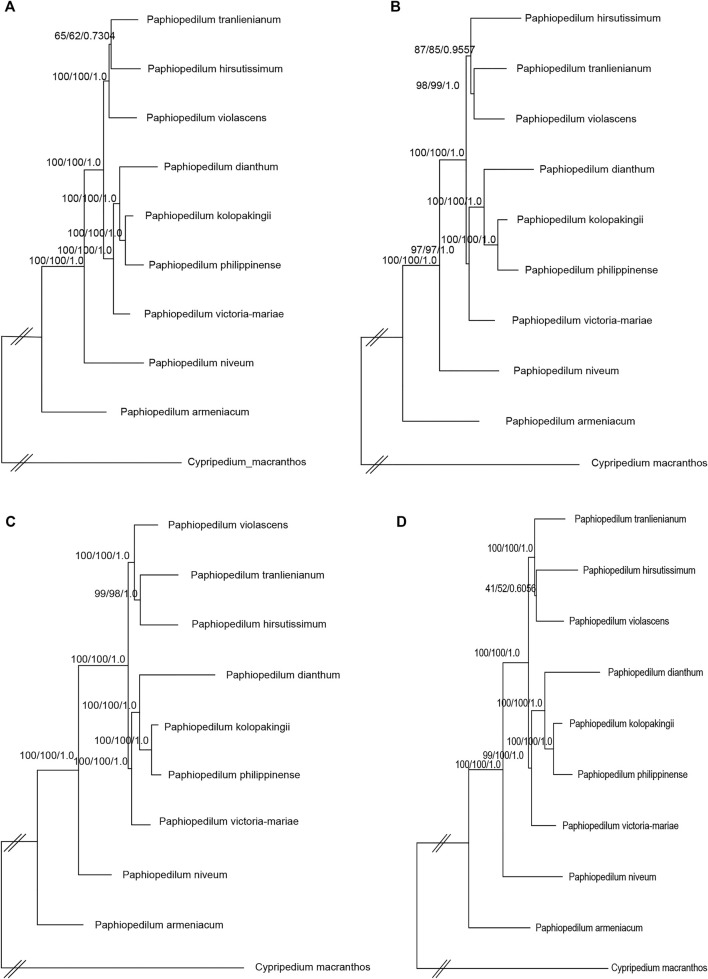
Phylogenetic relationships of the nine *Paphiopedilum* taxa and one *Cypripedium* taxon constructed by each of the four DNA sequence alignment datasets, including whole cp genomes **(A)**, coding regions **(B)**, non-coding regions **(C)**, and ten hypervariable regions **(D)** using maximum parsimony (MP), maximum likelihood (ML), and Bayesian inference (BI) methods, respectively. ML topology is shown with MP bootstrap support values/ML bootstrap support value/Bayesian posterior probability listed at each node.

To further figure out the backbone phylogeny of *Paphiopedilum*, besides whole cp genome sequences, the coding regions, non-coding regions, and 10 hypervariable regions were extracted, respectively, to construct the infrageneric phylogenetic tree for 10 species, including nine *Paphiopedilum* taxa and one *Cypripedium* taxon, with maximum parimony (MP), maximum likelihood (ML), and Bayesian Inference (BI) methods, respectively. The backbone structure constructed with these four datasets was substantially consistent and in accordance with the phylogenetic relationship based on 66 Orchidaceae cp genomes mentioned above, except a slight conflict on species of *P. tranlienianum*, *P. hirsutissimum*, and *P. violascens* (Figure 9)*. P. hirsutissimum* showed a closer genetic relationship to *P. violascens* than to *P. tranlienianum* in the phylogenetic tree with the whole cp genome data (Figure 9A) but exhibited a closer relationship to *P. tranlienianum* than to *P. violascens* in phylogenetic trees with the sequences of coding regions or the 10 hypervariable regions (Figures 9B,D). Based on the non-coding sequences, however, the phylogenetic tree showed that *P. tranlienianum* and *P. violascens* were closer in genetic relationship (Figure 9C), which was identical to the result with 66 Orchidaceae cp genomes (Figure 8).

**FIGURE 10:**
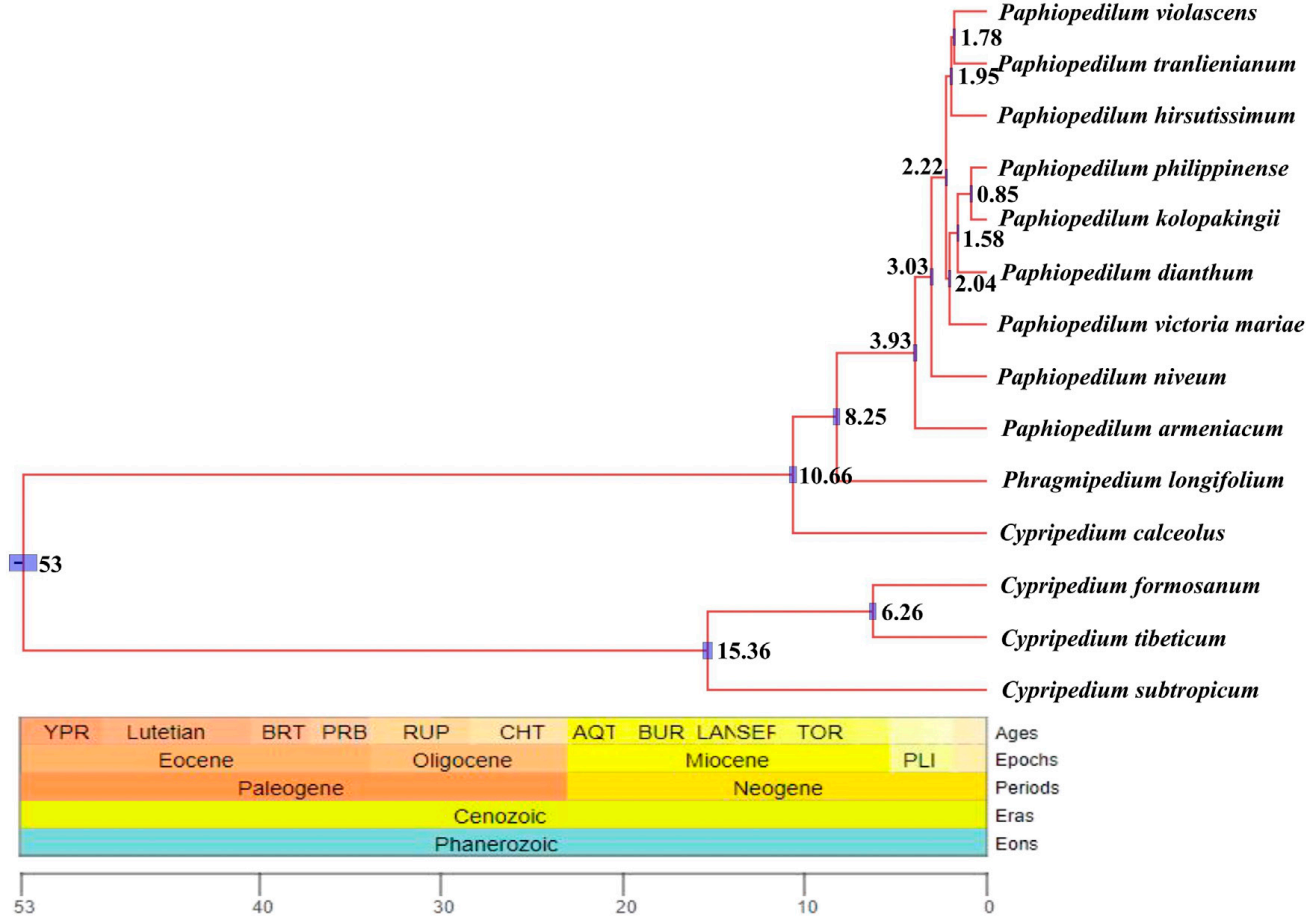
Divergent time estimate of the nine *Paphiopedilum* taxa and *Cypripedium* taxa constructed by whole cp genomes. The branch length of cladogram reflected the divergent time, and the number beside the node denoted the node age, with the purple bar as 95% highest probability density (HPD). The posterior probabilities were 100% for all the clades.

Divergent time estimate showed that the divergent time between genus *Paphiopedilum* and *Phragmipedium* were dated back to the Late Miocene (8.25 Ma), whereas that of the most current ancestor of genus *Paphiopedilum* was at Early Pliocene (3.93 Ma). Nearly most of the species in genus *Paphiopedilum* were diverged within 4 Ma, which hinted that this genus might go through a rapid radiation. According to the cladogram, one species in *Cypripedium* was split away off the *Cypripedium*’*s* clade and grouped together with *Phragmipedium*, which may be due to long branch attraction (Figure 10).

## Discussion

### The evolution of *Paphiopedilum* cp genomes

In this study, six cp genomes of *Paphiopedilum* species were sequenced and annotated. The size of these cp genomes varied from 154,908 bp (*P. hirsutissimum*) to 161,300 bp (*P. victoria-mariae*), which was larger than the average cp genome size of 150 kb for most flowering plants (Ruhlman and Jansen, 2014). Overall, the cp genomes of *Paphiopedilum* species showed high similarity in gene composition, GC content and other aspects (Table 1), but their SSC regions were much shorter (524 bp–2,225 bp) than other orchid species, such as *Cremastra appendiculata* (15,478 bp), *Calanthe davidii* (15,672 bp) and *Plathathera japonica* (13,664 bp) (Dong et al., 2018). Compared to other sequenced plastomes of orchid, such as *Cymbidium* (Yang et al., 2013), *Dendrobium* (Shrestha et al., 2019), *Holcoglossum* (Kim et al., 2020), and Aeridinae (Chris Blazier et al., 2011), *Paphiopedilum* cp genomes showed larger variation of size at genus level, which were mainly due to IR expansion. The IR regions of *Paphiopedilum* cp genomes we sequenced were 34,080 bp (*P. violascens*) to 34,856 bp (*P. victoria-mariae*), evidently larger than that (∼25 kb) of most angiosperms (Ruhlman and Jansen, 2014), including the majority of orchid species (Shrestha et al., 2019; Kim et al., 2020). A large-scale IR expansion over several kb had also been reported in a number of other angiosperms, such as *Mahonia* (Ma et al., 2013), *Passiflora* (Shrestha et al., 2019), *Pelargonium* (Chumley et al., 2006; Weng et al., 2017), *Plantago* (Zhu et al., 2016), and some Fabaceae species (Dugas et al., 2015; Wang et al., 2017; Wang et al., 2018). Different from that in *Pelargonium* where IR expanded towards both the SSC and LSC regions (Chumley et al., 2006), the IR expansion in *Paphiopedilum* mainly trended in the SSC region, which was similar to those found in a few other orchid species including *Vanilla* (Lin et al., 2015; Niu et al., 2017), *Pogonia*, and *Hetaeria* (Kim et al., 2020), as well as in some other monocots (Martin et al., 2013; Shetty et al., 2016).

In addition, we found that protein-coding genes in the *ndh* family varied among *Paphiopedilum* species. In higher plants, there are 11 *ndh* genes (*ndhA-K*) in the cp genomes encoding nicotinamide-adenine dinucleotide (NADH) dehydrogenase subunits, which associates with nuclear-encoded subunits to form the NADH dehydrogenase-like (NDH) complex involving in cyclic electron flow around photosystem I (PSI) and chlororespiration (Ruhlman and Jansen, 2014; Ruhlman and Jansen, 2021). Although chloroplast NDH complex mediates the cyclic electron transport in PSI, no deleterious effects have been observed in *ndh*-deficient mutants or transgenics under favorable growth conditions (Yang et al., 2013), indicating that chloroplast *ndh* genes might be dispensable in autotrophic plants. Indeed, loss or pseudogenization of plastid *ndh* genes has been found in diverse lineages of photoautotrophic seed plants (Chris Blazier et al., 2011; Kim et al., 2020). In *Paphiopedilum*, we found all species investigated lack *ndhA*, *ndhE*, *ndhF*, *ndhG*, *ndhH*, and *ndhI* genes, and all the existed *ndh* genes in some species including *ndhB*, *ndhC*, *ndhD*, *ndhJ*, and *ndhK* are pseudogenes (Supplementary Table S2). *ndh* deletion and pseudogenization were assumed to be a widely occurring phenomenon in Orchidaceae family (Amiryousefi et al., 2017); however, full *ndh* genes have also been reported in some orchids, including *Cypripedium* (Lin et al., 2015; Guo et al., 2021b), a genus belonging to the same subfamily (Cypripedioideae) as *Paphiopedilum*. Given that all *ndh* genes were also lost or pseudogenized in *Phragmipedium longifolium* belonging to Cypripedioideae (Kim et al., 2015), we inferred that the loss/pseudogenization of *ndh* genes might occur after the divergent of *Paphiopedilum* from *Cypripedium* but before the separation of *Paphiopedilum* and *Phragmipedium*.

Besides IR expansion, the SSC region of *Paphiopedilum* might have experienced gene rearrangement. In most angiosperms, there are two gene clusters in the SSC region, i.e. *rpl32*(+)*-trnL-UAG*(+)*-ccsA*(+) and *ndhD*(-)-*psaC*(-)-*rps15*(-) (Kim and Chase, 2017). In *Paphiopedilum*, the original *ndhD*(-)-*psaC*(-)-*rps15*(-) linkage was preserved in most species, except for *P. tranlienianum* (losing *ndhD*) and *P. armeniacum* in which *ndhD*(-) was inversed to *ndhD*(+), although they were mostly shifted into the IRa region, while the *rpl32*(+)*-trnL-UAG*(+)*-ccsA*(+) linkage was only retained in *P. armeniacum* (Supplementary Figure S1). In other species of the genus, the *rpl32*(+)*-trnL-UAG*(+)*-ccsA*(+) fragments were changed into *trnL-UAG*(+)*-rpl32*(+)*-ccsA*(+), which indicated that there was gene recombination in the SSC region.

### Sequence divergence and mutation hotspot

Sequence diversity analysis revealed that the whole cp genomes of *Paphiopedilum* species were relatively conservative. Most non-coding regions (introns and intergenic spacers) had less than 10% variation (Figure 6B, Supplementary Table S7), and majority of the coding regions had less than 5% variation (Figure 6A, Supplementary Table S6). The overall nucleotide diversity of the non-coding region (6.69%) was more than twice as much as that of the coding region (2.38%), and the LSC and SSC regions had more variation than the IR regions (Table 3), which was in accordance with the results in most other species (Smith, 2015; Dong et al., 2018; Gu et al., 2019). There were five coding regions and 20 non-coding regions that exhibited extremely high variation (100%) in *Paphiopedilum* cp genomes (Figure 6, Supplementary Table S6, 7), and the noncoding regions *trnP_psaJ*, *rps8_rpl14*, and *psbK_psbI* showed relatively high variation (>20%). Interestingly, these high variable regions were mostly different from the mutational hotspots identified in other orchid genera or the markers used for identifying species (Yang et al., 2013; Luo et al., 2014; Niu et al., 2017), suggesting that an evolutionarily conserved locus in one orchid genus may be a high variable locus in another genus. These sequences can be used as important references for future studies on the evolution and diversity in specific genus.

Generally, SSRs consisting of one to six nucleotide repeat units were widely distributed across the entire genome and may have a significant impact on recombination and rearrangement of the genome (Cavalier-Smith, 2002; Vieira et al., 2016). SSRs in the cp genome can be highly variable at the inter-specific and even intra-specific level, and so are usually used as genetic markers in evolutionary studies (Vieira et al., 2016; Xu et al., 2017; Dong et al., 2018). In this study, a total of 2,799 SSRs were detected throughout the nine *Paphiopedilum* cp genomes, among which the mononucleotide and dinucleotide repeats were the most common (Figure 3, Supplementary Table S4), similar to the results reported in other orchids (Kang et al., 2015; Dong et al., 2018). In addition, the distribution of SSRs in different regions varied considerably, with the majority contained in the LSC regions and the intergenic regions, but the density of SSRs was the highest in the SSC region (Table 2). Dong et al. (2018) identified 233 SSRs in four orchid species (*Cremastra appendiculata*, *Calanthe davidii*, *Epipactis mairei*, and *Platanthera japonica*), and found 77.68% of SSRs were distributed in the intergenic and intron regions, suggesting the preferential appearance of SSRs in intergenic region may be a common feature in Orchidaceae.

Previously, a series of cp fragments have been recommended as plant barcodes, such as the coding regions *accD*, *matK*, *rbcL*, *rpoC1*, *rpoC2*, and *ycf1*, and noncoding regions *atpF-atpH*, *atpI-atpH*, *psbK-psbI*, *trnH-psbA*, and *trnL* intron, because of their relatively high degree of variation (Kress et al., 2005; Guo et al., 2016). Moreover, the combination of *rbcL* and *matK* was recommended as a core plant barcode (CBOL Plant Working Group, 2009). In *Paphiopedilum*, however, the resolution for species identification of these loci was not high enough, with the highest accuracy (28.97%) for the combination of *matK* and *atpF-atpH*. In this study, 10 hypervariable regions of *Paphiopedilum* cp genome, including one gene region and nine intergenic regions (Figure 4), were discovered by sliding window analysis, which had higher variability than the abovementioned frequently-used barcode. For example, the variability percentage of the clpP-encoding region was 5.55%, higher than that of the matK-encoding region (4.52%) (Supplementary Table S6); seven of the nine hypervariable intergenic regions also showed higher variability percentage (16.33%–100%) than the *atpF*-*atpH* region (15%) (Supplementary Table S7). The results indicated that these hypervariable regions may have better resolution for species identification than the common barcodes previously reported. Further study is needed to determine which highly variable sites or SSR locus can efficiently distinguish different species in *Paphiopedilum.*

### Phylogenetic relationship

According to Chase et al. (2015), the Orchidaceae family was classified into five subfamilies (Apostasioideae, Vanilloideae, Cypripedioideae, Orchidoideae, and Epidendroideae), with two, four, and 16 tribes included in Vanilloideae, Orchidoideae, and Epidendroideae subfamily, respectively. In this study, a maximum likelihood (ML) tree was constructed with 66 complete cp genome sequences from 61 representative orchid species of five subfamilies and 14 of 22 tribes, using two cp genomes from Liliaceae as outgroup. The phylogenetic tree divided the five subfamilies as monophyly with high bootstrap values, supporting an evolutionary order of Apostasioideae-Vanilloideae-Cypripedioideae-Orchidoideae-Epidendroideae; likewise, the tribes or genera in the subfamily were clustered as monophyly with high bootstrap values (Figure 8), which was overall consistent with the results reported previously (Givnish et al., 2015; Amiryousefi et al., 2017; Niu et al., 2017). However, the ML tree constructed recently by Dong et al. (2018) with 50 complete cp genomes of orchids demonstrated that Orchidoideae was a nested other than a monophyletic subfamily; the phylogenetic tree constructed with 38 protein-coding genes of mitochondrial genome from 74 orchids by Dugas et al. (2015), Li et al. (2019) placed Cypripedioideae at an evolutionary status predating Vanilloideae. In addition, the phylogenetic relationships of some tribes in Epidendroideae were variable from different studies, which may be due to the differences in the collected samples used (Dugas et al., 2015; Givnish et al., 2015; Niu et al., 2017; Dong et al., 2018).

Within Cypripedioideae, three monophyletic genera could be demarcated, with *Paphiopedilum* and *Phragmipedium* having a closer relationship, which was congruent with morphological classification (Cox et al., 1997). According to previous studies, *Paphiopedilum* can be divided into three subgenera, *Parvisepalum*, *Brachypetalum*, and *Paphiopedilum*, and the latter can be further subdivided into five sections: *Paphiopedilum*, *Barbata*, *Cochlopetalum*, *Coryopedilum*, and *Pardalopetalum* (Cribb, 1997; Tsai et al., 2020), which was supported by our phylogenetic tree (Figure 8)*.* It is worth noting that *P. hirsutissimum*, a species belonging to sect. *Paphiopedilum* by morphology, was placed outside the section as a paraphyly to *Paphiopedilum* plus *Barbata* sections (Figure 8), which was different from two previous reports (Guo et al., 2016; Tsai et al., 2020). Guo et al. (2016) constructed a neighbor-joining (NJ) tree based on the combination of eight cp DNAs (*rbcL*, *accD*, *matK*, *ycf1*, *rpoC2*, *trnS-trnfM*, *atpI-atpH*, and *atpF-atpH*) from 77 *Paphiopedilum* species, in which two *P. hirsutissimum* samples were both clustered together with the species of sect. *Barbata* in multiple phylogenetic trees (NJ, MP, and ML) constructed by Tsai et al. (2020) with combined data of nuclear ribosomal ITS, plastid *trnL* intron, *trnL*-*trnF* spacer, and *atpB*-*rbcL* spacer from 78 *Paphiopedilum* taxa; however, *P. hirsutissimum* was grouped together with the species from sect. *Paphiopedilum*, although with medium bootstrap support values (∼50%). Recently, a more comprehensive ML tree was constructed with complete cp genome data of *Paphiopedilum*, which placed two *P. hirsutissimum* samples together with *P. rungiyasanum* and formed a parallel relationship with sect. *Paphiopedilum* and sect. *Barbata* (Guo et al., 2021a), similar to the results in our study. To further confirm the phylogenetic position of *P. hirsutissimum*, we constructed four different infrageneric phylogenetic trees with the whole cp genome sequences, coding regions, non-coding regions, and ten hypervariable regions of nine *Paphiopedilum* taxa and one outgroup species (*Cypripedium macranthos*), respectively. The results showed that *P. hirsutissimum* had a closer relationship to *P. violascens* (sect. *Barbata*) than to *P. tranlienianum* (sect. *Paphiopedilum*) based on the whole cp genome data (Figure 9A) but was closer to *P. tranlienianum* when using the sequences of coding regions or the 10 hypervariable regions (Figures 9B,D). In the phylogenetic tree built from the non-coding sequences, *P. hirsutissimum* was positioned at the place parallel to *P. tranlienianum* and *P. violascens* (Figure 9C), which was identical to the result with 66 orchid cp genomes (Figure 8). So, further studies are necessary to determine the phylogenetic position of *P. hirsutissimum.*

In addition, we found that *P. wardii* was clustered in the subgenus *Parvisepalum*, according to recent molecular documents; however, this species was grouped in sect. *Barbata* of the subgenus *Paphiopedilum* (Tsai et al., 2020; Guo et al., 2021a). Braem and Chiron (2003) classified *P. wardii* into sect. *Planipetalum* of subgenus *Sigmatopetalum*, which also included sect. *Sigmatopetalum, Spathopetalum, Blepharopetalum, Punctuatum,* and *Barbata*, suggesting *P. wardii* is closer to the species in the sect. *Barbata.* Based on these documents, we suspected that the sequence of *P. wardii* submitted in NCBI might be wrongly obtained from other species in the subgenus *Parvisepalum.*

## Data Availability

The original contributions presented in the study are publicly available in NCBI under accession numbers MW794129-MW794134.
