# Supplementary material for: Comparative Analysis of the Complete Chloroplast Genomes of Nine Paphiopedilum Species
Source: Front Genet. 2022 Feb 4;12:772415. doi: 10.3389/fgene.2021.772415 (PMC8854857; doi:10.3389/fgene.2021.772415)
Supplement: Supplementary file 1 [file DataSheet1.docx]

Supplementary Material

## Supplementary Figures


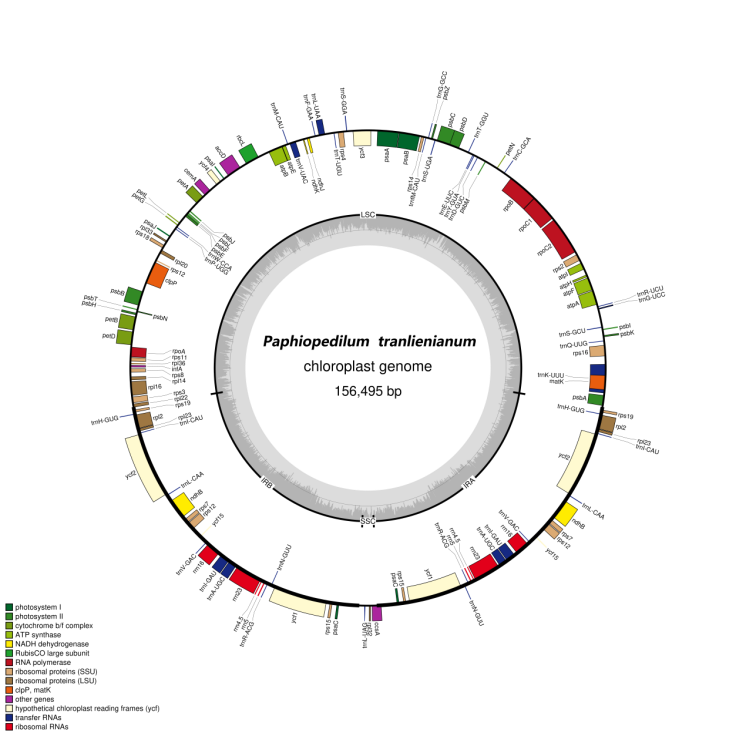

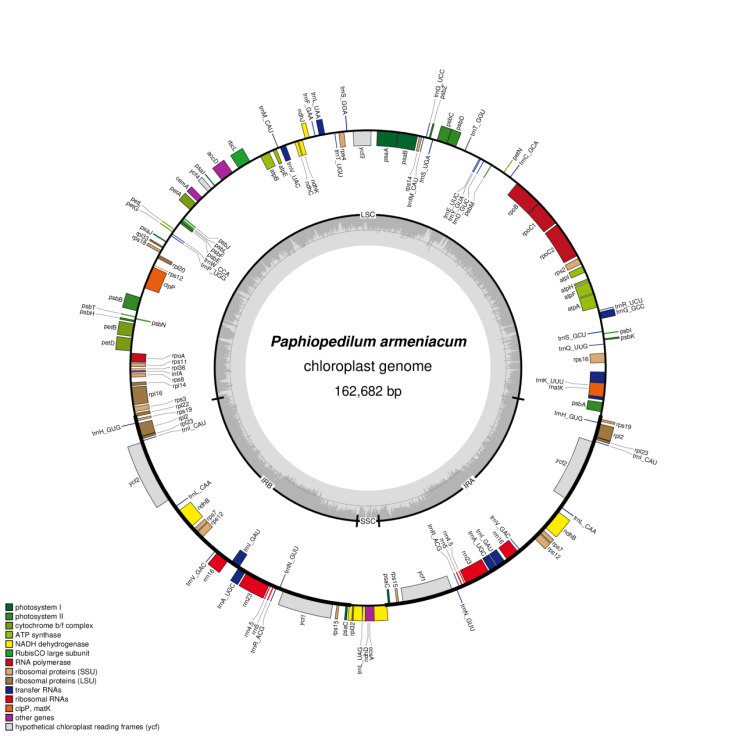


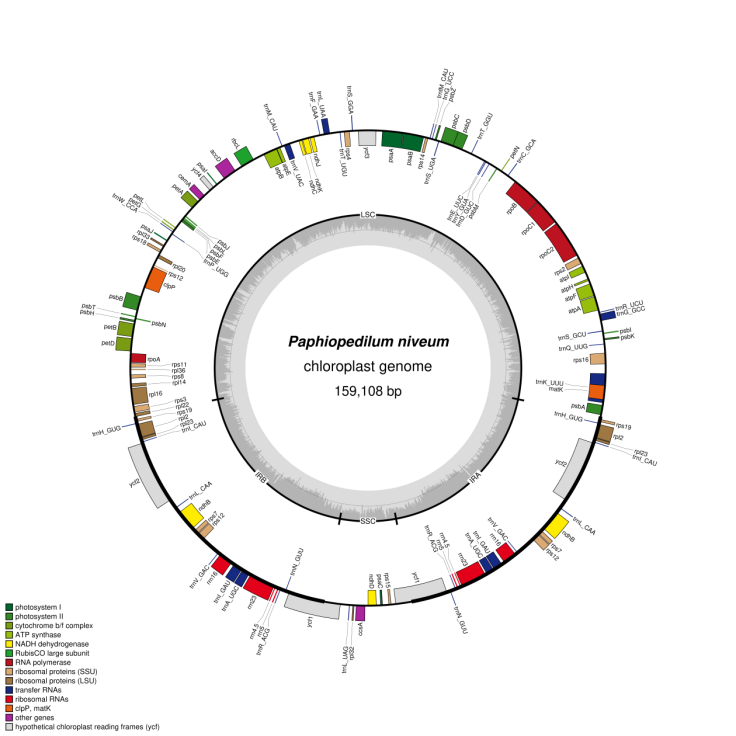

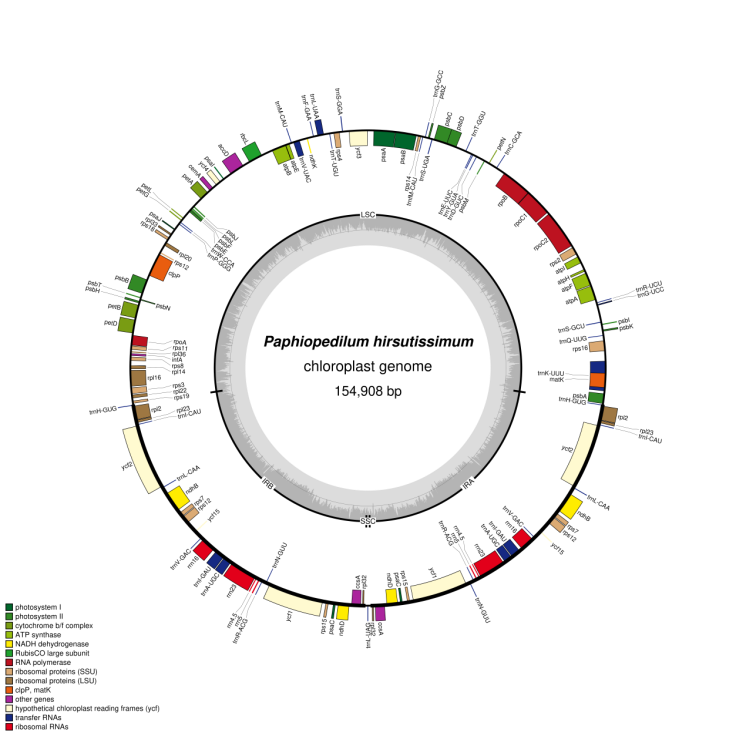


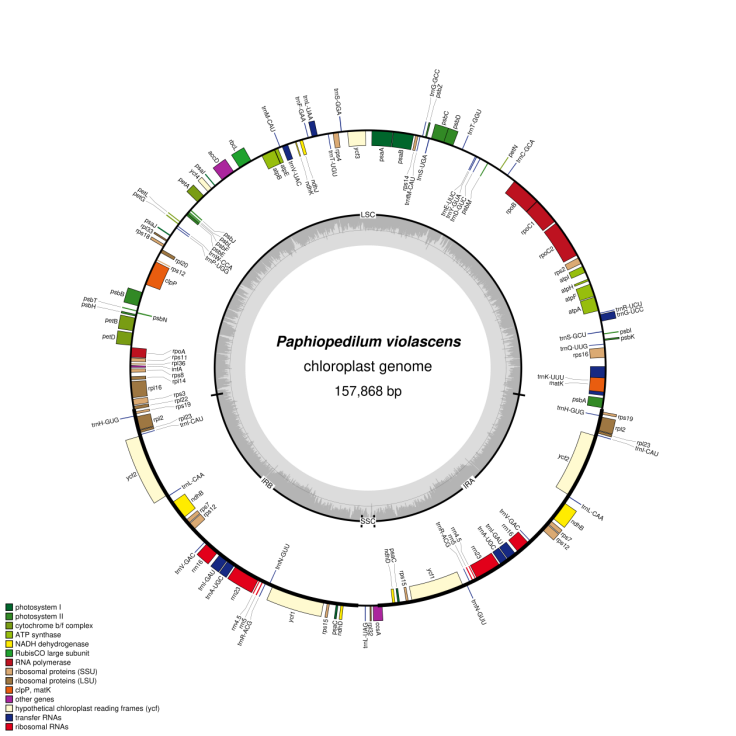

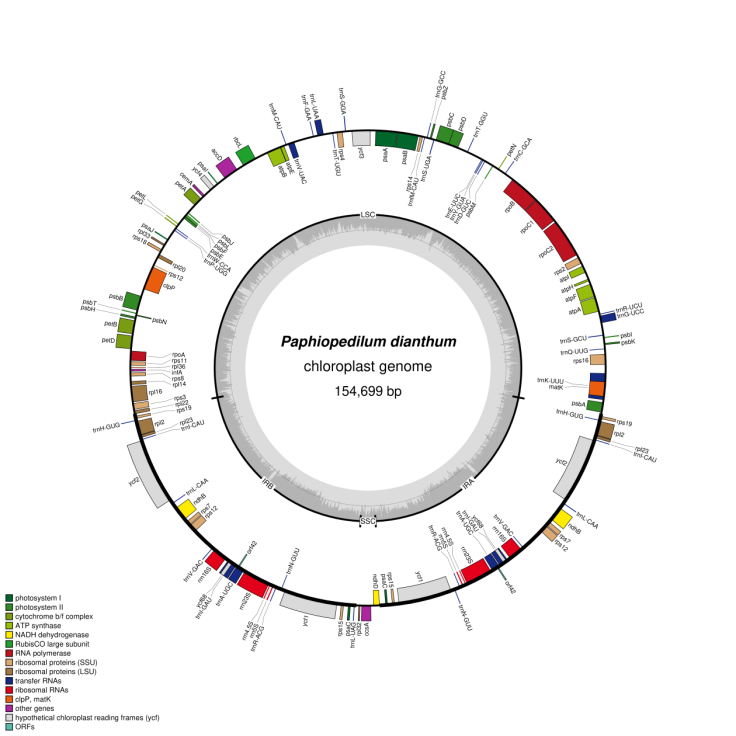


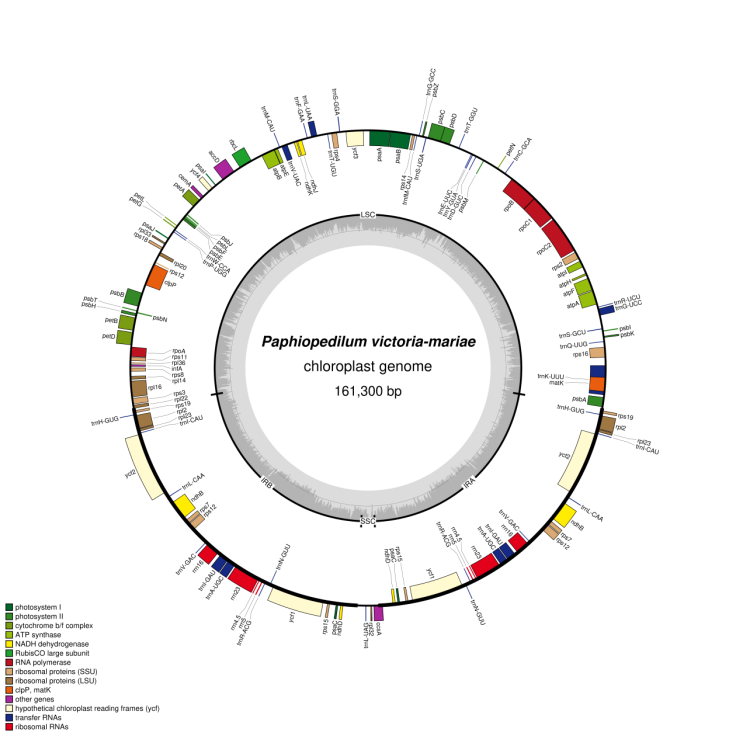

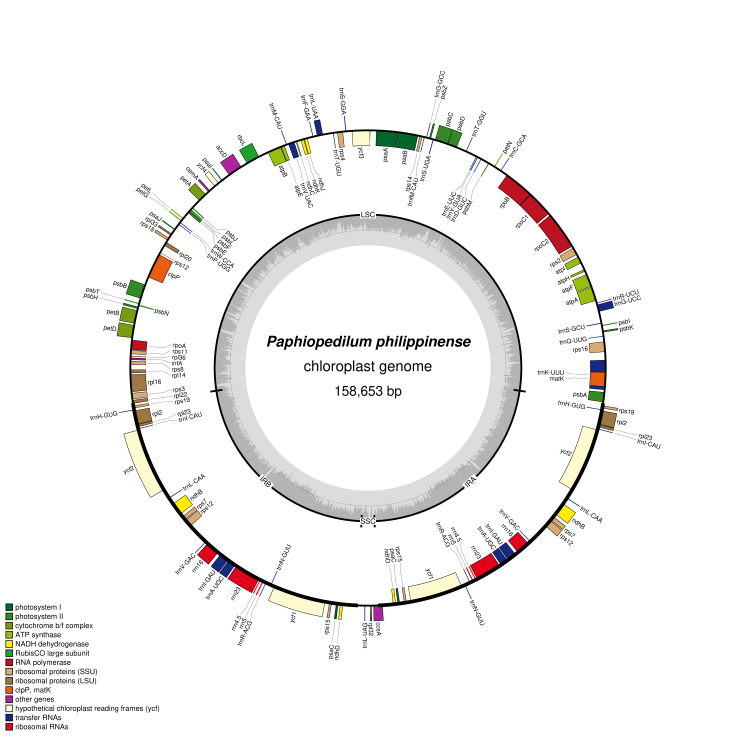


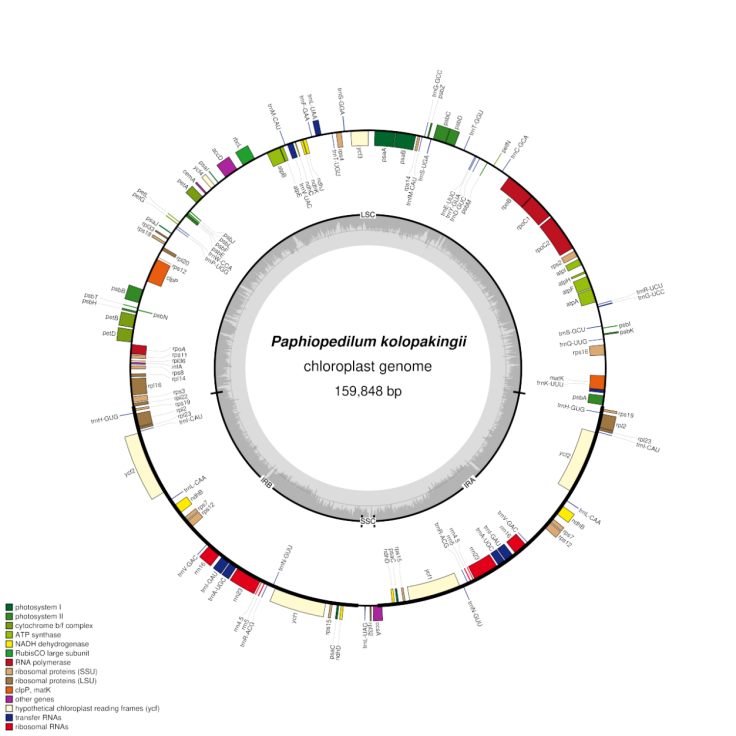


**Figure S1.** Structure map of the nine *Paphiopedilum* chloroplast genomes.

## Supplementary Tables

**Table S1**. List of the cp genomes used in phylogenetic analysis.

| **No.** | **Subfamily** | **Tribe** | **Species** | **Accession number** |
| --- | --- | --- | --- | --- |
|  | **Apostasioideae** |  | *Apostasia odorata* | KM244734.1 |
|  |  |  | *Apostasia wallichii* | LC199394.1 |
|  |  |  | *Neuwiedia singapureana* | KM244735.1 |
|  | **Vanilloideae** | Vanilleae | *Vanilla aphylla* | LC085348.1 |
|  |  |  | *Vanilla pompona* | MF197310.1 |
|  | **Cypripedioideae** |  | *Cypripedium calceolus* | MN602053.1 |
|  |  |  | *Cypripedium formosanum* | KJ501998.1 |
|  |  |  | *Cypripedium japonicum* | KJ625630.1 |
|  |  |  | *Cypripedium subtropicum* | MT937100.1 |
|  |  |  | *Cypripedium tibeticum* | MN561380.1 |
|  |  |  | *Cypripedium tibeticum* | MT937101.1 |
|  |  |  | *Paphiopedilum armeniacum* | KJ566307.1 |
|  |  |  | *Paphiopedilum armeniacum* | KT388109.1 |
|  |  |  | *Paphiopedilum armeniacum* | LC085347.1 |
|  |  |  | *Paphiopedilum barbigerum* | MN153814.1 |
|  |  |  | *Paphiopedilum concolor* | MH191340.1 |
|  |  |  | *Paphiopedilum delenatii* | MK463585.1 |
|  |  |  | *Paphiopedilum dianthum* | MF983795.1 |
|  |  |  | *Paphiopedilum emersonii* | MT648789.1 |
|  |  |  | *Paphiopedilum gratrixianum* | MW284890.1 |
|  |  |  | *Paphiopedilum hirsutissimum* | MN153815.1 |
|  |  |  | *Paphiopedilum hirsutissimum* | MW794130* |
|  |  |  | *Paphiopedilum kolopakingii* | MW794132* |
|  |  |  | *Paphiopedilum malipoense* | MN016934.1 |
|  |  |  | *Paphiopedilum micranthum* | MN535014.1 |
|  |  |  | *Paphiopedilum niveum* | KJ524105.1 |
|  |  |  | *Paphiopedilum parishii* | MW528213.1 |
|  |  |  | *Paphiopedilum philippinense* | MW794131* |
|  |  |  | *Paphiopedilum purpuratum* | MN535015.1 |
|  |  |  | *Paphiopedilum spicerianum* | MT683624.1 |
|  |  |  | *Paphiopedilum tranlienianum* | MH150886.1 |
|  |  |  | *Paphiopedilum tranlienianum* | MW794129* |
|  |  |  | *Paphiopedilum victoria-mariae* | MW794133* |
|  |  |  | *Paphiopedilum violascens* | MW794134* |
|  |  |  | *Paphiopedilum wardii* | MH191341.1 |
|  |  |  | *Phragmipedium longifolium* | KM032625.1 |
|  | **Orchidoideae** | Orchideae | *Galearis cyclochila* | MN200388.1 |
|  |  |  | *Gymnadenia conopsea* | MN200391.1 |
|  |  |  | *Habenaria radiata* | KX871237.1 |
|  |  |  | *Platanthera japonica* | MN631092.1 |
|  |  |  | *Ponerorchis gracilis* | MN200376.1 |
|  |  | Cranichideae | *Anoectochilus emeiensis* | LC057212.1 |
|  |  |  | *Aspidogyne longicornu* | MN597437.1 |
|  |  |  | *Eurystyles cotyledon* | MN597435.1 |
|  |  |  | *Goodyera procera* | KT886429.1 |
|  |  |  | *Ludisia discolor* | KU578274.1 |
|  |  |  | *Prescottia stachyodes* | MN597433.1 |
|  |  |  | *Sauroglossum elatum* | MN597432.1 |
|  |  |  | *Spiranthes sinensis* | MK936427.1 |
|  |  | Diurideae | *Rhizanthella gardneri* | GQ413967.1 |
|  | **Epidendroideae** | Neottieae | *Palmorchis pabstii* | MH590357.1 |
|  |  |  | *Epipactis mairei* | MG925367.1 |
|  |  | Sobralieae | *Sobralia callosal* | KM032623.1 |
|  |  | Gastrodieae | *Gastrodia elata* | MF163256.1 |
|  |  | Arethuseae | *Bletilla striata* | KT588924.1 |
|  |  | Malaxeae | *Bulbophyllum mentosum* | MN604056.1 |
|  |  |  | *Dendrobium nobile* | KT591465.1 |
|  |  |  | *Liparis japonica* | MK886513.1 |
|  |  | Cymbidieae | *Cymbidium faberi* | KR919606.1 |
|  |  |  | *Oncidium Gower Ramsey* | GQ324949.1 |
|  |  | Vandeae | *Phalaenopsis equestris* | JF719062.1 |
|  |  |  | *Vanda concolor* | MK836105.1 |
|  |  | Podochileae | *Eria corneri* | MN477202.1 |
|  |  | Collabieae | *Calanthe delavayi* | MK388860.1 |
|  |  | Epidendreae | *Cattleya crispate* | KP168671.1 |
|  |  |  | *Cremastra appendiculata* | MH356724.1 |
|  | **Liliaceae** |  | *Lilium regale* | MK493302.1 |
|  |  |  | *Tulipa altaica* | MK673755.1 |

* The cp genomes sequenced in this study.

**Table S2.** List of genes in the nine *Paphiopedilum* chloroplast genomes.

| Category of genes | Function of genes | Name of genes | | | | | |
| --- | --- | --- | --- | --- | --- | --- | --- |
| Ribosomal RNA genes | Self-replication | *rrn16#* | *rrn23#* | *rrn4.5#* | *rrn5#* |  |  |
| Transfer RNA genes | Self-replication | *trnA-UGC*#* | *trnC-GCA* | *trnD-GUC* | *trnE-UUC* | *trnF-GAA* | *trnG-GCC* |
|  |  | *trnG-UCC* | *trnH-GUG#* | *trnI-CAU#* | *trnI-GAU*#* | *trnK-UUU** | *trnL-CAA#* |
|  |  | *trnL-UAA** | *trnL-UAG* | *trnM-CAU* | *trnN-GUU#* | *trnP-GGG*(4) | *trnP-UGG* |
|  |  | *trnQ-UUG* | *trnR-ACG#* | *trnR-UCU* | *trnS-GCU* | *trnS-GGA* | *trnS-UGA* |
|  |  | *trnT-GGU* | *trnT-UGU* | *trnV-GAC#* | *trnV-UAC** | *trnW-CCA* | *trnY-GUA* |
|  |  | *trnfM* |  |  |  |  |  |
| Subunits of Photosystem I | Genes for photosynthesis | *psaA* | *psaB* | *psaC#* | *psaI* | *psaJ* | *ycf3*** |
|  |  | *ycf4* |  |  |  |  |  |
| Subunits of Photosystem II | Genes for photosynthesis | *psbA* | *psbB* | *psbC* | *psbD* | *psbE* | *psbF* |
|  |  | *psbH* | *psbI* | *psbJ* | *psbK* | *psbL* | *psbM* |
|  |  | *psbN* | *psbT* | *psbZ* |  |  |  |
| Subunits of NADH dehydrogenase | Genes for photosynthesis | *ndhB*#ψ | *ndhD*[1]#ψ | *ndhJ*[2,4,6]ψ | *ndhK*[2,6]ψ | *ndhC*(3,8,9)ψ |  |
| Subunits of cytochrome | Genes for photosynthesis | *petA* | *petB** | *petD** | *petG* | *petL* | *petN* |
| Subunits of ATP synthase | Genes for photosynthesis | *atpA* | *atpB* | *atpE* | *atpF** | *atpH* | *atpI* |
| Large subunit of Rubisco | Genes for photosynthesis | *rbcL* |  |  |  |  |  |
| Large subunit of ribosome (LSU) | Self-replication | *rpl14* | *rpl16** | *rpl2*#* | *rpl20* | *rpl22* | *rpl23#* |
|  |  | *rpl32#* | *rpl33* | *rpl36* |  |  |  |
| Small subunit of ribosome (SSU) | Self-replication | *rps11* | *rps12*#* | *rps14* | *rps15#* | *rps16** | *rps18* |
|  |  | *rps19#* | *rps2* | *rps3* | *rps4* | *rps7*# | *rps8* |
| DNA-dependent RNA polymerase | Self-replication | *rpoA* | *rpoB* | *rpoC1** | *rpoC2* |  |  |
| Other genes | acetyl-CoA carboxylase carboxyltransferase beta subunit | *accD* |  |  |  |  |  |
|  | cytochrome c heme attachment protein | *ccsA#* |  |  |  |  |  |
|  | Chloroplast envelope membrane protein | *cemA*[5]ψ |  |  |  |  |  |
|  | ATP-dependent protease subunit P | *clpP*** |  |  |  |  |  |
|  | Translation initiation factor | *infA*[3] |  |  |  |  |  |
|  | maturase K | *matK* |  |  |  |  |  |
|  | hypothetical protein RF1 | *ycf1#* |  |  |  |  |  |
|  | hypothetical protein RF2 | *ycf2#* |  |  |  |  |  |
|  | Ycf15 | *ycf15*(1,4)# |  |  |  |  |  |
|  |  | *ycf68*(6)#ψ | *orf42*(6)#ψ |  |  |  |  |

*—Gene containing a single intron; **—Gene containing two introns; #—One gene copy in each IR; Ψ—Pseudogene; ()—Gene exists in some species; []—Gene do not exist in some species

Species: 1, *P. tranlienianum*; 2, *P. armeniacum*; 3, *P. niveum*; 4, *P. hirsutissimum*; 5, *P. violascens*; 6, *P. dianthum*; 7, *P. victoria-mariae*; 8, *P. kolopakingii*; 9, *P. philippinense*

**Table S3.** SSRs quantity distributed in the nine *Paphiopedilum* chloroplast genomes.

| **Species** | **Category** | **Type** | **Number** | **Intergenic** | **Gene** | **Intron** | **LSC** | **SSC** | **IRa** | **IRb** |
| --- | --- | --- | --- | --- | --- | --- | --- | --- | --- | --- |
| ***P. tranlienianum*** | Mononucleotide | (A)8 | 23 | 9 | 12 | 2 | 12 | 0 | 4 | 7 |
|  |  | (A)9 | 16 | 6 | 8 | 2 | 7 | 1 | 4 | 4 |
|  |  | (A)10 | 11 | 5 | 6 | 0 | 6 | 0 | 2 | 3 |
|  |  | (A)11 | 4 | 2 | 2 | 0 | 2 | 0 | 0 | 2 |
|  |  | (A)12 | 7 | 7 | 0 | 0 | 7 | 0 | 0 | 0 |
|  |  | (A)13 | 2 | 2 | 0 | 0 | 2 | 0 | 0 | 0 |
|  |  | (A)14 | 2 | 1 | 0 | 1 | 2 | 0 | 0 | 0 |
|  |  | (A)16 | 1 | 1 | 0 | 0 | 0 | 0 | 1 | 0 |
|  |  | (A)17 | 1 | 0 | 0 | 1 | 1 | 0 | 0 | 0 |
|  |  | (A)22 | 1 | 1 | 0 | 0 | 1 | 0 | 0 | 0 |
|  |  | (C)9 | 2 | 1 | 0 | 1 | 2 | 0 | 0 | 0 |
|  |  | (C)10 | 1 | 0 | 0 | 1 | 0 | 0 | 1 | 0 |
|  |  | (C)13 | 1 | 1 | 0 | 0 | 1 | 0 | 0 | 0 |
|  |  | (G)9 | 1 | 1 | 0 | 0 | 1 | 0 | 0 | 0 |
|  |  | (G)10 | 1 | 0 | 0 | 1 | 0 | 0 | 0 | 1 |
|  |  | (G)11 | 1 | 1 | 0 | 0 | 1 | 0 | 0 | 0 |
|  |  | (T)8 | 41 | 18 | 14 | 9 | 30 | 0 | 7 | 4 |
|  |  | (T)9 | 23 | 9 | 9 | 5 | 14 | 1 | 4 | 4 |
|  |  | (T)10 | 13 | 5 | 5 | 3 | 8 | 0 | 3 | 2 |
|  |  | (T)11 | 8 | 4 | 2 | 2 | 6 | 0 | 2 | 0 |
|  |  | (T)12 | 4 | 4 | 0 | 0 | 4 | 0 | 0 | 0 |
|  |  | (T)14 | 1 | 0 | 0 | 1 | 1 | 0 | 0 | 0 |
|  |  | (T)15 | 1 | 1 | 0 | 0 | 1 | 0 | 0 | 0 |
|  |  | (T)16 | 4 | 2 | 0 | 2 | 3 | 0 | 0 | 1 |
|  |  | (T)17 | 1 | 1 | 0 | 0 | 1 | 0 | 0 | 0 |
|  |  | (T)18 | 1 | 1 | 0 | 0 | 1 | 0 | 0 | 0 |
|  |  | Subtotal | 172 | 83 | 58 | 31 | 114 | 2 | 28 | 28 |
|  | Dinucleotide | (AC)4 | 1 | 1 | 0 | 0 | 1 | 0 | 0 | 0 |
|  |  | (AG)4 | 4 | 1 | 2 | 1 | 1 | 0 | 2 | 1 |
|  |  | (AT)4 | 15 | 11 | 3 | 1 | 15 | 0 | 0 | 0 |
|  |  | (AT)5 | 3 | 2 | 1 | 0 | 3 | 0 | 0 | 0 |
|  |  | (AT)7 | 2 | 2 | 0 | 0 | 0 | 0 | 1 | 1 |
|  |  | (AT)9 | 1 | 1 | 0 | 0 | 1 | 0 | 0 | 0 |
|  |  | (AT)10 | 1 | 1 | 0 | 0 | 1 | 0 | 0 | 0 |
|  |  | (CT)4 | 4 | 1 | 3 | 0 | 1 | 0 | 1 | 2 |
|  |  | (GA)4 | 4 | 1 | 3 | 0 | 1 | 0 | 1 | 2 |
|  |  | (GA)5 | 1 | 0 | 1 | 0 | 0 | 0 | 0 | 1 |
|  |  | (TA)4 | 30 | 27 | 2 | 1 | 26 | 2 | 1 | 1 |
|  |  | (TA)5 | 3 | 3 | 0 | 0 | 3 | 0 | 0 | 0 |
|  |  | (TA)6 | 5 | 4 | 0 | 1 | 5 | 0 | 0 | 0 |
|  |  | (TA)7 | 3 | 2 | 0 | 1 | 3 | 0 | 0 | 0 |
|  |  | (TC)4 | 4 | 1 | 3 | 0 | 1 | 0 | 2 | 1 |
|  |  | (TC)5 | 1 | 0 | 1 | 0 | 0 | 0 | 1 | 0 |
|  |  | (TG)4 | 1 | 0 | 1 | 0 | 1 | 0 | 0 | 0 |
|  |  | Subtotal | 83 | 58 | 20 | 5 | 63 | 2 | 9 | 9 |
|  | Trinucleotide | (AAT)4 | 1 | 1 | 0 | 0 | 0 | 1 | 0 | 0 |
|  |  | (AGA)4 | 1 | 0 | 1 | 0 | 0 | 0 | 0 | 1 |
|  |  | (ATA)4 | 4 | 4 | 0 | 0 | 3 | 0 | 0 | 1 |
|  |  | (ATA)5 | 1 | 1 | 0 | 0 | 1 | 0 | 0 | 0 |
|  |  | (ATT)4 | 4 | 3 | 0 | 1 | 3 | 0 | 1 | 0 |
|  |  | (TAA)4 | 1 | 1 | 0 | 0 | 1 | 0 | 0 | 0 |
|  |  | (TAT)4 | 3 | 3 | 0 | 0 | 3 | 0 | 0 | 0 |
|  |  | (TCT)4 | 2 | 1 | 1 | 0 | 1 | 0 | 1 | 0 |
|  |  | (TTA)4 | 2 | 1 | 0 | 1 | 2 | 0 | 0 | 0 |
|  |  | Subtotal | 19 | 15 | 2 | 2 | 14 | 1 | 2 | 2 |
|  | Tetranucleotide | (AAAT)3 | 1 | 0 | 1 | 0 | 0 | 0 | 0 | 1 |
|  |  | (AATG)3 | 1 | 0 | 1 | 0 | 1 | 0 | 0 | 0 |
|  |  | (ACAT)3 | 1 | 0 | 1 | 0 | 1 | 0 | 0 | 0 |
|  |  | (ATAG)3 | 1 | 1 | 0 | 0 | 0 | 0 | 1 | 0 |
|  |  | (ATTC)3 | 1 | 0 | 1 | 0 | 0 | 0 | 0 | 1 |
|  |  | (ATTT)3 | 1 | 0 | 0 | 1 | 1 | 0 | 0 | 0 |
|  |  | (CTTT)3 | 1 | 1 | 0 | 0 | 1 | 0 | 0 | 0 |
|  |  | (GAAT)3 | 1 | 0 | 1 | 0 | 0 | 0 | 1 | 0 |
|  |  | (GATA)4 | 1 | 1 | 0 | 0 | 1 | 0 | 0 | 0 |
|  |  | (GTCT)3 | 1 | 0 | 1 | 0 | 1 | 0 | 0 | 0 |
|  |  | (TAAA)3 | 2 | 2 | 0 | 0 | 2 | 0 | 0 | 0 |
|  |  | (TAAT)4 | 1 | 1 | 0 | 0 | 1 | 0 | 0 | 0 |
|  |  | (TATT)3 | 1 | 0 | 1 | 0 | 0 | 0 | 1 | 0 |
|  |  | (TCTA)3 | 1 | 1 | 0 | 0 | 0 | 0 | 0 | 1 |
|  |  | (TTAT)3 | 1 | 1 | 0 | 0 | 1 | 0 | 0 | 0 |
|  |  | (TTTA)3 | 1 | 1 | 0 | 0 | 0 | 1 | 0 | 0 |
|  |  | Subtotal | 17 | 9 | 7 | 1 | 10 | 1 | 3 | 3 |
|  | Pentanucleotide | (ATAAT)3 | 1 | 1 | 0 | 0 | 1 | 0 | 0 | 0 |
|  |  | (ATATA)3 | 1 | 1 | 0 | 0 | 1 | 0 | 0 | 0 |
|  |  | (TATTG)3 | 1 | 1 | 0 | 0 | 1 | 0 | 0 | 0 |
|  |  | (TTATA)3 | 2 | 1 | 0 | 1 | 2 | 0 | 0 | 0 |
|  |  | Subtotal | 5 | 4 | 0 | 1 | 5 | 0 | 0 | 0 |
|  | Hexanucleotide | (AAGAGG)4 | 1 | 0 | 1 | 0 | 0 | 0 | 0 | 1 |
|  |  | (CCTCTT)4 | 1 | 0 | 1 | 0 | 0 | 0 | 1 | 0 |
|  |  | (CTATAG)3 | 1 | 0 | 0 | 1 | 1 | 0 | 0 | 0 |
|  |  | (CTCTTC)4 | 1 | 0 | 1 | 0 | 0 | 0 | 1 | 0 |
|  |  | (CTTCTA)3 | 1 | 0 | 1 | 0 | 0 | 0 | 1 | 0 |
|  |  | (GGAAGA)4 | 1 | 0 | 1 | 0 | 0 | 0 | 0 | 1 |
|  |  | (TAGAAG)3 | 1 | 0 | 1 | 0 | 0 | 0 | 0 | 1 |
|  |  | Subtotal | 7 | 0 | 6 | 1 | 1 | 0 | 3 | 3 |
|  | Subtotal |  | 303 | 169 | 93 | 41 | 207 | 6 | 45 | 45 |
| ***P. victoria-mariae*** | Mononucleotide | (A)8 | 28 | 11 | 13 | 4 | 18 | 0 | 3 | 7 |
|  |  | (A)9 | 19 | 9 | 7 | 3 | 8 | 1 | 6 | 4 |
|  |  | (A)10 | 9 | 6 | 3 | 0 | 6 | 0 | 1 | 2 |
|  |  | (A)11 | 1 | 0 | 1 | 0 | 0 | 0 | 0 | 1 |
|  |  | (A)12 | 2 | 2 | 0 | 0 | 2 | 0 | 0 | 0 |
|  |  | (A)13 | 4 | 3 | 1 | 0 | 3 | 0 | 0 | 1 |
|  |  | (A)15 | 1 | 0 | 0 | 1 | 1 | 0 | 0 | 0 |
|  |  | (A)16 | 1 | 0 | 1 | 0 | 1 | 0 | 0 | 0 |
|  |  | (A)17 | 1 | 0 | 0 | 1 | 1 | 0 | 0 | 0 |
|  |  | (A)19 | 1 | 1 | 0 | 0 | 1 | 0 | 0 | 0 |
|  |  | (A)20 | 1 | 1 | 0 | 0 | 0 | 0 | 1 | 0 |
|  |  | (A)26 | 1 | 1 | 0 | 0 | 1 | 0 | 0 | 0 |
|  |  | (C)8 | 2 | 1 | 0 | 1 | 2 | 0 | 0 | 0 |
|  |  | (C)10 | 1 | 0 | 0 | 1 | 0 | 0 | 1 | 0 |
|  |  | (G)8 | 2 | 2 | 0 | 0 | 2 | 0 | 0 | 0 |
|  |  | (G)10 | 1 | 0 | 0 | 1 | 0 | 0 | 0 | 1 |
|  |  | (G)11 | 1 | 1 | 0 | 0 | 1 | 0 | 0 | 0 |
|  |  | (T)8 | 36 | 15 | 15 | 6 | 26 | 0 | 7 | 3 |
|  |  | (T)9 | 24 | 10 | 8 | 6 | 14 | 0 | 4 | 6 |
|  |  | (T)10 | 14 | 5 | 4 | 5 | 10 | 1 | 2 | 1 |
|  |  | (T)11 | 8 | 4 | 2 | 2 | 7 | 0 | 1 | 0 |
|  |  | (T)12 | 6 | 4 | 0 | 2 | 6 | 0 | 0 | 0 |
|  |  | (T)13 | 1 | 0 | 1 | 0 | 0 | 0 | 1 | 0 |
|  |  | (T)14 | 2 | 0 | 0 | 2 | 2 | 0 | 0 | 0 |
|  |  | (T)16 | 1 | 1 | 0 | 0 | 1 | 0 | 0 | 0 |
|  |  | (T)18 | 1 | 1 | 0 | 0 | 0 | 1 | 0 | 0 |
|  |  | (T)20 | 1 | 1 | 0 | 0 | 0 | 0 | 0 | 1 |
|  |  | (T)23 | 1 | 1 | 0 | 0 | 1 | 0 | 0 | 0 |
|  |  | Subtotal | 171 | 80 | 56 | 35 | 114 | 3 | 27 | 27 |
|  | Dinucleotide | (AC)4 | 1 | 1 | 0 | 0 | 1 | 0 | 0 | 0 |
|  |  | (AG)4 | 4 | 1 | 2 | 1 | 1 | 0 | 2 | 1 |
|  |  | (AT)4 | 30 | 25 | 3 | 2 | 27 | 3 | 0 | 0 |
|  |  | (AT)5 | 6 | 5 | 1 | 0 | 6 | 0 | 0 | 0 |
|  |  | (AT)6 | 2 | 1 | 0 | 1 | 1 | 0 | 1 | 0 |
|  |  | (AT)7 | 2 | 2 | 0 | 0 | 0 | 0 | 1 | 1 |
|  |  | (AT)8 | 1 | 1 | 0 | 0 | 1 | 0 | 0 | 0 |
|  |  | (CT)4 | 4 | 1 | 3 | 0 | 1 | 0 | 1 | 2 |
|  |  | (GA)4 | 4 | 1 | 3 | 0 | 1 | 0 | 1 | 2 |
|  |  | (GA)5 | 1 | 0 | 1 | 0 | 0 | 0 | 0 | 1 |
|  |  | (TA)4 | 28 | 23 | 2 | 3 | 25 | 1 | 1 | 1 |
|  |  | (TA)5 | 6 | 6 | 0 | 0 | 6 | 0 | 0 | 0 |
|  |  | (TA)6 | 8 | 6 | 1 | 1 | 6 | 1 | 0 | 1 |
|  |  | (TA)7 | 2 | 1 | 0 | 1 | 2 | 0 | 0 | 0 |
|  |  | (TA)8 | 2 | 2 | 0 | 0 | 1 | 1 | 0 | 0 |
|  |  | (TA)14 | 1 | 1 | 0 | 0 | 1 | 0 | 0 | 0 |
|  |  | (TC)4 | 3 | 1 | 2 | 0 | 0 | 0 | 2 | 1 |
|  |  | (TC)5 | 1 | 0 | 1 | 0 | 0 | 0 | 1 | 0 |
|  |  | (TG)4 | 1 | 0 | 1 | 0 | 1 | 0 | 0 | 0 |
|  |  | Subtotal | 107 | 78 | 20 | 9 | 81 | 6 | 10 | 10 |
|  | Trinucleotide | (AAT)4 | 1 | 1 | 0 | 0 | 1 | 0 | 0 | 0 |
|  |  | (AGA)4 | 1 | 0 | 1 | 0 | 0 | 0 | 0 | 1 |
|  |  | (ATA)4 | 6 | 6 | 0 | 0 | 5 | 1 | 0 | 0 |
|  |  | (ATA)5 | 1 | 0 | 0 | 1 | 1 | 0 | 0 | 0 |
|  |  | (ATT)4 | 2 | 1 | 0 | 1 | 2 | 0 | 0 | 0 |
|  |  | (TAA)4 | 1 | 1 | 0 | 0 | 1 | 0 | 0 | 0 |
|  |  | (TAT)4 | 4 | 4 | 0 | 0 | 4 | 0 | 0 | 0 |
|  |  | (TAT)5 | 1 | 1 | 0 | 0 | 1 | 0 | 0 | 0 |
|  |  | (TAT)6 | 1 | 1 | 0 | 0 | 1 | 0 | 0 | 0 |
|  |  | (TCT)4 | 2 | 1 | 1 | 0 | 1 | 0 | 1 | 0 |
|  |  | (TTA)4 | 1 | 0 | 0 | 1 | 1 | 0 | 0 | 0 |
|  |  | Subtotal | 21 | 16 | 2 | 3 | 18 | 1 | 1 | 1 |
|  | Tetranucleotide | (AAAT)3 | 1 | 1 | 0 | 0 | 1 | 0 | 0 | 0 |
|  |  | (AATA)4 | 1 | 1 | 0 | 0 | 0 | 0 | 1 | 0 |
|  |  | (AATG)3 | 1 | 0 | 1 | 0 | 1 | 0 | 0 | 0 |
|  |  | (ACAT)3 | 1 | 0 | 1 | 0 | 1 | 0 | 0 | 0 |
|  |  | (ATAG)3 | 1 | 1 | 0 | 0 | 0 | 0 | 1 | 0 |
|  |  | (ATTC)3 | 1 | 0 | 1 | 0 | 0 | 0 | 0 | 1 |
|  |  | (CTTT)3 | 1 | 1 | 0 | 0 | 1 | 0 | 0 | 0 |
|  |  | (GAAT)3 | 1 | 0 | 1 | 0 | 0 | 0 | 1 | 0 |
|  |  | (GATA)3 | 1 | 1 | 0 | 0 | 1 | 0 | 0 | 0 |
|  |  | (GTCT)3 | 1 | 0 | 1 | 0 | 1 | 0 | 0 | 0 |
|  |  | (TAAA)3 | 1 | 1 | 0 | 0 | 1 | 0 | 0 | 0 |
|  |  | (TCTA)3 | 1 | 1 | 0 | 0 | 0 | 0 | 0 | 1 |
|  |  | (TTAT)3 | 2 | 1 | 0 | 1 | 2 | 0 | 0 | 0 |
|  |  | (TTAT)4 | 2 | 2 | 0 | 0 | 0 | 1 | 0 | 1 |
|  |  | (TTTA)3 | 1 | 1 | 0 | 0 | 0 | 1 | 0 | 0 |
|  |  | Subtotal | 17 | 11 | 5 | 1 | 9 | 2 | 3 | 3 |
|  | Pentanucleotide | (ATTAT)3 | 1 | 1 | 0 | 0 | 1 | 0 | 0 | 0 |
|  |  | (TATAA)3 | 1 | 1 | 0 | 0 | 1 | 0 | 0 | 0 |
|  |  | (TATAT)3 | 1 | 1 | 0 | 0 | 1 | 0 | 0 | 0 |
|  |  | (TATAT)5 | 1 | 1 | 0 | 0 | 0 | 1 | 0 | 0 |
|  |  | (TTATA)3 | 1 | 1 | 0 | 0 | 1 | 0 | 0 | 0 |
|  |  | Subtotal | 5 | 5 | 0 | 0 | 4 | 1 | 0 | 0 |
|  | Hexanucleotide | (CCTCTT)6 | 1 | 0 | 1 | 0 | 0 | 0 | 1 | 0 |
|  |  | (CTTCTA)3 | 1 | 0 | 1 | 0 | 0 | 0 | 1 | 0 |
|  |  | (GGAAGA)6 | 1 | 0 | 1 | 0 | 0 | 0 | 0 | 1 |
|  |  | (TAGAAG)3 | 1 | 0 | 1 | 0 | 0 | 0 | 0 | 1 |
|  |  | Subtotal | 4 | 0 | 4 | 0 | 0 | 0 | 2 | 2 |
|  | Subtotal |  | 325 | 190 | 87 | 48 | 226 | 13 | 43 | 43 |
| ***P. armeniacum*** | Mononucleotide | (A)8 | 33 | 16 | 14 | 3 | 21 | 2 | 4 | 6 |
|  |  | (A)9 | 23 | 8 | 11 | 4 | 10 | 1 | 5 | 7 |
|  |  | (A)10 | 13 | 11 | 2 | 0 | 10 | 0 | 1 | 2 |
|  |  | (A)11 | 6 | 2 | 1 | 3 | 5 | 0 | 0 | 1 |
|  |  | (A)12 | 3 | 2 | 1 | 0 | 2 | 0 | 1 | 0 |
|  |  | (A)13 | 2 | 1 | 1 | 0 | 1 | 0 | 0 | 1 |
|  |  | (A)16 | 1 | 0 | 1 | 0 | 0 | 0 | 0 | 1 |
|  |  | (A)18 | 1 | 1 | 0 | 0 | 0 | 1 | 0 | 0 |
|  |  | (A)21 | 1 | 0 | 0 | 1 | 1 | 0 | 0 | 0 |
|  |  | (C)8 | 2 | 1 | 1 | 0 | 1 | 0 | 0 | 1 |
|  |  | (C)9 | 2 | 1 | 0 | 1 | 1 | 0 | 1 | 0 |
|  |  | (C)12 | 1 | 0 | 0 | 1 | 1 | 0 | 0 | 0 |
|  |  | (G)8 | 1 | 0 | 1 | 0 | 0 | 0 | 1 | 0 |
|  |  | (G)9 | 2 | 1 | 0 | 1 | 1 | 0 | 0 | 1 |
|  |  | (G)11 | 1 | 1 | 0 | 0 | 1 | 0 | 0 | 0 |
|  |  | (T)8 | 37 | 21 | 13 | 3 | 27 | 0 | 6 | 4 |
|  |  | (T)9 | 23 | 10 | 11 | 2 | 11 | 0 | 7 | 5 |
|  |  | (T)10 | 10 | 5 | 3 | 2 | 6 | 1 | 2 | 1 |
|  |  | (T)11 | 10 | 8 | 1 | 1 | 7 | 2 | 1 | 0 |
|  |  | (T)12 | 6 | 4 | 1 | 1 | 4 | 1 | 0 | 1 |
|  |  | (T)13 | 1 | 0 | 1 | 0 | 0 | 0 | 1 | 0 |
|  |  | (T)14 | 1 | 0 | 0 | 1 | 1 | 0 | 0 | 0 |
|  |  | (T)15 | 1 | 0 | 0 | 1 | 1 | 0 | 0 | 0 |
|  |  | (T)16 | 3 | 2 | 1 | 0 | 2 | 0 | 1 | 0 |
|  |  | (T)17 | 1 | 0 | 0 | 1 | 1 | 0 | 0 | 0 |
|  |  | (T)18 | 1 | 0 | 0 | 1 | 1 | 0 | 0 | 0 |
|  |  | Subtotal | 186 | 95 | 64 | 27 | 116 | 8 | 31 | 31 |
|  | Dinucleotide | (AC)4 | 1 | 1 | 0 | 0 | 1 | 0 | 0 | 0 |
|  |  | (AG)4 | 3 | 0 | 1 | 2 | 1 | 0 | 1 | 1 |
|  |  | (AT)4 | 13 | 10 | 2 | 1 | 13 | 0 | 0 | 0 |
|  |  | (AT)5 | 7 | 6 | 1 | 0 | 5 | 0 | 1 | 1 |
|  |  | (AT)6 | 4 | 4 | 0 | 0 | 3 | 0 | 1 | 0 |
|  |  | (AT)8 | 1 | 1 | 0 | 0 | 1 | 0 | 0 | 0 |
|  |  | (CT)4 | 3 | 0 | 2 | 1 | 1 | 0 | 1 | 1 |
|  |  | (GA)4 | 3 | 0 | 3 | 0 | 1 | 0 | 0 | 2 |
|  |  | (GA)5 | 1 | 0 | 1 | 0 | 0 | 0 | 0 | 1 |
|  |  | (GT)4 | 1 | 1 | 0 | 0 | 1 | 0 | 0 | 0 |
|  |  | (TA)4 | 21 | 18 | 2 | 1 | 19 | 0 | 1 | 1 |
|  |  | (TA)5 | 10 | 5 | 0 | 5 | 10 | 0 | 0 | 0 |
|  |  | (TA)6 | 7 | 6 | 0 | 1 | 6 | 0 | 0 | 1 |
|  |  | (TA)7 | 3 | 3 | 0 | 0 | 3 | 0 | 0 | 0 |
|  |  | (TA)8 | 4 | 3 | 0 | 1 | 4 | 0 | 0 | 0 |
|  |  | (TA)10 | 3 | 3 | 0 | 0 | 3 | 0 | 0 | 0 |
|  |  | (TA)12 | 1 | 1 | 0 | 0 | 1 | 0 | 0 | 0 |
|  |  | (TA)15 | 1 | 1 | 0 | 0 | 0 | 1 | 0 | 0 |
|  |  | (TA)19 | 1 | 0 | 0 | 1 | 1 | 0 | 0 | 0 |
|  |  | (TC)4 | 3 | 0 | 3 | 0 | 1 | 0 | 2 | 0 |
|  |  | (TC)5 | 1 | 0 | 1 | 0 | 0 | 0 | 1 | 0 |
|  |  | (TG)4 | 1 | 0 | 1 | 0 | 1 | 0 | 0 | 0 |
|  |  | Subtotal | 93 | 63 | 17 | 13 | 76 | 1 | 8 | 8 |
|  | Trinucleotide | (AAT)4 | 2 | 2 | 0 | 0 | 2 | 0 | 0 | 0 |
|  |  | (AGA)4 | 1 | 0 | 1 | 0 | 0 | 0 | 0 | 1 |
|  |  | (ATA)4 | 1 | 1 | 0 | 0 | 1 | 0 | 0 | 0 |
|  |  | (ATT)4 | 2 | 1 | 0 | 1 | 2 | 0 | 0 | 0 |
|  |  | (TAA)5 | 1 | 1 | 0 | 0 | 1 | 0 | 0 | 0 |
|  |  | (TAT)4 | 4 | 3 | 0 | 1 | 3 | 1 | 0 | 0 |
|  |  | (TCT)4 | 2 | 1 | 1 | 0 | 1 | 0 | 1 | 0 |
|  |  | (TTA)4 | 2 | 2 | 0 | 0 | 1 | 1 | 0 | 0 |
|  |  | Subtotal | 15 | 11 | 2 | 2 | 11 | 2 | 1 | 1 |
|  | Tetranucleotide | (AAAT)3 | 3 | 3 | 0 | 0 | 2 | 0 | 1 | 0 |
|  |  | (AATA)3 | 1 | 0 | 1 | 0 | 1 | 0 | 0 | 0 |
|  |  | (AATA)4 | 1 | 1 | 0 | 0 | 1 | 0 | 0 | 0 |
|  |  | (AATG)3 | 1 | 0 | 1 | 0 | 1 | 0 | 0 | 0 |
|  |  | (ACAT)3 | 1 | 0 | 1 | 0 | 1 | 0 | 0 | 0 |
|  |  | (ATAG)3 | 1 | 1 | 0 | 0 | 0 | 0 | 1 | 0 |
|  |  | (ATTC)3 | 1 | 0 | 1 | 0 | 0 | 0 | 0 | 1 |
|  |  | (CTTT)3 | 1 | 1 | 0 | 0 | 1 | 0 | 0 | 0 |
|  |  | (GAAT)3 | 1 | 0 | 1 | 0 | 0 | 0 | 1 | 0 |
|  |  | (GTCT)3 | 1 | 0 | 1 | 0 | 1 | 0 | 0 | 0 |
|  |  | (TATT)3 | 1 | 1 | 0 | 0 | 1 | 0 | 0 | 0 |
|  |  | (TATT)4 | 1 | 1 | 0 | 0 | 1 | 0 | 0 | 0 |
|  |  | (TCTA)3 | 1 | 1 | 0 | 0 | 0 | 0 | 0 | 1 |
|  |  | (TTAT)3 | 5 | 4 | 0 | 1 | 4 | 0 | 0 | 1 |
|  |  | Subtotal | 20 | 13 | 6 | 1 | 14 | 0 | 3 | 3 |
|  | Pentanucleotide | (AAAAT)3 | 1 | 1 | 0 | 0 | 0 | 0 | 0 | 1 |
|  |  | (ATTTA)3 | 1 | 0 | 0 | 1 | 1 | 0 | 0 | 0 |
|  |  | (TATAC)3 | 1 | 1 | 0 | 0 | 1 | 0 | 0 | 0 |
|  |  | (TATAT)3 | 1 | 1 | 0 | 0 | 1 | 0 | 0 | 0 |
|  |  | (TATTT)3 | 1 | 1 | 0 | 0 | 0 | 0 | 1 | 0 |
|  |  | Subtotal | 5 | 4 | 0 | 1 | 3 | 0 | 1 | 1 |
|  | Hexanucleotide | (AATAGT)3 | 1 | 1 | 0 | 0 | 0 | 0 | 1 | 0 |
|  |  | (ATATAA)3 | 1 | 1 | 0 | 0 | 1 | 0 | 0 | 0 |
|  |  | (CTATTA)3 | 1 | 1 | 0 | 0 | 0 | 0 | 0 | 1 |
|  |  | (CTTCTA)3 | 1 | 0 | 1 | 0 | 0 | 0 | 1 | 0 |
|  |  | (TAGAAG)3 | 1 | 0 | 1 | 0 | 0 | 0 | 0 | 1 |
|  |  | Subtotal | 5 | 3 | 2 | 0 | 1 | 0 | 2 | 2 |
|  | Subtotal |  | 324 | 189 | 91 | 44 | 221 | 11 | 46 | 46 |
| ***P. dianthum*** | Mononucleotide | (A)8 | 23 | 7 | 12 | 4 | 13 | 0 | 4 | 6 |
|  |  | (A)9 | 22 | 11 | 8 | 3 | 13 | 1 | 4 | 4 |
|  |  | (A)10 | 6 | 2 | 2 | 2 | 4 | 0 | 1 | 1 |
|  |  | (A)11 | 3 | 2 | 1 | 0 | 2 | 0 | 0 | 1 |
|  |  | (A)12 | 2 | 2 | 0 | 0 | 2 | 0 | 0 | 0 |
|  |  | (A)13 | 2 | 1 | 1 | 0 | 1 | 0 | 0 | 1 |
|  |  | (A)14 | 2 | 1 | 0 | 1 | 2 | 0 | 0 | 0 |
|  |  | (A)17 | 1 | 1 | 0 | 0 | 1 | 0 | 0 | 0 |
|  |  | (A)18 | 1 | 1 | 0 | 0 | 0 | 0 | 1 | 0 |
|  |  | (C)8 | 1 | 0 | 0 | 1 | 1 | 0 | 0 | 0 |
|  |  | (C)9 | 1 | 1 | 0 | 0 | 1 | 0 | 0 | 0 |
|  |  | (C)10 | 2 | 1 | 0 | 1 | 1 | 0 | 1 | 0 |
|  |  | (C)11 | 1 | 0 | 0 | 1 | 1 | 0 | 0 | 0 |
|  |  | (G)8 | 1 | 1 | 0 | 0 | 1 | 0 | 0 | 0 |
|  |  | (G)10 | 1 | 0 | 0 | 1 | 0 | 0 | 0 | 1 |
|  |  | (T)8 | 27 | 9 | 12 | 6 | 16 | 1 | 6 | 4 |
|  |  | (T)9 | 28 | 13 | 10 | 5 | 18 | 2 | 4 | 4 |
|  |  | (T)10 | 8 | 4 | 2 | 2 | 6 | 0 | 1 | 1 |
|  |  | (T)11 | 10 | 5 | 2 | 3 | 9 | 0 | 1 | 0 |
|  |  | (T)12 | 3 | 0 | 0 | 3 | 3 | 0 | 0 | 0 |
|  |  | (T)13 | 2 | 1 | 1 | 0 | 1 | 0 | 1 | 0 |
|  |  | (T)16 | 1 | 1 | 0 | 0 | 1 | 0 | 0 | 0 |
|  |  | (T)18 | 1 | 1 | 0 | 0 | 0 | 0 | 0 | 1 |
|  |  | (T)19 | 1 | 1 | 0 | 0 | 1 | 0 | 0 | 0 |
|  |  | (T)22 | 2 | 1 | 0 | 1 | 2 | 0 | 0 | 0 |
|  |  | Subtotal | 152 | 67 | 51 | 34 | 100 | 4 | 24 | 24 |
|  | Dinucleotide | (AC)4 | 1 | 1 | 0 | 0 | 1 | 0 | 0 | 0 |
|  |  | (AG)4 | 3 | 0 | 1 | 2 | 1 | 0 | 1 | 1 |
|  |  | (AT)4 | 21 | 17 | 3 | 1 | 20 | 0 | 1 | 0 |
|  |  | (AT)5 | 3 | 2 | 1 | 0 | 3 | 0 | 0 | 0 |
|  |  | (AT)6 | 1 | 1 | 0 | 0 | 0 | 0 | 1 | 0 |
|  |  | (AT)7 | 2 | 2 | 0 | 0 | 0 | 0 | 1 | 1 |
|  |  | (AT)9 | 2 | 2 | 0 | 0 | 1 | 0 | 1 | 0 |
|  |  | (CT)4 | 4 | 1 | 2 | 1 | 1 | 0 | 1 | 2 |
|  |  | (GA)4 | 4 | 1 | 3 | 0 | 1 | 0 | 1 | 2 |
|  |  | (GA)5 | 1 | 0 | 1 | 0 | 0 | 0 | 0 | 1 |
|  |  | (TA)4 | 26 | 22 | 2 | 2 | 23 | 0 | 1 | 2 |
|  |  | (TA)5 | 2 | 2 | 0 | 0 | 2 | 0 | 0 | 0 |
|  |  | (TA)6 | 5 | 5 | 0 | 0 | 2 | 0 | 1 | 2 |
|  |  | (TA)7 | 6 | 5 | 1 | 0 | 4 | 0 | 1 | 1 |
|  |  | (TA)8 | 1 | 1 | 0 | 0 | 1 | 0 | 0 | 0 |
|  |  | (TA)9 | 1 | 1 | 0 | 0 | 0 | 0 | 0 | 1 |
|  |  | (TA)12 | 1 | 1 | 0 | 0 | 1 | 0 | 0 | 0 |
|  |  | (TC)4 | 2 | 0 | 2 | 0 | 0 | 0 | 2 | 0 |
|  |  | (TC)5 | 1 | 0 | 1 | 0 | 0 | 0 | 1 | 0 |
|  |  | (TG)4 | 1 | 0 | 1 | 0 | 1 | 0 | 0 | 0 |
|  |  | Subtotal | 88 | 64 | 18 | 6 | 62 | 0 | 13 | 13 |
|  | Trinucleotide | (AAT)4 | 1 | 1 | 0 | 0 | 1 | 0 | 0 | 0 |
|  |  | (AGA)4 | 1 | 0 | 1 | 0 | 0 | 0 | 0 | 1 |
|  |  | (ATA)4 | 3 | 3 | 0 | 0 | 3 | 0 | 0 | 0 |
|  |  | (ATT)4 | 3 | 2 | 0 | 1 | 3 | 0 | 0 | 0 |
|  |  | (TAA)4 | 1 | 1 | 0 | 0 | 1 | 0 | 0 | 0 |
|  |  | (TAA)5 | 1 | 1 | 0 | 0 | 1 | 0 | 0 | 0 |
|  |  | (TAT)4 | 3 | 3 | 0 | 0 | 3 | 0 | 0 | 0 |
|  |  | (TCT)4 | 2 | 1 | 1 | 0 | 1 | 0 | 1 | 0 |
|  |  | (TTA)4 | 1 | 1 | 0 | 0 | 1 | 0 | 0 | 0 |
|  |  | Subtotal | 16 | 13 | 2 | 1 | 14 | 0 | 1 | 1 |
|  | Tetranucleotide | (AAAT)3 | 1 | 1 | 0 | 0 | 1 | 0 | 0 | 0 |
|  |  | (AATA)3 | 1 | 1 | 0 | 0 | 1 | 0 | 0 | 0 |
|  |  | (AATA)4 | 1 | 1 | 0 | 0 | 0 | 0 | 1 | 0 |
|  |  | (AATG)3 | 1 | 0 | 0 | 1 | 1 | 0 | 0 | 0 |
|  |  | (ACAT)3 | 1 | 0 | 1 | 0 | 1 | 0 | 0 | 0 |
|  |  | (ATAG)3 | 1 | 1 | 0 | 0 | 0 | 0 | 1 | 0 |
|  |  | (ATTA)3 | 1 | 1 | 0 | 0 | 0 | 0 | 1 | 0 |
|  |  | (ATTC)3 | 1 | 0 | 1 | 0 | 0 | 0 | 0 | 1 |
|  |  | (ATTT)4 | 1 | 1 | 0 | 0 | 1 | 0 | 0 | 0 |
|  |  | (CTTT)3 | 1 | 1 | 0 | 0 | 1 | 0 | 0 | 0 |
|  |  | (GAAT)3 | 1 | 0 | 1 | 0 | 0 | 0 | 1 | 0 |
|  |  | (GTCT)3 | 1 | 0 | 1 | 0 | 1 | 0 | 0 | 0 |
|  |  | (TAAT)3 | 1 | 1 | 0 | 0 | 0 | 0 | 0 | 1 |
|  |  | (TAGA)3 | 1 | 1 | 0 | 0 | 1 | 0 | 0 | 0 |
|  |  | (TCTA)3 | 1 | 1 | 0 | 0 | 0 | 0 | 0 | 1 |
|  |  | (TTAT)3 | 2 | 1 | 0 | 1 | 2 | 0 | 0 | 0 |
|  |  | (TTAT)4 | 1 | 1 | 0 | 0 | 0 | 0 | 0 | 1 |
|  |  | Subtotal | 18 | 12 | 4 | 2 | 10 | 0 | 4 | 4 |
|  | Pentanucleotide | (TATAT)3 | 1 | 1 | 0 | 0 | 1 | 0 | 0 | 0 |
|  |  | (TATTA)3 | 1 | 1 | 0 | 0 | 1 | 0 | 0 | 0 |
|  |  | (TTATA)3 | 1 | 1 | 0 | 0 | 1 | 0 | 0 | 0 |
|  |  | Subtotal | 3 | 3 | 0 | 0 | 3 | 0 | 0 | 0 |
|  | Hexanucleotide | (CCTCTT)7 | 1 | 0 | 1 | 0 | 0 | 0 | 1 | 0 |
|  |  | (CTTCTA)3 | 1 | 0 | 1 | 0 | 0 | 0 | 1 | 0 |
|  |  | (GGAAGA)7 | 1 | 0 | 1 | 0 | 0 | 0 | 0 | 1 |
|  |  | (TAGAAG)3 | 1 | 0 | 1 | 0 | 0 | 0 | 0 | 1 |
|  |  | (TTTATA)3 | 1 | 1 | 0 | 0 | 1 | 0 | 0 | 0 |
|  |  | Subtotal | 5 | 1 | 4 | 0 | 1 | 0 | 2 | 2 |
|  | Subtotal |  | 282 | 160 | 79 | 43 | 190 | 4 | 44 | 44 |
| ***P. violascens*** | Mononucleotide | (A)8 | 30 | 11 | 14 | 5 | 18 | 0 | 4 | 8 |
|  |  | (A)9 | 16 | 9 | 6 | 1 | 10 | 1 | 2 | 3 |
|  |  | (A)10 | 7 | 3 | 3 | 1 | 3 | 0 | 2 | 2 |
|  |  | (A)11 | 7 | 6 | 1 | 0 | 5 | 0 | 1 | 1 |
|  |  | (A)12 | 2 | 1 | 0 | 1 | 2 | 0 | 0 | 0 |
|  |  | (A)13 | 4 | 2 | 2 | 0 | 1 | 1 | 1 | 1 |
|  |  | (A)14 | 1 | 0 | 0 | 1 | 1 | 0 | 0 | 0 |
|  |  | (A)15 | 2 | 2 | 0 | 0 | 2 | 0 | 0 | 0 |
|  |  | (C)8 | 2 | 2 | 0 | 0 | 2 | 0 | 0 | 0 |
|  |  | (C)9 | 2 | 1 | 0 | 1 | 1 | 0 | 1 | 0 |
|  |  | (C)10 | 1 | 0 | 0 | 1 | 1 | 0 | 0 | 0 |
|  |  | (G)8 | 2 | 2 | 0 | 0 | 2 | 0 | 0 | 0 |
|  |  | (G)9 | 2 | 1 | 0 | 1 | 1 | 0 | 0 | 1 |
|  |  | (T)8 | 37 | 14 | 15 | 8 | 25 | 0 | 8 | 4 |
|  |  | (T)9 | 20 | 8 | 7 | 5 | 15 | 0 | 3 | 2 |
|  |  | (T)10 | 14 | 7 | 3 | 4 | 9 | 1 | 2 | 2 |
|  |  | (T)11 | 5 | 3 | 1 | 1 | 3 | 0 | 1 | 1 |
|  |  | (T)13 | 6 | 3 | 1 | 2 | 4 | 0 | 1 | 1 |
|  |  | (T)14 | 2 | 2 | 0 | 0 | 2 | 0 | 0 | 0 |
|  |  | (T)15 | 1 | 1 | 0 | 0 | 1 | 0 | 0 | 0 |
|  |  | (T)16 | 1 | 1 | 0 | 0 | 1 | 0 | 0 | 0 |
|  |  | (T)18 | 1 | 1 | 0 | 0 | 1 | 0 | 0 | 0 |
|  |  | Subtotal | 165 | 80 | 53 | 32 | 110 | 3 | 26 | 26 |
|  | Dinucleotide | (AC)4 | 1 | 1 | 0 | 0 | 1 | 0 | 0 | 0 |
|  |  | (AG)4 | 4 | 1 | 2 | 1 | 1 | 0 | 2 | 1 |
|  |  | (AT)4 | 31 | 25 | 3 | 3 | 31 | 0 | 0 | 0 |
|  |  | (AT)5 | 4 | 3 | 1 | 0 | 3 | 1 | 0 | 0 |
|  |  | (AT)6 | 3 | 3 | 0 | 0 | 1 | 0 | 1 | 1 |
|  |  | (CT)4 | 4 | 1 | 3 | 0 | 1 | 0 | 1 | 2 |
|  |  | (GA)4 | 4 | 1 | 3 | 0 | 1 | 0 | 1 | 2 |
|  |  | (GA)5 | 1 | 0 | 1 | 0 | 0 | 0 | 0 | 1 |
|  |  | (TA)4 | 30 | 22 | 2 | 6 | 27 | 1 | 1 | 1 |
|  |  | (TA)5 | 3 | 1 | 0 | 2 | 3 | 0 | 0 | 0 |
|  |  | (TA)6 | 3 | 3 | 0 | 0 | 3 | 0 | 0 | 0 |
|  |  | (TA)7 | 4 | 2 | 0 | 2 | 4 | 0 | 0 | 0 |
|  |  | (TA)8 | 2 | 2 | 0 | 0 | 2 | 0 | 0 | 0 |
|  |  | (TA)9 | 1 | 1 | 0 | 0 | 1 | 0 | 0 | 0 |
|  |  | (TC)4 | 3 | 1 | 2 | 0 | 0 | 0 | 2 | 1 |
|  |  | (TC)5 | 1 | 0 | 1 | 0 | 0 | 0 | 1 | 0 |
|  |  | (TG)4 | 1 | 0 | 1 | 0 | 1 | 0 | 0 | 0 |
|  |  | Subtotal | 100 | 67 | 19 | 14 | 80 | 2 | 9 | 9 |
|  | Trinucleotide | (AGA)4 | 1 | 0 | 1 | 0 | 0 | 0 | 0 | 1 |
|  |  | (ATA)4 | 4 | 4 | 0 | 0 | 3 | 1 | 0 | 0 |
|  |  | (ATA)6 | 2 | 1 | 0 | 1 | 2 | 0 | 0 | 0 |
|  |  | (ATT)4 | 3 | 1 | 0 | 2 | 3 | 0 | 0 | 0 |
|  |  | (ATT)7 | 1 | 1 | 0 | 0 | 1 | 0 | 0 | 0 |
|  |  | (TAA)4 | 1 | 1 | 0 | 0 | 1 | 0 | 0 | 0 |
|  |  | (TAT)4 | 5 | 5 | 0 | 0 | 5 | 0 | 0 | 0 |
|  |  | (TCT)4 | 2 | 1 | 1 | 0 | 1 | 0 | 1 | 0 |
|  |  | (TTA)4 | 1 | 1 | 0 | 0 | 1 | 0 | 0 | 0 |
|  |  | (TTA)6 | 1 | 1 | 0 | 0 | 1 | 0 | 0 | 0 |
|  |  | Subtotal | 21 | 16 | 2 | 3 | 18 | 1 | 1 | 1 |
|  | Tetranucleotide | (AAAT)3 | 3 | 3 | 0 | 0 | 3 | 0 | 0 | 0 |
|  |  | (AATA)4 | 2 | 2 | 0 | 0 | 1 | 0 | 1 | 0 |
|  |  | (ACAT)3 | 1 | 0 | 1 | 0 | 1 | 0 | 0 | 0 |
|  |  | (ATAG)3 | 1 | 1 | 0 | 0 | 0 | 0 | 1 | 0 |
|  |  | (ATTC)3 | 1 | 0 | 1 | 0 | 0 | 0 | 0 | 1 |
|  |  | (CTTT)3 | 1 | 1 | 0 | 0 | 1 | 0 | 0 | 0 |
|  |  | (GAAT)3 | 1 | 0 | 1 | 0 | 0 | 0 | 1 | 0 |
|  |  | (GTCT)3 | 1 | 0 | 1 | 0 | 1 | 0 | 0 | 0 |
|  |  | (TAAA)4 | 1 | 1 | 0 | 0 | 1 | 0 | 0 | 0 |
|  |  | (TCTA)3 | 1 | 1 | 0 | 0 | 0 | 0 | 0 | 1 |
|  |  | (TTAT)3 | 4 | 2 | 0 | 2 | 4 | 0 | 0 | 0 |
|  |  | (TTAT)4 | 2 | 1 | 0 | 1 | 1 | 0 | 0 | 1 |
|  |  | (TTTA)3 | 1 | 1 | 0 | 0 | 0 | 1 | 0 | 0 |
|  |  | Subtotal | 20 | 13 | 4 | 3 | 13 | 1 | 3 | 3 |
|  | Pentanucleotide | (AAATA)3 | 1 | 1 | 0 | 0 | 1 | 0 | 0 | 0 |
|  |  | (AATAT)4 | 1 | 1 | 0 | 0 | 1 | 0 | 0 | 0 |
|  |  | (ATATA)3 | 1 | 1 | 0 | 0 | 1 | 0 | 0 | 0 |
|  |  | (ATATT)3 | 2 | 1 | 0 | 1 | 2 | 0 | 0 | 0 |
|  |  | (ATTAT)3 | 1 | 0 | 0 | 1 | 1 | 0 | 0 | 0 |
|  |  | (TATAC)3 | 1 | 0 | 0 | 1 | 1 | 0 | 0 | 0 |
|  |  | (TATAT)3 | 1 | 0 | 0 | 1 | 1 | 0 | 0 | 0 |
|  |  | (TTATA)3 | 1 | 0 | 0 | 1 | 1 | 0 | 0 | 0 |
|  |  | Subtotal | 9 | 4 | 0 | 5 | 9 | 0 | 0 | 0 |
|  | Hexanucleotide | (ATATTA)3 | 1 | 1 | 0 | 0 | 1 | 0 | 0 | 0 |
|  |  | (CAAATA)3 | 1 | 1 | 0 | 0 | 1 | 0 | 0 | 0 |
|  |  | (CCTCTT)3 | 1 | 0 | 1 | 0 | 0 | 0 | 1 | 0 |
|  |  | (CTTCTA)3 | 1 | 0 | 1 | 0 | 0 | 0 | 1 | 0 |
|  |  | (GGAAGA)3 | 1 | 0 | 1 | 0 | 0 | 0 | 0 | 1 |
|  |  | (TAGAAG)3 | 1 | 0 | 1 | 0 | 0 | 0 | 0 | 1 |
|  |  | Subtotal | 6 | 2 | 4 | 0 | 2 | 0 | 2 | 2 |
|  | Subtotal |  | 321 | 182 | 82 | 57 | 232 | 7 | 41 | 41 |
| ***P. niveum*** | Mononucleotide | (A)8 | 29 | 11 | 13 | 5 | 17 | 9 | 2 | 1 |
|  |  | (A)9 | 19 | 9 | 8 | 2 | 12 | 5 | 1 | 1 |
|  |  | (A)10 | 7 | 5 | 2 | 0 | 5 | 2 | 0 | 0 |
|  |  | (A)11 | 6 | 3 | 3 | 0 | 5 | 1 | 0 | 0 |
|  |  | (A)12 | 4 | 4 | 0 | 0 | 2 | 1 | 1 | 0 |
|  |  | (A)13 | 4 | 2 | 1 | 1 | 3 | 1 | 0 | 0 |
|  |  | (A)16 | 1 | 0 | 0 | 1 | 1 | 0 | 0 | 0 |
|  |  | (A)17 | 1 | 1 | 0 | 0 | 1 | 0 | 0 | 0 |
|  |  | (A)18 | 1 | 1 | 0 | 0 | 1 | 0 | 0 | 0 |
|  |  | (A)19 | 1 | 1 | 0 | 0 | 0 | 1 | 0 | 0 |
|  |  | (A)21 | 1 | 1 | 0 | 0 | 1 | 0 | 0 | 0 |
|  |  | (C)8 | 1 | 1 | 0 | 0 | 1 | 0 | 0 | 0 |
|  |  | (C)9 | 2 | 0 | 0 | 2 | 2 | 0 | 0 | 0 |
|  |  | (C)11 | 1 | 0 | 0 | 1 | 0 | 1 | 0 | 0 |
|  |  | (G)9 | 2 | 2 | 0 | 0 | 2 | 0 | 0 | 0 |
|  |  | (G)11 | 1 | 0 | 0 | 1 | 0 | 1 | 0 | 0 |
|  |  | (T)8 | 39 | 16 | 14 | 9 | 28 | 8 | 1 | 2 |
|  |  | (T)9 | 23 | 9 | 8 | 6 | 17 | 4 | 1 | 1 |
|  |  | (T)10 | 10 | 5 | 3 | 2 | 7 | 3 | 0 | 0 |
|  |  | (T)11 | 5 | 1 | 1 | 3 | 4 | 1 | 0 | 0 |
|  |  | (T)12 | 6 | 4 | 0 | 2 | 4 | 1 | 0 | 1 |
|  |  | (T)13 | 1 | 0 | 1 | 0 | 0 | 1 | 0 | 0 |
|  |  | (T)14 | 2 | 1 | 0 | 1 | 2 | 0 | 0 | 0 |
|  |  | (T)15 | 2 | 1 | 0 | 1 | 2 | 0 | 0 | 0 |
|  |  | (T)25 | 1 | 1 | 0 | 0 | 0 | 1 | 0 | 0 |
|  |  | Subtotal | 170 | 79 | 54 | 37 | 117 | 41 | 6 | 6 |
|  | Dinucleotide | (AC)4 | 1 | 1 | 0 | 0 | 1 | 0 | 0 | 0 |
|  |  | (AG)4 | 4 | 0 | 1 | 3 | 1 | 2 | 0 | 1 |
|  |  | (AT)4 | 17 | 12 | 3 | 2 | 17 | 0 | 0 | 0 |
|  |  | (AT)5 | 3 | 1 | 1 | 1 | 3 | 0 | 0 | 0 |
|  |  | (AT)6 | 2 | 2 | 0 | 0 | 1 | 0 | 1 | 0 |
|  |  | (AT)7 | 2 | 2 | 0 | 0 | 2 | 0 | 0 | 0 |
|  |  | (AT)8 | 1 | 1 | 0 | 0 | 0 | 1 | 0 | 0 |
|  |  | (AT)9 | 1 | 1 | 0 | 0 | 1 | 0 | 0 | 0 |
|  |  | (CT)4 | 4 | 1 | 2 | 1 | 1 | 1 | 1 | 1 |
|  |  | (GA)4 | 4 | 1 | 3 | 0 | 1 | 0 | 1 | 2 |
|  |  | (GA)5 | 1 | 0 | 1 | 0 | 0 | 0 | 0 | 1 |
|  |  | (TA)4 | 26 | 20 | 2 | 4 | 24 | 0 | 1 | 1 |
|  |  | (TA)5 | 7 | 6 | 0 | 1 | 7 | 0 | 0 | 0 |
|  |  | (TA)6 | 4 | 3 | 0 | 1 | 3 | 0 | 0 | 1 |
|  |  | (TA)7 | 5 | 4 | 0 | 1 | 5 | 0 | 0 | 0 |
|  |  | (TA)8 | 2 | 1 | 0 | 1 | 2 | 0 | 0 | 0 |
|  |  | (TC)4 | 3 | 0 | 3 | 0 | 1 | 0 | 2 | 0 |
|  |  | (TC)5 | 1 | 0 | 1 | 0 | 0 | 0 | 1 | 0 |
|  |  | (TG)4 | 1 | 0 | 1 | 0 | 1 | 0 | 0 | 0 |
|  |  | Subtotal | 89 | 56 | 18 | 15 | 71 | 4 | 7 | 7 |
|  | Trinucleotide | (AAT)4 | 3 | 3 | 0 | 0 | 3 | 0 | 0 | 0 |
|  |  | (AGA)4 | 1 | 0 | 1 | 0 | 0 | 1 | 0 | 0 |
|  |  | (ATA)4 | 4 | 2 | 0 | 2 | 4 | 0 | 0 | 0 |
|  |  | (ATA)5 | 3 | 3 | 0 | 0 | 3 | 0 | 0 | 0 |
|  |  | (ATT)4 | 1 | 0 | 0 | 1 | 1 | 0 | 0 | 0 |
|  |  | (ATT)5 | 1 | 1 | 0 | 0 | 1 | 0 | 0 | 0 |
|  |  | (TAA)4 | 1 | 1 | 0 | 0 | 1 | 0 | 0 | 0 |
|  |  | (TAT)4 | 3 | 3 | 0 | 0 | 3 | 0 | 0 | 0 |
|  |  | (TCT)4 | 2 | 1 | 1 | 0 | 1 | 1 | 0 | 0 |
|  |  | Subtotal | 19 | 14 | 2 | 3 | 17 | 2 | 0 | 0 |
|  | Tetranucleotide | (AAAT)3 | 1 | 1 | 0 | 0 | 1 | 0 | 0 | 0 |
|  |  | (AATG)3 | 1 | 0 | 1 | 0 | 1 | 0 | 0 | 0 |
|  |  | (ACAT)3 | 1 | 0 | 1 | 0 | 1 | 0 | 0 | 0 |
|  |  | (ATAG)3 | 3 | 3 | 0 | 0 | 2 | 1 | 0 | 0 |
|  |  | (ATTC)3 | 1 | 0 | 1 | 0 | 0 | 1 | 0 | 0 |
|  |  | (CTTT)3 | 1 | 1 | 0 | 0 | 1 | 0 | 0 | 0 |
|  |  | (GAAT)3 | 1 | 0 | 1 | 0 | 0 | 1 | 0 | 0 |
|  |  | (GTCT)3 | 1 | 0 | 1 | 0 | 1 | 0 | 0 | 0 |
|  |  | (TAAT)4 | 2 | 2 | 0 | 0 | 2 | 0 | 0 | 0 |
|  |  | (TATG)3 | 1 | 1 | 0 | 0 | 1 | 0 | 0 | 0 |
|  |  | (TATT)3 | 1 | 0 | 0 | 1 | 1 | 0 | 0 | 0 |
|  |  | (TCTA)3 | 1 | 1 | 0 | 0 | 0 | 1 | 0 | 0 |
|  |  | (TTAT)3 | 4 | 3 | 0 | 1 | 3 | 1 | 0 | 0 |
|  |  | (TTTA)3 | 1 | 1 | 0 | 0 | 0 | 1 | 0 | 0 |
|  |  | Subtotal | 20 | 13 | 5 | 2 | 14 | 6 | 0 | 0 |
|  | Pentanucleotide | (ATAAT)3 | 2 | 2 | 0 | 0 | 2 | 0 | 0 | 0 |
|  |  | (ATATT)3 | 1 | 0 | 0 | 1 | 1 | 0 | 0 | 0 |
|  |  | (CCAGA)3 | 1 | 1 | 0 | 0 | 0 | 0 | 1 | 0 |
|  |  | (GGTCT)3 | 1 | 1 | 0 | 0 | 0 | 0 | 0 | 1 |
|  |  | (TAAAA)3 | 1 | 1 | 0 | 0 | 1 | 0 | 0 | 0 |
|  |  | (TATAC)3 | 1 | 1 | 0 | 0 | 1 | 0 | 0 | 0 |
|  |  | (TATTG)3 | 1 | 1 | 0 | 0 | 1 | 0 | 0 | 0 |
|  |  | (TATTT)3 | 1 | 1 | 0 | 0 | 1 | 0 | 0 | 0 |
|  |  | Subtotal | 9 | 8 | 0 | 1 | 7 | 0 | 1 | 1 |
|  | Hexanucleotide | (ATTAAA)3 | 1 | 0 | 1 | 0 | 0 | 1 | 0 | 0 |
|  |  | (ATTTAT)3 | 1 | 1 | 0 | 0 | 1 | 0 | 0 | 0 |
|  |  | (CTTCTA)3 | 1 | 0 | 1 | 0 | 0 | 0 | 1 | 0 |
|  |  | (TAGAAG)3 | 1 | 0 | 1 | 0 | 0 | 0 | 0 | 1 |
|  |  | (TTTTAA)3 | 1 | 0 | 1 | 0 | 0 | 1 | 0 | 0 |
|  |  | Subtotal | 5 | 1 | 4 | 0 | 1 | 2 | 1 | 1 |
|  | Subtotal |  | 312 | 171 | 83 | 58 | 227 | 55 | 15 | 15 |
| ***P. kolopakingii*** | Mononucleotide | (A)8 | 22 | 6 | 14 | 2 | 11 | 0 | 4 | 7 |
|  |  | (A)9 | 22 | 12 | 8 | 2 | 12 | 1 | 4 | 5 |
|  |  | (A)10 | 8 | 5 | 3 | 0 | 5 | 0 | 2 | 1 |
|  |  | (A)11 | 4 | 3 | 1 | 0 | 3 | 0 | 0 | 1 |
|  |  | (A)12 | 2 | 2 | 0 | 0 | 2 | 0 | 0 | 0 |
|  |  | (A)13 | 2 | 1 | 1 | 0 | 1 | 0 | 0 | 1 |
|  |  | (A)14 | 2 | 1 | 0 | 1 | 2 | 0 | 0 | 0 |
|  |  | (A)15 | 3 | 3 | 0 | 0 | 2 | 0 | 1 | 0 |
|  |  | (A)16 | 1 | 1 | 0 | 0 | 1 | 0 | 0 | 0 |
|  |  | (A)20 | 1 | 0 | 0 | 1 | 1 | 0 | 0 | 0 |
|  |  | (C)10 | 2 | 0 | 0 | 2 | 1 | 0 | 1 | 0 |
|  |  | (C)11 | 1 | 1 | 0 | 0 | 1 | 0 | 0 | 0 |
|  |  | (G)8 | 1 | 1 | 0 | 0 | 1 | 0 | 0 | 0 |
|  |  | (G)10 | 1 | 0 | 0 | 1 | 0 | 0 | 0 | 1 |
|  |  | (T)8 | 44 | 22 | 14 | 8 | 33 | 0 | 7 | 4 |
|  |  | (T)9 | 24 | 9 | 10 | 5 | 14 | 1 | 5 | 4 |
|  |  | (T)10 | 9 | 6 | 2 | 1 | 4 | 2 | 1 | 2 |
|  |  | (T)11 | 8 | 5 | 1 | 2 | 7 | 0 | 1 | 0 |
|  |  | (T)12 | 5 | 5 | 0 | 0 | 5 | 0 | 0 | 0 |
|  |  | (T)13 | 1 | 0 | 1 | 0 | 0 | 0 | 1 | 0 |
|  |  | (T)14 | 4 | 1 | 0 | 3 | 4 | 0 | 0 | 0 |
|  |  | (T)15 | 2 | 1 | 0 | 1 | 1 | 0 | 0 | 1 |
|  |  | (T)18 | 1 | 1 | 0 | 0 | 1 | 0 | 0 | 0 |
|  |  | Subtotal | 170 | 86 | 55 | 29 | 112 | 4 | 27 | 27 |
|  | Dinucleotide | (AC)4 | 1 | 1 | 0 | 0 | 1 | 0 | 0 | 0 |
|  |  | (AG)4 | 4 | 1 | 2 | 1 | 1 | 0 | 2 | 1 |
|  |  | (AT)4 | 26 | 22 | 3 | 1 | 24 | 0 | 2 | 0 |
|  |  | (AT)5 | 6 | 5 | 1 | 0 | 5 | 0 | 1 | 0 |
|  |  | (AT)7 | 5 | 5 | 0 | 0 | 1 | 0 | 3 | 1 |
|  |  | (CT)4 | 4 | 1 | 3 | 0 | 1 | 0 | 1 | 2 |
|  |  | (GA)4 | 4 | 1 | 3 | 0 | 1 | 0 | 1 | 2 |
|  |  | (GA)5 | 1 | 0 | 1 | 0 | 0 | 0 | 0 | 1 |
|  |  | (TA)4 | 31 | 24 | 2 | 5 | 22 | 3 | 2 | 4 |
|  |  | (TA)5 | 7 | 6 | 0 | 1 | 6 | 0 | 0 | 1 |
|  |  | (TA)6 | 5 | 5 | 0 | 0 | 5 | 0 | 0 | 0 |
|  |  | (TA)7 | 4 | 3 | 1 | 0 | 2 | 0 | 0 | 2 |
|  |  | (TA)8 | 3 | 2 | 0 | 1 | 3 | 0 | 0 | 0 |
|  |  | (TC)4 | 3 | 1 | 2 | 0 | 0 | 0 | 2 | 1 |
|  |  | (TC)5 | 1 | 0 | 1 | 0 | 0 | 0 | 1 | 0 |
|  |  | (TG)4 | 1 | 0 | 1 | 0 | 1 | 0 | 0 | 0 |
|  |  | Subtotal | 106 | 77 | 20 | 9 | 73 | 3 | 15 | 15 |
|  | Trinucleotide | (AAT)4 | 3 | 1 | 0 | 2 | 3 | 0 | 0 | 0 |
|  |  | (AGA)4 | 1 | 0 | 1 | 0 | 0 | 0 | 0 | 1 |
|  |  | (ATA)4 | 4 | 2 | 0 | 2 | 4 | 0 | 0 | 0 |
|  |  | (ATT)4 | 2 | 1 | 0 | 1 | 2 | 0 | 0 | 0 |
|  |  | (TAA)4 | 1 | 1 | 0 | 0 | 1 | 0 | 0 | 0 |
|  |  | (TAT)4 | 4 | 3 | 0 | 1 | 4 | 0 | 0 | 0 |
|  |  | (TAT)5 | 2 | 2 | 0 | 0 | 2 | 0 | 0 | 0 |
|  |  | (TCT)4 | 2 | 1 | 1 | 0 | 1 | 0 | 1 | 0 |
|  |  | (TTA)4 | 2 | 2 | 0 | 0 | 2 | 0 | 0 | 0 |
|  |  | Subtotal | 21 | 13 | 2 | 6 | 19 | 0 | 1 | 1 |
|  | Tetranucleotide | (AAAT)3 | 1 | 1 | 0 | 0 | 1 | 0 | 0 | 0 |
|  |  | (AATA)3 | 2 | 2 | 0 | 0 | 1 | 0 | 1 | 0 |
|  |  | (AATG)3 | 1 | 1 | 0 | 0 | 1 | 0 | 0 | 0 |
|  |  | (ACAT)3 | 1 | 0 | 1 | 0 | 1 | 0 | 0 | 0 |
|  |  | (ATAA)3 | 1 | 1 | 0 | 0 | 1 | 0 | 0 | 0 |
|  |  | (ATAG)3 | 1 | 1 | 0 | 0 | 0 | 0 | 1 | 0 |
|  |  | (ATTA)4 | 1 | 1 | 0 | 0 | 1 | 0 | 0 | 0 |
|  |  | (ATTC)3 | 1 | 0 | 1 | 0 | 0 | 0 | 0 | 1 |
|  |  | (CTTT)3 | 1 | 1 | 0 | 0 | 1 | 0 | 0 | 0 |
|  |  | (GAAT)3 | 1 | 0 | 1 | 0 | 0 | 0 | 1 | 0 |
|  |  | (GATA)3 | 1 | 1 | 0 | 0 | 1 | 0 | 0 | 0 |
|  |  | (GTCT)3 | 1 | 0 | 1 | 0 | 1 | 0 | 0 | 0 |
|  |  | (TAAA)3 | 1 | 1 | 0 | 0 | 1 | 0 | 0 | 0 |
|  |  | (TCTA)3 | 1 | 1 | 0 | 0 | 0 | 0 | 0 | 1 |
|  |  | (TTAT)3 | 4 | 2 | 0 | 2 | 3 | 0 | 0 | 1 |
|  |  | (TTAT)4 | 1 | 1 | 0 | 0 | 1 | 0 | 0 | 0 |
|  |  | Subtotal | 20 | 14 | 4 | 2 | 14 | 0 | 3 | 3 |
|  | Pentanucleotide | (AAATA)3 | 1 | 1 | 0 | 0 | 1 | 0 | 0 | 0 |
|  |  | (ATATA)3 | 1 | 1 | 0 | 0 | 1 | 0 | 0 | 0 |
|  |  | (ATATT)3 | 2 | 2 | 0 | 0 | 0 | 0 | 0 | 2 |
|  |  | (ATCAT)3 | 1 | 0 | 0 | 1 | 1 | 0 | 0 | 0 |
|  |  | (TAATA)3 | 3 | 3 | 0 | 0 | 0 | 0 | 3 | 0 |
|  |  | (TATAT)3 | 1 | 1 | 0 | 0 | 0 | 0 | 0 | 1 |
|  |  | (TTTTA)3 | 1 | 1 | 0 | 0 | 1 | 0 | 0 | 0 |
|  |  | Subtotal | 10 | 9 | 0 | 1 | 4 | 0 | 3 | 3 |
|  | Hexanucleotide | (CCTCTT)6 | 1 | 0 | 1 | 0 | 0 | 0 | 1 | 0 |
|  |  | (CTTCTA)3 | 1 | 0 | 1 | 0 | 0 | 0 | 1 | 0 |
|  |  | (GGAAGA)6 | 1 | 0 | 1 | 0 | 0 | 0 | 0 | 1 |
|  |  | (TAGAAG)3 | 1 | 0 | 1 | 0 | 0 | 0 | 0 | 1 |
|  |  | (TATTAG)3 | 1 | 1 | 0 | 0 | 1 | 0 | 0 | 0 |
|  |  | Subtotal | 5 | 1 | 4 | 0 | 1 | 0 | 2 | 2 |
|  | Subtotal |  | 332 | 200 | 85 | 47 | 223 | 7 | 51 | 51 |
| ***P. hirsutissimum*** | Mononucleotide | (A)8 | 29 | 9 | 18 | 2 | 15 | 10 | 1 | 3 |
|  |  | (A)9 | 15 | 8 | 5 | 2 | 8 | 3 | 3 | 1 |
|  |  | (A)10 | 12 | 5 | 6 | 1 | 8 | 1 | 1 | 2 |
|  |  | (A)11 | 5 | 4 | 1 | 0 | 3 | 1 | 1 | 0 |
|  |  | (A)12 | 4 | 4 | 0 | 0 | 3 | 1 | 0 | 0 |
|  |  | (A)13 | 2 | 1 | 0 | 1 | 2 | 0 | 0 | 0 |
|  |  | (A)15 | 1 | 1 | 0 | 0 | 0 | 1 | 0 | 0 |
|  |  | (A)19 | 1 | 1 | 0 | 0 | 1 | 0 | 0 | 0 |
|  |  | (C)8 | 2 | 2 | 0 | 0 | 2 | 0 | 0 | 0 |
|  |  | (C)9 | 2 | 0 | 0 | 2 | 1 | 0 | 1 | 0 |
|  |  | (G)8 | 1 | 1 | 0 | 0 | 1 | 0 | 0 | 0 |
|  |  | (G)9 | 2 | 1 | 0 | 1 | 1 | 0 | 0 | 1 |
|  |  | (T)8 | 35 | 9 | 20 | 6 | 21 | 10 | 3 | 1 |
|  |  | (T)9 | 25 | 11 | 7 | 7 | 18 | 3 | 1 | 3 |
|  |  | (T)10 | 16 | 5 | 4 | 7 | 12 | 1 | 2 | 1 |
|  |  | (T)11 | 7 | 3 | 2 | 2 | 5 | 1 | 0 | 1 |
|  |  | (T)12 | 2 | 2 | 0 | 0 | 1 | 1 | 0 | 0 |
|  |  | (T)14 | 1 | 0 | 0 | 1 | 1 | 0 | 0 | 0 |
|  |  | (T)15 | 2 | 2 | 0 | 0 | 1 | 1 | 0 | 0 |
|  |  | (T)16 | 1 | 1 | 0 | 0 | 1 | 0 | 0 | 0 |
|  |  | (T)17 | 1 | 1 | 0 | 0 | 1 | 0 | 0 | 0 |
|  |  | (T)23 | 1 | 1 | 0 | 0 | 1 | 0 | 0 | 0 |
|  |  | Subtotal | 167 | 72 | 63 | 32 | 107 | 34 | 13 | 13 |
|  | Dinucleotide | (AC)4 | 1 | 1 | 0 | 0 | 1 | 0 | 0 | 0 |
|  |  | (AG)4 | 4 | 0 | 2 | 2 | 1 | 1 | 1 | 1 |
|  |  | (AT)4 | 15 | 10 | 3 | 2 | 15 | 0 | 0 | 0 |
|  |  | (AT)5 | 2 | 1 | 1 | 0 | 2 | 0 | 0 | 0 |
|  |  | (AT)7 | 1 | 1 | 0 | 0 | 0 | 0 | 1 | 0 |
|  |  | (AT)8 | 1 | 1 | 0 | 0 | 1 | 0 | 0 | 0 |
|  |  | (CT)4 | 4 | 1 | 3 | 0 | 1 | 0 | 1 | 2 |
|  |  | (GA)4 | 4 | 1 | 3 | 0 | 1 | 0 | 1 | 2 |
|  |  | (GA)5 | 1 | 0 | 1 | 0 | 0 | 0 | 0 | 1 |
|  |  | (TA)4 | 18 | 13 | 2 | 3 | 16 | 0 | 1 | 1 |
|  |  | (TA)6 | 4 | 3 | 0 | 1 | 4 | 0 | 0 | 0 |
|  |  | (TA)7 | 2 | 2 | 0 | 0 | 1 | 0 | 0 | 1 |
|  |  | (TA)9 | 1 | 1 | 0 | 0 | 1 | 0 | 0 | 0 |
|  |  | (TC)4 | 4 | 1 | 2 | 1 | 1 | 1 | 2 | 0 |
|  |  | (TC)5 | 1 | 0 | 1 | 0 | 0 | 0 | 1 | 0 |
|  |  | (TG)4 | 1 | 0 | 1 | 0 | 1 | 0 | 0 | 0 |
|  |  | Subtotal | 64 | 36 | 19 | 9 | 46 | 2 | 8 | 8 |
|  | Trinucleotide | (AAT)4 | 2 | 2 | 0 | 0 | 2 | 0 | 0 | 0 |
|  |  | (AGA)4 | 1 | 0 | 1 | 0 | 0 | 0 | 0 | 1 |
|  |  | (ATA)4 | 3 | 3 | 0 | 0 | 3 | 0 | 0 | 0 |
|  |  | (ATT)4 | 3 | 2 | 0 | 1 | 3 | 0 | 0 | 0 |
|  |  | (ATT)5 | 1 | 1 | 0 | 0 | 1 | 0 | 0 | 0 |
|  |  | (TAA)4 | 1 | 1 | 0 | 0 | 1 | 0 | 0 | 0 |
|  |  | (TCT)4 | 2 | 1 | 1 | 0 | 1 | 0 | 1 | 0 |
|  |  | (TTA)4 | 2 | 0 | 0 | 2 | 2 | 0 | 0 | 0 |
|  |  | Subtotal | 15 | 10 | 2 | 3 | 13 | 0 | 1 | 1 |
|  | Tetranucleotide | (AAAT)3 | 2 | 1 | 0 | 1 | 1 | 1 | 0 | 0 |
|  |  | (AAAT)4 | 1 | 1 | 0 | 0 | 1 | 0 | 0 | 0 |
|  |  | (AATA)4 | 1 | 1 | 0 | 0 | 0 | 0 | 1 | 0 |
|  |  | (AATT)3 | 1 | 1 | 0 | 0 | 1 | 0 | 0 | 0 |
|  |  | (ACAT)3 | 1 | 0 | 1 | 0 | 1 | 0 | 0 | 0 |
|  |  | (ATAG)3 | 1 | 1 | 0 | 0 | 0 | 0 | 1 | 0 |
|  |  | (ATTC)3 | 1 | 0 | 1 | 0 | 0 | 1 | 0 | 0 |
|  |  | (CTTT)3 | 1 | 1 | 0 | 0 | 1 | 0 | 0 | 0 |
|  |  | (GAAT)3 | 1 | 0 | 1 | 0 | 0 | 1 | 0 | 0 |
|  |  | (GTCT)3 | 1 | 0 | 1 | 0 | 1 | 0 | 0 | 0 |
|  |  | (TAAA)3 | 1 | 1 | 0 | 0 | 1 | 0 | 0 | 0 |
|  |  | (TAAT)3 | 1 | 1 | 0 | 0 | 1 | 0 | 0 | 0 |
|  |  | (TATT)3 | 1 | 0 | 0 | 1 | 1 | 0 | 0 | 0 |
|  |  | (TCTA)3 | 1 | 1 | 0 | 0 | 0 | 0 | 0 | 1 |
|  |  | (TTAT)3 | 2 | 2 | 0 | 0 | 2 | 0 | 0 | 0 |
|  |  | (TTAT)4 | 1 | 1 | 0 | 0 | 0 | 0 | 0 | 1 |
|  |  | (TTTA)3 | 1 | 1 | 0 | 0 | 0 | 1 | 0 | 0 |
|  |  | Subtotal | 19 | 13 | 4 | 2 | 11 | 4 | 2 | 2 |
|  | Pentanucleotide | (AAATA)3 | 1 | 1 | 0 | 0 | 1 | 0 | 0 | 0 |
|  |  | (ATAAT)3 | 1 | 1 | 0 | 0 | 1 | 0 | 0 | 0 |
|  |  | (ATATT)3 | 1 | 0 | 0 | 1 | 1 | 0 | 0 | 0 |
|  |  | (ATTTT)3 | 1 | 1 | 0 | 0 | 1 | 0 | 0 | 0 |
|  |  | (TATAC)3 | 1 | 0 | 0 | 1 | 1 | 0 | 0 | 0 |
|  |  | (TTATA)4 | 2 | 2 | 0 | 0 | 2 | 0 | 0 | 0 |
|  |  | Subtotal | 7 | 5 | 0 | 2 | 7 | 0 | 0 | 0 |
|  | Hexanucleotide | (CCTCTT)22 | 1 | 0 | 1 | 0 | 0 | 0 | 1 | 0 |
|  |  | (CTTCTA)3 | 1 | 0 | 1 | 0 | 0 | 0 | 1 | 0 |
|  |  | (GGAAGA)21 | 1 | 0 | 1 | 0 | 0 | 0 | 0 | 1 |
|  |  | (TAGAAG)3 | 1 | 0 | 1 | 0 | 0 | 0 | 0 | 1 |
|  |  | Subtotal | 4 | 0 | 4 | 0 | 0 | 0 | 2 | 2 |
|  | Subtotal |  | 276 | 136 | 92 | 48 | 184 | 40 | 26 | 26 |
| ***P. philippinense*** | Mononucleotide | (A)8 | 27 | 10 | 13 | 4 | 16 | 0 | 4 | 7 |
|  |  | (A)9 | 19 | 9 | 9 | 1 | 9 | 1 | 4 | 5 |
|  |  | (A)10 | 10 | 4 | 3 | 3 | 7 | 0 | 2 | 1 |
|  |  | (A)11 | 2 | 1 | 1 | 0 | 1 | 0 | 0 | 1 |
|  |  | (A)12 | 3 | 2 | 0 | 1 | 3 | 0 | 0 | 0 |
|  |  | (A)13 | 1 | 0 | 1 | 0 | 0 | 0 | 0 | 1 |
|  |  | (A)14 | 3 | 2 | 0 | 1 | 3 | 0 | 0 | 0 |
|  |  | (A)15 | 1 | 1 | 0 | 0 | 1 | 0 | 0 | 0 |
|  |  | (A)16 | 1 | 1 | 0 | 0 | 1 | 0 | 0 | 0 |
|  |  | (A)18 | 1 | 1 | 0 | 0 | 0 | 0 | 1 | 0 |
|  |  | (A)19 | 1 | 1 | 0 | 0 | 1 | 0 | 0 | 0 |
|  |  | (C)8 | 1 | 1 | 0 | 0 | 1 | 0 | 0 | 0 |
|  |  | (C)9 | 2 | 1 | 0 | 1 | 1 | 0 | 1 | 0 |
|  |  | (C)10 | 1 | 0 | 0 | 1 | 1 | 0 | 0 | 0 |
|  |  | (G)8 | 1 | 1 | 0 | 0 | 1 | 0 | 0 | 0 |
|  |  | (G)9 | 1 | 0 | 0 | 1 | 0 | 0 | 0 | 1 |
|  |  | (T)8 | 40 | 19 | 15 | 6 | 29 | 0 | 7 | 4 |
|  |  | (T)9 | 23 | 7 | 10 | 6 | 12 | 2 | 5 | 4 |
|  |  | (T)10 | 14 | 6 | 2 | 6 | 11 | 0 | 1 | 2 |
|  |  | (T)11 | 6 | 4 | 2 | 0 | 5 | 0 | 1 | 0 |
|  |  | (T)12 | 6 | 3 | 0 | 3 | 6 | 0 | 0 | 0 |
|  |  | (T)13 | 3 | 2 | 1 | 0 | 2 | 0 | 1 | 0 |
|  |  | (T)14 | 1 | 1 | 0 | 0 | 1 | 0 | 0 | 0 |
|  |  | (T)15 | 1 | 0 | 0 | 1 | 1 | 0 | 0 | 0 |
|  |  | (T)16 | 1 | 0 | 0 | 1 | 1 | 0 | 0 | 0 |
|  |  | (T)18 | 2 | 1 | 0 | 1 | 1 | 0 | 0 | 1 |
|  |  | (T)25 | 1 | 1 | 0 | 0 | 0 | 1 | 0 | 0 |
|  |  | Subtotal | 173 | 79 | 57 | 37 | 115 | 4 | 27 | 27 |
|  | Dinucleotide | (AC)4 | 1 | 1 | 0 | 0 | 1 | 0 | 0 | 0 |
|  |  | (AG)4 | 4 | 1 | 2 | 1 | 1 | 0 | 2 | 1 |
|  |  | (AT)4 | 26 | 21 | 3 | 2 | 25 | 0 | 1 | 0 |
|  |  | (AT)5 | 4 | 3 | 1 | 0 | 4 | 0 | 0 | 0 |
|  |  | (AT)7 | 2 | 2 | 0 | 0 | 0 | 0 | 1 | 1 |
|  |  | (AT)9 | 2 | 2 | 0 | 0 | 2 | 0 | 0 | 0 |
|  |  | (CT)4 | 4 | 1 | 3 | 0 | 1 | 0 | 1 | 2 |
|  |  | (GA)4 | 4 | 1 | 3 | 0 | 1 | 0 | 1 | 2 |
|  |  | (GA)5 | 1 | 0 | 1 | 0 | 0 | 0 | 0 | 1 |
|  |  | (TA)4 | 27 | 19 | 2 | 6 | 22 | 2 | 1 | 2 |
|  |  | (TA)5 | 8 | 5 | 0 | 3 | 8 | 0 | 0 | 0 |
|  |  | (TA)6 | 3 | 2 | 0 | 1 | 3 | 0 | 0 | 0 |
|  |  | (TA)7 | 6 | 5 | 1 | 0 | 5 | 1 | 0 | 0 |
|  |  | (TA)10 | 1 | 1 | 0 | 0 | 1 | 0 | 0 | 0 |
|  |  | (TA)15 | 1 | 1 | 0 | 0 | 1 | 0 | 0 | 0 |
|  |  | (TC)4 | 3 | 1 | 2 | 0 | 0 | 0 | 2 | 1 |
|  |  | (TC)5 | 1 | 0 | 1 | 0 | 0 | 0 | 1 | 0 |
|  |  | (TG)4 | 1 | 0 | 1 | 0 | 1 | 0 | 0 | 0 |
|  |  | Subtotal | 99 | 66 | 20 | 13 | 76 | 3 | 10 | 10 |
|  | Trinucleotide | (AAT)4 | 2 | 1 | 0 | 1 | 2 | 0 | 0 | 0 |
|  |  | (AGA)4 | 1 | 0 | 1 | 0 | 0 | 0 | 0 | 1 |
|  |  | (ATA)4 | 2 | 2 | 0 | 0 | 2 | 0 | 0 | 0 |
|  |  | (ATA)5 | 1 | 0 | 0 | 1 | 1 | 0 | 0 | 0 |
|  |  | (ATT)4 | 1 | 0 | 0 | 1 | 1 | 0 | 0 | 0 |
|  |  | (TAA)4 | 1 | 1 | 0 | 0 | 1 | 0 | 0 | 0 |
|  |  | (TAT)4 | 4 | 2 | 0 | 2 | 3 | 1 | 0 | 0 |
|  |  | (TAT)5 | 1 | 1 | 0 | 0 | 1 | 0 | 0 | 0 |
|  |  | (TCT)4 | 2 | 1 | 1 | 0 | 1 | 0 | 1 | 0 |
|  |  | (TTA)4 | 2 | 1 | 0 | 1 | 2 | 0 | 0 | 0 |
|  |  | Subtotal | 17 | 9 | 2 | 6 | 14 | 1 | 1 | 1 |
|  | Tetranucleotide | (AAAT)3 | 1 | 1 | 0 | 0 | 1 | 0 | 0 | 0 |
|  |  | (AATA)6 | 1 | 1 | 0 | 0 | 0 | 0 | 1 | 0 |
|  |  | (AATG)3 | 1 | 1 | 0 | 0 | 1 | 0 | 0 | 0 |
|  |  | (ACAT)3 | 1 | 0 | 1 | 0 | 1 | 0 | 0 | 0 |
|  |  | (ATAA)3 | 3 | 3 | 0 | 0 | 3 | 0 | 0 | 0 |
|  |  | (ATAG)3 | 1 | 1 | 0 | 0 | 0 | 0 | 1 | 0 |
|  |  | (ATTC)3 | 1 | 0 | 1 | 0 | 0 | 0 | 0 | 1 |
|  |  | (ATTT)3 | 1 | 1 | 0 | 0 | 1 | 0 | 0 | 0 |
|  |  | (CTTT)3 | 1 | 1 | 0 | 0 | 1 | 0 | 0 | 0 |
|  |  | (GAAT)3 | 1 | 0 | 1 | 0 | 0 | 0 | 1 | 0 |
|  |  | (GATA)3 | 1 | 1 | 0 | 0 | 1 | 0 | 0 | 0 |
|  |  | (GTCT)3 | 1 | 0 | 1 | 0 | 1 | 0 | 0 | 0 |
|  |  | (TAAA)3 | 1 | 1 | 0 | 0 | 1 | 0 | 0 | 0 |
|  |  | (TCTA)3 | 1 | 1 | 0 | 0 | 0 | 0 | 0 | 1 |
|  |  | (TTAT)3 | 3 | 1 | 0 | 2 | 3 | 0 | 0 | 0 |
|  |  | (TTAT)6 | 1 | 1 | 0 | 0 | 0 | 0 | 0 | 1 |
|  |  | Subtotal | 20 | 14 | 4 | 2 | 14 | 0 | 3 | 3 |
|  | Pentanucleotide | (ATAAT)3 | 1 | 1 | 0 | 0 | 0 | 0 | 1 | 0 |
|  |  | (ATATA)3 | 3 | 1 | 0 | 2 | 2 | 0 | 1 | 0 |
|  |  | (TATAT)3 | 2 | 2 | 0 | 0 | 0 | 0 | 0 | 2 |
|  |  | (TATTG)3 | 1 | 1 | 0 | 0 | 1 | 0 | 0 | 0 |
|  |  | (TTATA)3 | 1 | 0 | 0 | 1 | 1 | 0 | 0 | 0 |
|  |  | (TTTTA)3 | 1 | 1 | 0 | 0 | 1 | 0 | 0 | 0 |
|  |  | Subtotal | 9 | 6 | 0 | 3 | 5 | 0 | 2 | 2 |
|  | Hexanucleotide | (AAGAAT)3 | 1 | 1 | 0 | 0 | 1 | 0 | 0 | 0 |
|  |  | (CCTCTT)7 | 1 | 0 | 1 | 0 | 0 | 0 | 1 | 0 |
|  |  | (CTTCTA)3 | 1 | 0 | 1 | 0 | 0 | 0 | 1 | 0 |
|  |  | (GGAAGA)7 | 1 | 0 | 1 | 0 | 0 | 0 | 0 | 1 |
|  |  | (TAAATC)3 | 1 | 1 | 0 | 0 | 1 | 0 | 0 | 0 |
|  |  | (TAGAAG)3 | 1 | 0 | 1 | 0 | 0 | 0 | 0 | 1 |
|  |  | Subtotal | 6 | 2 | 4 | 0 | 2 | 0 | 2 | 2 |
|  | Subtotal |  | 324 | 176 | 87 | 61 | 226 | 8 | 45 | 45 |
|  | Total |  | 2799 | 1573 | 779 | 447 | 1936 | 151 | 356 | 356 |

**Table S4.** Number of SSRs (percentage in the total) in different categories of the cp genomes.

|  | Mononucleotide | Dinucleotide | Trinucleotide | Tetranucleotide | Pentanucleotide | Hexanucleotide | Total |
| --- | --- | --- | --- | --- | --- | --- | --- |
| *P. tranlienianum* | 172 (56.77%) | 83 (27.39%) | 19 (6.27%) | 17 (5.61%) | 5 (1.65%) | 7 (2.31%) | 303 |
| 1. *armeniacum* | 186 (57.41%) | 93 (28.70%) | 15 (4.63%) | 20 (6.17%) | 5 (1.54%) | 5 (1.54%) | 324 |
| *P. niveum* | 170 (54.49%) | 89 (28.53%) | 19 (6.09%) | 20 (6.41%) | 9 (2.88%) | 5 (1.60%) | 312 |
| *P. hirsutissimum* | 167 (60.51%) | 64 (23.19%) | 15 (5.43%) | 19 (6.88%) | 7 (2.54%) | 4 (1.45%) | 276 |
| 1. *violascens* | 165 (51.4%) | 100 (31.15%) | 21 (6.54%) | 20 (6.23%) | 9 (2.80%) | 6 (1.87%) | 321 |
| *P. dianthum* | 152 (55.32%) | 88 (31.21%) | 16 (5.67%) | 18 (6.38%) | 3 (1.06%) | 5 (1.77%) | 282 |
| *P. victoria-mariae* | 171 (52.62%) | 107 (32.92%) | 21 (6.46%) | 17 (5.23%) | 5 (1.54%) | 4 (1.23%) | 325 |
| *P. kolopakingii* | 170 (51.20%) | 106 (31.93%) | 21 (6.33%) | 20 (6.02%) | 10 (3.01%) | 5 (1.51%) | 332 |
| *P. philippinense* | 173 (53.40%) | 99 (30.56%) | 17 (5.25%) | 20 (6.17%) | 9 (2.78%) | 6 (1.85%) | 324 |
| Average | 169.5 | 92.1 | 18.2 | 19 | 6.9 | 5.2 | 311 |
| Min.-Max. | 152-186 | 64-107 | 15-21 | 17-20 | 5-10 | 4-7 | 276-332 |
| Total | 1526 (54.52%) | 829 (29.62%) | 164 (5.86%) | 171 (6.11%) | 62 (2.22%) | 47 (1.68%) | 2799 |

**Table S5.** SSRs location in the 9 *Paphiopedilum* chloroplast genomes.

| **Taxa** | **SSR type** | **SSR** | **Size** | **Start** | **End** | **Locus** |
| --- | --- | --- | --- | --- | --- | --- |
| Paphiopedilum_emersonii | p1 | (T)8 | 8 | 1764 | 1771 | trnK-UUU intron |
| Paphiopedilum_emersonii | p1 | (T)9 | 9 | 1773 | 1781 | trnK-UUU intron |
| Paphiopedilum_emersonii | p1 | (T)8 | 8 | 2967 | 2974 | trnK-UUU intron |
| Paphiopedilum_emersonii | p1 | (A)8 | 8 | 3075 | 3082 | trnK-UUU intron |
| Paphiopedilum_emersonii | p1 | (T)9 | 9 | 3578 | 3586 | trnK-UUU intron |
| Paphiopedilum_emersonii | p1 | (T)8 | 8 | 4011 | 4018 | trnK-UUU intron |
| Paphiopedilum_emersonii | p1 | (A)9 | 9 | 4085 | 4093 | trnK-UUU intron |
| Paphiopedilum_emersonii | p1 | (A)12 | 12 | 4757 | 4768 | matK_rps16 |
| Paphiopedilum_emersonii | p1 | (A)11 | 11 | 5076 | 5086 | matK_rps16 |
| Paphiopedilum_emersonii | p1 | (C)9 | 9 | 5159 | 5167 | matK_rps16 |
| Paphiopedilum_emersonii | p1 | (G)9 | 9 | 5172 | 5180 | matK_rps16 |
| Paphiopedilum_emersonii | p1 | (A)8 | 8 | 5768 | 5775 | rps16 intron |
| Paphiopedilum_emersonii | p1 | (T)10 | 10 | 5887 | 5896 | rps16 intron |
| Paphiopedilum_emersonii | p1 | (T)8 | 8 | 6045 | 6052 | rps16 intron |
| Paphiopedilum_emersonii | p1 | (T)11 | 11 | 6142 | 6152 | rps16 intron |
| Paphiopedilum_emersonii | p1 | (A)9 | 9 | 6695 | 6703 | rps16_trnQ-UUG |
| Paphiopedilum_emersonii | p1 | (T)8 | 8 | 7243 | 7250 | trnQ-UUG_psbK |
| Paphiopedilum_emersonii | p1 | (A)12 | 12 | 7876 | 7887 | psbK_psbI |
| Paphiopedilum_emersonii | p1 | (A)12 | 12 | 8058 | 8069 | psbK_psbI |
| Paphiopedilum_emersonii | p1 | (A)10 | 10 | 8558 | 8567 | trnS-GCU_trnG-UCC |
| Paphiopedilum_emersonii | p1 | (A)8 | 8 | 9880 | 9887 | trnS-GCU_trnG-UCC |
| Paphiopedilum_emersonii | p1 | (T)8 | 8 | 10522 | 10529 | trnG-UCC_trnR-UCU |
| Paphiopedilum_emersonii | p1 | (A)9 | 9 | 12398 | 12406 | atpA_atpF |
| Paphiopedilum_emersonii | p1 | (T)10 | 10 | 13165 | 13174 | atpF intron |
| Paphiopedilum_emersonii | p1 | (T)16 | 16 | 13342 | 13357 | atpF intron |
| Paphiopedilum_emersonii | p1 | (A)8 | 8 | 13688 | 13695 | atpF |
| Paphiopedilum_emersonii | p1 | (T)8 | 8 | 13891 | 13898 | atpF_atpH |
| Paphiopedilum_emersonii | p1 | (A)12 | 12 | 14181 | 14192 | atpH_atpI |
| Paphiopedilum_emersonii | p1 | (T)10 | 10 | 14299 | 14308 | atpH_atpI |
| Paphiopedilum_emersonii | p1 | (A)10 | 10 | 15655 | 15664 | atpI_rps2 |
| Paphiopedilum_emersonii | p1 | (T)15 | 15 | 16534 | 16548 | rps2_rpoC2 |
| Paphiopedilum_emersonii | p1 | (A)9 | 9 | 16795 | 16803 | rps2_rpoC2 |
| Paphiopedilum_emersonii | p1 | (T)9 | 9 | 18575 | 18583 | rpoC2 |
| Paphiopedilum_emersonii | p1 | (T)8 | 8 | 18750 | 18757 | rpoC2 |
| Paphiopedilum_emersonii | p1 | (A)8 | 8 | 18896 | 18903 | rpoC2 |
| Paphiopedilum_emersonii | p1 | (A)8 | 8 | 22579 | 22586 | rpoC1 |
| Paphiopedilum_emersonii | p1 | (C)9 | 9 | 22713 | 22721 | rpoC1 intron |
| Paphiopedilum_emersonii | p1 | (T)8 | 8 | 22890 | 22897 | rpoC1 intron |
| Paphiopedilum_emersonii | p1 | (T)8 | 8 | 26508 | 26515 | rpoB |
| Paphiopedilum_emersonii | p1 | (T)11 | 11 | 28548 | 28558 | trnC-GCA_petN |
| Paphiopedilum_emersonii | p1 | (T)8 | 8 | 28609 | 28616 | trnC-GCA_petN |
| Paphiopedilum_emersonii | p1 | (A)9 | 9 | 29063 | 29071 | petN_psbM |
| Paphiopedilum_emersonii | p1 | (T)8 | 8 | 29320 | 29327 | petN_psbM |
| Paphiopedilum_emersonii | p1 | (A)8 | 8 | 30625 | 30632 | psbM_trnD-GUC |
| Paphiopedilum_emersonii | p1 | (T)8 | 8 | 31360 | 31367 | trnD-GUC_trnY-GUA |
| Paphiopedilum_emersonii | p1 | (T)8 | 8 | 31908 | 31915 | trnE-UUC_trnT-GGU |
| Paphiopedilum_emersonii | p1 | (A)13 | 13 | 32029 | 32041 | trnE-UUC_trnT-GGU |
| Paphiopedilum_emersonii | p1 | (T)9 | 9 | 32205 | 32213 | trnE-UUC_trnT-GGU |
| Paphiopedilum_emersonii | p1 | (A)14 | 14 | 32495 | 32508 | trnT-GGU_psbD |
| Paphiopedilum_emersonii | p1 | (T)12 | 12 | 37693 | 37704 | rps14_psaB |
| Paphiopedilum_emersonii | p1 | (A)8 | 8 | 42875 | 42882 | psaA_ycf3 |
| Paphiopedilum_emersonii | p1 | (A)12 | 12 | 46428 | 46439 | rps4_trnT-UGU |
| Paphiopedilum_emersonii | p1 | (T)16 | 16 | 46904 | 46919 | trnT-UGU_trnL-UAA |
| Paphiopedilum_emersonii | p1 | (A)12 | 12 | 47421 | 47432 | trnT-UGU_trnL-UAA |
| Paphiopedilum_emersonii | p1 | (T)8 | 8 | 48445 | 48452 | trnL-UAA_trnF-GAA |
| Paphiopedilum_emersonii | p1 | (T)12 | 12 | 49139 | 49150 | trnF-GAA_ndhJ |
| Paphiopedilum_emersonii | p1 | (T)8 | 8 | 49155 | 49162 | trnF-GAA_ndhJ |
| Paphiopedilum_emersonii | p1 | (T)8 | 8 | 49689 | 49696 | ndhJ |
| Paphiopedilum_emersonii | p1 | (T)8 | 8 | 53987 | 53994 | atpB_rbcL |
| Paphiopedilum_emersonii | p1 | (T)10 | 10 | 54370 | 54379 | atpB_rbcL |
| Paphiopedilum_emersonii | p1 | (T)12 | 12 | 55080 | 55091 | atpB_rbcL |
| Paphiopedilum_emersonii | p1 | (T)8 | 8 | 56684 | 56691 | rbcL_accD |
| Paphiopedilum_emersonii | p1 | (T)8 | 8 | 57158 | 57165 | rbcL_accD |
| Paphiopedilum_emersonii | p1 | (A)12 | 12 | 57323 | 57334 | rbcL_accD |
| Paphiopedilum_emersonii | p1 | (T)10 | 10 | 58127 | 58136 | accD |
| Paphiopedilum_emersonii | p1 | (A)10 | 10 | 58313 | 58322 | accD |
| Paphiopedilum_emersonii | p1 | (A)10 | 10 | 58878 | 58887 | accD |
| Paphiopedilum_emersonii | p1 | (T)11 | 11 | 59694 | 59704 | psaI_ycf4 |
| Paphiopedilum_emersonii | p1 | (A)8 | 8 | 60611 | 60618 | ycf4_cemA |
| Paphiopedilum_emersonii | p1 | (T)11 | 11 | 61149 | 61159 | ycf4_cemA |
| Paphiopedilum_emersonii | p1 | (A)10 | 10 | 61368 | 61377 | cemA |
| Paphiopedilum_emersonii | p1 | (T)8 | 8 | 62148 | 62155 | cemA_petA |
| Paphiopedilum_emersonii | p1 | (A)8 | 8 | 62736 | 62743 | petA |
| Paphiopedilum_emersonii | p1 | (T)9 | 9 | 63345 | 63353 | petA_psbJ |
| Paphiopedilum_emersonii | p1 | (T)9 | 9 | 63642 | 63650 | petA_psbJ |
| Paphiopedilum_emersonii | p1 | (C)13 | 13 | 64434 | 64446 | psbJ_psbL |
| Paphiopedilum_emersonii | p1 | (G)11 | 11 | 65132 | 65142 | psbE_petL |
| Paphiopedilum_emersonii | p1 | (T)12 | 12 | 65475 | 65486 | psbE_petL |
| Paphiopedilum_emersonii | p1 | (A)10 | 10 | 65614 | 65623 | psbE_petL |
| Paphiopedilum_emersonii | p1 | (T)8 | 8 | 65807 | 65814 | psbE_petL |
| Paphiopedilum_emersonii | p1 | (A)22 | 22 | 66084 | 66105 | petL_petG |
| Paphiopedilum_emersonii | p1 | (T)18 | 18 | 66953 | 66970 | trnP-UGG_psaJ |
| Paphiopedilum_emersonii | p1 | (T)9 | 9 | 67332 | 67340 | trnP-UGG_psaJ |
| Paphiopedilum_emersonii | p1 | (A)8 | 8 | 67925 | 67932 | psaJ_rpl33 |
| Paphiopedilum_emersonii | p1 | (A)8 | 8 | 68610 | 68617 | rpl33_rps18 |
| Paphiopedilum_emersonii | p1 | (A)9 | 9 | 69139 | 69147 | rps18 |
| Paphiopedilum_emersonii | p1 | (T)10 | 10 | 69274 | 69283 | rps18_rpl20 |
| Paphiopedilum_emersonii | p1 | (T)9 | 9 | 69741 | 69749 | rpl20 |
| Paphiopedilum_emersonii | p1 | (T)9 | 9 | 70144 | 70152 | rpl20_rps12 |
| Paphiopedilum_emersonii | p1 | (T)9 | 9 | 70265 | 70273 | rpl20_rps12 |
| Paphiopedilum_emersonii | p1 | (T)9 | 9 | 71548 | 71556 | clpP intron 1 |
| Paphiopedilum_emersonii | p1 | (T)11 | 11 | 71777 | 71787 | clpP intron 1 |
| Paphiopedilum_emersonii | p1 | (T)9 | 9 | 71829 | 71837 | clpP intron 1 |
| Paphiopedilum_emersonii | p1 | (T)8 | 8 | 72252 | 72259 | clpP intron 2 |
| Paphiopedilum_emersonii | p1 | (T)16 | 16 | 72307 | 72322 | clpP intron 2 |
| Paphiopedilum_emersonii | p1 | (A)17 | 17 | 72377 | 72393 | clpP intron 2 |
| Paphiopedilum_emersonii | p1 | (T)14 | 14 | 72580 | 72593 | clpP intron 2 |
| Paphiopedilum_emersonii | p1 | (T)8 | 8 | 74933 | 74940 | psbB |
| Paphiopedilum_emersonii | p1 | (T)8 | 8 | 75583 | 75590 | psbB |
| Paphiopedilum_emersonii | p1 | (T)11 | 11 | 75744 | 75754 | psbB_psbT |
| Paphiopedilum_emersonii | p1 | (T)9 | 9 | 76062 | 76070 | psbB_psbT |
| Paphiopedilum_emersonii | p1 | (A)14 | 14 | 77409 | 77422 | petB intron |
| Paphiopedilum_emersonii | p1 | (T)8 | 8 | 78809 | 78816 | petD intron |
| Paphiopedilum_emersonii | p1 | (A)9 | 9 | 80100 | 80108 | petD_rpoA |
| Paphiopedilum_emersonii | p1 | (A)11 | 11 | 81683 | 81693 | rps11_rpl36 |
| Paphiopedilum_emersonii | p1 | (T)10 | 10 | 81998 | 82007 | rpl36_infA |
| Paphiopedilum_emersonii | p1 | (T)9 | 9 | 82636 | 82644 | rps8 |
| Paphiopedilum_emersonii | p1 | (T)8 | 8 | 82876 | 82883 | rps8_rpl14 |
| Paphiopedilum_emersonii | p1 | (T)17 | 17 | 83636 | 83652 | rpl14_rpl16 |
| Paphiopedilum_emersonii | p1 | (T)8 | 8 | 84901 | 84908 | rpl16 intron |
| Paphiopedilum_emersonii | p1 | (T)10 | 10 | 85004 | 85013 | rpl16 intron |
| Paphiopedilum_emersonii | p1 | (T)8 | 8 | 85191 | 85198 | rpl16 intron |
| Paphiopedilum_emersonii | p1 | (T)8 | 8 | 86172 | 86179 | rpl22 |
| Paphiopedilum_emersonii | p1 | (T)9 | 9 | 87012 | 87020 | rps19 |
| Paphiopedilum_emersonii | p1 | (T)8 | 8 | 87048 | 87055 | rps19_trnH-GUG |
| Paphiopedilum_emersonii | p1 | (A)8 | 8 | 91323 | 91330 | ycf2 |
| Paphiopedilum_emersonii | p1 | (A)9 | 9 | 92549 | 92557 | ycf2 |
| Paphiopedilum_emersonii | p1 | (T)8 | 8 | 101261 | 101268 | rps12_ycf15 |
| Paphiopedilum_emersonii | p1 | (T)9 | 9 | 101292 | 101300 | rps12_ycf15 |
| Paphiopedilum_emersonii | p1 | (T)10 | 10 | 102480 | 102489 | ycf15_trnV-GAC |
| Paphiopedilum_emersonii | p1 | (G)10 | 10 | 105175 | 105184 | trnI-GAU intron |
| Paphiopedilum_emersonii | p1 | (T)9 | 9 | 105377 | 105385 | trnI-GAU intron |
| Paphiopedilum_emersonii | p1 | (A)10 | 10 | 112380 | 112389 | ycf1 |
| Paphiopedilum_emersonii | p1 | (T)9 | 9 | 112427 | 112435 | ycf1 |
| Paphiopedilum_emersonii | p1 | (T)10 | 10 | 112518 | 112527 | ycf1 |
| Paphiopedilum_emersonii | p1 | (A)10 | 10 | 112681 | 112690 | ycf1 |
| Paphiopedilum_emersonii | p1 | (A)9 | 9 | 112803 | 112811 | ycf1 |
| Paphiopedilum_emersonii | p1 | (A)8 | 8 | 113006 | 113013 | ycf1 |
| Paphiopedilum_emersonii | p1 | (T)8 | 8 | 113720 | 113727 | ycf1 |
| Paphiopedilum_emersonii | p1 | (A)11 | 11 | 114709 | 114719 | ycf1 |
| Paphiopedilum_emersonii | p1 | (A)9 | 9 | 114902 | 114910 | ycf1 |
| Paphiopedilum_emersonii | p1 | (A)8 | 8 | 115330 | 115337 | ycf1 |
| Paphiopedilum_emersonii | p1 | (A)8 | 8 | 115461 | 115468 | ycf1 |
| Paphiopedilum_emersonii | p1 | (A)8 | 8 | 115512 | 115519 | ycf1 |
| Paphiopedilum_emersonii | p1 | (A)8 | 8 | 115850 | 115857 | ycf1 |
| Paphiopedilum_emersonii | p1 | (A)9 | 9 | 115860 | 115868 | ycf1 |
| Paphiopedilum_emersonii | p1 | (A)11 | 11 | 116300 | 116310 | ycf1 |
| Paphiopedilum_emersonii | p1 | (A)8 | 8 | 116678 | 116685 | ycf1 |
| Paphiopedilum_emersonii | p1 | (T)8 | 8 | 117132 | 117139 | ycf1_rps15 |
| Paphiopedilum_emersonii | p1 | (T)16 | 16 | 117789 | 117804 | rps15_psaC |
| Paphiopedilum_emersonii | p1 | (A)10 | 10 | 120539 | 120548 | psaC_trnL-UAG |
| Paphiopedilum_emersonii | p1 | (A)9 | 9 | 121638 | 121646 | rpl32 |
| Paphiopedilum_emersonii | p1 | (T)9 | 9 | 121821 | 121829 | rpl32_ccsA |
| Paphiopedilum_emersonii | p1 | (T)10 | 10 | 122407 | 122416 | ccsA |
| Paphiopedilum_emersonii | p1 | (A)16 | 16 | 125151 | 125166 | psaC_rps15 |
| Paphiopedilum_emersonii | p1 | (A)8 | 8 | 125816 | 125823 | rps15_ycf1 |
| Paphiopedilum_emersonii | p1 | (T)8 | 8 | 126270 | 126277 | ycf1 |
| Paphiopedilum_emersonii | p1 | (T)11 | 11 | 126635 | 126645 | ycf1 |
| Paphiopedilum_emersonii | p1 | (T)9 | 9 | 127087 | 127095 | ycf1 |
| Paphiopedilum_emersonii | p1 | (T)8 | 8 | 127098 | 127105 | ycf1 |
| Paphiopedilum_emersonii | p1 | (T)8 | 8 | 127436 | 127443 | ycf1 |
| Paphiopedilum_emersonii | p1 | (T)8 | 8 | 127487 | 127494 | ycf1 |
| Paphiopedilum_emersonii | p1 | (T)8 | 8 | 127618 | 127625 | ycf1 |
| Paphiopedilum_emersonii | p1 | (T)9 | 9 | 128045 | 128053 | ycf1 |
| Paphiopedilum_emersonii | p1 | (T)11 | 11 | 128236 | 128246 | ycf1 |
| Paphiopedilum_emersonii | p1 | (A)8 | 8 | 129228 | 129235 | ycf1 |
| Paphiopedilum_emersonii | p1 | (T)8 | 8 | 129942 | 129949 | ycf1 |
| Paphiopedilum_emersonii | p1 | (T)9 | 9 | 130144 | 130152 | ycf1 |
| Paphiopedilum_emersonii | p1 | (T)10 | 10 | 130265 | 130274 | ycf1 |
| Paphiopedilum_emersonii | p1 | (A)10 | 10 | 130428 | 130437 | ycf1 |
| Paphiopedilum_emersonii | p1 | (A)9 | 9 | 130520 | 130528 | ycf1 |
| Paphiopedilum_emersonii | p1 | (T)10 | 10 | 130566 | 130575 | ycf1 |
| Paphiopedilum_emersonii | p1 | (A)9 | 9 | 137570 | 137578 | trnI-GAU intron |
| Paphiopedilum_emersonii | p1 | (C)10 | 10 | 137771 | 137780 | trnI-GAU intron |
| Paphiopedilum_emersonii | p1 | (A)10 | 10 | 140466 | 140475 | trnV-GAC_ycf15 |
| Paphiopedilum_emersonii | p1 | (A)9 | 9 | 141655 | 141663 | ycf15_rps12 |
| Paphiopedilum_emersonii | p1 | (A)8 | 8 | 141687 | 141694 | ycf15_rps12 |
| Paphiopedilum_emersonii | p1 | (T)9 | 9 | 150398 | 150406 | ycf2 |
| Paphiopedilum_emersonii | p1 | (T)8 | 8 | 151625 | 151632 | ycf2 |
| Paphiopedilum_emersonii | p1 | (A)8 | 8 | 155900 | 155907 | trnH-GUG_rps19 |
| Paphiopedilum_emersonii | p1 | (A)9 | 9 | 155935 | 155943 | rps19 |
| Paphiopedilum_victoria-mariae | p1 | (T)9 | 9 | 1755 | 1763 | trnK-UUU intron |
| Paphiopedilum_victoria-mariae | p1 | (T)9 | 9 | 1765 | 1773 | trnK-UUU intron |
| Paphiopedilum_victoria-mariae | p1 | (T)9 | 9 | 2959 | 2967 | trnK-UUU intron |
| Paphiopedilum_victoria-mariae | p1 | (A)8 | 8 | 3067 | 3074 | trnK-UUU intron |
| Paphiopedilum_victoria-mariae | p1 | (T)10 | 10 | 3588 | 3597 | trnK-UUU intron |
| Paphiopedilum_victoria-mariae | p1 | (T)8 | 8 | 4146 | 4153 | trnK-UUU intron |
| Paphiopedilum_victoria-mariae | p1 | (A)9 | 9 | 4220 | 4228 | trnK-UUU intron |
| Paphiopedilum_victoria-mariae | p1 | (A)9 | 9 | 4689 | 4697 | matK_rps16 |
| Paphiopedilum_victoria-mariae | p1 | (A)10 | 10 | 5033 | 5042 | matK_rps16 |
| Paphiopedilum_victoria-mariae | p1 | (C)8 | 8 | 5324 | 5331 | matK_rps16 |
| Paphiopedilum_victoria-mariae | p1 | (G)8 | 8 | 5336 | 5343 | matK_rps16 |
| Paphiopedilum_victoria-mariae | p1 | (A)9 | 9 | 5890 | 5898 | rps16 intron |
| Paphiopedilum_victoria-mariae | p1 | (T)12 | 12 | 6010 | 6021 | rps16 intron |
| Paphiopedilum_victoria-mariae | p1 | (T)12 | 12 | 6270 | 6281 | rps16 intron |
| Paphiopedilum_victoria-mariae | p1 | (A)10 | 10 | 6824 | 6833 | rps16_trnQ-UUG |
| Paphiopedilum_victoria-mariae | p1 | (T)9 | 9 | 7262 | 7270 | trnQ-UUG_psbK |
| Paphiopedilum_victoria-mariae | p1 | (T)8 | 8 | 7518 | 7525 | trnQ-UUG_psbK |
| Paphiopedilum_victoria-mariae | p1 | (A)12 | 12 | 8766 | 8777 | trnS-GCU_trnG-UCC |
| Paphiopedilum_victoria-mariae | p1 | (A)8 | 8 | 10276 | 10283 | trnG-UCC intron |
| Paphiopedilum_victoria-mariae | p1 | (A)8 | 8 | 10934 | 10941 | trnG-UCC_trnR-UCU |
| Paphiopedilum_victoria-mariae | p1 | (A)10 | 10 | 12887 | 12896 | atpA_atpF |
| Paphiopedilum_victoria-mariae | p1 | (A)8 | 8 | 13088 | 13095 | atpF |
| Paphiopedilum_victoria-mariae | p1 | (T)10 | 10 | 13646 | 13655 | atpF intron |
| Paphiopedilum_victoria-mariae | p1 | (T)11 | 11 | 13826 | 13836 | atpF intron |
| Paphiopedilum_victoria-mariae | p1 | (T)10 | 10 | 13841 | 13850 | atpF intron |
| Paphiopedilum_victoria-mariae | p1 | (A)8 | 8 | 14100 | 14107 | atpF intron |
| Paphiopedilum_victoria-mariae | p1 | (A)8 | 8 | 14189 | 14196 | atpF |
| Paphiopedilum_victoria-mariae | p1 | (T)8 | 8 | 14392 | 14399 | atpF_atpH |
| Paphiopedilum_victoria-mariae | p1 | (T)8 | 8 | 14617 | 14624 | atpF_atpH |
| Paphiopedilum_victoria-mariae | p1 | (A)13 | 13 | 14915 | 14927 | atpH_atpI |
| Paphiopedilum_victoria-mariae | p1 | (T)9 | 9 | 15034 | 15042 | atpH_atpI |
| Paphiopedilum_victoria-mariae | p1 | (A)9 | 9 | 16495 | 16503 | atpI_rps2 |
| Paphiopedilum_victoria-mariae | p1 | (T)11 | 11 | 17373 | 17383 | rps2 |
| Paphiopedilum_victoria-mariae | p1 | (A)8 | 8 | 17630 | 17637 | rps2_rpoC2 |
| Paphiopedilum_victoria-mariae | p1 | (T)9 | 9 | 19409 | 19417 | rpoC2 |
| Paphiopedilum_victoria-mariae | p1 | (T)8 | 8 | 19584 | 19591 | rpoC2 |
| Paphiopedilum_victoria-mariae | p1 | (A)8 | 8 | 19730 | 19737 | rpoC2 |
| Paphiopedilum_victoria-mariae | p1 | (A)8 | 8 | 23413 | 23420 | rpoC1 |
| Paphiopedilum_victoria-mariae | p1 | (C)8 | 8 | 23547 | 23554 | rpoC1 intron |
| Paphiopedilum_victoria-mariae | p1 | (T)8 | 8 | 23723 | 23730 | rpoC1 intron |
| Paphiopedilum_victoria-mariae | p1 | (T)8 | 8 | 27341 | 27348 | rpoB |
| Paphiopedilum_victoria-mariae | p1 | (T)12 | 12 | 29441 | 29452 | trnC-GCA_petN |
| Paphiopedilum_victoria-mariae | p1 | (T)9 | 9 | 29572 | 29580 | trnC-GCA_petN |
| Paphiopedilum_victoria-mariae | p1 | (A)19 | 19 | 30010 | 30028 | petN_psbM |
| Paphiopedilum_victoria-mariae | p1 | (T)10 | 10 | 31272 | 31281 | petN_psbM |
| Paphiopedilum_victoria-mariae | p1 | (T)8 | 8 | 32636 | 32643 | trnD-GUC_trnY-GUA |
| Paphiopedilum_victoria-mariae | p1 | (T)8 | 8 | 33184 | 33191 | trnE-UUC_trnT-GGU |
| Paphiopedilum_victoria-mariae | p1 | (A)10 | 10 | 33280 | 33289 | trnE-UUC_trnT-GGU |
| Paphiopedilum_victoria-mariae | p1 | (A)8 | 8 | 34273 | 34280 | trnE-UUC_trnT-GGU |
| Paphiopedilum_victoria-mariae | p1 | (T)8 | 8 | 34444 | 34451 | trnE-UUC_trnT-GGU |
| Paphiopedilum_victoria-mariae | p1 | (A)13 | 13 | 34721 | 34733 | trnT-GGU_psbD |
| Paphiopedilum_victoria-mariae | p1 | (T)10 | 10 | 39969 | 39978 | rps14_psaB |
| Paphiopedilum_victoria-mariae | p1 | (G)8 | 8 | 39982 | 39989 | rps14_psaB |
| Paphiopedilum_victoria-mariae | p1 | (T)8 | 8 | 44899 | 44906 | psaA_ycf3 |
| Paphiopedilum_victoria-mariae | p1 | (A)8 | 8 | 45144 | 45151 | psaA_ycf3 |
| Paphiopedilum_victoria-mariae | p1 | (T)8 | 8 | 45958 | 45965 | ycf3 intron 1 |
| Paphiopedilum_victoria-mariae | p1 | (A)9 | 9 | 48698 | 48706 | rps4_trnT-UGU |
| Paphiopedilum_victoria-mariae | p1 | (T)12 | 12 | 49174 | 49185 | trnT-UGU_trnL-UAA |
| Paphiopedilum_victoria-mariae | p1 | (A)26 | 26 | 49862 | 49887 | trnT-UGU_trnL-UAA |
| Paphiopedilum_victoria-mariae | p1 | (T)11 | 11 | 50896 | 50906 | trnL-UAA_trnF-GAA |
| Paphiopedilum_victoria-mariae | p1 | (T)12 | 12 | 51642 | 51653 | trnF-GAA_ndhJ |
| Paphiopedilum_victoria-mariae | p1 | (T)8 | 8 | 51658 | 51665 | trnF-GAA_ndhJ |
| Paphiopedilum_victoria-mariae | p1 | (T)8 | 8 | 52334 | 52341 | ndhJ |
| Paphiopedilum_victoria-mariae | p1 | (T)9 | 9 | 56634 | 56642 | atpB_rbcL |
| Paphiopedilum_victoria-mariae | p1 | (T)16 | 16 | 56984 | 56999 | atpB_rbcL |
| Paphiopedilum_victoria-mariae | p1 | (T)9 | 9 | 57744 | 57752 | atpB_rbcL |
| Paphiopedilum_victoria-mariae | p1 | (T)9 | 9 | 59345 | 59353 | rbcL_accD |
| Paphiopedilum_victoria-mariae | p1 | (T)9 | 9 | 59827 | 59835 | rbcL_accD |
| Paphiopedilum_victoria-mariae | p1 | (A)10 | 10 | 59993 | 60002 | rbcL_accD |
| Paphiopedilum_victoria-mariae | p1 | (A)10 | 10 | 60981 | 60990 | accD |
| Paphiopedilum_victoria-mariae | p1 | (A)16 | 16 | 61546 | 61561 | accD |
| Paphiopedilum_victoria-mariae | p1 | (A)8 | 8 | 61874 | 61881 | accD_psaI |
| Paphiopedilum_victoria-mariae | p1 | (A)9 | 9 | 61950 | 61958 | accD_psaI |
| Paphiopedilum_victoria-mariae | p1 | (A)8 | 8 | 63713 | 63720 | ycf4_cemA |
| Paphiopedilum_victoria-mariae | p1 | (A)8 | 8 | 63769 | 63776 | ycf4_cemA |
| Paphiopedilum_victoria-mariae | p1 | (A)8 | 8 | 65310 | 65317 | petA |
| Paphiopedilum_victoria-mariae | p1 | (T)8 | 8 | 65919 | 65926 | petA_psbJ |
| Paphiopedilum_victoria-mariae | p1 | (T)11 | 11 | 66223 | 66233 | petA_psbJ |
| Paphiopedilum_victoria-mariae | p1 | (G)11 | 11 | 67693 | 67703 | psbE_petL |
| Paphiopedilum_victoria-mariae | p1 | (T)8 | 8 | 68035 | 68042 | psbE_petL |
| Paphiopedilum_victoria-mariae | p1 | (A)9 | 9 | 68172 | 68180 | psbE_petL |
| Paphiopedilum_victoria-mariae | p1 | (T)8 | 8 | 68364 | 68371 | psbE_petL |
| Paphiopedilum_victoria-mariae | p1 | (A)13 | 13 | 68649 | 68661 | petL_petG |
| Paphiopedilum_victoria-mariae | p1 | (T)11 | 11 | 69505 | 69515 | trnP-UGG_psaJ |
| Paphiopedilum_victoria-mariae | p1 | (A)8 | 8 | 70417 | 70424 | psaJ_rpl33 |
| Paphiopedilum_victoria-mariae | p1 | (A)8 | 8 | 71107 | 71114 | rpl33_rps18 |
| Paphiopedilum_victoria-mariae | p1 | (T)12 | 12 | 71805 | 71816 | rps18_rpl20 |
| Paphiopedilum_victoria-mariae | p1 | (T)8 | 8 | 72276 | 72283 | rpl20 |
| Paphiopedilum_victoria-mariae | p1 | (T)10 | 10 | 72678 | 72687 | rpl20_rps12 |
| Paphiopedilum_victoria-mariae | p1 | (T)10 | 10 | 72799 | 72808 | rpl20_rps12 |
| Paphiopedilum_victoria-mariae | p1 | (T)10 | 10 | 73807 | 73816 | clpP intron 1 |
| Paphiopedilum_victoria-mariae | p1 | (T)9 | 9 | 74133 | 74141 | clpP intron 1 |
| Paphiopedilum_victoria-mariae | p1 | (T)14 | 14 | 74362 | 74375 | clpP intron 1 |
| Paphiopedilum_victoria-mariae | p1 | (T)9 | 9 | 74414 | 74422 | clpP intron 1 |
| Paphiopedilum_victoria-mariae | p1 | (T)8 | 8 | 74837 | 74844 | clpP intron 2 |
| Paphiopedilum_victoria-mariae | p1 | (T)10 | 10 | 74884 | 74893 | clpP intron 2 |
| Paphiopedilum_victoria-mariae | p1 | (A)17 | 17 | 74954 | 74970 | clpP intron 2 |
| Paphiopedilum_victoria-mariae | p1 | (T)11 | 11 | 75157 | 75167 | clpP intron 2 |
| Paphiopedilum_victoria-mariae | p1 | (T)8 | 8 | 77505 | 77512 | psbB |
| Paphiopedilum_victoria-mariae | p1 | (T)8 | 8 | 78155 | 78162 | psbB |
| Paphiopedilum_victoria-mariae | p1 | (T)11 | 11 | 78339 | 78349 | psbB_psbT |
| Paphiopedilum_victoria-mariae | p1 | (T)8 | 8 | 78665 | 78672 | psbB_psbT |
| Paphiopedilum_victoria-mariae | p1 | (A)15 | 15 | 79995 | 80009 | petB intron |
| Paphiopedilum_victoria-mariae | p1 | (T)8 | 8 | 81403 | 81410 | petD intron |
| Paphiopedilum_victoria-mariae | p1 | (A)9 | 9 | 84255 | 84263 | rps11_rpl36 |
| Paphiopedilum_victoria-mariae | p1 | (T)8 | 8 | 84357 | 84364 | rps11_rpl36 |
| Paphiopedilum_victoria-mariae | p1 | (T)10 | 10 | 84569 | 84578 | rpl36_infA |
| Paphiopedilum_victoria-mariae | p1 | (T)9 | 9 | 85207 | 85215 | rps8 |
| Paphiopedilum_victoria-mariae | p1 | (T)23 | 23 | 86262 | 86284 | rpl14_rpl16 |
| Paphiopedilum_victoria-mariae | p1 | (A)8 | 8 | 87016 | 87023 | rpl16 intron |
| Paphiopedilum_victoria-mariae | p1 | (T)14 | 14 | 87913 | 87926 | rpl16 intron |
| Paphiopedilum_victoria-mariae | p1 | (T)8 | 8 | 88104 | 88111 | rpl16 intron |
| Paphiopedilum_victoria-mariae | p1 | (T)8 | 8 | 89076 | 89083 | rpl22 |
| Paphiopedilum_victoria-mariae | p1 | (T)9 | 9 | 89881 | 89889 | rps19 |
| Paphiopedilum_victoria-mariae | p1 | (T)8 | 8 | 89917 | 89924 | rps19_trnH-GUG |
| Paphiopedilum_victoria-mariae | p1 | (A)8 | 8 | 94144 | 94151 | ycf2 |
| Paphiopedilum_victoria-mariae | p1 | (A)9 | 9 | 95436 | 95444 | ycf2 |
| Paphiopedilum_victoria-mariae | p1 | (T)8 | 8 | 104586 | 104593 | rps12_trnV-GAC |
| Paphiopedilum_victoria-mariae | p1 | (T)9 | 9 | 104617 | 104625 | rps12_trnV-GAC |
| Paphiopedilum_victoria-mariae | p1 | (G)10 | 10 | 108423 | 108432 | trnI-GAU intron |
| Paphiopedilum_victoria-mariae | p1 | (T)9 | 9 | 108625 | 108633 | trnI-GAU intron |
| Paphiopedilum_victoria-mariae | p1 | (T)9 | 9 | 115912 | 115920 | ycf1 |
| Paphiopedilum_victoria-mariae | p1 | (T)10 | 10 | 116003 | 116012 | ycf1 |
| Paphiopedilum_victoria-mariae | p1 | (A)10 | 10 | 116166 | 116175 | ycf1 |
| Paphiopedilum_victoria-mariae | p1 | (A)9 | 9 | 116288 | 116296 | ycf1 |
| Paphiopedilum_victoria-mariae | p1 | (A)8 | 8 | 116491 | 116498 | ycf1 |
| Paphiopedilum_victoria-mariae | p1 | (T)8 | 8 | 117103 | 117110 | ycf1 |
| Paphiopedilum_victoria-mariae | p1 | (A)11 | 11 | 118092 | 118102 | ycf1 |
| Paphiopedilum_victoria-mariae | p1 | (A)9 | 9 | 118285 | 118293 | ycf1 |
| Paphiopedilum_victoria-mariae | p1 | (A)8 | 8 | 118713 | 118720 | ycf1 |
| Paphiopedilum_victoria-mariae | p1 | (A)8 | 8 | 118844 | 118851 | ycf1 |
| Paphiopedilum_victoria-mariae | p1 | (A)8 | 8 | 118895 | 118902 | ycf1 |
| Paphiopedilum_victoria-mariae | p1 | (A)8 | 8 | 119233 | 119240 | ycf1 |
| Paphiopedilum_victoria-mariae | p1 | (A)9 | 9 | 119243 | 119251 | ycf1 |
| Paphiopedilum_victoria-mariae | p1 | (A)13 | 13 | 119683 | 119695 | ycf1 |
| Paphiopedilum_victoria-mariae | p1 | (A)8 | 8 | 120061 | 120068 | ycf1 |
| Paphiopedilum_victoria-mariae | p1 | (T)9 | 9 | 120515 | 120523 | ycf1_rps15 |
| Paphiopedilum_victoria-mariae | p1 | (T)20 | 20 | 121268 | 121287 | rps15_psaC |
| Paphiopedilum_victoria-mariae | p1 | (T)9 | 9 | 121470 | 121478 | rps15_psaC |
| Paphiopedilum_victoria-mariae | p1 | (A)10 | 10 | 124197 | 124206 | ndhD_trnL-UAG |
| Paphiopedilum_victoria-mariae | p1 | (A)9 | 9 | 125659 | 125667 | rpl32 |
| Paphiopedilum_victoria-mariae | p1 | (T)10 | 10 | 125733 | 125742 | rpl32 |
| Paphiopedilum_victoria-mariae | p1 | (T)18 | 18 | 125863 | 125880 | rpl32_ccsA |
| Paphiopedilum_victoria-mariae | p1 | (T)10 | 10 | 126458 | 126467 | ccsA |
| Paphiopedilum_victoria-mariae | p1 | (A)9 | 9 | 129186 | 129194 | psaC_rps15 |
| Paphiopedilum_victoria-mariae | p1 | (A)20 | 20 | 129377 | 129396 | psaC_rps15 |
| Paphiopedilum_victoria-mariae | p1 | (A)9 | 9 | 130141 | 130149 | rps15_ycf1 |
| Paphiopedilum_victoria-mariae | p1 | (T)8 | 8 | 130596 | 130603 | ycf1 |
| Paphiopedilum_victoria-mariae | p1 | (T)13 | 13 | 130969 | 130981 | ycf1 |
| Paphiopedilum_victoria-mariae | p1 | (T)9 | 9 | 131413 | 131421 | ycf1 |
| Paphiopedilum_victoria-mariae | p1 | (T)8 | 8 | 131424 | 131431 | ycf1 |
| Paphiopedilum_victoria-mariae | p1 | (T)8 | 8 | 131762 | 131769 | ycf1 |
| Paphiopedilum_victoria-mariae | p1 | (T)8 | 8 | 131813 | 131820 | ycf1 |
| Paphiopedilum_victoria-mariae | p1 | (T)8 | 8 | 131944 | 131951 | ycf1 |
| Paphiopedilum_victoria-mariae | p1 | (T)9 | 9 | 132371 | 132379 | ycf1 |
| Paphiopedilum_victoria-mariae | p1 | (T)11 | 11 | 132562 | 132572 | ycf1 |
| Paphiopedilum_victoria-mariae | p1 | (A)8 | 8 | 133554 | 133561 | ycf1 |
| Paphiopedilum_victoria-mariae | p1 | (T)8 | 8 | 134166 | 134173 | ycf1 |
| Paphiopedilum_victoria-mariae | p1 | (T)9 | 9 | 134368 | 134376 | ycf1 |
| Paphiopedilum_victoria-mariae | p1 | (T)10 | 10 | 134489 | 134498 | ycf1 |
| Paphiopedilum_victoria-mariae | p1 | (A)10 | 10 | 134652 | 134661 | ycf1 |
| Paphiopedilum_victoria-mariae | p1 | (A)9 | 9 | 134744 | 134752 | ycf1 |
| Paphiopedilum_victoria-mariae | p1 | (A)9 | 9 | 142031 | 142039 | trnI-GAU intron |
| Paphiopedilum_victoria-mariae | p1 | (C)10 | 10 | 142232 | 142241 | trnI-GAU intron |
| Paphiopedilum_victoria-mariae | p1 | (A)9 | 9 | 146039 | 146047 | trnV-GAC_rps12 |
| Paphiopedilum_victoria-mariae | p1 | (A)8 | 8 | 146071 | 146078 | trnV-GAC_rps12 |
| Paphiopedilum_victoria-mariae | p1 | (T)9 | 9 | 155220 | 155228 | ycf2 |
| Paphiopedilum_victoria-mariae | p1 | (T)8 | 8 | 156513 | 156520 | ycf2 |
| Paphiopedilum_victoria-mariae | p1 | (A)8 | 8 | 160740 | 160747 | trnH-GUG_rps19 |
| Paphiopedilum_victoria-mariae | p1 | (A)9 | 9 | 160775 | 160783 | rps19 |
| Paphiopedilum_armeniacum | p1 | (T)10 | 10 | 1729 | 1738 | trnK-UUU intron |
| Paphiopedilum_armeniacum | p1 | (A)8 | 8 | 3032 | 3039 | trnK-UUU intron |
| Paphiopedilum_armeniacum | p1 | (A)8 | 8 | 4124 | 4131 | trnK-UUU intron |
| Paphiopedilum_armeniacum | p1 | (T)8 | 8 | 4395 | 4402 | trnK-UUU intron |
| Paphiopedilum_armeniacum | p1 | (A)10 | 10 | 4569 | 4578 | matK_rps16 |
| Paphiopedilum_armeniacum | p1 | (A)10 | 10 | 4923 | 4932 | matK_rps16 |
| Paphiopedilum_armeniacum | p1 | (A)8 | 8 | 4962 | 4969 | matK_rps16 |
| Paphiopedilum_armeniacum | p1 | (C)9 | 9 | 5341 | 5349 | matK_rps16 |
| Paphiopedilum_armeniacum | p1 | (G)9 | 9 | 5354 | 5362 | matK_rps16 |
| Paphiopedilum_armeniacum | p1 | (A)10 | 10 | 5375 | 5384 | matK_rps16 |
| Paphiopedilum_armeniacum | p1 | (A)8 | 8 | 5881 | 5888 | rps16 intron |
| Paphiopedilum_armeniacum | p1 | (T)10 | 10 | 6000 | 6009 | rps16 intron |
| Paphiopedilum_armeniacum | p1 | (A)11 | 11 | 6788 | 6798 | rps16_trnQ-UUG |
| Paphiopedilum_armeniacum | p1 | (A)9 | 9 | 6809 | 6817 | rps16_trnQ-UUG |
| Paphiopedilum_armeniacum | p1 | (A)8 | 8 | 6956 | 6963 | rps16_trnQ-UUG |
| Paphiopedilum_armeniacum | p1 | (T)16 | 16 | 7039 | 7054 | rps16_trnQ-UUG |
| Paphiopedilum_armeniacum | p1 | (A)8 | 8 | 7292 | 7299 | rps16_trnQ-UUG |
| Paphiopedilum_armeniacum | p1 | (T)12 | 12 | 7616 | 7627 | trnQ-UUG_psbK |
| Paphiopedilum_armeniacum | p1 | (T)8 | 8 | 7874 | 7881 | trnQ-UUG_psbK |
| Paphiopedilum_armeniacum | p1 | (A)8 | 8 | 9108 | 9115 | trnS-GCU_trnG-UCC |
| Paphiopedilum_armeniacum | p1 | (A)10 | 10 | 11204 | 11213 | trnG-UCC_trnR-UCU |
| Paphiopedilum_armeniacum | p1 | (A)9 | 9 | 13205 | 13213 | atpA_atpF |
| Paphiopedilum_armeniacum | p1 | (A)8 | 8 | 13405 | 13412 | atpF |
| Paphiopedilum_armeniacum | p1 | (A)11 | 11 | 13975 | 13985 | atpF intron |
| Paphiopedilum_armeniacum | p1 | (T)11 | 11 | 14148 | 14158 | atpF intron |
| Paphiopedilum_armeniacum | p1 | (A)9 | 9 | 14393 | 14401 | atpF intron |
| Paphiopedilum_armeniacum | p1 | (A)8 | 8 | 14483 | 14490 | atpF |
| Paphiopedilum_armeniacum | p1 | (A)13 | 13 | 14978 | 14990 | atpH_atpI |
| Paphiopedilum_armeniacum | p1 | (T)11 | 11 | 15113 | 15123 | atpH_atpI |
| Paphiopedilum_armeniacum | p1 | (T)11 | 11 | 15130 | 15140 | atpH_atpI |
| Paphiopedilum_armeniacum | p1 | (C)8 | 8 | 15539 | 15546 | atpH_atpI |
| Paphiopedilum_armeniacum | p1 | (A)9 | 9 | 16581 | 16589 | atpI_rps2 |
| Paphiopedilum_armeniacum | p1 | (T)8 | 8 | 17436 | 17443 | rps2_rpoC2 |
| Paphiopedilum_armeniacum | p1 | (A)8 | 8 | 17688 | 17695 | rps2_rpoC2 |
| Paphiopedilum_armeniacum | p1 | (T)9 | 9 | 19473 | 19481 | rpoC2 |
| Paphiopedilum_armeniacum | p1 | (T)9 | 9 | 19647 | 19655 | rpoC2 |
| Paphiopedilum_armeniacum | p1 | (A)8 | 8 | 19794 | 19801 | rpoC2 |
| Paphiopedilum_armeniacum | p1 | (A)8 | 8 | 23486 | 23493 | rpoC1 |
| Paphiopedilum_armeniacum | p1 | (C)12 | 12 | 23619 | 23630 | rpoC1 intron |
| Paphiopedilum_armeniacum | p1 | (T)14 | 14 | 23799 | 23812 | rpoC1 intron |
| Paphiopedilum_armeniacum | p1 | (A)11 | 11 | 24147 | 24157 | rpoC1 intron |
| Paphiopedilum_armeniacum | p1 | (T)8 | 8 | 27416 | 27423 | rpoB |
| Paphiopedilum_armeniacum | p1 | (A)9 | 9 | 29320 | 29328 | rpoB_trnC-GCA |
| Paphiopedilum_armeniacum | p1 | (A)8 | 8 | 29336 | 29343 | rpoB_trnC-GCA |
| Paphiopedilum_armeniacum | p1 | (T)11 | 11 | 29976 | 29986 | trnC-GCA_petN |
| Paphiopedilum_armeniacum | p1 | (A)10 | 10 | 30460 | 30469 | petN_psbM |
| Paphiopedilum_armeniacum | p1 | (T)10 | 10 | 34110 | 34119 | trnE-UUC_trnT-GGU |
| Paphiopedilum_armeniacum | p1 | (A)12 | 12 | 35296 | 35307 | trnT-GGU_psbD |
| Paphiopedilum_armeniacum | p1 | (T)8 | 8 | 40525 | 40532 | rps14_psaB |
| Paphiopedilum_armeniacum | p1 | (A)10 | 10 | 45494 | 45503 | psaA_ycf3 |
| Paphiopedilum_armeniacum | p1 | (A)11 | 11 | 46053 | 46063 | ycf3 intron 1 |
| Paphiopedilum_armeniacum | p1 | (A)10 | 10 | 48007 | 48016 | ycf3_trnS-GGA |
| Paphiopedilum_armeniacum | p1 | (T)8 | 8 | 48451 | 48458 | trnS-GGA_rps4 |
| Paphiopedilum_armeniacum | p1 | (A)10 | 10 | 49115 | 49124 | rps4_trnT-UGU |
| Paphiopedilum_armeniacum | p1 | (T)12 | 12 | 49598 | 49609 | trnT-UGU_trnL-UAA |
| Paphiopedilum_armeniacum | p1 | (A)10 | 10 | 50440 | 50449 | trnT-UGU_trnL-UAA |
| Paphiopedilum_armeniacum | p1 | (T)10 | 10 | 51259 | 51268 | trnL-UAA_trnF-GAA |
| Paphiopedilum_armeniacum | p1 | (A)8 | 8 | 51869 | 51876 | trnF-GAA_trnV-UAC |
| Paphiopedilum_armeniacum | p1 | (T)8 | 8 | 52003 | 52010 | trnF-GAA_trnV-UAC |
| Paphiopedilum_armeniacum | p1 | (T)8 | 8 | 53100 | 53107 | trnF-GAA_trnV-UAC |
| Paphiopedilum_armeniacum | p1 | (T)8 | 8 | 54202 | 54209 | trnF-GAA_trnV-UAC |
| Paphiopedilum_armeniacum | p1 | (T)9 | 9 | 58068 | 58076 | atpB_rbcL |
| Paphiopedilum_armeniacum | p1 | (T)8 | 8 | 58464 | 58471 | atpB_rbcL |
| Paphiopedilum_armeniacum | p1 | (T)8 | 8 | 58614 | 58621 | atpB_rbcL |
| Paphiopedilum_armeniacum | p1 | (T)8 | 8 | 59202 | 59209 | atpB_rbcL |
| Paphiopedilum_armeniacum | p1 | (A)8 | 8 | 61121 | 61128 | rbcL_accD |
| Paphiopedilum_armeniacum | p1 | (T)8 | 8 | 61247 | 61254 | rbcL_accD |
| Paphiopedilum_armeniacum | p1 | (A)9 | 9 | 62428 | 62436 | accD |
| Paphiopedilum_armeniacum | p1 | (A)8 | 8 | 62992 | 62999 | accD |
| Paphiopedilum_armeniacum | p1 | (A)9 | 9 | 63302 | 63310 | accD_psaI |
| Paphiopedilum_armeniacum | p1 | (A)10 | 10 | 65165 | 65174 | ycf4_cemA |
| Paphiopedilum_armeniacum | p1 | (T)9 | 9 | 65778 | 65786 | ycf4_cemA |
| Paphiopedilum_armeniacum | p1 | (A)8 | 8 | 65993 | 66000 | cemA |
| Paphiopedilum_armeniacum | p1 | (T)8 | 8 | 67962 | 67969 | petA_psbJ |
| Paphiopedilum_armeniacum | p1 | (T)11 | 11 | 68258 | 68268 | petA_psbJ |
| Paphiopedilum_armeniacum | p1 | (A)9 | 9 | 68742 | 68750 | petA_psbJ |
| Paphiopedilum_armeniacum | p1 | (G)11 | 11 | 69753 | 69763 | psbE_petL |
| Paphiopedilum_armeniacum | p1 | (T)10 | 10 | 70083 | 70092 | psbE_petL |
| Paphiopedilum_armeniacum | p1 | (A)11 | 11 | 70222 | 70232 | psbE_petL |
| Paphiopedilum_armeniacum | p1 | (T)8 | 8 | 70416 | 70423 | psbE_petL |
| Paphiopedilum_armeniacum | p1 | (A)12 | 12 | 70700 | 70711 | petL_petG |
| Paphiopedilum_armeniacum | p1 | (T)8 | 8 | 71051 | 71058 | petG_trnW-CCA |
| Paphiopedilum_armeniacum | p1 | (T)8 | 8 | 71557 | 71564 | trnP-UGG_psaJ |
| Paphiopedilum_armeniacum | p1 | (A)8 | 8 | 71703 | 71710 | trnP-UGG_psaJ |
| Paphiopedilum_armeniacum | p1 | (T)8 | 8 | 72274 | 72281 | psaJ |
| Paphiopedilum_armeniacum | p1 | (T)12 | 12 | 72754 | 72765 | psaJ_rpl33 |
| Paphiopedilum_armeniacum | p1 | (A)8 | 8 | 73145 | 73152 | rpl33_rps18 |
| Paphiopedilum_armeniacum | p1 | (T)8 | 8 | 74193 | 74200 | rpl20 |
| Paphiopedilum_armeniacum | p1 | (T)9 | 9 | 74592 | 74600 | rpl20_rps12 |
| Paphiopedilum_armeniacum | p1 | (T)9 | 9 | 74716 | 74724 | rpl20_rps12 |
| Paphiopedilum_armeniacum | p1 | (T)9 | 9 | 74996 | 75004 | rpl20_rps12 |
| Paphiopedilum_armeniacum | p1 | (T)8 | 8 | 75974 | 75981 | clpP intron 1 |
| Paphiopedilum_armeniacum | p1 | (T)15 | 15 | 76202 | 76216 | clpP intron 1 |
| Paphiopedilum_armeniacum | p1 | (T)12 | 12 | 76224 | 76235 | clpP intron 1 |
| Paphiopedilum_armeniacum | p1 | (T)9 | 9 | 76280 | 76288 | clpP intron 1 |
| Paphiopedilum_armeniacum | p1 | (T)18 | 18 | 76703 | 76720 | clpP intron 2 |
| Paphiopedilum_armeniacum | p1 | (A)21 | 21 | 76775 | 76795 | clpP intron 2 |
| Paphiopedilum_armeniacum | p1 | (T)9 | 9 | 76982 | 76990 | clpP intron 2 |
| Paphiopedilum_armeniacum | p1 | (T)11 | 11 | 78060 | 78070 | clpP_psbB |
| Paphiopedilum_armeniacum | p1 | (T)10 | 10 | 78089 | 78098 | clpP_psbB |
| Paphiopedilum_armeniacum | p1 | (T)8 | 8 | 79598 | 79605 | psbB |
| Paphiopedilum_armeniacum | p1 | (T)8 | 8 | 80248 | 80255 | psbB |
| Paphiopedilum_armeniacum | p1 | (T)16 | 16 | 80409 | 80424 | psbB_psbT |
| Paphiopedilum_armeniacum | p1 | (T)9 | 9 | 80444 | 80452 | psbB_psbT |
| Paphiopedilum_armeniacum | p1 | (T)8 | 8 | 80643 | 80650 | psbB_psbT |
| Paphiopedilum_armeniacum | p1 | (T)9 | 9 | 80932 | 80940 | psbB_psbT |
| Paphiopedilum_armeniacum | p1 | (A)9 | 9 | 82269 | 82277 | petB intron |
| Paphiopedilum_armeniacum | p1 | (A)8 | 8 | 86544 | 86551 | rps11_rpl36 |
| Paphiopedilum_armeniacum | p1 | (T)11 | 11 | 86859 | 86869 | rpl36_infA |
| Paphiopedilum_armeniacum | p1 | (T)8 | 8 | 87719 | 87726 | rps8_rpl14 |
| Paphiopedilum_armeniacum | p1 | (T)8 | 8 | 88605 | 88612 | rpl14_rpl16 |
| Paphiopedilum_armeniacum | p1 | (T)17 | 17 | 90136 | 90152 | rpl16 intron |
| Paphiopedilum_armeniacum | p1 | (A)9 | 9 | 90290 | 90298 | rpl16 intron |
| Paphiopedilum_armeniacum | p1 | (T)8 | 8 | 90664 | 90671 | rpl16 intron |
| Paphiopedilum_armeniacum | p1 | (A)8 | 8 | 90815 | 90822 | rpl16_rps3 |
| Paphiopedilum_armeniacum | p1 | (T)8 | 8 | 91672 | 91679 | rpl22 |
| Paphiopedilum_armeniacum | p1 | (T)9 | 9 | 92477 | 92485 | rps19 |
| Paphiopedilum_armeniacum | p1 | (T)8 | 8 | 92513 | 92520 | rps19_trnH-GUG |
| Paphiopedilum_armeniacum | p1 | (C)8 | 8 | 94084 | 94091 | rpl2 |
| Paphiopedilum_armeniacum | p1 | (A)8 | 8 | 96782 | 96789 | ycf2 |
| Paphiopedilum_armeniacum | p1 | (A)9 | 9 | 98074 | 98082 | ycf2 |
| Paphiopedilum_armeniacum | p1 | (T)8 | 8 | 107664 | 107671 | rps12_trnV-GAC |
| Paphiopedilum_armeniacum | p1 | (T)9 | 9 | 107695 | 107703 | rps12_trnV-GAC |
| Paphiopedilum_armeniacum | p1 | (T)9 | 9 | 108938 | 108946 | rps12_trnV-GAC |
| Paphiopedilum_armeniacum | p1 | (G)9 | 9 | 111632 | 111640 | trnI-GAU intron |
| Paphiopedilum_armeniacum | p1 | (A)10 | 10 | 118859 | 118868 | ycf1 |
| Paphiopedilum_armeniacum | p1 | (T)9 | 9 | 118906 | 118914 | ycf1 |
| Paphiopedilum_armeniacum | p1 | (T)12 | 12 | 118997 | 119008 | ycf1 |
| Paphiopedilum_armeniacum | p1 | (A)10 | 10 | 119160 | 119169 | ycf1 |
| Paphiopedilum_armeniacum | p1 | (A)9 | 9 | 119282 | 119290 | ycf1 |
| Paphiopedilum_armeniacum | p1 | (A)16 | 16 | 119649 | 119664 | ycf1 |
| Paphiopedilum_armeniacum | p1 | (T)8 | 8 | 120073 | 120080 | ycf1 |
| Paphiopedilum_armeniacum | p1 | (A)11 | 11 | 120774 | 120784 | ycf1 |
| Paphiopedilum_armeniacum | p1 | (A)9 | 9 | 120910 | 120918 | ycf1 |
| Paphiopedilum_armeniacum | p1 | (A)8 | 8 | 121059 | 121066 | ycf1 |
| Paphiopedilum_armeniacum | p1 | (A)9 | 9 | 121261 | 121269 | ycf1 |
| Paphiopedilum_armeniacum | p1 | (A)9 | 9 | 121691 | 121699 | ycf1 |
| Paphiopedilum_armeniacum | p1 | (A)8 | 8 | 121823 | 121830 | ycf1 |
| Paphiopedilum_armeniacum | p1 | (A)8 | 8 | 121874 | 121881 | ycf1 |
| Paphiopedilum_armeniacum | p1 | (A)8 | 8 | 122212 | 122219 | ycf1 |
| Paphiopedilum_armeniacum | p1 | (A)9 | 9 | 122222 | 122230 | ycf1 |
| Paphiopedilum_armeniacum | p1 | (A)9 | 9 | 122603 | 122611 | ycf1 |
| Paphiopedilum_armeniacum | p1 | (A)13 | 13 | 122662 | 122674 | ycf1 |
| Paphiopedilum_armeniacum | p1 | (A)8 | 8 | 123040 | 123047 | ycf1 |
| Paphiopedilum_armeniacum | p1 | (T)8 | 8 | 124249 | 124256 | rps15_psaC |
| Paphiopedilum_armeniacum | p1 | (T)9 | 9 | 125451 | 125459 | psaC_rpl32 |
| Paphiopedilum_armeniacum | p1 | (A)9 | 9 | 125690 | 125698 | rpl32 |
| Paphiopedilum_armeniacum | p1 | (A)8 | 8 | 125778 | 125785 | rpl32 |
| Paphiopedilum_armeniacum | p1 | (T)12 | 12 | 126077 | 126088 | rpl32_trnL-UAG |
| Paphiopedilum_armeniacum | p1 | (T)11 | 11 | 126363 | 126373 | rpl32_trnL-UAG |
| Paphiopedilum_armeniacum | p1 | (A)8 | 8 | 126468 | 126475 | rpl32_trnL-UAG |
| Paphiopedilum_armeniacum | p1 | (A)18 | 18 | 126893 | 126910 | trnL-UAG_ccsA |
| Paphiopedilum_armeniacum | p1 | (T)10 | 10 | 127510 | 127519 | ccsA |
| Paphiopedilum_armeniacum | p1 | (T)11 | 11 | 128129 | 128139 | ccsA_ndhD |
| Paphiopedilum_armeniacum | p1 | (A)9 | 9 | 129166 | 129174 | ndhD intron |
| Paphiopedilum_armeniacum | p1 | (A)8 | 8 | 130276 | 130283 | psaC_rps15 |
| Paphiopedilum_armeniacum | p1 | (A)10 | 10 | 130356 | 130365 | psaC_rps15 |
| Paphiopedilum_armeniacum | p1 | (T)8 | 8 | 131578 | 131585 | ycf1 |
| Paphiopedilum_armeniacum | p1 | (T)13 | 13 | 131951 | 131963 | ycf1 |
| Paphiopedilum_armeniacum | p1 | (T)9 | 9 | 132014 | 132022 | ycf1 |
| Paphiopedilum_armeniacum | p1 | (T)9 | 9 | 132395 | 132403 | ycf1 |
| Paphiopedilum_armeniacum | p1 | (T)8 | 8 | 132406 | 132413 | ycf1 |
| Paphiopedilum_armeniacum | p1 | (T)8 | 8 | 132744 | 132751 | ycf1 |
| Paphiopedilum_armeniacum | p1 | (T)8 | 8 | 132795 | 132802 | ycf1 |
| Paphiopedilum_armeniacum | p1 | (T)9 | 9 | 132926 | 132934 | ycf1 |
| Paphiopedilum_armeniacum | p1 | (T)9 | 9 | 133356 | 133364 | ycf1 |
| Paphiopedilum_armeniacum | p1 | (T)8 | 8 | 133559 | 133566 | ycf1 |
| Paphiopedilum_armeniacum | p1 | (T)9 | 9 | 133707 | 133715 | ycf1 |
| Paphiopedilum_armeniacum | p1 | (T)11 | 11 | 133841 | 133851 | ycf1 |
| Paphiopedilum_armeniacum | p1 | (A)8 | 8 | 134545 | 134552 | ycf1 |
| Paphiopedilum_armeniacum | p1 | (T)16 | 16 | 134961 | 134976 | ycf1 |
| Paphiopedilum_armeniacum | p1 | (T)9 | 9 | 135335 | 135343 | ycf1 |
| Paphiopedilum_armeniacum | p1 | (T)10 | 10 | 135456 | 135465 | ycf1 |
| Paphiopedilum_armeniacum | p1 | (A)12 | 12 | 135617 | 135628 | ycf1 |
| Paphiopedilum_armeniacum | p1 | (A)9 | 9 | 135711 | 135719 | ycf1 |
| Paphiopedilum_armeniacum | p1 | (T)10 | 10 | 135757 | 135766 | ycf1 |
| Paphiopedilum_armeniacum | p1 | (C)9 | 9 | 142985 | 142993 | trnI-GAU intron |
| Paphiopedilum_armeniacum | p1 | (A)9 | 9 | 145679 | 145687 | trnV-GAC_rps12 |
| Paphiopedilum_armeniacum | p1 | (A)9 | 9 | 146922 | 146930 | trnV-GAC_rps12 |
| Paphiopedilum_armeniacum | p1 | (A)8 | 8 | 146954 | 146961 | trnV-GAC_rps12 |
| Paphiopedilum_armeniacum | p1 | (T)9 | 9 | 156543 | 156551 | ycf2 |
| Paphiopedilum_armeniacum | p1 | (T)8 | 8 | 157836 | 157843 | ycf2 |
| Paphiopedilum_armeniacum | p1 | (G)8 | 8 | 160534 | 160541 | rpl2 |
| Paphiopedilum_armeniacum | p1 | (A)8 | 8 | 162105 | 162112 | trnH-GUG_rps19 |
| Paphiopedilum_armeniacum | p1 | (A)9 | 9 | 162140 | 162148 | rps19 |
| Paphiopedilum_dianthum | p1 | (T)8 | 8 | 1755 | 1762 | trnK-UUU intron |
| Paphiopedilum_dianthum | p1 | (T)9 | 9 | 2948 | 2956 | trnK-UUU intron |
| Paphiopedilum_dianthum | p1 | (A)8 | 8 | 3044 | 3051 | trnK-UUU intron |
| Paphiopedilum_dianthum | p1 | (T)11 | 11 | 3705 | 3715 | trnK-UUU intron |
| Paphiopedilum_dianthum | p1 | (A)9 | 9 | 3782 | 3790 | trnK-UUU intron |
| Paphiopedilum_dianthum | p1 | (A)9 | 9 | 4248 | 4256 | matK_rps16 |
| Paphiopedilum_dianthum | p1 | (A)17 | 17 | 4577 | 4593 | matK_rps16 |
| Paphiopedilum_dianthum | p1 | (C)10 | 10 | 4917 | 4926 | matK_rps16 |
| Paphiopedilum_dianthum | p1 | (G)8 | 8 | 4931 | 4938 | matK_rps16 |
| Paphiopedilum_dianthum | p1 | (C)11 | 11 | 5324 | 5334 | rps16 intron |
| Paphiopedilum_dianthum | p1 | (A)9 | 9 | 5486 | 5494 | rps16 intron |
| Paphiopedilum_dianthum | p1 | (T)11 | 11 | 5606 | 5616 | rps16 intron |
| Paphiopedilum_dianthum | p1 | (T)12 | 12 | 5764 | 5775 | rps16 intron |
| Paphiopedilum_dianthum | p1 | (T)9 | 9 | 5863 | 5871 | rps16 intron |
| Paphiopedilum_dianthum | p1 | (A)8 | 8 | 6399 | 6406 | rps16_trnQ-UUG |
| Paphiopedilum_dianthum | p1 | (T)9 | 9 | 6783 | 6791 | trnQ-UUG_psbK |
| Paphiopedilum_dianthum | p1 | (A)9 | 9 | 7028 | 7036 | trnQ-UUG_psbK |
| Paphiopedilum_dianthum | p1 | (A)12 | 12 | 8318 | 8329 | trnS-GCU_trnG-UCC |
| Paphiopedilum_dianthum | p1 | (T)8 | 8 | 9523 | 9530 | trnG-UCC intron |
| Paphiopedilum_dianthum | p1 | (A)8 | 8 | 9652 | 9659 | trnG-UCC intron |
| Paphiopedilum_dianthum | p1 | (A)9 | 9 | 10002 | 10010 | trnG-UCC intron |
| Paphiopedilum_dianthum | p1 | (T)8 | 8 | 10291 | 10298 | trnG-UCC_trnR-UCU |
| Paphiopedilum_dianthum | p1 | (A)9 | 9 | 12228 | 12236 | atpA_atpF |
| Paphiopedilum_dianthum | p1 | (A)8 | 8 | 12428 | 12435 | atpF |
| Paphiopedilum_dianthum | p1 | (T)12 | 12 | 12979 | 12990 | atpF intron |
| Paphiopedilum_dianthum | p1 | (T)22 | 22 | 13173 | 13194 | atpF intron |
| Paphiopedilum_dianthum | p1 | (A)10 | 10 | 13436 | 13445 | atpF intron |
| Paphiopedilum_dianthum | p1 | (A)8 | 8 | 13527 | 13534 | atpF |
| Paphiopedilum_dianthum | p1 | (A)12 | 12 | 14242 | 14253 | atpH_atpI |
| Paphiopedilum_dianthum | p1 | (T)9 | 9 | 14361 | 14369 | atpH_atpI |
| Paphiopedilum_dianthum | p1 | (A)11 | 11 | 15822 | 15832 | atpI_rps2 |
| Paphiopedilum_dianthum | p1 | (T)11 | 11 | 16702 | 16712 | rps2_rpoC2 |
| Paphiopedilum_dianthum | p1 | (T)11 | 11 | 16802 | 16812 | rps2_rpoC2 |
| Paphiopedilum_dianthum | p1 | (T)9 | 9 | 18741 | 18749 | rpoC2 |
| Paphiopedilum_dianthum | p1 | (T)8 | 8 | 18916 | 18923 | rpoC2 |
| Paphiopedilum_dianthum | p1 | (A)8 | 8 | 19062 | 19069 | rpoC2 |
| Paphiopedilum_dianthum | p1 | (A)8 | 8 | 22745 | 22752 | rpoC1 |
| Paphiopedilum_dianthum | p1 | (C)8 | 8 | 22879 | 22886 | rpoC1 intron |
| Paphiopedilum_dianthum | p1 | (T)8 | 8 | 23055 | 23062 | rpoC1 intron |
| Paphiopedilum_dianthum | p1 | (T)8 | 8 | 26682 | 26689 | rpoB |
| Paphiopedilum_dianthum | p1 | (T)10 | 10 | 28812 | 28821 | trnC-GCA_petN |
| Paphiopedilum_dianthum | p1 | (A)9 | 9 | 29266 | 29274 | petN_psbM |
| Paphiopedilum_dianthum | p1 | (A)9 | 9 | 30086 | 30094 | psbM_trnD-GUC |
| Paphiopedilum_dianthum | p1 | (A)9 | 9 | 31392 | 31400 | trnE-UUC_trnT-GGU |
| Paphiopedilum_dianthum | p1 | (A)8 | 8 | 32543 | 32550 | trnE-UUC_trnT-GGU |
| Paphiopedilum_dianthum | p1 | (T)16 | 16 | 32721 | 32736 | trnE-UUC_trnT-GGU |
| Paphiopedilum_dianthum | p1 | (A)14 | 14 | 32913 | 32926 | trnT-GGU_psbD |
| Paphiopedilum_dianthum | p1 | (A)11 | 11 | 37204 | 37214 | psbZ_trnG-GCC |
| Paphiopedilum_dianthum | p1 | (T)9 | 9 | 38100 | 38108 | rps14_psaB |
| Paphiopedilum_dianthum | p1 | (A)8 | 8 | 43158 | 43165 | psaA_ycf3 |
| Paphiopedilum_dianthum | p1 | (T)8 | 8 | 43971 | 43978 | ycf3 intron 1 |
| Paphiopedilum_dianthum | p1 | (T)19 | 19 | 47204 | 47222 | trnT-UGU_trnL-UAA |
| Paphiopedilum_dianthum | p1 | (A)13 | 13 | 47716 | 47728 | trnT-UGU_trnL-UAA |
| Paphiopedilum_dianthum | p1 | (T)9 | 9 | 48738 | 48746 | trnL-UAA_trnF-GAA |
| Paphiopedilum_dianthum | p1 | (T)9 | 9 | 49462 | 49470 | trnF-GAA_trnV-UAC |
| Paphiopedilum_dianthum | p1 | (T)8 | 8 | 52285 | 52292 | trnM-CAU_atpE |
| Paphiopedilum_dianthum | p1 | (T)9 | 9 | 54263 | 54271 | atpB_rbcL |
| Paphiopedilum_dianthum | p1 | (T)9 | 9 | 54642 | 54650 | atpB_rbcL |
| Paphiopedilum_dianthum | p1 | (T)8 | 8 | 57082 | 57089 | rbcL_accD |
| Paphiopedilum_dianthum | p1 | (T)9 | 9 | 57539 | 57547 | rbcL_accD |
| Paphiopedilum_dianthum | p1 | (A)9 | 9 | 57697 | 57705 | rbcL_accD |
| Paphiopedilum_dianthum | p1 | (A)9 | 9 | 58755 | 58763 | accD |
| Paphiopedilum_dianthum | p1 | (T)9 | 9 | 59542 | 59550 | accD_psaI |
| Paphiopedilum_dianthum | p1 | (C)9 | 9 | 59557 | 59565 | accD_psaI |
| Paphiopedilum_dianthum | p1 | (T)11 | 11 | 60552 | 60562 | psaI_ycf4 |
| Paphiopedilum_dianthum | p1 | (A)8 | 8 | 61471 | 61478 | ycf4_cemA |
| Paphiopedilum_dianthum | p1 | (A)8 | 8 | 63009 | 63016 | petA |
| Paphiopedilum_dianthum | p1 | (T)10 | 10 | 63921 | 63930 | petA_psbJ |
| Paphiopedilum_dianthum | p1 | (A)9 | 9 | 65332 | 65340 | psbE_petL |
| Paphiopedilum_dianthum | p1 | (T)8 | 8 | 65671 | 65678 | psbE_petL |
| Paphiopedilum_dianthum | p1 | (A)9 | 9 | 65725 | 65733 | psbE_petL |
| Paphiopedilum_dianthum | p1 | (T)8 | 8 | 65917 | 65924 | psbE_petL |
| Paphiopedilum_dianthum | p1 | (A)10 | 10 | 66202 | 66211 | petL_petG |
| Paphiopedilum_dianthum | p1 | (T)9 | 9 | 67062 | 67070 | trnP-UGG_psaJ |
| Paphiopedilum_dianthum | p1 | (T)22 | 22 | 67072 | 67093 | trnP-UGG_psaJ |
| Paphiopedilum_dianthum | p1 | (A)8 | 8 | 68260 | 68267 | psaJ_rpl33 |
| Paphiopedilum_dianthum | p1 | (T)9 | 9 | 69696 | 69704 | rps18_rpl20 |
| Paphiopedilum_dianthum | p1 | (T)8 | 8 | 70164 | 70171 | rpl20 |
| Paphiopedilum_dianthum | p1 | (T)11 | 11 | 70568 | 70578 | rpl20_rps12 |
| Paphiopedilum_dianthum | p1 | (T)11 | 11 | 70690 | 70700 | rpl20_rps12 |
| Paphiopedilum_dianthum | p1 | (T)12 | 12 | 71971 | 71982 | clpP intron 1 |
| Paphiopedilum_dianthum | p1 | (T)10 | 10 | 72203 | 72212 | clpP intron 1 |
| Paphiopedilum_dianthum | p1 | (T)9 | 9 | 72251 | 72259 | clpP intron 1 |
| Paphiopedilum_dianthum | p1 | (T)9 | 9 | 72674 | 72682 | clpP intron 2 |
| Paphiopedilum_dianthum | p1 | (T)11 | 11 | 72722 | 72732 | clpP intron 2 |
| Paphiopedilum_dianthum | p1 | (A)14 | 14 | 72787 | 72800 | clpP intron 2 |
| Paphiopedilum_dianthum | p1 | (T)10 | 10 | 72987 | 72996 | clpP intron 2 |
| Paphiopedilum_dianthum | p1 | (T)8 | 8 | 75288 | 75295 | psbB |
| Paphiopedilum_dianthum | p1 | (T)8 | 8 | 75938 | 75945 | psbB |
| Paphiopedilum_dianthum | p1 | (T)8 | 8 | 76163 | 76170 | psbB_psbT |
| Paphiopedilum_dianthum | p1 | (A)10 | 10 | 77629 | 77638 | petB intron |
| Paphiopedilum_dianthum | p1 | (A)10 | 10 | 81864 | 81873 | rps11_rpl36 |
| Paphiopedilum_dianthum | p1 | (T)10 | 10 | 82174 | 82183 | rpl36_infA |
| Paphiopedilum_dianthum | p1 | (T)9 | 9 | 82805 | 82813 | rps8 |
| Paphiopedilum_dianthum | p1 | (T)10 | 10 | 83065 | 83074 | rps8_rpl14 |
| Paphiopedilum_dianthum | p1 | (T)13 | 13 | 83856 | 83868 | rpl14_rpl16 |
| Paphiopedilum_dianthum | p1 | (A)8 | 8 | 84601 | 84608 | rpl16 intron |
| Paphiopedilum_dianthum | p1 | (T)9 | 9 | 85416 | 85424 | rpl16 intron |
| Paphiopedilum_dianthum | p1 | (T)8 | 8 | 85602 | 85609 | rpl16 intron |
| Paphiopedilum_dianthum | p1 | (T)11 | 11 | 86574 | 86584 | rpl22 |
| Paphiopedilum_dianthum | p1 | (T)9 | 9 | 87379 | 87387 | rps19 |
| Paphiopedilum_dianthum | p1 | (T)8 | 8 | 87415 | 87422 | rps19_trnH-GUG |
| Paphiopedilum_dianthum | p1 | (A)8 | 8 | 91617 | 91624 | ycf2 |
| Paphiopedilum_dianthum | p1 | (A)9 | 9 | 92909 | 92917 | ycf2 |
| Paphiopedilum_dianthum | p1 | (T)8 | 8 | 101126 | 101133 | rps12_trnV-GAC |
| Paphiopedilum_dianthum | p1 | (T)9 | 9 | 101157 | 101165 | rps12_trnV-GAC |
| Paphiopedilum_dianthum | p1 | (G)10 | 10 | 105566 | 105575 | trnI-GAU intron |
| Paphiopedilum_dianthum | p1 | (T)8 | 8 | 105768 | 105775 | trnI-GAU intron |
| Paphiopedilum_dianthum | p1 | (T)9 | 9 | 112970 | 112978 | ycf1 |
| Paphiopedilum_dianthum | p1 | (T)10 | 10 | 113061 | 113070 | ycf1 |
| Paphiopedilum_dianthum | p1 | (A)10 | 10 | 113224 | 113233 | ycf1 |
| Paphiopedilum_dianthum | p1 | (A)9 | 9 | 113346 | 113354 | ycf1 |
| Paphiopedilum_dianthum | p1 | (A)8 | 8 | 113549 | 113556 | ycf1 |
| Paphiopedilum_dianthum | p1 | (T)8 | 8 | 114167 | 114174 | ycf1 |
| Paphiopedilum_dianthum | p1 | (A)11 | 11 | 115156 | 115166 | ycf1 |
| Paphiopedilum_dianthum | p1 | (A)9 | 9 | 115349 | 115357 | ycf1 |
| Paphiopedilum_dianthum | p1 | (A)8 | 8 | 115777 | 115784 | ycf1 |
| Paphiopedilum_dianthum | p1 | (A)8 | 8 | 115908 | 115915 | ycf1 |
| Paphiopedilum_dianthum | p1 | (A)8 | 8 | 115959 | 115966 | ycf1 |
| Paphiopedilum_dianthum | p1 | (A)9 | 9 | 116307 | 116315 | ycf1 |
| Paphiopedilum_dianthum | p1 | (A)13 | 13 | 116747 | 116759 | ycf1 |
| Paphiopedilum_dianthum | p1 | (A)8 | 8 | 117125 | 117132 | ycf1 |
| Paphiopedilum_dianthum | p1 | (T)9 | 9 | 117579 | 117587 | ycf1_rps15 |
| Paphiopedilum_dianthum | p1 | (T)18 | 18 | 118322 | 118339 | rps15_psaC |
| Paphiopedilum_dianthum | p1 | (A)9 | 9 | 119862 | 119870 | rpl32 |
| Paphiopedilum_dianthum | p1 | (T)9 | 9 | 119936 | 119944 | rpl32 |
| Paphiopedilum_dianthum | p1 | (T)8 | 8 | 120028 | 120035 | rpl32_ccsA |
| Paphiopedilum_dianthum | p1 | (T)9 | 9 | 120623 | 120631 | ccsA |
| Paphiopedilum_dianthum | p1 | (A)18 | 18 | 123222 | 123239 | psaC_rps15 |
| Paphiopedilum_dianthum | p1 | (A)9 | 9 | 123974 | 123982 | rps15_ycf1 |
| Paphiopedilum_dianthum | p1 | (T)8 | 8 | 124429 | 124436 | ycf1 |
| Paphiopedilum_dianthum | p1 | (T)13 | 13 | 124802 | 124814 | ycf1 |
| Paphiopedilum_dianthum | p1 | (T)9 | 9 | 125246 | 125254 | ycf1 |
| Paphiopedilum_dianthum | p1 | (T)8 | 8 | 125595 | 125602 | ycf1 |
| Paphiopedilum_dianthum | p1 | (T)8 | 8 | 125646 | 125653 | ycf1 |
| Paphiopedilum_dianthum | p1 | (T)8 | 8 | 125777 | 125784 | ycf1 |
| Paphiopedilum_dianthum | p1 | (T)9 | 9 | 126204 | 126212 | ycf1 |
| Paphiopedilum_dianthum | p1 | (T)11 | 11 | 126395 | 126405 | ycf1 |
| Paphiopedilum_dianthum | p1 | (A)8 | 8 | 127387 | 127394 | ycf1 |
| Paphiopedilum_dianthum | p1 | (T)8 | 8 | 128005 | 128012 | ycf1 |
| Paphiopedilum_dianthum | p1 | (T)9 | 9 | 128207 | 128215 | ycf1 |
| Paphiopedilum_dianthum | p1 | (T)10 | 10 | 128328 | 128337 | ycf1 |
| Paphiopedilum_dianthum | p1 | (A)10 | 10 | 128491 | 128500 | ycf1 |
| Paphiopedilum_dianthum | p1 | (A)9 | 9 | 128583 | 128591 | ycf1 |
| Paphiopedilum_dianthum | p1 | (A)8 | 8 | 135786 | 135793 | trnI-GAU intron |
| Paphiopedilum_dianthum | p1 | (C)10 | 10 | 135986 | 135995 | trnI-GAU intron |
| Paphiopedilum_dianthum | p1 | (A)9 | 9 | 140396 | 140404 | trnV-GAC_rps12 |
| Paphiopedilum_dianthum | p1 | (A)8 | 8 | 140428 | 140435 | trnV-GAC_rps12 |
| Paphiopedilum_dianthum | p1 | (T)9 | 9 | 148644 | 148652 | ycf2 |
| Paphiopedilum_dianthum | p1 | (T)8 | 8 | 149937 | 149944 | ycf2 |
| Paphiopedilum_dianthum | p1 | (A)8 | 8 | 154139 | 154146 | trnH-GUG_rps19 |
| Paphiopedilum_dianthum | p1 | (A)9 | 9 | 154174 | 154182 | rps19 |
| Paphiopedilum_violascens | p1 | (T)8 | 8 | 1750 | 1757 | trnK-UUU intron |
| Paphiopedilum_violascens | p1 | (T)10 | 10 | 1759 | 1768 | trnK-UUU intron |
| Paphiopedilum_violascens | p1 | (T)9 | 9 | 2954 | 2962 | trnK-UUU intron |
| Paphiopedilum_violascens | p1 | (A)8 | 8 | 3062 | 3069 | trnK-UUU intron |
| Paphiopedilum_violascens | p1 | (T)9 | 9 | 3564 | 3572 | trnK-UUU intron |
| Paphiopedilum_violascens | p1 | (T)8 | 8 | 4045 | 4052 | trnK-UUU intron |
| Paphiopedilum_violascens | p1 | (A)10 | 10 | 4119 | 4128 | trnK-UUU intron |
| Paphiopedilum_violascens | p1 | (A)9 | 9 | 4589 | 4597 | matK_rps16 |
| Paphiopedilum_violascens | p1 | (T)10 | 10 | 4931 | 4940 | matK_rps16 |
| Paphiopedilum_violascens | p1 | (T)9 | 9 | 4942 | 4950 | matK_rps16 |
| Paphiopedilum_violascens | p1 | (C)9 | 9 | 5268 | 5276 | matK_rps16 |
| Paphiopedilum_violascens | p1 | (G)8 | 8 | 5281 | 5288 | matK_rps16 |
| Paphiopedilum_violascens | p1 | (A)9 | 9 | 5810 | 5818 | rps16 intron |
| Paphiopedilum_violascens | p1 | (T)11 | 11 | 5930 | 5940 | rps16 intron |
| Paphiopedilum_violascens | p1 | (T)9 | 9 | 6185 | 6193 | rps16 intron |
| Paphiopedilum_violascens | p1 | (A)8 | 8 | 6721 | 6728 | rps16_trnQ-UUG |
| Paphiopedilum_violascens | p1 | (T)8 | 8 | 6941 | 6948 | trnQ-UUG_psbK |
| Paphiopedilum_violascens | p1 | (A)9 | 9 | 7493 | 7501 | psbK_psbI |
| Paphiopedilum_violascens | p1 | (A)15 | 15 | 8381 | 8395 | trnS-GCU_trnG-UCC |
| Paphiopedilum_violascens | p1 | (A)8 | 8 | 9520 | 9527 | trnG-UCC intron |
| Paphiopedilum_violascens | p1 | (A)9 | 9 | 12035 | 12043 | atpA_atpF |
| Paphiopedilum_violascens | p1 | (A)8 | 8 | 12235 | 12242 | atpF |
| Paphiopedilum_violascens | p1 | (T)9 | 9 | 12793 | 12801 | atpF intron |
| Paphiopedilum_violascens | p1 | (T)9 | 9 | 12987 | 12995 | atpF intron |
| Paphiopedilum_violascens | p1 | (A)8 | 8 | 13237 | 13244 | atpF intron |
| Paphiopedilum_violascens | p1 | (A)8 | 8 | 13326 | 13333 | atpF |
| Paphiopedilum_violascens | p1 | (T)8 | 8 | 13529 | 13536 | atpF_atpH |
| Paphiopedilum_violascens | p1 | (T)8 | 8 | 13760 | 13767 | atpF_atpH |
| Paphiopedilum_violascens | p1 | (A)15 | 15 | 14050 | 14064 | atpH_atpI |
| Paphiopedilum_violascens | p1 | (T)9 | 9 | 14171 | 14179 | atpH_atpI |
| Paphiopedilum_violascens | p1 | (A)11 | 11 | 15641 | 15651 | atpI_rps2 |
| Paphiopedilum_violascens | p1 | (T)16 | 16 | 16521 | 16536 | rps2_rpoC2 |
| Paphiopedilum_violascens | p1 | (A)8 | 8 | 16783 | 16790 | rps2_rpoC2 |
| Paphiopedilum_violascens | p1 | (T)9 | 9 | 18562 | 18570 | rpoC2 |
| Paphiopedilum_violascens | p1 | (T)8 | 8 | 18737 | 18744 | rpoC2 |
| Paphiopedilum_violascens | p1 | (A)8 | 8 | 18883 | 18890 | rpoC2 |
| Paphiopedilum_violascens | p1 | (A)8 | 8 | 22566 | 22573 | rpoC1 |
| Paphiopedilum_violascens | p1 | (C)10 | 10 | 22700 | 22709 | rpoC1 intron |
| Paphiopedilum_violascens | p1 | (T)8 | 8 | 22878 | 22885 | rpoC1 intron |
| Paphiopedilum_violascens | p1 | (T)8 | 8 | 26496 | 26503 | rpoB |
| Paphiopedilum_violascens | p1 | (T)13 | 13 | 28588 | 28600 | trnC-GCA_petN |
| Paphiopedilum_violascens | p1 | (T)9 | 9 | 28798 | 28806 | trnC-GCA_petN |
| Paphiopedilum_violascens | p1 | (A)12 | 12 | 29211 | 29222 | petN_psbM |
| Paphiopedilum_violascens | p1 | (A)9 | 9 | 30809 | 30817 | psbM_trnD-GUC |
| Paphiopedilum_violascens | p1 | (T)9 | 9 | 31550 | 31558 | trnD-GUC_trnY-GUA |
| Paphiopedilum_violascens | p1 | (T)10 | 10 | 31583 | 31592 | trnD-GUC_trnY-GUA |
| Paphiopedilum_violascens | p1 | (T)8 | 8 | 32105 | 32112 | trnE-UUC_trnT-GGU |
| Paphiopedilum_violascens | p1 | (A)9 | 9 | 32213 | 32221 | trnE-UUC_trnT-GGU |
| Paphiopedilum_violascens | p1 | (A)9 | 9 | 33242 | 33250 | trnE-UUC_trnT-GGU |
| Paphiopedilum_violascens | p1 | (T)8 | 8 | 33414 | 33421 | trnE-UUC_trnT-GGU |
| Paphiopedilum_violascens | p1 | (A)9 | 9 | 33704 | 33712 | trnT-GGU_psbD |
| Paphiopedilum_violascens | p1 | (T)10 | 10 | 38885 | 38894 | rps14_psaB |
| Paphiopedilum_violascens | p1 | (G)8 | 8 | 38898 | 38905 | rps14_psaB |
| Paphiopedilum_violascens | p1 | (A)8 | 8 | 44055 | 44062 | psaA_ycf3 |
| Paphiopedilum_violascens | p1 | (A)8 | 8 | 46495 | 46502 | ycf3_trnS-GGA |
| Paphiopedilum_violascens | p1 | (A)11 | 11 | 47607 | 47617 | rps4_trnT-UGU |
| Paphiopedilum_violascens | p1 | (T)13 | 13 | 48163 | 48175 | trnT-UGU_trnL-UAA |
| Paphiopedilum_violascens | p1 | (A)11 | 11 | 48829 | 48839 | trnT-UGU_trnL-UAA |
| Paphiopedilum_violascens | p1 | (T)11 | 11 | 49848 | 49858 | trnL-UAA_trnF-GAA |
| Paphiopedilum_violascens | p1 | (T)14 | 14 | 50546 | 50559 | trnF-GAA_ndhJ |
| Paphiopedilum_violascens | p1 | (C)8 | 8 | 50584 | 50591 | trnF-GAA_ndhJ |
| Paphiopedilum_violascens | p1 | (T)9 | 9 | 51220 | 51228 | ndhJ_ndhK |
| Paphiopedilum_violascens | p1 | (T)15 | 15 | 55912 | 55926 | atpB_rbcL |
| Paphiopedilum_violascens | p1 | (T)10 | 10 | 56635 | 56644 | atpB_rbcL |
| Paphiopedilum_violascens | p1 | (T)8 | 8 | 58237 | 58244 | rbcL_accD |
| Paphiopedilum_violascens | p1 | (T)9 | 9 | 58681 | 58689 | rbcL_accD |
| Paphiopedilum_violascens | p1 | (A)9 | 9 | 58847 | 58855 | rbcL_accD |
| Paphiopedilum_violascens | p1 | (A)10 | 10 | 59843 | 59852 | accD |
| Paphiopedilum_violascens | p1 | (A)13 | 13 | 60408 | 60420 | accD |
| Paphiopedilum_violascens | p1 | (A)8 | 8 | 60733 | 60740 | accD_psaI |
| Paphiopedilum_violascens | p1 | (A)9 | 9 | 60804 | 60812 | accD_psaI |
| Paphiopedilum_violascens | p1 | (A)8 | 8 | 62567 | 62574 | ycf4_petA |
| Paphiopedilum_violascens | p1 | (A)8 | 8 | 63379 | 63386 | petA |
| Paphiopedilum_violascens | p1 | (T)8 | 8 | 63988 | 63995 | petA_psbJ |
| Paphiopedilum_violascens | p1 | (T)8 | 8 | 64292 | 64299 | petA_psbJ |
| Paphiopedilum_violascens | p1 | (C)8 | 8 | 65092 | 65099 | psbJ_psbL |
| Paphiopedilum_violascens | p1 | (G)9 | 9 | 65785 | 65793 | psbE_petL |
| Paphiopedilum_violascens | p1 | (T)8 | 8 | 66143 | 66150 | psbE_petL |
| Paphiopedilum_violascens | p1 | (A)11 | 11 | 66280 | 66290 | psbE_petL |
| Paphiopedilum_violascens | p1 | (T)8 | 8 | 66474 | 66481 | psbE_petL |
| Paphiopedilum_violascens | p1 | (A)11 | 11 | 66759 | 66769 | petL_petG |
| Paphiopedilum_violascens | p1 | (T)14 | 14 | 67613 | 67626 | trnP-UGG_psaJ |
| Paphiopedilum_violascens | p1 | (A)8 | 8 | 68640 | 68647 | psaJ_rpl33 |
| Paphiopedilum_violascens | p1 | (A)8 | 8 | 69336 | 69343 | rpl33_rps18 |
| Paphiopedilum_violascens | p1 | (T)9 | 9 | 70029 | 70037 | rps18_rpl20 |
| Paphiopedilum_violascens | p1 | (T)8 | 8 | 70504 | 70511 | rpl20 |
| Paphiopedilum_violascens | p1 | (T)11 | 11 | 70907 | 70917 | rpl20_rps12 |
| Paphiopedilum_violascens | p1 | (T)9 | 9 | 71029 | 71037 | rpl20_rps12 |
| Paphiopedilum_violascens | p1 | (T)8 | 8 | 72453 | 72460 | clpP intron 1 |
| Paphiopedilum_violascens | p1 | (T)13 | 13 | 72682 | 72694 | clpP intron 1 |
| Paphiopedilum_violascens | p1 | (T)10 | 10 | 72739 | 72748 | clpP intron 1 |
| Paphiopedilum_violascens | p1 | (T)10 | 10 | 73163 | 73172 | clpP intron 2 |
| Paphiopedilum_violascens | p1 | (T)13 | 13 | 73212 | 73224 | clpP intron 2 |
| Paphiopedilum_violascens | p1 | (A)14 | 14 | 73279 | 73292 | clpP intron 2 |
| Paphiopedilum_violascens | p1 | (T)10 | 10 | 73479 | 73488 | clpP intron 2 |
| Paphiopedilum_violascens | p1 | (T)8 | 8 | 75897 | 75904 | psbB |
| Paphiopedilum_violascens | p1 | (T)8 | 8 | 76547 | 76554 | psbB |
| Paphiopedilum_violascens | p1 | (A)12 | 12 | 78421 | 78432 | petB intron |
| Paphiopedilum_violascens | p1 | (T)8 | 8 | 79829 | 79836 | petD intron |
| Paphiopedilum_violascens | p1 | (A)8 | 8 | 82656 | 82663 | rps11_rpl36 |
| Paphiopedilum_violascens | p1 | (T)8 | 8 | 82946 | 82953 | rpl36_infA |
| Paphiopedilum_violascens | p1 | (T)10 | 10 | 82969 | 82978 | rpl36_infA |
| Paphiopedilum_violascens | p1 | (T)9 | 9 | 83599 | 83607 | rps8 |
| Paphiopedilum_violascens | p1 | (T)8 | 8 | 83859 | 83866 | rps8_rpl14 |
| Paphiopedilum_violascens | p1 | (T)18 | 18 | 84566 | 84583 | rpl14_rpl16 |
| Paphiopedilum_violascens | p1 | (A)8 | 8 | 85315 | 85322 | rpl16 intron |
| Paphiopedilum_violascens | p1 | (T)8 | 8 | 86230 | 86237 | rpl16 intron |
| Paphiopedilum_violascens | p1 | (T)8 | 8 | 86415 | 86422 | rpl16 intron |
| Paphiopedilum_violascens | p1 | (T)8 | 8 | 87387 | 87394 | rpl22 |
| Paphiopedilum_violascens | p1 | (T)9 | 9 | 88192 | 88200 | rps19 |
| Paphiopedilum_violascens | p1 | (T)8 | 8 | 88228 | 88235 | rps19_trnH-GUG |
| Paphiopedilum_violascens | p1 | (A)8 | 8 | 92497 | 92504 | ycf2 |
| Paphiopedilum_violascens | p1 | (A)8 | 8 | 93790 | 93797 | ycf2 |
| Paphiopedilum_violascens | p1 | (T)10 | 10 | 103463 | 103472 | rps12_trnV-GAC |
| Paphiopedilum_violascens | p1 | (G)9 | 9 | 106158 | 106166 | trnI-GAU intron |
| Paphiopedilum_violascens | p1 | (T)8 | 8 | 106359 | 106366 | trnI-GAU intron |
| Paphiopedilum_violascens | p1 | (T)9 | 9 | 113508 | 113516 | ycf1 |
| Paphiopedilum_violascens | p1 | (T)10 | 10 | 113599 | 113608 | ycf1 |
| Paphiopedilum_violascens | p1 | (A)10 | 10 | 113762 | 113771 | ycf1 |
| Paphiopedilum_violascens | p1 | (A)9 | 9 | 113884 | 113892 | ycf1 |
| Paphiopedilum_violascens | p1 | (A)8 | 8 | 114087 | 114094 | ycf1 |
| Paphiopedilum_violascens | p1 | (T)8 | 8 | 114693 | 114700 | ycf1 |
| Paphiopedilum_violascens | p1 | (A)11 | 11 | 115682 | 115692 | ycf1 |
| Paphiopedilum_violascens | p1 | (A)9 | 9 | 115875 | 115883 | ycf1 |
| Paphiopedilum_violascens | p1 | (A)8 | 8 | 116303 | 116310 | ycf1 |
| Paphiopedilum_violascens | p1 | (A)8 | 8 | 116434 | 116441 | ycf1 |
| Paphiopedilum_violascens | p1 | (A)8 | 8 | 116485 | 116492 | ycf1 |
| Paphiopedilum_violascens | p1 | (A)8 | 8 | 116823 | 116830 | ycf1 |
| Paphiopedilum_violascens | p1 | (A)9 | 9 | 116833 | 116841 | ycf1 |
| Paphiopedilum_violascens | p1 | (A)13 | 13 | 117273 | 117285 | ycf1 |
| Paphiopedilum_violascens | p1 | (A)8 | 8 | 117651 | 117658 | ycf1 |
| Paphiopedilum_violascens | p1 | (T)8 | 8 | 118105 | 118112 | ycf1_rps15 |
| Paphiopedilum_violascens | p1 | (T)13 | 13 | 118762 | 118774 | rps15_psaC |
| Paphiopedilum_violascens | p1 | (T)11 | 11 | 118973 | 118983 | rps15_psaC |
| Paphiopedilum_violascens | p1 | (A)10 | 10 | 121717 | 121726 | ndhD_trnL-UAG |
| Paphiopedilum_violascens | p1 | (A)13 | 13 | 122125 | 122137 | ndhD_trnL-UAG |
| Paphiopedilum_violascens | p1 | (A)9 | 9 | 123004 | 123012 | rpl32 |
| Paphiopedilum_violascens | p1 | (T)10 | 10 | 123221 | 123230 | rpl32_ccsA |
| Paphiopedilum_violascens | p1 | (T)10 | 10 | 123802 | 123811 | ccsA |
| Paphiopedilum_violascens | p1 | (A)11 | 11 | 126545 | 126555 | psaC_rps15 |
| Paphiopedilum_violascens | p1 | (A)13 | 13 | 126754 | 126766 | psaC_rps15 |
| Paphiopedilum_violascens | p1 | (A)8 | 8 | 127416 | 127423 | rps15_ycf1 |
| Paphiopedilum_violascens | p1 | (T)8 | 8 | 127870 | 127877 | ycf1 |
| Paphiopedilum_violascens | p1 | (T)13 | 13 | 128243 | 128255 | ycf1 |
| Paphiopedilum_violascens | p1 | (T)9 | 9 | 128687 | 128695 | ycf1 |
| Paphiopedilum_violascens | p1 | (T)8 | 8 | 128698 | 128705 | ycf1 |
| Paphiopedilum_violascens | p1 | (T)8 | 8 | 129036 | 129043 | ycf1 |
| Paphiopedilum_violascens | p1 | (T)8 | 8 | 129087 | 129094 | ycf1 |
| Paphiopedilum_violascens | p1 | (T)8 | 8 | 129218 | 129225 | ycf1 |
| Paphiopedilum_violascens | p1 | (T)9 | 9 | 129645 | 129653 | ycf1 |
| Paphiopedilum_violascens | p1 | (T)11 | 11 | 129836 | 129846 | ycf1 |
| Paphiopedilum_violascens | p1 | (A)8 | 8 | 130828 | 130835 | ycf1 |
| Paphiopedilum_violascens | p1 | (T)8 | 8 | 131434 | 131441 | ycf1 |
| Paphiopedilum_violascens | p1 | (T)9 | 9 | 131636 | 131644 | ycf1 |
| Paphiopedilum_violascens | p1 | (T)10 | 10 | 131757 | 131766 | ycf1 |
| Paphiopedilum_violascens | p1 | (A)10 | 10 | 131920 | 131929 | ycf1 |
| Paphiopedilum_violascens | p1 | (A)9 | 9 | 132012 | 132020 | ycf1 |
| Paphiopedilum_violascens | p1 | (A)8 | 8 | 139162 | 139169 | trnI-GAU intron |
| Paphiopedilum_violascens | p1 | (C)9 | 9 | 139362 | 139370 | trnI-GAU intron |
| Paphiopedilum_violascens | p1 | (A)10 | 10 | 142056 | 142065 | trnV-GAC_rps12 |
| Paphiopedilum_violascens | p1 | (T)8 | 8 | 151731 | 151738 | ycf2 |
| Paphiopedilum_violascens | p1 | (T)8 | 8 | 153024 | 153031 | ycf2 |
| Paphiopedilum_violascens | p1 | (A)8 | 8 | 157293 | 157300 | trnH-GUG_rps19 |
| Paphiopedilum_violascens | p1 | (A)9 | 9 | 157328 | 157336 | rps19 |
| Paphiopedilum_niveum | p1 | (T)8 | 8 | 1762 | 1769 | trnK-UUU intron |
| Paphiopedilum_niveum | p1 | (T)10 | 10 | 1771 | 1780 | trnK-UUU intron |
| Paphiopedilum_niveum | p1 | (T)9 | 9 | 2056 | 2064 | trnK-UUU intron |
| Paphiopedilum_niveum | p1 | (T)9 | 9 | 2966 | 2974 | trnK-UUU intron |
| Paphiopedilum_niveum | p1 | (A)8 | 8 | 3074 | 3081 | trnK-UUU intron |
| Paphiopedilum_niveum | p1 | (T)9 | 9 | 3587 | 3595 | trnK-UUU intron |
| Paphiopedilum_niveum | p1 | (T)8 | 8 | 4126 | 4133 | trnK-UUU intron |
| Paphiopedilum_niveum | p1 | (A)9 | 9 | 4200 | 4208 | trnK-UUU intron |
| Paphiopedilum_niveum | p1 | (A)10 | 10 | 4656 | 4665 | matK_rps16 |
| Paphiopedilum_niveum | p1 | (A)9 | 9 | 5010 | 5018 | matK_rps16 |
| Paphiopedilum_niveum | p1 | (A)11 | 11 | 5356 | 5366 | matK_rps16 |
| Paphiopedilum_niveum | p1 | (C)8 | 8 | 5439 | 5446 | matK_rps16 |
| Paphiopedilum_niveum | p1 | (C)9 | 9 | 5843 | 5851 | rps16 intron |
| Paphiopedilum_niveum | p1 | (T)8 | 8 | 6039 | 6046 | rps16 intron |
| Paphiopedilum_niveum | p1 | (A)9 | 9 | 6063 | 6071 | rps16 intron |
| Paphiopedilum_niveum | p1 | (T)11 | 11 | 6183 | 6193 | rps16 intron |
| Paphiopedilum_niveum | p1 | (T)9 | 9 | 6442 | 6450 | rps16 intron |
| Paphiopedilum_niveum | p1 | (A)9 | 9 | 6994 | 7002 | rps16_trnQ-UUG |
| Paphiopedilum_niveum | p1 | (T)10 | 10 | 7202 | 7211 | rps16_trnQ-UUG |
| Paphiopedilum_niveum | p1 | (A)11 | 11 | 7481 | 7491 | rps16_trnQ-UUG |
| Paphiopedilum_niveum | p1 | (T)10 | 10 | 8064 | 8073 | trnQ-UUG_psbK |
| Paphiopedilum_niveum | p1 | (A)9 | 9 | 9313 | 9321 | trnS-GCU_trnG-GCC |
| Paphiopedilum_niveum | p1 | (T)8 | 8 | 10068 | 10075 | trnS-GCU_trnG-GCC |
| Paphiopedilum_niveum | p1 | (A)8 | 8 | 10454 | 10461 | trnG-GCC intron |
| Paphiopedilum_niveum | p1 | (A)8 | 8 | 10810 | 10817 | trnG-GCC intron |
| Paphiopedilum_niveum | p1 | (T)10 | 10 | 10843 | 10852 | trnG-GCC intron |
| Paphiopedilum_niveum | p1 | (T)8 | 8 | 11098 | 11105 | trnG-GCC_trnR-UCU |
| Paphiopedilum_niveum | p1 | (A)9 | 9 | 11114 | 11122 | trnG-GCC_trnR-UCU |
| Paphiopedilum_niveum | p1 | (A)9 | 9 | 12988 | 12996 | atpA_atpF |
| Paphiopedilum_niveum | p1 | (A)8 | 8 | 13188 | 13195 | atpF |
| Paphiopedilum_niveum | p1 | (T)8 | 8 | 13739 | 13746 | atpF intron |
| Paphiopedilum_niveum | p1 | (T)9 | 9 | 13918 | 13926 | atpF intron |
| Paphiopedilum_niveum | p1 | (T)15 | 15 | 13931 | 13945 | atpF intron |
| Paphiopedilum_niveum | p1 | (A)8 | 8 | 14187 | 14194 | atpF intron |
| Paphiopedilum_niveum | p1 | (A)8 | 8 | 14276 | 14283 | atpF |
| Paphiopedilum_niveum | p1 | (T)8 | 8 | 14479 | 14486 | atpF_atpH |
| Paphiopedilum_niveum | p1 | (A)12 | 12 | 14993 | 15004 | atpH_atpI |
| Paphiopedilum_niveum | p1 | (T)9 | 9 | 15116 | 15124 | atpH_atpI |
| Paphiopedilum_niveum | p1 | (A)8 | 8 | 16575 | 16582 | atpI_rps2 |
| Paphiopedilum_niveum | p1 | (T)9 | 9 | 17452 | 17460 | rps2_rpoC2 |
| Paphiopedilum_niveum | p1 | (A)8 | 8 | 17705 | 17712 | rps2_rpoC2 |
| Paphiopedilum_niveum | p1 | (T)9 | 9 | 19484 | 19492 | rpoC2 |
| Paphiopedilum_niveum | p1 | (T)8 | 8 | 19659 | 19666 | rpoC2 |
| Paphiopedilum_niveum | p1 | (A)8 | 8 | 19805 | 19812 | rpoC2 |
| Paphiopedilum_niveum | p1 | (A)8 | 8 | 23488 | 23495 | rpoC1 |
| Paphiopedilum_niveum | p1 | (C)9 | 9 | 23622 | 23630 | rpoC1 intron |
| Paphiopedilum_niveum | p1 | (T)8 | 8 | 23799 | 23806 | rpoC1 intron |
| Paphiopedilum_niveum | p1 | (T)8 | 8 | 27422 | 27429 | rpoB |
| Paphiopedilum_niveum | p1 | (T)10 | 10 | 29540 | 29549 | trnC-GCA_petN |
| Paphiopedilum_niveum | p1 | (T)8 | 8 | 29644 | 29651 | trnC-GCA_petN |
| Paphiopedilum_niveum | p1 | (A)17 | 17 | 30046 | 30062 | petN_psbM |
| Paphiopedilum_niveum | p1 | (A)9 | 9 | 30744 | 30752 | psbM_trnD-GUC |
| Paphiopedilum_niveum | p1 | (A)18 | 18 | 32135 | 32152 | trnE-UUC_trnT-GGU |
| Paphiopedilum_niveum | p1 | (T)8 | 8 | 33419 | 33426 | trnE-UUC_trnT-GGU |
| Paphiopedilum_niveum | p1 | (A)13 | 13 | 33708 | 33720 | trnT-GGU_psbD |
| Paphiopedilum_niveum | p1 | (G)9 | 9 | 38900 | 38908 | rps14_psaB |
| Paphiopedilum_niveum | p1 | (A)8 | 8 | 44080 | 44087 | psaA_ycf3 |
| Paphiopedilum_niveum | p1 | (T)8 | 8 | 44894 | 44901 | ycf3 intron 1 |
| Paphiopedilum_niveum | p1 | (T)11 | 11 | 45834 | 45844 | ycf3 intron 2 |
| Paphiopedilum_niveum | p1 | (A)10 | 10 | 46479 | 46488 | ycf3_trnS-GGA |
| Paphiopedilum_niveum | p1 | (A)11 | 11 | 47592 | 47602 | rps4_trnT-UGU |
| Paphiopedilum_niveum | p1 | (T)8 | 8 | 47770 | 47777 | rps4_trnT-UGU |
| Paphiopedilum_niveum | p1 | (T)12 | 12 | 48079 | 48090 | trnT-UGU_trnL-UAA |
| Paphiopedilum_niveum | p1 | (A)21 | 21 | 48716 | 48736 | trnT-UGU_trnL-UAA |
| Paphiopedilum_niveum | p1 | (T)8 | 8 | 49742 | 49749 | trnL-UAA_trnF-GAA |
| Paphiopedilum_niveum | p1 | (T)9 | 9 | 50472 | 50480 | trnF-GAA_ndhJ |
| Paphiopedilum_niveum | p1 | (T)12 | 12 | 52685 | 52696 | ndhC_trnV-UAC |
| Paphiopedilum_niveum | p1 | (T)9 | 9 | 56517 | 56525 | atpB_rbcL |
| Paphiopedilum_niveum | p1 | (T)10 | 10 | 56901 | 56910 | atpB_rbcL |
| Paphiopedilum_niveum | p1 | (T)9 | 9 | 57615 | 57623 | atpB_rbcL |
| Paphiopedilum_niveum | p1 | (T)8 | 8 | 59216 | 59223 | rbcL_accD |
| Paphiopedilum_niveum | p1 | (A)8 | 8 | 59571 | 59578 | rbcL_accD |
| Paphiopedilum_niveum | p1 | (T)9 | 9 | 59698 | 59706 | rbcL_accD |
| Paphiopedilum_niveum | p1 | (A)13 | 13 | 59864 | 59876 | rbcL_accD |
| Paphiopedilum_niveum | p1 | (A)9 | 9 | 60856 | 60864 | accD |
| Paphiopedilum_niveum | p1 | (A)11 | 11 | 61420 | 61430 | accD |
| Paphiopedilum_niveum | p1 | (A)8 | 8 | 61744 | 61751 | accD_psaI |
| Paphiopedilum_niveum | p1 | (A)10 | 10 | 61815 | 61824 | accD_psaI |
| Paphiopedilum_niveum | p1 | (A)8 | 8 | 63599 | 63606 | ycf4_cemA |
| Paphiopedilum_niveum | p1 | (A)11 | 11 | 64173 | 64183 | cemA |
| Paphiopedilum_niveum | p1 | (A)8 | 8 | 65536 | 65543 | petA |
| Paphiopedilum_niveum | p1 | (T)9 | 9 | 66145 | 66153 | petA_psbJ |
| Paphiopedilum_niveum | p1 | (T)14 | 14 | 66450 | 66463 | petA_psbJ |
| Paphiopedilum_niveum | p1 | (G)9 | 9 | 67934 | 67942 | psbE_petL |
| Paphiopedilum_niveum | p1 | (A)10 | 10 | 68412 | 68421 | psbE_petL |
| Paphiopedilum_niveum | p1 | (T)8 | 8 | 68605 | 68612 | psbE_petL |
| Paphiopedilum_niveum | p1 | (A)12 | 12 | 68890 | 68901 | petL_petG |
| Paphiopedilum_niveum | p1 | (A)8 | 8 | 70629 | 70636 | psaJ_rpl33 |
| Paphiopedilum_niveum | p1 | (T)9 | 9 | 72046 | 72054 | rps18_rpl20 |
| Paphiopedilum_niveum | p1 | (T)8 | 8 | 72511 | 72518 | rpl20 |
| Paphiopedilum_niveum | p1 | (T)8 | 8 | 72910 | 72917 | rpl20_rps12 |
| Paphiopedilum_niveum | p1 | (T)9 | 9 | 73023 | 73031 | rpl20_rps12 |
| Paphiopedilum_niveum | p1 | (T)8 | 8 | 74254 | 74261 | clpP intron 1 |
| Paphiopedilum_niveum | p1 | (T)12 | 12 | 74482 | 74493 | clpP intron 1 |
| Paphiopedilum_niveum | p1 | (T)9 | 9 | 74528 | 74536 | clpP intron 1 |
| Paphiopedilum_niveum | p1 | (T)11 | 11 | 74951 | 74961 | clpP intron 2 |
| Paphiopedilum_niveum | p1 | (T)8 | 8 | 75001 | 75008 | clpP intron 2 |
| Paphiopedilum_niveum | p1 | (A)16 | 16 | 75063 | 75078 | clpP intron 2 |
| Paphiopedilum_niveum | p1 | (T)14 | 14 | 75265 | 75278 | clpP intron 2 |
| Paphiopedilum_niveum | p1 | (T)8 | 8 | 76299 | 76306 | clpP_psbB |
| Paphiopedilum_niveum | p1 | (T)8 | 8 | 77770 | 77777 | psbB |
| Paphiopedilum_niveum | p1 | (T)8 | 8 | 78420 | 78427 | psbB |
| Paphiopedilum_niveum | p1 | (T)11 | 11 | 79140 | 79150 | psbB_psbT |
| Paphiopedilum_niveum | p1 | (A)9 | 9 | 79893 | 79901 | psbH_petB |
| Paphiopedilum_niveum | p1 | (A)13 | 13 | 80474 | 80486 | petB intron |
| Paphiopedilum_niveum | p1 | (A)9 | 9 | 84713 | 84721 | rps11_rpl36 |
| Paphiopedilum_niveum | p1 | (T)8 | 8 | 84814 | 84821 | rps11_rpl36 |
| Paphiopedilum_niveum | p1 | (T)10 | 10 | 85026 | 85035 | rpl36_rps8 |
| Paphiopedilum_niveum | p1 | (T)9 | 9 | 85694 | 85702 | rps8 |
| Paphiopedilum_niveum | p1 | (T)8 | 8 | 85962 | 85969 | rps8_rpl14 |
| Paphiopedilum_niveum | p1 | (A)8 | 8 | 87535 | 87542 | rpl16 intron |
| Paphiopedilum_niveum | p1 | (T)12 | 12 | 88439 | 88450 | rpl16 intron |
| Paphiopedilum_niveum | p1 | (T)8 | 8 | 88628 | 88635 | rpl16 intron |
| Paphiopedilum_niveum | p1 | (A)10 | 10 | 88779 | 88788 | rpl16_rps3 |
| Paphiopedilum_niveum | p1 | (T)8 | 8 | 89668 | 89675 | rpl22 |
| Paphiopedilum_niveum | p1 | (T)9 | 9 | 90508 | 90516 | rps19 |
| Paphiopedilum_niveum | p1 | (T)8 | 8 | 90544 | 90551 | rps19_trnH-GUG |
| Paphiopedilum_niveum | p1 | (A)8 | 8 | 94813 | 94820 | ycf2 |
| Paphiopedilum_niveum | p1 | (A)9 | 9 | 95961 | 95969 | ycf2 |
| Paphiopedilum_niveum | p1 | (T)8 | 8 | 105526 | 105533 | rps12_trnV-GAC |
| Paphiopedilum_niveum | p1 | (T)12 | 12 | 105557 | 105568 | rps12_trnV-GAC |
| Paphiopedilum_niveum | p1 | (T)12 | 12 | 106934 | 106945 | rps12_trnV-GAC |
| Paphiopedilum_niveum | p1 | (G)11 | 11 | 109631 | 109641 | trnI-GAU intron |
| Paphiopedilum_niveum | p1 | (T)9 | 9 | 117042 | 117050 | ycf1 |
| Paphiopedilum_niveum | p1 | (T)10 | 10 | 117133 | 117142 | ycf1 |
| Paphiopedilum_niveum | p1 | (A)10 | 10 | 117296 | 117305 | ycf1 |
| Paphiopedilum_niveum | p1 | (A)9 | 9 | 117418 | 117426 | ycf1 |
| Paphiopedilum_niveum | p1 | (A)8 | 8 | 117621 | 117628 | ycf1 |
| Paphiopedilum_niveum | p1 | (T)8 | 8 | 118209 | 118216 | ycf1 |
| Paphiopedilum_niveum | p1 | (A)9 | 9 | 119055 | 119063 | ycf1 |
| Paphiopedilum_niveum | p1 | (A)11 | 11 | 119201 | 119211 | ycf1 |
| Paphiopedilum_niveum | p1 | (A)8 | 8 | 119822 | 119829 | ycf1 |
| Paphiopedilum_niveum | p1 | (A)8 | 8 | 119953 | 119960 | ycf1 |
| Paphiopedilum_niveum | p1 | (A)8 | 8 | 120004 | 120011 | ycf1 |
| Paphiopedilum_niveum | p1 | (A)8 | 8 | 120342 | 120349 | ycf1 |
| Paphiopedilum_niveum | p1 | (A)9 | 9 | 120352 | 120360 | ycf1 |
| Paphiopedilum_niveum | p1 | (A)13 | 13 | 120798 | 120810 | ycf1 |
| Paphiopedilum_niveum | p1 | (A)8 | 8 | 121176 | 121183 | ycf1 |
| Paphiopedilum_niveum | p1 | (T)8 | 8 | 121630 | 121637 | ycf1_trnL-UAG |
| Paphiopedilum_niveum | p1 | (A)9 | 9 | 122958 | 122966 | rpl32 |
| Paphiopedilum_niveum | p1 | (T)25 | 25 | 123170 | 123194 | rpl32_ccsA |
| Paphiopedilum_niveum | p1 | (T)10 | 10 | 123767 | 123776 | ccsA |
| Paphiopedilum_niveum | p1 | (A)8 | 8 | 126491 | 126498 | psaC_rps15 |
| Paphiopedilum_niveum | p1 | (A)19 | 19 | 126671 | 126689 | psaC_rps15 |
| Paphiopedilum_niveum | p1 | (A)8 | 8 | 127430 | 127437 | rps15_ycf1 |
| Paphiopedilum_niveum | p1 | (T)8 | 8 | 127884 | 127891 | ycf1 |
| Paphiopedilum_niveum | p1 | (T)13 | 13 | 128257 | 128269 | ycf1 |
| Paphiopedilum_niveum | p1 | (T)9 | 9 | 128707 | 128715 | ycf1 |
| Paphiopedilum_niveum | p1 | (T)8 | 8 | 128718 | 128725 | ycf1 |
| Paphiopedilum_niveum | p1 | (T)8 | 8 | 129056 | 129063 | ycf1 |
| Paphiopedilum_niveum | p1 | (T)8 | 8 | 129107 | 129114 | ycf1 |
| Paphiopedilum_niveum | p1 | (T)8 | 8 | 129238 | 129245 | ycf1 |
| Paphiopedilum_niveum | p1 | (T)11 | 11 | 129856 | 129866 | ycf1 |
| Paphiopedilum_niveum | p1 | (T)9 | 9 | 130004 | 130012 | ycf1 |
| Paphiopedilum_niveum | p1 | (A)8 | 8 | 130851 | 130858 | ycf1 |
| Paphiopedilum_niveum | p1 | (T)8 | 8 | 131439 | 131446 | ycf1 |
| Paphiopedilum_niveum | p1 | (T)9 | 9 | 131641 | 131649 | ycf1 |
| Paphiopedilum_niveum | p1 | (T)10 | 10 | 131762 | 131771 | ycf1 |
| Paphiopedilum_niveum | p1 | (A)10 | 10 | 131925 | 131934 | ycf1 |
| Paphiopedilum_niveum | p1 | (A)9 | 9 | 132017 | 132025 | ycf1 |
| Paphiopedilum_niveum | p1 | (C)11 | 11 | 139426 | 139436 | trnI-GAU intron |
| Paphiopedilum_niveum | p1 | (A)12 | 12 | 142122 | 142133 | trnV-GAC_rps12 |
| Paphiopedilum_niveum | p1 | (A)12 | 12 | 143499 | 143510 | trnV-GAC_rps12 |
| Paphiopedilum_niveum | p1 | (A)8 | 8 | 143534 | 143541 | trnV-GAC_rps12 |
| Paphiopedilum_niveum | p1 | (T)9 | 9 | 153098 | 153106 | ycf2 |
| Paphiopedilum_niveum | p1 | (T)8 | 8 | 154247 | 154254 | ycf2 |
| Paphiopedilum_niveum | p1 | (A)8 | 8 | 158516 | 158523 | trnH-GUG_rps19 |
| Paphiopedilum_niveum | p1 | (A)9 | 9 | 158551 | 158559 | rps19 |
| Paphiopedilum_kolopakingii | p1 | (T)9 | 9 | 1731 | 1739 | trnK-UUU intron |
| Paphiopedilum_kolopakingii | p1 | (T)9 | 9 | 2925 | 2933 | trnK-UUU intron |
| Paphiopedilum_kolopakingii | p1 | (A)8 | 8 | 3033 | 3040 | trnK-UUU |
| Paphiopedilum_kolopakingii | p1 | (T)11 | 11 | 3554 | 3564 | matK_rps16 |
| Paphiopedilum_kolopakingii | p1 | (T)8 | 8 | 4104 | 4111 | matK_rps16 |
| Paphiopedilum_kolopakingii | p1 | (A)9 | 9 | 4178 | 4186 | matK_rps16 |
| Paphiopedilum_kolopakingii | p1 | (A)11 | 11 | 4647 | 4657 | matK_rps16 |
| Paphiopedilum_kolopakingii | p1 | (A)16 | 16 | 4987 | 5002 | matK_rps16 |
| Paphiopedilum_kolopakingii | p1 | (C)11 | 11 | 5301 | 5311 | matK_rps16 |
| Paphiopedilum_kolopakingii | p1 | (C)10 | 10 | 5708 | 5717 | rps16 intron |
| Paphiopedilum_kolopakingii | p1 | (A)8 | 8 | 5893 | 5900 | rps16 intron |
| Paphiopedilum_kolopakingii | p1 | (T)14 | 14 | 6012 | 6025 | rps16 intron |
| Paphiopedilum_kolopakingii | p1 | (T)11 | 11 | 6273 | 6283 | rps16 intron |
| Paphiopedilum_kolopakingii | p1 | (A)9 | 9 | 6826 | 6834 | rps16_trnQ-UUG |
| Paphiopedilum_kolopakingii | p1 | (T)8 | 8 | 7272 | 7279 | trnQ-UUG_psbK |
| Paphiopedilum_kolopakingii | p1 | (A)9 | 9 | 7516 | 7524 | trnQ-UUG_psbK |
| Paphiopedilum_kolopakingii | p1 | (T)8 | 8 | 7528 | 7535 | trnQ-UUG_psbK |
| Paphiopedilum_kolopakingii | p1 | (A)9 | 9 | 8803 | 8811 | trnS-GCU_trnG-UCC |
| Paphiopedilum_kolopakingii | p1 | (T)11 | 11 | 9219 | 9229 | trnS-GCU_trnG-UCC |
| Paphiopedilum_kolopakingii | p1 | (A)8 | 8 | 10248 | 10255 | trnS-GCU_trnG-UCC |
| Paphiopedilum_kolopakingii | p1 | (T)8 | 8 | 10890 | 10897 | trnG-UCC_trnR-UCU |
| Paphiopedilum_kolopakingii | p1 | (A)15 | 15 | 11151 | 11165 | trnR-UCU_atpA |
| Paphiopedilum_kolopakingii | p1 | (A)8 | 8 | 12828 | 12835 | atpA_atpF |
| Paphiopedilum_kolopakingii | p1 | (A)8 | 8 | 13027 | 13034 | atpF |
| Paphiopedilum_kolopakingii | p1 | (T)9 | 9 | 13744 | 13752 | atpF intron |
| Paphiopedilum_kolopakingii | p1 | (T)8 | 8 | 13757 | 13764 | atpF intron |
| Paphiopedilum_kolopakingii | p1 | (A)9 | 9 | 14014 | 14022 | atpF intron |
| Paphiopedilum_kolopakingii | p1 | (A)8 | 8 | 14104 | 14111 | atpF |
| Paphiopedilum_kolopakingii | p1 | (T)8 | 8 | 14307 | 14314 | atpF_atpH |
| Paphiopedilum_kolopakingii | p1 | (T)8 | 8 | 14532 | 14539 | atpF_atpH |
| Paphiopedilum_kolopakingii | p1 | (A)11 | 11 | 14822 | 14832 | atpH_atpI |
| Paphiopedilum_kolopakingii | p1 | (T)9 | 9 | 14939 | 14947 | atpH_atpI |
| Paphiopedilum_kolopakingii | p1 | (A)9 | 9 | 16414 | 16422 | atpI_rps2 |
| Paphiopedilum_kolopakingii | p1 | (T)12 | 12 | 17292 | 17303 | rps2_rpoC2 |
| Paphiopedilum_kolopakingii | p1 | (T)9 | 9 | 19329 | 19337 | rpoC2 |
| Paphiopedilum_kolopakingii | p1 | (T)8 | 8 | 19504 | 19511 | rpoC2 |
| Paphiopedilum_kolopakingii | p1 | (A)8 | 8 | 19650 | 19657 | rpoC2 |
| Paphiopedilum_kolopakingii | p1 | (A)8 | 8 | 23333 | 23340 | rpoC1 |
| Paphiopedilum_kolopakingii | p1 | (T)8 | 8 | 23642 | 23649 | rpoC1 intron |
| Paphiopedilum_kolopakingii | p1 | (T)8 | 8 | 27260 | 27267 | rpoB |
| Paphiopedilum_kolopakingii | p1 | (T)9 | 9 | 29354 | 29362 | trnC-GCA_petN |
| Paphiopedilum_kolopakingii | p1 | (T)8 | 8 | 29627 | 29634 | trnC-GCA_petN |
| Paphiopedilum_kolopakingii | p1 | (A)12 | 12 | 30069 | 30080 | petN_psbM |
| Paphiopedilum_kolopakingii | p1 | (A)14 | 14 | 30953 | 30966 | psbM_trnD-GUC |
| Paphiopedilum_kolopakingii | p1 | (T)8 | 8 | 31699 | 31706 | trnD-GUC_trnY-GUA |
| Paphiopedilum_kolopakingii | p1 | (T)8 | 8 | 32255 | 32262 | trnE-UUC_trnT-GGU |
| Paphiopedilum_kolopakingii | p1 | (A)8 | 8 | 32351 | 32358 | trnE-UUC_trnT-GGU |
| Paphiopedilum_kolopakingii | p1 | (A)9 | 9 | 33277 | 33285 | trnE-UUC_trnT-GGU |
| Paphiopedilum_kolopakingii | p1 | (T)8 | 8 | 33453 | 33460 | trnE-UUC_trnT-GGU |
| Paphiopedilum_kolopakingii | p1 | (A)11 | 11 | 33637 | 33647 | trnT-GGU_psbD |
| Paphiopedilum_kolopakingii | p1 | (T)8 | 8 | 38830 | 38837 | rps14_psaB |
| Paphiopedilum_kolopakingii | p1 | (T)10 | 10 | 44814 | 44823 | ycf3 intron 1 |
| Paphiopedilum_kolopakingii | p1 | (A)10 | 10 | 47566 | 47575 | rps4_trnT-UGU |
| Paphiopedilum_kolopakingii | p1 | (T)10 | 10 | 48048 | 48057 | trnT-UGU_trnL-UAA |
| Paphiopedilum_kolopakingii | p1 | (A)15 | 15 | 48742 | 48756 | trnT-UGU_trnL-UAA |
| Paphiopedilum_kolopakingii | p1 | (T)10 | 10 | 49752 | 49761 | trnL-UAA_trnF-GAA |
| Paphiopedilum_kolopakingii | p1 | (T)12 | 12 | 50495 | 50506 | trnF-GAA_ndhJ |
| Paphiopedilum_kolopakingii | p1 | (T)12 | 12 | 50511 | 50522 | trnF-GAA_ndhJ |
| Paphiopedilum_kolopakingii | p1 | (T)9 | 9 | 55311 | 55319 | atpB_rbcL |
| Paphiopedilum_kolopakingii | p1 | (T)18 | 18 | 55690 | 55707 | atpB_rbcL |
| Paphiopedilum_kolopakingii | p1 | (T)11 | 11 | 56596 | 56606 | atpB_rbcL |
| Paphiopedilum_kolopakingii | p1 | (T)8 | 8 | 58199 | 58206 | rbcL_accD |
| Paphiopedilum_kolopakingii | p1 | (T)9 | 9 | 58670 | 58678 | rbcL_accD |
| Paphiopedilum_kolopakingii | p1 | (A)9 | 9 | 58836 | 58844 | rbcL_accD |
| Paphiopedilum_kolopakingii | p1 | (A)10 | 10 | 59823 | 59832 | accD |
| Paphiopedilum_kolopakingii | p1 | (A)9 | 9 | 60389 | 60397 | accD |
| Paphiopedilum_kolopakingii | p1 | (T)8 | 8 | 60611 | 60618 | accD_psaI |
| Paphiopedilum_kolopakingii | p1 | (A)10 | 10 | 60710 | 60719 | accD_psaI |
| Paphiopedilum_kolopakingii | p1 | (T)8 | 8 | 61640 | 61647 | psaI_ycf4 |
| Paphiopedilum_kolopakingii | p1 | (A)8 | 8 | 62555 | 62562 | ycf4_cemA |
| Paphiopedilum_kolopakingii | p1 | (A)8 | 8 | 64027 | 64034 | petA |
| Paphiopedilum_kolopakingii | p1 | (T)8 | 8 | 64636 | 64643 | petA_psbJ |
| Paphiopedilum_kolopakingii | p1 | (T)12 | 12 | 64940 | 64951 | petA_psbJ |
| Paphiopedilum_kolopakingii | p1 | (A)9 | 9 | 66417 | 66425 | psbE_petL |
| Paphiopedilum_kolopakingii | p1 | (T)8 | 8 | 66752 | 66759 | psbE_petL |
| Paphiopedilum_kolopakingii | p1 | (A)10 | 10 | 66889 | 66898 | psbE_petL |
| Paphiopedilum_kolopakingii | p1 | (T)8 | 8 | 67082 | 67089 | psbE_petL |
| Paphiopedilum_kolopakingii | p1 | (A)12 | 12 | 67367 | 67378 | petL_petG |
| Paphiopedilum_kolopakingii | p1 | (G)8 | 8 | 67898 | 67905 | trnW-CCA_trnP-UGG |
| Paphiopedilum_kolopakingii | p1 | (T)11 | 11 | 68230 | 68240 | trnP-UGG_psaJ |
| Paphiopedilum_kolopakingii | p1 | (A)9 | 9 | 68950 | 68958 | psaJ_rpl33 |
| Paphiopedilum_kolopakingii | p1 | (T)9 | 9 | 70328 | 70336 | rps18_rpl20 |
| Paphiopedilum_kolopakingii | p1 | (T)8 | 8 | 70795 | 70802 | rpl20 |
| Paphiopedilum_kolopakingii | p1 | (T)10 | 10 | 71178 | 71187 | rpl20_rps12 |
| Paphiopedilum_kolopakingii | p1 | (T)8 | 8 | 71200 | 71207 | rpl20_rps12 |
| Paphiopedilum_kolopakingii | p1 | (T)9 | 9 | 71319 | 71327 | rpl20_rps12 |
| Paphiopedilum_kolopakingii | p1 | (T)8 | 8 | 72332 | 72339 | clpP intron 1 |
| Paphiopedilum_kolopakingii | p1 | (T)8 | 8 | 72462 | 72469 | clpP intron 1 |
| Paphiopedilum_kolopakingii | p1 | (T)15 | 15 | 72653 | 72667 | clpP intron 1 |
| Paphiopedilum_kolopakingii | p1 | (T)14 | 14 | 72888 | 72901 | clpP intron 1 |
| Paphiopedilum_kolopakingii | p1 | (T)9 | 9 | 72940 | 72948 | clpP intron 1 |
| Paphiopedilum_kolopakingii | p1 | (T)8 | 8 | 73363 | 73370 | clpP intron 2 |
| Paphiopedilum_kolopakingii | p1 | (T)14 | 14 | 73410 | 73423 | clpP intron 2 |
| Paphiopedilum_kolopakingii | p1 | (A)20 | 20 | 73478 | 73497 | clpP intron 2 |
| Paphiopedilum_kolopakingii | p1 | (T)9 | 9 | 73684 | 73692 | clpP intron 2 |
| Paphiopedilum_kolopakingii | p1 | (T)8 | 8 | 76227 | 76234 | psbB |
| Paphiopedilum_kolopakingii | p1 | (T)8 | 8 | 76877 | 76884 | psbB |
| Paphiopedilum_kolopakingii | p1 | (T)8 | 8 | 77051 | 77058 | psbB_psbT |
| Paphiopedilum_kolopakingii | p1 | (A)10 | 10 | 78191 | 78200 | psbH_petB |
| Paphiopedilum_kolopakingii | p1 | (A)14 | 14 | 78784 | 78797 | petB intron |
| Paphiopedilum_kolopakingii | p1 | (T)8 | 8 | 80185 | 80192 | petD intron |
| Paphiopedilum_kolopakingii | p1 | (A)13 | 13 | 83032 | 83044 | rps11_rpl36 |
| Paphiopedilum_kolopakingii | p1 | (T)12 | 12 | 83145 | 83156 | rps11_rpl36 |
| Paphiopedilum_kolopakingii | p1 | (T)11 | 11 | 83362 | 83372 | rpl36_infA |
| Paphiopedilum_kolopakingii | p1 | (T)9 | 9 | 83993 | 84001 | rps8 |
| Paphiopedilum_kolopakingii | p1 | (T)8 | 8 | 84260 | 84267 | rps8_rpl14 |
| Paphiopedilum_kolopakingii | p1 | (T)14 | 14 | 85024 | 85037 | rpl14_rpl16 |
| Paphiopedilum_kolopakingii | p1 | (A)9 | 9 | 85780 | 85788 | rpl16 intron |
| Paphiopedilum_kolopakingii | p1 | (T)11 | 11 | 86736 | 86746 | rpl16 intron |
| Paphiopedilum_kolopakingii | p1 | (T)8 | 8 | 86924 | 86931 | rpl16 intron |
| Paphiopedilum_kolopakingii | p1 | (T)8 | 8 | 87871 | 87878 | rpl22 |
| Paphiopedilum_kolopakingii | p1 | (T)9 | 9 | 88676 | 88684 | rps19 |
| Paphiopedilum_kolopakingii | p1 | (T)8 | 8 | 88712 | 88719 | rps19_trnH-GUG |
| Paphiopedilum_kolopakingii | p1 | (A)8 | 8 | 92981 | 92988 | ycf2 |
| Paphiopedilum_kolopakingii | p1 | (A)9 | 9 | 94273 | 94281 | ycf2 |
| Paphiopedilum_kolopakingii | p1 | (T)8 | 8 | 103125 | 103132 | rps12_trnV-GAC |
| Paphiopedilum_kolopakingii | p1 | (T)9 | 9 | 103156 | 103164 | rps12_trnV-GAC |
| Paphiopedilum_kolopakingii | p1 | (T)10 | 10 | 104806 | 104815 | rps12_trnV-GAC |
| Paphiopedilum_kolopakingii | p1 | (G)10 | 10 | 107501 | 107510 | trnI-GAU intron |
| Paphiopedilum_kolopakingii | p1 | (T)8 | 8 | 107703 | 107710 | trnI-GAU intron |
| Paphiopedilum_kolopakingii | p1 | (T)9 | 9 | 114925 | 114933 | ycf1 |
| Paphiopedilum_kolopakingii | p1 | (T)10 | 10 | 115016 | 115025 | ycf1 |
| Paphiopedilum_kolopakingii | p1 | (A)10 | 10 | 115179 | 115188 | ycf1 |
| Paphiopedilum_kolopakingii | p1 | (A)9 | 9 | 115301 | 115309 | ycf1 |
| Paphiopedilum_kolopakingii | p1 | (A)8 | 8 | 115504 | 115511 | ycf1 |
| Paphiopedilum_kolopakingii | p1 | (T)8 | 8 | 116116 | 116123 | ycf1 |
| Paphiopedilum_kolopakingii | p1 | (A)11 | 11 | 117105 | 117115 | ycf1 |
| Paphiopedilum_kolopakingii | p1 | (A)9 | 9 | 117298 | 117306 | ycf1 |
| Paphiopedilum_kolopakingii | p1 | (A)8 | 8 | 117726 | 117733 | ycf1 |
| Paphiopedilum_kolopakingii | p1 | (A)8 | 8 | 117857 | 117864 | ycf1 |
| Paphiopedilum_kolopakingii | p1 | (A)8 | 8 | 117908 | 117915 | ycf1 |
| Paphiopedilum_kolopakingii | p1 | (A)8 | 8 | 118246 | 118253 | ycf1 |
| Paphiopedilum_kolopakingii | p1 | (A)9 | 9 | 118256 | 118264 | ycf1 |
| Paphiopedilum_kolopakingii | p1 | (A)13 | 13 | 118696 | 118708 | ycf1 |
| Paphiopedilum_kolopakingii | p1 | (A)8 | 8 | 119074 | 119081 | ycf1 |
| Paphiopedilum_kolopakingii | p1 | (T)9 | 9 | 119528 | 119536 | ycf1_rps15 |
| Paphiopedilum_kolopakingii | p1 | (T)15 | 15 | 120271 | 120285 | rps15_psaC |
| Paphiopedilum_kolopakingii | p1 | (A)9 | 9 | 122955 | 122963 | ndhD_trnL-UAG |
| Paphiopedilum_kolopakingii | p1 | (T)10 | 10 | 124047 | 124056 | trnL-UAG_rpl32 |
| Paphiopedilum_kolopakingii | p1 | (A)9 | 9 | 124254 | 124262 | rpl32 |
| Paphiopedilum_kolopakingii | p1 | (T)9 | 9 | 124328 | 124336 | rpl32 |
| Paphiopedilum_kolopakingii | p1 | (T)10 | 10 | 124462 | 124471 | rpl32_ccsA |
| Paphiopedilum_kolopakingii | p1 | (T)9 | 9 | 125044 | 125052 | ccsA |
| Paphiopedilum_kolopakingii | p1 | (A)15 | 15 | 127722 | 127736 | psaC_rps15 |
| Paphiopedilum_kolopakingii | p1 | (A)9 | 9 | 128471 | 128479 | rps15_ycf1 |
| Paphiopedilum_kolopakingii | p1 | (T)8 | 8 | 128926 | 128933 | ycf1 |
| Paphiopedilum_kolopakingii | p1 | (T)13 | 13 | 129299 | 129311 | ycf1 |
| Paphiopedilum_kolopakingii | p1 | (T)9 | 9 | 129743 | 129751 | ycf1 |
| Paphiopedilum_kolopakingii | p1 | (T)8 | 8 | 129754 | 129761 | ycf1 |
| Paphiopedilum_kolopakingii | p1 | (T)8 | 8 | 130092 | 130099 | ycf1 |
| Paphiopedilum_kolopakingii | p1 | (T)8 | 8 | 130143 | 130150 | ycf1 |
| Paphiopedilum_kolopakingii | p1 | (T)8 | 8 | 130274 | 130281 | ycf1 |
| Paphiopedilum_kolopakingii | p1 | (T)9 | 9 | 130701 | 130709 | ycf1 |
| Paphiopedilum_kolopakingii | p1 | (T)11 | 11 | 130892 | 130902 | ycf1 |
| Paphiopedilum_kolopakingii | p1 | (A)8 | 8 | 131884 | 131891 | ycf1 |
| Paphiopedilum_kolopakingii | p1 | (T)8 | 8 | 132496 | 132503 | ycf1 |
| Paphiopedilum_kolopakingii | p1 | (T)9 | 9 | 132698 | 132706 | ycf1 |
| Paphiopedilum_kolopakingii | p1 | (T)10 | 10 | 132819 | 132828 | ycf1 |
| Paphiopedilum_kolopakingii | p1 | (A)10 | 10 | 132982 | 132991 | ycf1 |
| Paphiopedilum_kolopakingii | p1 | (A)9 | 9 | 133074 | 133082 | ycf1 |
| Paphiopedilum_kolopakingii | p1 | (A)8 | 8 | 140297 | 140304 | trnI-GAU intron |
| Paphiopedilum_kolopakingii | p1 | (C)10 | 10 | 140497 | 140506 | trnI-GAU intron |
| Paphiopedilum_kolopakingii | p1 | (A)10 | 10 | 143192 | 143201 | trnV-GAC_rps12 |
| Paphiopedilum_kolopakingii | p1 | (A)9 | 9 | 144843 | 144851 | trnV-GAC_rps12 |
| Paphiopedilum_kolopakingii | p1 | (A)8 | 8 | 144875 | 144882 | trnV-GAC_rps12 |
| Paphiopedilum_kolopakingii | p1 | (T)9 | 9 | 153726 | 153734 | ycf2 |
| Paphiopedilum_kolopakingii | p1 | (T)8 | 8 | 155019 | 155026 | ycf2 |
| Paphiopedilum_kolopakingii | p1 | (A)8 | 8 | 159288 | 159295 | trnH-GUG_rps19 |
| Paphiopedilum_kolopakingii | p1 | (A)9 | 9 | 159323 | 159331 | rps19 |
| Paphiopedilum_hirsutissimum | p1 | (T)9 | 9 | 1704 | 1712 | trnK-UUU intron |
| Paphiopedilum_hirsutissimum | p1 | (T)11 | 11 | 1714 | 1724 | trnK-UUU intron |
| Paphiopedilum_hirsutissimum | p1 | (T)9 | 9 | 2916 | 2924 | trnK-UUU intron |
| Paphiopedilum_hirsutissimum | p1 | (T)9 | 9 | 3522 | 3530 | trnK-UUU intron |
| Paphiopedilum_hirsutissimum | p1 | (T)8 | 8 | 4038 | 4045 | trnK-UUU intron |
| Paphiopedilum_hirsutissimum | p1 | (A)9 | 9 | 4112 | 4120 | trnK-UUU intron |
| Paphiopedilum_hirsutissimum | p1 | (A)8 | 8 | 4630 | 4637 | matK_rps16 |
| Paphiopedilum_hirsutissimum | p1 | (A)10 | 10 | 4927 | 4936 | matK_rps16 |
| Paphiopedilum_hirsutissimum | p1 | (C)8 | 8 | 5372 | 5379 | matK_rps16 |
| Paphiopedilum_hirsutissimum | p1 | (G)8 | 8 | 5384 | 5391 | matK_rps16 |
| Paphiopedilum_hirsutissimum | p1 | (A)9 | 9 | 5912 | 5920 | rps16 intron |
| Paphiopedilum_hirsutissimum | p1 | (T)11 | 11 | 6032 | 6042 | rps16 intron |
| Paphiopedilum_hirsutissimum | p1 | (T)8 | 8 | 6191 | 6198 | rps16 intron |
| Paphiopedilum_hirsutissimum | p1 | (T)10 | 10 | 6290 | 6299 | rps16 intron |
| Paphiopedilum_hirsutissimum | p1 | (A)9 | 9 | 6842 | 6850 | rps16_trnQ-UUG |
| Paphiopedilum_hirsutissimum | p1 | (A)11 | 11 | 7235 | 7245 | trnQ-UUG_psbK |
| Paphiopedilum_hirsutissimum | p1 | (A)9 | 9 | 8243 | 8251 | psbK_psbI |
| Paphiopedilum_hirsutissimum | p1 | (A)11 | 11 | 8740 | 8750 | trnS-GCU_trnG-UCC |
| Paphiopedilum_hirsutissimum | p1 | (A)8 | 8 | 9927 | 9934 | trnS-GCU_trnG-UCC |
| Paphiopedilum_hirsutissimum | p1 | (T)8 | 8 | 10569 | 10576 | trnG-UCC_trnR-UCU |
| Paphiopedilum_hirsutissimum | p1 | (A)9 | 9 | 10585 | 10593 | trnG-UCC_trnR-UCU |
| Paphiopedilum_hirsutissimum | p1 | (A)9 | 9 | 12535 | 12543 | atpA_atpF |
| Paphiopedilum_hirsutissimum | p1 | (A)8 | 8 | 12735 | 12742 | atpF |
| Paphiopedilum_hirsutissimum | p1 | (T)8 | 8 | 12802 | 12809 | atpF |
| Paphiopedilum_hirsutissimum | p1 | (T)10 | 10 | 13293 | 13302 | atpF intron |
| Paphiopedilum_hirsutissimum | p1 | (T)8 | 8 | 13327 | 13334 | atpF intron |
| Paphiopedilum_hirsutissimum | p1 | (A)8 | 8 | 13721 | 13728 | atpF intron |
| Paphiopedilum_hirsutissimum | p1 | (A)8 | 8 | 13810 | 13817 | atpF |
| Paphiopedilum_hirsutissimum | p1 | (T)9 | 9 | 14012 | 14020 | atpF_atpH |
| Paphiopedilum_hirsutissimum | p1 | (A)11 | 11 | 14390 | 14400 | atpH_atpI |
| Paphiopedilum_hirsutissimum | p1 | (T)17 | 17 | 14507 | 14523 | atpH_atpI |
| Paphiopedilum_hirsutissimum | p1 | (A)12 | 12 | 15974 | 15985 | atpI_rps2 |
| Paphiopedilum_hirsutissimum | p1 | (T)11 | 11 | 16855 | 16865 | rps2 |
| Paphiopedilum_hirsutissimum | p1 | (A)8 | 8 | 17112 | 17119 | rps2_rpoC2 |
| Paphiopedilum_hirsutissimum | p1 | (T)9 | 9 | 18897 | 18905 | rpoC2 |
| Paphiopedilum_hirsutissimum | p1 | (T)8 | 8 | 19072 | 19079 | rpoC2 |
| Paphiopedilum_hirsutissimum | p1 | (A)8 | 8 | 19218 | 19225 | rpoC2 |
| Paphiopedilum_hirsutissimum | p1 | (A)8 | 8 | 22901 | 22908 | rpoC1 |
| Paphiopedilum_hirsutissimum | p1 | (C)9 | 9 | 23035 | 23043 | rpoC1 intron |
| Paphiopedilum_hirsutissimum | p1 | (T)10 | 10 | 23212 | 23221 | rpoC1 intron |
| Paphiopedilum_hirsutissimum | p1 | (T)8 | 8 | 26832 | 26839 | rpoB |
| Paphiopedilum_hirsutissimum | p1 | (T)9 | 9 | 28959 | 28967 | trnC-GCA_petN |
| Paphiopedilum_hirsutissimum | p1 | (T)8 | 8 | 29041 | 29048 | trnC-GCA_petN |
| Paphiopedilum_hirsutissimum | p1 | (A)9 | 9 | 29486 | 29494 | petN_psbM |
| Paphiopedilum_hirsutissimum | p1 | (A)9 | 9 | 30170 | 30178 | psbM_trnD-GUC |
| Paphiopedilum_hirsutissimum | p1 | (T)9 | 9 | 30911 | 30919 | trnD-GUC_trnY-GUA |
| Paphiopedilum_hirsutissimum | p1 | (T)10 | 10 | 31831 | 31840 | trnE-UUC_trnT-GGU |
| Paphiopedilum_hirsutissimum | p1 | (A)12 | 12 | 32183 | 32194 | trnT-GGU_psbD |
| Paphiopedilum_hirsutissimum | p1 | (T)15 | 15 | 37389 | 37403 | rps14_psaB |
| Paphiopedilum_hirsutissimum | p1 | (A)8 | 8 | 42555 | 42562 | psaA_ycf3 |
| Paphiopedilum_hirsutissimum | p1 | (A)13 | 13 | 44363 | 44375 | ycf3 intron 2 |
| Paphiopedilum_hirsutissimum | p1 | (A)10 | 10 | 46140 | 46149 | rps4_trnT-UGU |
| Paphiopedilum_hirsutissimum | p1 | (T)16 | 16 | 46623 | 46638 | trnT-UGU_trnL-UAA |
| Paphiopedilum_hirsutissimum | p1 | (A)19 | 19 | 46801 | 46819 | trnT-UGU_trnL-UAA |
| Paphiopedilum_hirsutissimum | p1 | (T)11 | 11 | 47828 | 47838 | trnL-UAA_trnF-GAA |
| Paphiopedilum_hirsutissimum | p1 | (T)9 | 9 | 48564 | 48572 | trnF-GAA_ndhK |
| Paphiopedilum_hirsutissimum | p1 | (T)9 | 9 | 48577 | 48585 | trnF-GAA_ndhK |
| Paphiopedilum_hirsutissimum | p1 | (T)9 | 9 | 52792 | 52800 | atpB_rbcL |
| Paphiopedilum_hirsutissimum | p1 | (T)9 | 9 | 53156 | 53164 | atpB_rbcL |
| Paphiopedilum_hirsutissimum | p1 | (T)10 | 10 | 53869 | 53878 | atpB_rbcL |
| Paphiopedilum_hirsutissimum | p1 | (A)8 | 8 | 55478 | 55485 | rbcL_accD |
| Paphiopedilum_hirsutissimum | p1 | (T)8 | 8 | 55952 | 55959 | rbcL_accD |
| Paphiopedilum_hirsutissimum | p1 | (A)13 | 13 | 56117 | 56129 | rbcL_accD |
| Paphiopedilum_hirsutissimum | p1 | (A)10 | 10 | 57108 | 57117 | accD |
| Paphiopedilum_hirsutissimum | p1 | (A)8 | 8 | 57674 | 57681 | accD |
| Paphiopedilum_hirsutissimum | p1 | (A)8 | 8 | 57983 | 57990 | accD_psaI |
| Paphiopedilum_hirsutissimum | p1 | (A)8 | 8 | 58055 | 58062 | accD_psaI |
| Paphiopedilum_hirsutissimum | p1 | (A)12 | 12 | 59837 | 59848 | ycf4_cemA |
| Paphiopedilum_hirsutissimum | p1 | (A)8 | 8 | 61198 | 61205 | petA |
| Paphiopedilum_hirsutissimum | p1 | (T)8 | 8 | 61807 | 61814 | petA_psbJ |
| Paphiopedilum_hirsutissimum | p1 | (T)8 | 8 | 62111 | 62118 | petA_psbJ |
| Paphiopedilum_hirsutissimum | p1 | (C)8 | 8 | 62897 | 62904 | psbJ_psbL |
| Paphiopedilum_hirsutissimum | p1 | (G)9 | 9 | 63580 | 63588 | psbE_petL |
| Paphiopedilum_hirsutissimum | p1 | (T)8 | 8 | 63918 | 63925 | psbE_petL |
| Paphiopedilum_hirsutissimum | p1 | (A)10 | 10 | 64055 | 64064 | psbE_petL |
| Paphiopedilum_hirsutissimum | p1 | (T)8 | 8 | 64248 | 64255 | psbE_petL |
| Paphiopedilum_hirsutissimum | p1 | (A)10 | 10 | 64533 | 64542 | petL_petG |
| Paphiopedilum_hirsutissimum | p1 | (T)23 | 23 | 65374 | 65396 | trnP-UGG_psaJ |
| Paphiopedilum_hirsutissimum | p1 | (A)8 | 8 | 66791 | 66798 | rpl33_rps18 |
| Paphiopedilum_hirsutissimum | p1 | (T)11 | 11 | 67382 | 67392 | rps18_rpl20 |
| Paphiopedilum_hirsutissimum | p1 | (T)8 | 8 | 67851 | 67858 | rpl20 |
| Paphiopedilum_hirsutissimum | p1 | (T)10 | 10 | 68253 | 68262 | rpl20_rps12 |
| Paphiopedilum_hirsutissimum | p1 | (T)9 | 9 | 68374 | 68382 | rpl20_rps12 |
| Paphiopedilum_hirsutissimum | p1 | (T)9 | 9 | 68653 | 68661 | rpl20_rps12 |
| Paphiopedilum_hirsutissimum | p1 | (T)10 | 10 | 69644 | 69653 | clpP intron 1 |
| Paphiopedilum_hirsutissimum | p1 | (T)9 | 9 | 69688 | 69696 | clpP intron 1 |
| Paphiopedilum_hirsutissimum | p1 | (T)14 | 14 | 69941 | 69954 | clpP intron 1 |
| Paphiopedilum_hirsutissimum | p1 | (T)9 | 9 | 70000 | 70008 | clpP intron 1 |
| Paphiopedilum_hirsutissimum | p1 | (T)10 | 10 | 70423 | 70432 | clpP intron 2 |
| Paphiopedilum_hirsutissimum | p1 | (T)10 | 10 | 70472 | 70481 | clpP intron 2 |
| Paphiopedilum_hirsutissimum | p1 | (T)10 | 10 | 70720 | 70729 | clpP intron 2 |
| Paphiopedilum_hirsutissimum | p1 | (T)8 | 8 | 72615 | 72622 | psbB |
| Paphiopedilum_hirsutissimum | p1 | (T)8 | 8 | 73265 | 73272 | psbB |
| Paphiopedilum_hirsutissimum | p1 | (T)10 | 10 | 73750 | 73759 | psbB_psbT |
| Paphiopedilum_hirsutissimum | p1 | (A)10 | 10 | 75102 | 75111 | petB intron |
| Paphiopedilum_hirsutissimum | p1 | (T)8 | 8 | 76499 | 76506 | petD intron |
| Paphiopedilum_hirsutissimum | p1 | (A)10 | 10 | 79374 | 79383 | ndhE |
| Paphiopedilum_hirsutissimum | p1 | (T)10 | 10 | 79687 | 79696 | rpl36_infA |
| Paphiopedilum_hirsutissimum | p1 | (T)8 | 8 | 80323 | 80330 | rps8 |
| Paphiopedilum_hirsutissimum | p1 | (T)12 | 12 | 81358 | 81369 | rpl14_rpl16 |
| Paphiopedilum_hirsutissimum | p1 | (T)9 | 9 | 82923 | 82931 | rpl16 intron |
| Paphiopedilum_hirsutissimum | p1 | (T)9 | 9 | 82949 | 82957 | rpl16 intron |
| Paphiopedilum_hirsutissimum | p1 | (T)8 | 8 | 83108 | 83115 | rpl16 intron |
| Paphiopedilum_hirsutissimum | p1 | (T)8 | 8 | 84080 | 84087 | rpl22 |
| Paphiopedilum_hirsutissimum | p1 | (T)9 | 9 | 84927 | 84935 | rps19 |
| Paphiopedilum_hirsutissimum | p1 | (T)8 | 8 | 84963 | 84970 | rps19_trnH-GUG |
| Paphiopedilum_hirsutissimum | p1 | (A)8 | 8 | 89103 | 89110 | ycf2 |
| Paphiopedilum_hirsutissimum | p1 | (A)9 | 9 | 90386 | 90394 | ycf2 |
| Paphiopedilum_hirsutissimum | p1 | (T)9 | 9 | 99125 | 99133 | rps12_ycf15 |
| Paphiopedilum_hirsutissimum | p1 | (T)9 | 9 | 99157 | 99165 | rps12_ycf15 |
| Paphiopedilum_hirsutissimum | p1 | (T)11 | 11 | 100482 | 100492 | ycf15_trnV-GAC |
| Paphiopedilum_hirsutissimum | p1 | (G)9 | 9 | 103178 | 103186 | trnI-GAU intron |
| Paphiopedilum_hirsutissimum | p1 | (T)8 | 8 | 103379 | 103386 | trnI-GAU intron |
| Paphiopedilum_hirsutissimum | p1 | (T)9 | 9 | 110553 | 110561 | ycf1 |
| Paphiopedilum_hirsutissimum | p1 | (T)10 | 10 | 110644 | 110653 | ycf1 |
| Paphiopedilum_hirsutissimum | p1 | (A)10 | 10 | 110807 | 110816 | ycf1 |
| Paphiopedilum_hirsutissimum | p1 | (A)8 | 8 | 110930 | 110937 | ycf1 |
| Paphiopedilum_hirsutissimum | p1 | (A)8 | 8 | 111132 | 111139 | ycf1 |
| Paphiopedilum_hirsutissimum | p1 | (A)10 | 10 | 111296 | 111305 | ycf1 |
| Paphiopedilum_hirsutissimum | p1 | (T)8 | 8 | 111834 | 111841 | ycf1 |
| Paphiopedilum_hirsutissimum | p1 | (A)11 | 11 | 112823 | 112833 | ycf1 |
| Paphiopedilum_hirsutissimum | p1 | (A)9 | 9 | 113016 | 113024 | ycf1 |
| Paphiopedilum_hirsutissimum | p1 | (A)8 | 8 | 113444 | 113451 | ycf1 |
| Paphiopedilum_hirsutissimum | p1 | (A)8 | 8 | 113575 | 113582 | ycf1 |
| Paphiopedilum_hirsutissimum | p1 | (A)8 | 8 | 113626 | 113633 | ycf1 |
| Paphiopedilum_hirsutissimum | p1 | (A)8 | 8 | 113964 | 113971 | ycf1 |
| Paphiopedilum_hirsutissimum | p1 | (A)9 | 9 | 113974 | 113982 | ycf1 |
| Paphiopedilum_hirsutissimum | p1 | (A)8 | 8 | 114383 | 114390 | ycf1 |
| Paphiopedilum_hirsutissimum | p1 | (A)8 | 8 | 114413 | 114420 | ycf1 |
| Paphiopedilum_hirsutissimum | p1 | (A)8 | 8 | 114443 | 114450 | ycf1 |
| Paphiopedilum_hirsutissimum | p1 | (A)8 | 8 | 114816 | 114823 | ycf1 |
| Paphiopedilum_hirsutissimum | p1 | (T)8 | 8 | 115270 | 115277 | ycf1_rps15 |
| Paphiopedilum_hirsutissimum | p1 | (T)15 | 15 | 115927 | 115941 | rps15_psaC |
| Paphiopedilum_hirsutissimum | p1 | (A)10 | 10 | 118715 | 118724 | ccsA |
| Paphiopedilum_hirsutissimum | p1 | (A)12 | 12 | 119297 | 119308 | ccsA_rpl32 |
| Paphiopedilum_hirsutissimum | p1 | (T)9 | 9 | 119504 | 119512 | rpl32 |
| Paphiopedilum_hirsutissimum | p1 | (A)9 | 9 | 120451 | 120459 | rpl32 |
| Paphiopedilum_hirsutissimum | p1 | (T)12 | 12 | 120655 | 120666 | rpl32_ccsA |
| Paphiopedilum_hirsutissimum | p1 | (T)10 | 10 | 121239 | 121248 | ccsA |
| Paphiopedilum_hirsutissimum | p1 | (A)15 | 15 | 124022 | 124036 | psaC_rps15 |
| Paphiopedilum_hirsutissimum | p1 | (A)8 | 8 | 124686 | 124693 | rps15_ycf1 |
| Paphiopedilum_hirsutissimum | p1 | (T)8 | 8 | 125140 | 125147 | ycf1 |
| Paphiopedilum_hirsutissimum | p1 | (T)8 | 8 | 125513 | 125520 | ycf1 |
| Paphiopedilum_hirsutissimum | p1 | (T)8 | 8 | 125543 | 125550 | ycf1 |
| Paphiopedilum_hirsutissimum | p1 | (T)8 | 8 | 125573 | 125580 | ycf1 |
| Paphiopedilum_hirsutissimum | p1 | (T)9 | 9 | 125981 | 125989 | ycf1 |
| Paphiopedilum_hirsutissimum | p1 | (T)8 | 8 | 125992 | 125999 | ycf1 |
| Paphiopedilum_hirsutissimum | p1 | (T)8 | 8 | 126330 | 126337 | ycf1 |
| Paphiopedilum_hirsutissimum | p1 | (T)8 | 8 | 126381 | 126388 | ycf1 |
| Paphiopedilum_hirsutissimum | p1 | (T)8 | 8 | 126512 | 126519 | ycf1 |
| Paphiopedilum_hirsutissimum | p1 | (T)9 | 9 | 126939 | 126947 | ycf1 |
| Paphiopedilum_hirsutissimum | p1 | (T)11 | 11 | 127130 | 127140 | ycf1 |
| Paphiopedilum_hirsutissimum | p1 | (A)8 | 8 | 128122 | 128129 | ycf1 |
| Paphiopedilum_hirsutissimum | p1 | (T)10 | 10 | 128664 | 128673 | ycf1 |
| Paphiopedilum_hirsutissimum | p1 | (T)8 | 8 | 128830 | 128837 | ycf1 |
| Paphiopedilum_hirsutissimum | p1 | (T)8 | 8 | 129032 | 129039 | ycf1 |
| Paphiopedilum_hirsutissimum | p1 | (T)10 | 10 | 129153 | 129162 | ycf1 |
| Paphiopedilum_hirsutissimum | p1 | (A)10 | 10 | 129316 | 129325 | ycf1 |
| Paphiopedilum_hirsutissimum | p1 | (A)9 | 9 | 129408 | 129416 | ycf1 |
| Paphiopedilum_hirsutissimum | p1 | (A)8 | 8 | 136583 | 136590 | trnI-GAU intron |
| Paphiopedilum_hirsutissimum | p1 | (C)9 | 9 | 136783 | 136791 | trnI-GAU intron |
| Paphiopedilum_hirsutissimum | p1 | (A)11 | 11 | 139477 | 139487 | trnV-GAC_ycf15 |
| Paphiopedilum_hirsutissimum | p1 | (A)9 | 9 | 140804 | 140812 | ycf15_rps12 |
| Paphiopedilum_hirsutissimum | p1 | (A)9 | 9 | 140836 | 140844 | ycf15_rps12 |
| Paphiopedilum_hirsutissimum | p1 | (T)9 | 9 | 149575 | 149583 | ycf2 |
| Paphiopedilum_hirsutissimum | p1 | (T)8 | 8 | 150859 | 150866 | ycf2 |
| Paphiopedilum_philippinense | p1 | (T)8 | 8 | 1728 | 1735 | trnK-UUU intron |
| Paphiopedilum_philippinense | p1 | (T)9 | 9 | 2921 | 2929 | trnK-UUU intron |
| Paphiopedilum_philippinense | p1 | (A)8 | 8 | 3029 | 3036 | trnK-UUU intron |
| Paphiopedilum_philippinense | p1 | (T)12 | 12 | 3563 | 3574 | trnK-UUU intron |
| Paphiopedilum_philippinense | p1 | (T)8 | 8 | 4112 | 4119 | trnK-UUU intron |
| Paphiopedilum_philippinense | p1 | (A)10 | 10 | 4186 | 4195 | trnK-UUU intron |
| Paphiopedilum_philippinense | p1 | (A)8 | 8 | 4656 | 4663 | matK_rps16 |
| Paphiopedilum_philippinense | p1 | (A)10 | 10 | 5003 | 5012 | matK_rps16 |
| Paphiopedilum_philippinense | p1 | (A)9 | 9 | 5197 | 5205 | matK_rps16 |
| Paphiopedilum_philippinense | p1 | (C)9 | 9 | 5284 | 5292 | matK_rps16 |
| Paphiopedilum_philippinense | p1 | (C)10 | 10 | 5689 | 5698 | rps16 intron |
| Paphiopedilum_philippinense | p1 | (A)8 | 8 | 5826 | 5833 | rps16 intron |
| Paphiopedilum_philippinense | p1 | (T)15 | 15 | 5944 | 5958 | rps16 intron |
| Paphiopedilum_philippinense | p1 | (T)9 | 9 | 6206 | 6214 | rps16 intron |
| Paphiopedilum_philippinense | p1 | (A)9 | 9 | 6757 | 6765 | rps16_trnQ-UUG |
| Paphiopedilum_philippinense | p1 | (T)9 | 9 | 7184 | 7192 | trnQ-UUG_psbK |
| Paphiopedilum_philippinense | p1 | (A)9 | 9 | 7429 | 7437 | trnQ-UUG_psbK |
| Paphiopedilum_philippinense | p1 | (T)10 | 10 | 7441 | 7450 | trnQ-UUG_psbK |
| Paphiopedilum_philippinense | p1 | (A)9 | 9 | 8696 | 8704 | trnS-GCU_trnG-UCC |
| Paphiopedilum_philippinense | p1 | (A)10 | 10 | 10002 | 10011 | trnG-UCC intron |
| Paphiopedilum_philippinense | p1 | (T)8 | 8 | 10646 | 10653 | trnG-UCC_trnR-UCU |
| Paphiopedilum_philippinense | p1 | (A)9 | 9 | 10878 | 10886 | trnR-UCU_atpA |
| Paphiopedilum_philippinense | p1 | (A)8 | 8 | 12562 | 12569 | atpA_atpF |
| Paphiopedilum_philippinense | p1 | (A)8 | 8 | 12761 | 12768 | atpF |
| Paphiopedilum_philippinense | p1 | (T)8 | 8 | 13492 | 13499 | atpF intron |
| Paphiopedilum_philippinense | p1 | (T)10 | 10 | 13504 | 13513 | atpF intron |
| Paphiopedilum_philippinense | p1 | (T)18 | 18 | 13515 | 13532 | atpF intron |
| Paphiopedilum_philippinense | p1 | (A)8 | 8 | 13770 | 13777 | atpF intron |
| Paphiopedilum_philippinense | p1 | (A)8 | 8 | 13859 | 13866 | atpF |
| Paphiopedilum_philippinense | p1 | (T)8 | 8 | 14065 | 14072 | atpF_atpH |
| Paphiopedilum_philippinense | p1 | (T)8 | 8 | 14290 | 14297 | atpF_atpH |
| Paphiopedilum_philippinense | p1 | (A)10 | 10 | 14580 | 14589 | atpH_atpI |
| Paphiopedilum_philippinense | p1 | (T)8 | 8 | 14696 | 14703 | atpH_atpI |
| Paphiopedilum_philippinense | p1 | (A)9 | 9 | 16170 | 16178 | atpI_rps2 |
| Paphiopedilum_philippinense | p1 | (T)11 | 11 | 17048 | 17058 | rps2 |
| Paphiopedilum_philippinense | p1 | (T)9 | 9 | 19084 | 19092 | rpoC2 |
| Paphiopedilum_philippinense | p1 | (T)8 | 8 | 19259 | 19266 | rpoC2 |
| Paphiopedilum_philippinense | p1 | (A)8 | 8 | 19405 | 19412 | rpoC2 |
| Paphiopedilum_philippinense | p1 | (T)8 | 8 | 19452 | 19459 | rpoC2 |
| Paphiopedilum_philippinense | p1 | (A)8 | 8 | 23088 | 23095 | rpoC1 |
| Paphiopedilum_philippinense | p1 | (T)10 | 10 | 23397 | 23406 | rpoC1 intron |
| Paphiopedilum_philippinense | p1 | (T)8 | 8 | 27017 | 27024 | rpoB |
| Paphiopedilum_philippinense | p1 | (T)13 | 13 | 28982 | 28994 | trnC-GCA_petN |
| Paphiopedilum_philippinense | p1 | (A)10 | 10 | 29382 | 29391 | petN_psbM |
| Paphiopedilum_philippinense | p1 | (A)8 | 8 | 30241 | 30248 | psbM_trnD-GUC |
| Paphiopedilum_philippinense | p1 | (T)8 | 8 | 30981 | 30988 | trnD-GUC_trnY-GUA |
| Paphiopedilum_philippinense | p1 | (T)8 | 8 | 31529 | 31536 | trnE-UUC_trnT-GGU |
| Paphiopedilum_philippinense | p1 | (A)8 | 8 | 32427 | 32434 | trnE-UUC_trnT-GGU |
| Paphiopedilum_philippinense | p1 | (T)8 | 8 | 32604 | 32611 | trnE-UUC_trnT-GGU |
| Paphiopedilum_philippinense | p1 | (A)14 | 14 | 32788 | 32801 | trnT-GGU_psbD |
| Paphiopedilum_philippinense | p1 | (T)11 | 11 | 33523 | 33533 | trnT-GGU_psbD |
| Paphiopedilum_philippinense | p1 | (T)9 | 9 | 38002 | 38010 | rps14_psaB |
| Paphiopedilum_philippinense | p1 | (T)10 | 10 | 43998 | 44007 | ycf3 intron 1 |
| Paphiopedilum_philippinense | p1 | (T)9 | 9 | 44936 | 44944 | ycf3 intron 2 |
| Paphiopedilum_philippinense | p1 | (A)11 | 11 | 46738 | 46748 | rps4_trnT-UGU |
| Paphiopedilum_philippinense | p1 | (T)12 | 12 | 47221 | 47232 | trnT-UGU_trnL-UAA |
| Paphiopedilum_philippinense | p1 | (A)19 | 19 | 47897 | 47915 | trnT-UGU_trnL-UAA |
| Paphiopedilum_philippinense | p1 | (T)14 | 14 | 48911 | 48924 | trnL-UAA_trnF-GAA |
| Paphiopedilum_philippinense | p1 | (T)11 | 11 | 49632 | 49642 | trnF-GAA_ndhJ |
| Paphiopedilum_philippinense | p1 | (T)10 | 10 | 49647 | 49656 | trnF-GAA_ndhJ |
| Paphiopedilum_philippinense | p1 | (T)9 | 9 | 49817 | 49825 | trnF-GAA_ndhJ |
| Paphiopedilum_philippinense | p1 | (A)9 | 9 | 50620 | 50628 | ndhK |
| Paphiopedilum_philippinense | p1 | (T)8 | 8 | 54434 | 54441 | atpB_rbcL |
| Paphiopedilum_philippinense | p1 | (T)10 | 10 | 54812 | 54821 | atpB_rbcL |
| Paphiopedilum_philippinense | p1 | (T)11 | 11 | 55367 | 55377 | atpB_rbcL |
| Paphiopedilum_philippinense | p1 | (T)8 | 8 | 56970 | 56977 | rbcL_accD |
| Paphiopedilum_philippinense | p1 | (T)8 | 8 | 57414 | 57421 | rbcL_accD |
| Paphiopedilum_philippinense | p1 | (A)15 | 15 | 57579 | 57593 | rbcL_accD |
| Paphiopedilum_philippinense | p1 | (A)10 | 10 | 58581 | 58590 | accD |
| Paphiopedilum_philippinense | p1 | (A)9 | 9 | 59147 | 59155 | accD |
| Paphiopedilum_philippinense | p1 | (T)8 | 8 | 59369 | 59376 | accD_psaI |
| Paphiopedilum_philippinense | p1 | (C)8 | 8 | 59383 | 59390 | accD_psaI |
| Paphiopedilum_philippinense | p1 | (A)16 | 16 | 59469 | 59484 | accD_psaI |
| Paphiopedilum_philippinense | p1 | (T)9 | 9 | 60399 | 60407 | psaI_ycf4 |
| Paphiopedilum_philippinense | p1 | (A)8 | 8 | 61315 | 61322 | ycf4_cemA |
| Paphiopedilum_philippinense | p1 | (A)8 | 8 | 62823 | 62830 | petA |
| Paphiopedilum_philippinense | p1 | (T)8 | 8 | 63432 | 63439 | petA_psbJ |
| Paphiopedilum_philippinense | p1 | (T)12 | 12 | 63736 | 63747 | petA_psbJ |
| Paphiopedilum_philippinense | p1 | (A)8 | 8 | 64020 | 64027 | petA_psbJ |
| Paphiopedilum_philippinense | p1 | (G)8 | 8 | 65148 | 65155 | psbE_petL |
| Paphiopedilum_philippinense | p1 | (T)8 | 8 | 65489 | 65496 | psbE_petL |
| Paphiopedilum_philippinense | p1 | (T)8 | 8 | 65723 | 65730 | psbE_petL |
| Paphiopedilum_philippinense | p1 | (A)12 | 12 | 66007 | 66018 | petL_petG |
| Paphiopedilum_philippinense | p1 | (T)10 | 10 | 66876 | 66885 | trnP-UGG_psaJ |
| Paphiopedilum_philippinense | p1 | (A)8 | 8 | 67034 | 67041 | trnP-UGG_psaJ |
| Paphiopedilum_philippinense | p1 | (A)12 | 12 | 67552 | 67563 | psaJ_rpl33 |
| Paphiopedilum_philippinense | p1 | (T)10 | 10 | 68903 | 68912 | rps18_rpl20 |
| Paphiopedilum_philippinense | p1 | (T)8 | 8 | 69372 | 69379 | rpl20 |
| Paphiopedilum_philippinense | p1 | (T)11 | 11 | 69775 | 69785 | rpl20_rps12 |
| Paphiopedilum_philippinense | p1 | (T)8 | 8 | 70932 | 70939 | clpP intron 1 |
| Paphiopedilum_philippinense | p1 | (T)12 | 12 | 71303 | 71314 | clpP intron 1 |
| Paphiopedilum_philippinense | p1 | (T)16 | 16 | 71535 | 71550 | clpP intron 1 |
| Paphiopedilum_philippinense | p1 | (T)9 | 9 | 71593 | 71601 | clpP intron 1 |
| Paphiopedilum_philippinense | p1 | (T)12 | 12 | 72016 | 72027 | clpP intron 2 |
| Paphiopedilum_philippinense | p1 | (T)10 | 10 | 72067 | 72076 | clpP intron 2 |
| Paphiopedilum_philippinense | p1 | (A)12 | 12 | 72131 | 72142 | clpP intron 2 |
| Paphiopedilum_philippinense | p1 | (T)10 | 10 | 72329 | 72338 | clpP intron 2 |
| Paphiopedilum_philippinense | p1 | (T)8 | 8 | 74794 | 74801 | psbB |
| Paphiopedilum_philippinense | p1 | (T)8 | 8 | 75444 | 75451 | psbB |
| Paphiopedilum_philippinense | p1 | (T)8 | 8 | 75733 | 75740 | psbB_psbT |
| Paphiopedilum_philippinense | p1 | (A)14 | 14 | 77371 | 77384 | petB intron |
| Paphiopedilum_philippinense | p1 | (T)8 | 8 | 78772 | 78779 | petD intron |
| Paphiopedilum_philippinense | p1 | (A)9 | 9 | 81619 | 81627 | rps11_rpl36 |
| Paphiopedilum_philippinense | p1 | (T)9 | 9 | 81721 | 81729 | rps11_rpl36 |
| Paphiopedilum_philippinense | p1 | (T)9 | 9 | 82562 | 82570 | rps8 |
| Paphiopedilum_philippinense | p1 | (T)8 | 8 | 82829 | 82836 | rps8_rpl14 |
| Paphiopedilum_philippinense | p1 | (T)12 | 12 | 83635 | 83646 | rpl14_rpl16 |
| Paphiopedilum_philippinense | p1 | (T)13 | 13 | 83648 | 83660 | rpl14_rpl16 |
| Paphiopedilum_philippinense | p1 | (A)10 | 10 | 84403 | 84412 | rpl16 intron |
| Paphiopedilum_philippinense | p1 | (T)9 | 9 | 84890 | 84898 | rpl16 intron |
| Paphiopedilum_philippinense | p1 | (T)10 | 10 | 85387 | 85396 | rpl16 intron |
| Paphiopedilum_philippinense | p1 | (A)8 | 8 | 85427 | 85434 | rpl16 intron |
| Paphiopedilum_philippinense | p1 | (T)8 | 8 | 85575 | 85582 | rpl16 intron |
| Paphiopedilum_philippinense | p1 | (T)8 | 8 | 86548 | 86555 | rpl22 |
| Paphiopedilum_philippinense | p1 | (T)9 | 9 | 87353 | 87361 | rps19 |
| Paphiopedilum_philippinense | p1 | (T)8 | 8 | 87389 | 87396 | rps19_trnH-GUG |
| Paphiopedilum_philippinense | p1 | (A)8 | 8 | 91658 | 91665 | ycf2 |
| Paphiopedilum_philippinense | p1 | (A)9 | 9 | 92950 | 92958 | ycf2 |
| Paphiopedilum_philippinense | p1 | (T)8 | 8 | 101833 | 101840 | rps12_trnV-GAC |
| Paphiopedilum_philippinense | p1 | (T)8 | 8 | 101864 | 101871 | rps12_trnV-GAC |
| Paphiopedilum_philippinense | p1 | (T)10 | 10 | 103405 | 103414 | rps12_trnV-GAC |
| Paphiopedilum_philippinense | p1 | (G)9 | 9 | 106099 | 106107 | trnI-GAU intron |
| Paphiopedilum_philippinense | p1 | (T)9 | 9 | 106300 | 106308 | trnI-GAU intron |
| Paphiopedilum_philippinense | p1 | (T)9 | 9 | 113586 | 113594 | ycf1 |
| Paphiopedilum_philippinense | p1 | (T)10 | 10 | 113677 | 113686 | ycf1 |
| Paphiopedilum_philippinense | p1 | (A)10 | 10 | 113840 | 113849 | ycf1 |
| Paphiopedilum_philippinense | p1 | (A)9 | 9 | 113962 | 113970 | ycf1 |
| Paphiopedilum_philippinense | p1 | (A)8 | 8 | 114165 | 114172 | ycf1 |
| Paphiopedilum_philippinense | p1 | (T)8 | 8 | 114783 | 114790 | ycf1 |
| Paphiopedilum_philippinense | p1 | (A)11 | 11 | 115772 | 115782 | ycf1 |
| Paphiopedilum_philippinense | p1 | (A)9 | 9 | 115965 | 115973 | ycf1 |
| Paphiopedilum_philippinense | p1 | (A)8 | 8 | 116393 | 116400 | ycf1 |
| Paphiopedilum_philippinense | p1 | (A)8 | 8 | 116524 | 116531 | ycf1 |
| Paphiopedilum_philippinense | p1 | (A)8 | 8 | 116575 | 116582 | ycf1 |
| Paphiopedilum_philippinense | p1 | (A)8 | 8 | 116913 | 116920 | ycf1 |
| Paphiopedilum_philippinense | p1 | (A)9 | 9 | 116923 | 116931 | ycf1 |
| Paphiopedilum_philippinense | p1 | (A)13 | 13 | 117363 | 117375 | ycf1 |
| Paphiopedilum_philippinense | p1 | (A)8 | 8 | 117741 | 117748 | ycf1 |
| Paphiopedilum_philippinense | p1 | (T)9 | 9 | 118195 | 118203 | ycf1_rps15 |
| Paphiopedilum_philippinense | p1 | (T)18 | 18 | 118938 | 118955 | rps15_psaC |
| Paphiopedilum_philippinense | p1 | (A)9 | 9 | 121651 | 121659 | ndhD_trnL-UAG |
| Paphiopedilum_philippinense | p1 | (T)9 | 9 | 122816 | 122824 | trnL-UAG_rpl32 |
| Paphiopedilum_philippinense | p1 | (A)9 | 9 | 123028 | 123036 | rpl32 |
| Paphiopedilum_philippinense | p1 | (T)9 | 9 | 123102 | 123110 | rpl32 |
| Paphiopedilum_philippinense | p1 | (T)25 | 25 | 123236 | 123260 | rpl32_ccsA |
| Paphiopedilum_philippinense | p1 | (T)9 | 9 | 123832 | 123840 | ccsA |
| Paphiopedilum_philippinense | p1 | (A)18 | 18 | 126536 | 126553 | psaC_rps15 |
| Paphiopedilum_philippinense | p1 | (A)9 | 9 | 127288 | 127296 | rps15_ycf1 |
| Paphiopedilum_philippinense | p1 | (T)8 | 8 | 127743 | 127750 | ycf1 |
| Paphiopedilum_philippinense | p1 | (T)13 | 13 | 128116 | 128128 | ycf1 |
| Paphiopedilum_philippinense | p1 | (T)9 | 9 | 128560 | 128568 | ycf1 |
| Paphiopedilum_philippinense | p1 | (T)8 | 8 | 128571 | 128578 | ycf1 |
| Paphiopedilum_philippinense | p1 | (T)8 | 8 | 128909 | 128916 | ycf1 |
| Paphiopedilum_philippinense | p1 | (T)8 | 8 | 128960 | 128967 | ycf1 |
| Paphiopedilum_philippinense | p1 | (T)8 | 8 | 129091 | 129098 | ycf1 |
| Paphiopedilum_philippinense | p1 | (T)9 | 9 | 129518 | 129526 | ycf1 |
| Paphiopedilum_philippinense | p1 | (T)11 | 11 | 129709 | 129719 | ycf1 |
| Paphiopedilum_philippinense | p1 | (A)8 | 8 | 130701 | 130708 | ycf1 |
| Paphiopedilum_philippinense | p1 | (T)8 | 8 | 131319 | 131326 | ycf1 |
| Paphiopedilum_philippinense | p1 | (T)9 | 9 | 131521 | 131529 | ycf1 |
| Paphiopedilum_philippinense | p1 | (T)10 | 10 | 131642 | 131651 | ycf1 |
| Paphiopedilum_philippinense | p1 | (A)10 | 10 | 131805 | 131814 | ycf1 |
| Paphiopedilum_philippinense | p1 | (A)9 | 9 | 131897 | 131905 | ycf1 |
| Paphiopedilum_philippinense | p1 | (A)9 | 9 | 139183 | 139191 | trnI-GAU intron |
| Paphiopedilum_philippinense | p1 | (C)9 | 9 | 139384 | 139392 | trnI-GAU intron |
| Paphiopedilum_philippinense | p1 | (A)10 | 10 | 142077 | 142086 | trnV-GAC_rps12 |
| Paphiopedilum_philippinense | p1 | (A)8 | 8 | 143620 | 143627 | trnV-GAC_rps12 |
| Paphiopedilum_philippinense | p1 | (A)8 | 8 | 143651 | 143658 | trnV-GAC_rps12 |
| Paphiopedilum_philippinense | p1 | (T)9 | 9 | 152533 | 152541 | ycf2 |
| Paphiopedilum_philippinense | p1 | (T)8 | 8 | 153826 | 153833 | ycf2 |
| Paphiopedilum_philippinense | p1 | (A)8 | 8 | 158095 | 158102 | trnH-GUG_rps19 |
| Paphiopedilum_philippinense | p1 | (A)9 | 9 | 158130 | 158138 | rps19 |
| Paphiopedilum_emersonii | p2 | (TA)4 | 8 | 3715 | 3722 | trnK-UUU intron |
| Paphiopedilum_emersonii | p2 | (AT)4 | 8 | 9100 | 9107 | trnS-GCU_trnG-UCC |
| Paphiopedilum_emersonii | p2 | (TA)4 | 8 | 9180 | 9187 | trnS-GCU_trnG-UCC |
| Paphiopedilum_emersonii | p2 | (TA)5 | 10 | 9224 | 9233 | trnS-GCU_trnG-UCC |
| Paphiopedilum_emersonii | p2 | (TA)4 | 8 | 9278 | 9285 | trnS-GCU_trnG-UCC |
| Paphiopedilum_emersonii | p2 | (TA)4 | 8 | 9287 | 9294 | trnS-GCU_trnG-UCC |
| Paphiopedilum_emersonii | p2 | (TA)4 | 8 | 9296 | 9303 | trnS-GCU_trnG-UCC |
| Paphiopedilum_emersonii | p2 | (TA)5 | 10 | 9305 | 9314 | trnS-GCU_trnG-UCC |
| Paphiopedilum_emersonii | p2 | (TA)4 | 8 | 9361 | 9368 | trnS-GCU_trnG-UCC |
| Paphiopedilum_emersonii | p2 | (TA)6 | 12 | 9383 | 9394 | trnS-GCU_trnG-UCC |
| Paphiopedilum_emersonii | p2 | (TA)4 | 8 | 9409 | 9416 | trnS-GCU_trnG-UCC |
| Paphiopedilum_emersonii | p2 | (TA)4 | 8 | 9426 | 9433 | trnS-GCU_trnG-UCC |
| Paphiopedilum_emersonii | p2 | (AT)5 | 10 | 10687 | 10696 | trnR-UCU_atpA |
| Paphiopedilum_emersonii | p2 | (AT)4 | 8 | 20030 | 20037 | rpoC2 |
| Paphiopedilum_emersonii | p2 | (AT)5 | 10 | 20120 | 20129 | rpoC2 |
| Paphiopedilum_emersonii | p2 | (AT)4 | 8 | 21155 | 21162 | rpoC1 |
| Paphiopedilum_emersonii | p2 | (AT)4 | 8 | 27294 | 27301 | rpoB_trnC-GCA |
| Paphiopedilum_emersonii | p2 | (TA)6 | 12 | 28589 | 28600 | trnC-GCA_petN |
| Paphiopedilum_emersonii | p2 | (AT)4 | 8 | 28609 | 28616 | trnC-GCA_petN |
| Paphiopedilum_emersonii | p2 | (TA)4 | 8 | 29355 | 29362 | petN_psbM |
| Paphiopedilum_emersonii | p2 | (TA)6 | 12 | 29366 | 29377 | petN_psbM |
| Paphiopedilum_emersonii | p2 | (TA)4 | 8 | 29398 | 29405 | petN_psbM |
| Paphiopedilum_emersonii | p2 | (TA)4 | 8 | 29416 | 29423 | petN_psbM |
| Paphiopedilum_emersonii | p2 | (AT)4 | 8 | 29500 | 29507 | petN_psbM |
| Paphiopedilum_emersonii | p2 | (TA)7 | 14 | 29548 | 29561 | petN_psbM |
| Paphiopedilum_emersonii | p2 | (AT)4 | 8 | 29777 | 29784 | petN_psbM |
| Paphiopedilum_emersonii | p2 | (AT)4 | 8 | 29900 | 29907 | petN_psbM |
| Paphiopedilum_emersonii | p2 | (AT)5 | 10 | 29919 | 29928 | petN_psbM |
| Paphiopedilum_emersonii | p2 | (TA)4 | 8 | 30170 | 30177 | petN_psbM |
| Paphiopedilum_emersonii | p2 | (AC)4 | 8 | 30945 | 30952 | psbM_trnD-GUC |
| Paphiopedilum_emersonii | p2 | (TA)4 | 8 | 32142 | 32149 | trnE-UUC_trnT-GGU |
| Paphiopedilum_emersonii | p2 | (GA)4 | 8 | 36050 | 36057 | trnS-UGA |
| Paphiopedilum_emersonii | p2 | (CT)4 | 8 | 45501 | 45508 | trnS-GGA |
| Paphiopedilum_emersonii | p2 | (TA)4 | 8 | 46605 | 46612 | rps4_trnT-UGU |
| Paphiopedilum_emersonii | p2 | (AG)4 | 8 | 48299 | 48306 | trnL-UAA intron |
| Paphiopedilum_emersonii | p2 | (TA)4 | 8 | 54790 | 54797 | atpB_rbcL |
| Paphiopedilum_emersonii | p2 | (TA)4 | 8 | 54820 | 54827 | atpB_rbcL |
| Paphiopedilum_emersonii | p2 | (TA)5 | 10 | 54842 | 54851 | atpB_rbcL |
| Paphiopedilum_emersonii | p2 | (AT)4 | 8 | 56775 | 56782 | rbcL_accD |
| Paphiopedilum_emersonii | p2 | (AT)4 | 8 | 57186 | 57193 | rbcL_accD |
| Paphiopedilum_emersonii | p2 | (TG)4 | 8 | 58492 | 58499 | accD |
| Paphiopedilum_emersonii | p2 | (AT)4 | 8 | 60666 | 60673 | ycf4_cemA |
| Paphiopedilum_emersonii | p2 | (TC)4 | 8 | 61436 | 61443 | cemA |
| Paphiopedilum_emersonii | p2 | (TA)4 | 8 | 63912 | 63919 | petA_psbJ |
| Paphiopedilum_emersonii | p2 | (AT)9 | 18 | 66905 | 66922 | trnP-UGG_psaJ |
| Paphiopedilum_emersonii | p2 | (TA)4 | 8 | 67042 | 67049 | trnP-UGG_psaJ |
| Paphiopedilum_emersonii | p2 | (TA)4 | 8 | 67062 | 67069 | trnP-UGG_psaJ |
| Paphiopedilum_emersonii | p2 | (AT)10 | 20 | 67107 | 67126 | trnP-UGG_psaJ |
| Paphiopedilum_emersonii | p2 | (AT)4 | 8 | 68206 | 68213 | psaJ_rpl33 |
| Paphiopedilum_emersonii | p2 | (TA)4 | 8 | 68784 | 68791 | rpl33_rps18 |
| Paphiopedilum_emersonii | p2 | (TA)4 | 8 | 68795 | 68802 | rpl33_rps18 |
| Paphiopedilum_emersonii | p2 | (TA)6 | 12 | 71425 | 71436 | clpP intron 1 |
| Paphiopedilum_emersonii | p2 | (TA)7 | 14 | 72769 | 72782 | clpP intron 2 |
| Paphiopedilum_emersonii | p2 | (AT)4 | 8 | 73094 | 73101 | clpP intron 2 |
| Paphiopedilum_emersonii | p2 | (TA)4 | 8 | 73646 | 73653 | clpP_psbB |
| Paphiopedilum_emersonii | p2 | (TA)4 | 8 | 73659 | 73666 | clpP_psbB |
| Paphiopedilum_emersonii | p2 | (TA)4 | 8 | 73672 | 73679 | clpP_psbB |
| Paphiopedilum_emersonii | p2 | (TA)4 | 8 | 73818 | 73825 | clpP_psbB |
| Paphiopedilum_emersonii | p2 | (TA)7 | 14 | 75744 | 75757 | psbB_psbT |
| Paphiopedilum_emersonii | p2 | (TA)6 | 12 | 75870 | 75881 | psbB_psbT |
| Paphiopedilum_emersonii | p2 | (AT)4 | 8 | 78377 | 78384 | petB_petD |
| Paphiopedilum_emersonii | p2 | (AT)4 | 8 | 86430 | 86437 | rpl22 |
| Paphiopedilum_emersonii | p2 | (GA)4 | 8 | 89422 | 89429 | ycf2 |
| Paphiopedilum_emersonii | p2 | (GA)4 | 8 | 90415 | 90422 | ycf2 |
| Paphiopedilum_emersonii | p2 | (GA)5 | 10 | 92570 | 92579 | ycf2 |
| Paphiopedilum_emersonii | p2 | (TA)4 | 8 | 96007 | 96014 | ycf2 |
| Paphiopedilum_emersonii | p2 | (CT)4 | 8 | 96645 | 96652 | trnL-CAA_ndhB |
| Paphiopedilum_emersonii | p2 | (AG)4 | 8 | 97342 | 97349 | ndhB |
| Paphiopedilum_emersonii | p2 | (CT)4 | 8 | 108672 | 108679 | rrn23 |
| Paphiopedilum_emersonii | p2 | (AT)7 | 14 | 117904 | 117917 | rps15_psaC |
| Paphiopedilum_emersonii | p2 | (TC)4 | 8 | 119124 | 119131 | psaC_trnL-UAG |
| Paphiopedilum_emersonii | p2 | (TA)4 | 8 | 120782 | 120789 | psaC_trnL-UAG |
| Paphiopedilum_emersonii | p2 | (TA)4 | 8 | 120799 | 120806 | psaC_trnL-UAG |
| Paphiopedilum_emersonii | p2 | (AG)4 | 8 | 123823 | 123830 | ccsA_psaC |
| Paphiopedilum_emersonii | p2 | (AT)7 | 14 | 125038 | 125051 | psaC_rps15 |
| Paphiopedilum_emersonii | p2 | (AG)4 | 8 | 134276 | 134283 | rrn23 |
| Paphiopedilum_emersonii | p2 | (CT)4 | 8 | 145606 | 145613 | ndhB |
| Paphiopedilum_emersonii | p2 | (GA)4 | 8 | 146302 | 146309 | ndhB_trnL-CAA |
| Paphiopedilum_emersonii | p2 | (TA)4 | 8 | 146941 | 146948 | ycf2 |
| Paphiopedilum_emersonii | p2 | (TC)5 | 10 | 150376 | 150385 | ycf2 |
| Paphiopedilum_emersonii | p2 | (TC)4 | 8 | 152533 | 152540 | ycf2 |
| Paphiopedilum_emersonii | p2 | (TC)4 | 8 | 153526 | 153533 | ycf2 |
| Paphiopedilum_victoria-mariae | p2 | (TA)4 | 8 | 3634 | 3641 | trnK-UUU intron |
| Paphiopedilum_victoria-mariae | p2 | (TA)4 | 8 | 3822 | 3829 | trnK-UUU intron |
| Paphiopedilum_victoria-mariae | p2 | (TA)4 | 8 | 3850 | 3857 | trnK-UUU intron |
| Paphiopedilum_victoria-mariae | p2 | (AT)8 | 16 | 8823 | 8838 | trnS-GCU_trnG-UCC |
| Paphiopedilum_victoria-mariae | p2 | (TA)4 | 8 | 9431 | 9438 | trnS-GCU_trnG-UCC |
| Paphiopedilum_victoria-mariae | p2 | (TA)4 | 8 | 9458 | 9465 | trnS-GCU_trnG-UCC |
| Paphiopedilum_victoria-mariae | p2 | (TA)5 | 10 | 9482 | 9491 | trnS-GCU_trnG-UCC |
| Paphiopedilum_victoria-mariae | p2 | (TA)4 | 8 | 9495 | 9502 | trnS-GCU_trnG-UCC |
| Paphiopedilum_victoria-mariae | p2 | (TA)4 | 8 | 9516 | 9523 | trnS-GCU_trnG-UCC |
| Paphiopedilum_victoria-mariae | p2 | (TA)5 | 10 | 9540 | 9549 | trnS-GCU_trnG-UCC |
| Paphiopedilum_victoria-mariae | p2 | (TA)4 | 8 | 9553 | 9560 | trnS-GCU_trnG-UCC |
| Paphiopedilum_victoria-mariae | p2 | (AT)4 | 8 | 9684 | 9691 | trnS-GCU_trnG-UCC |
| Paphiopedilum_victoria-mariae | p2 | (TA)4 | 8 | 9753 | 9760 | trnS-GCU_trnG-UCC |
| Paphiopedilum_victoria-mariae | p2 | (TA)5 | 10 | 11117 | 11126 | trnR-UCU_atpA |
| Paphiopedilum_victoria-mariae | p2 | (AT)5 | 10 | 11164 | 11173 | trnR-UCU_atpA |
| Paphiopedilum_victoria-mariae | p2 | (AT)4 | 8 | 20864 | 20871 | rpoC2 |
| Paphiopedilum_victoria-mariae | p2 | (AT)5 | 10 | 20954 | 20963 | rpoC2 |
| Paphiopedilum_victoria-mariae | p2 | (AT)4 | 8 | 21989 | 21996 | rpoC1 |
| Paphiopedilum_victoria-mariae | p2 | (AT)4 | 8 | 28127 | 28134 | rpoB_trnC-GCA |
| Paphiopedilum_victoria-mariae | p2 | (TA)6 | 12 | 29500 | 29511 | trnC-GCA_petN |
| Paphiopedilum_victoria-mariae | p2 | (AT)4 | 8 | 29537 | 29544 | trnC-GCA_petN |
| Paphiopedilum_victoria-mariae | p2 | (TA)4 | 8 | 30180 | 30187 | petN_psbM |
| Paphiopedilum_victoria-mariae | p2 | (TA)4 | 8 | 30320 | 30327 | petN_psbM |
| Paphiopedilum_victoria-mariae | p2 | (TA)7 | 14 | 30333 | 30346 | petN_psbM |
| Paphiopedilum_victoria-mariae | p2 | (AT)4 | 8 | 30398 | 30405 | petN_psbM |
| Paphiopedilum_victoria-mariae | p2 | (AT)5 | 10 | 30412 | 30421 | petN_psbM |
| Paphiopedilum_victoria-mariae | p2 | (AT)4 | 8 | 30499 | 30506 | petN_psbM |
| Paphiopedilum_victoria-mariae | p2 | (TA)14 | 28 | 30581 | 30608 | petN_psbM |
| Paphiopedilum_victoria-mariae | p2 | (AT)4 | 8 | 30824 | 30831 | petN_psbM |
| Paphiopedilum_victoria-mariae | p2 | (AT)4 | 8 | 30871 | 30878 | petN_psbM |
| Paphiopedilum_victoria-mariae | p2 | (AT)4 | 8 | 30934 | 30941 | petN_psbM |
| Paphiopedilum_victoria-mariae | p2 | (TA)4 | 8 | 31441 | 31448 | petN_psbM |
| Paphiopedilum_victoria-mariae | p2 | (AC)4 | 8 | 32221 | 32228 | psbM_trnD-GUC |
| Paphiopedilum_victoria-mariae | p2 | (AT)4 | 8 | 33446 | 33453 | trnE-UUC_trnT-GGU |
| Paphiopedilum_victoria-mariae | p2 | (TA)6 | 12 | 33481 | 33492 | trnE-UUC_trnT-GGU |
| Paphiopedilum_victoria-mariae | p2 | (AT)4 | 8 | 33809 | 33816 | trnE-UUC_trnT-GGU |
| Paphiopedilum_victoria-mariae | p2 | (AT)4 | 8 | 33908 | 33915 | trnE-UUC_trnT-GGU |
| Paphiopedilum_victoria-mariae | p2 | (TA)4 | 8 | 34381 | 34388 | trnE-UUC_trnT-GGU |
| Paphiopedilum_victoria-mariae | p2 | (GA)4 | 8 | 38291 | 38298 | trnS-UGA |
| Paphiopedilum_victoria-mariae | p2 | (CT)4 | 8 | 47771 | 47778 | trnS-GGA |
| Paphiopedilum_victoria-mariae | p2 | (TA)4 | 8 | 48872 | 48879 | rps4_trnT-UGU |
| Paphiopedilum_victoria-mariae | p2 | (AG)4 | 8 | 50750 | 50757 | trnL-UAA intron |
| Paphiopedilum_victoria-mariae | p2 | (TA)4 | 8 | 57393 | 57400 | atpB_rbcL |
| Paphiopedilum_victoria-mariae | p2 | (AT)5 | 10 | 57450 | 57459 | atpB_rbcL |
| Paphiopedilum_victoria-mariae | p2 | (AT)4 | 8 | 57461 | 57468 | atpB_rbcL |
| Paphiopedilum_victoria-mariae | p2 | (TA)5 | 10 | 57506 | 57515 | atpB_rbcL |
| Paphiopedilum_victoria-mariae | p2 | (AT)4 | 8 | 59437 | 59444 | rbcL_accD |
| Paphiopedilum_victoria-mariae | p2 | (AT)4 | 8 | 59856 | 59863 | rbcL_accD |
| Paphiopedilum_victoria-mariae | p2 | (TG)4 | 8 | 61160 | 61167 | accD |
| Paphiopedilum_victoria-mariae | p2 | (TA)4 | 8 | 62700 | 62707 | psaI_ycf4 |
| Paphiopedilum_victoria-mariae | p2 | (TA)4 | 8 | 63878 | 63885 | ycf4_cemA |
| Paphiopedilum_victoria-mariae | p2 | (AT)4 | 8 | 63893 | 63900 | ycf4_cemA |
| Paphiopedilum_victoria-mariae | p2 | (TA)4 | 8 | 66495 | 66502 | petA_psbJ |
| Paphiopedilum_victoria-mariae | p2 | (AT)5 | 10 | 69452 | 69461 | trnP-UGG_psaJ |
| Paphiopedilum_victoria-mariae | p2 | (AT)4 | 8 | 69851 | 69858 | trnP-UGG_psaJ |
| Paphiopedilum_victoria-mariae | p2 | (AT)4 | 8 | 69896 | 69903 | trnP-UGG_psaJ |
| Paphiopedilum_victoria-mariae | p2 | (AT)4 | 8 | 69918 | 69925 | trnP-UGG_psaJ |
| Paphiopedilum_victoria-mariae | p2 | (AT)4 | 8 | 70698 | 70705 | psaJ_rpl33 |
| Paphiopedilum_victoria-mariae | p2 | (TA)4 | 8 | 71324 | 71331 | rpl33_rps18 |
| Paphiopedilum_victoria-mariae | p2 | (TA)6 | 12 | 73405 | 73416 | clpP |
| Paphiopedilum_victoria-mariae | p2 | (AT)4 | 8 | 73989 | 73996 | clpP intron 1 |
| Paphiopedilum_victoria-mariae | p2 | (AT)6 | 12 | 74003 | 74014 | clpP intron 1 |
| Paphiopedilum_victoria-mariae | p2 | (TA)7 | 14 | 75352 | 75365 | clpP intron 2 |
| Paphiopedilum_victoria-mariae | p2 | (AT)4 | 8 | 75688 | 75695 | clpP intron 2 |
| Paphiopedilum_victoria-mariae | p2 | (TA)4 | 8 | 76224 | 76231 | clpP_psbB |
| Paphiopedilum_victoria-mariae | p2 | (AT)5 | 10 | 76241 | 76250 | clpP_psbB |
| Paphiopedilum_victoria-mariae | p2 | (TA)5 | 10 | 76252 | 76261 | clpP_psbB |
| Paphiopedilum_victoria-mariae | p2 | (TA)4 | 8 | 76267 | 76274 | clpP_psbB |
| Paphiopedilum_victoria-mariae | p2 | (TA)4 | 8 | 76280 | 76287 | clpP_psbB |
| Paphiopedilum_victoria-mariae | p2 | (TA)4 | 8 | 76452 | 76459 | clpP_psbB |
| Paphiopedilum_victoria-mariae | p2 | (TA)6 | 12 | 78316 | 78327 | psbB_psbT |
| Paphiopedilum_victoria-mariae | p2 | (TA)5 | 10 | 78474 | 78483 | psbB_psbT |
| Paphiopedilum_victoria-mariae | p2 | (TA)8 | 16 | 78486 | 78501 | psbB_psbT |
| Paphiopedilum_victoria-mariae | p2 | (AT)4 | 8 | 80964 | 80971 | petB_petD |
| Paphiopedilum_victoria-mariae | p2 | (AT)4 | 8 | 89334 | 89341 | rpl22 |
| Paphiopedilum_victoria-mariae | p2 | (GA)4 | 8 | 92291 | 92298 | ycf2 |
| Paphiopedilum_victoria-mariae | p2 | (GA)4 | 8 | 93278 | 93285 | ycf2 |
| Paphiopedilum_victoria-mariae | p2 | (GA)5 | 10 | 95457 | 95466 | ycf2 |
| Paphiopedilum_victoria-mariae | p2 | (TA)4 | 8 | 98861 | 98868 | ycf2 |
| Paphiopedilum_victoria-mariae | p2 | (CT)4 | 8 | 99970 | 99977 | trnL-CAA_ndhB |
| Paphiopedilum_victoria-mariae | p2 | (AG)4 | 8 | 100667 | 100674 | ndhB |
| Paphiopedilum_victoria-mariae | p2 | (TA)6 | 12 | 105247 | 105258 | rps12_trnV-GAC |
| Paphiopedilum_victoria-mariae | p2 | (CT)4 | 8 | 111920 | 111927 | rrn23 |
| Paphiopedilum_victoria-mariae | p2 | (AT)7 | 14 | 121569 | 121582 | rps15_psaC |
| Paphiopedilum_victoria-mariae | p2 | (TC)4 | 8 | 122783 | 122790 | ndhD_trnL-UAG |
| Paphiopedilum_victoria-mariae | p2 | (TA)6 | 12 | 124552 | 124563 | ndhD_trnL-UAG |
| Paphiopedilum_victoria-mariae | p2 | (TA)8 | 16 | 124565 | 124580 | ndhD_trnL-UAG |
| Paphiopedilum_victoria-mariae | p2 | (AT)4 | 8 | 124660 | 124667 | ndhD_trnL-UAG |
| Paphiopedilum_victoria-mariae | p2 | (AT)4 | 8 | 124750 | 124757 | ndhD_trnL-UAG |
| Paphiopedilum_victoria-mariae | p2 | (AT)4 | 8 | 124769 | 124776 | ndhD_trnL-UAG |
| Paphiopedilum_victoria-mariae | p2 | (TA)4 | 8 | 125191 | 125198 | trnL-UAG_rpl32 |
| Paphiopedilum_victoria-mariae | p2 | (AG)4 | 8 | 127873 | 127880 | ccsA_ndhD |
| Paphiopedilum_victoria-mariae | p2 | (AT)7 | 14 | 129082 | 129095 | psaC_rps15 |
| Paphiopedilum_victoria-mariae | p2 | (AG)4 | 8 | 138737 | 138744 | rrn23 |
| Paphiopedilum_victoria-mariae | p2 | (AT)6 | 12 | 145405 | 145416 | trnV-GAC_rps12 |
| Paphiopedilum_victoria-mariae | p2 | (CT)4 | 8 | 149990 | 149997 | ndhB |
| Paphiopedilum_victoria-mariae | p2 | (GA)4 | 8 | 150686 | 150693 | ndhB_trnL-CAA |
| Paphiopedilum_victoria-mariae | p2 | (TA)4 | 8 | 151796 | 151803 | ycf2 |
| Paphiopedilum_victoria-mariae | p2 | (TC)5 | 10 | 155198 | 155207 | ycf2 |
| Paphiopedilum_victoria-mariae | p2 | (TC)4 | 8 | 157379 | 157386 | ycf2 |
| Paphiopedilum_victoria-mariae | p2 | (TC)4 | 8 | 158366 | 158373 | ycf2 |
| Paphiopedilum_armeniacum | p2 | (TA)6 | 12 | 3616 | 3627 | trnK-UUU intron |
| Paphiopedilum_armeniacum | p2 | (TA)5 | 10 | 3710 | 3719 | trnK-UUU intron |
| Paphiopedilum_armeniacum | p2 | (GT)4 | 8 | 8576 | 8583 | psbK_psbI |
| Paphiopedilum_armeniacum | p2 | (AT)6 | 12 | 9140 | 9151 | trnS-GCU_trnG-UCC |
| Paphiopedilum_armeniacum | p2 | (AT)4 | 8 | 9731 | 9738 | trnS-GCU_trnG-UCC |
| Paphiopedilum_armeniacum | p2 | (TA)4 | 8 | 9824 | 9831 | trnS-GCU_trnG-UCC |
| Paphiopedilum_armeniacum | p2 | (TA)8 | 16 | 9907 | 9922 | trnS-GCU_trnG-UCC |
| Paphiopedilum_armeniacum | p2 | (AT)5 | 10 | 11356 | 11365 | trnR-UCU_atpA |
| Paphiopedilum_armeniacum | p2 | (AT)5 | 10 | 11399 | 11408 | trnR-UCU_atpA |
| Paphiopedilum_armeniacum | p2 | (TA)12 | 24 | 11487 | 11510 | trnR-UCU_atpA |
| Paphiopedilum_armeniacum | p2 | (AT)4 | 8 | 20937 | 20944 | rpoC2 |
| Paphiopedilum_armeniacum | p2 | (AT)5 | 10 | 21027 | 21036 | rpoC2 |
| Paphiopedilum_armeniacum | p2 | (TA)6 | 12 | 30008 | 30019 | trnC-GCA_petN |
| Paphiopedilum_armeniacum | p2 | (TA)7 | 14 | 30647 | 30660 | petN_psbM |
| Paphiopedilum_armeniacum | p2 | (TA)8 | 16 | 30663 | 30678 | petN_psbM |
| Paphiopedilum_armeniacum | p2 | (TA)10 | 20 | 30681 | 30700 | petN_psbM |
| Paphiopedilum_armeniacum | p2 | (TA)4 | 8 | 30778 | 30785 | petN_psbM |
| Paphiopedilum_armeniacum | p2 | (TA)4 | 8 | 30792 | 30799 | petN_psbM |
| Paphiopedilum_armeniacum | p2 | (TA)4 | 8 | 30801 | 30808 | petN_psbM |
| Paphiopedilum_armeniacum | p2 | (AT)4 | 8 | 30869 | 30876 | petN_psbM |
| Paphiopedilum_armeniacum | p2 | (TA)5 | 10 | 30896 | 30905 | petN_psbM |
| Paphiopedilum_armeniacum | p2 | (AT)4 | 8 | 31125 | 31132 | petN_psbM |
| Paphiopedilum_armeniacum | p2 | (AT)4 | 8 | 31172 | 31179 | petN_psbM |
| Paphiopedilum_armeniacum | p2 | (AT)4 | 8 | 31226 | 31233 | petN_psbM |
| Paphiopedilum_armeniacum | p2 | (TA)6 | 12 | 31308 | 31319 | petN_psbM |
| Paphiopedilum_armeniacum | p2 | (AT)4 | 8 | 31334 | 31341 | petN_psbM |
| Paphiopedilum_armeniacum | p2 | (TA)4 | 8 | 31538 | 31545 | petN_psbM |
| Paphiopedilum_armeniacum | p2 | (TA)4 | 8 | 31703 | 31710 | petN_psbM |
| Paphiopedilum_armeniacum | p2 | (AC)4 | 8 | 32509 | 32516 | psbM_trnD-GUC |
| Paphiopedilum_armeniacum | p2 | (AT)5 | 10 | 33731 | 33740 | trnE-UUC_trnT-GGU |
| Paphiopedilum_armeniacum | p2 | (AT)4 | 8 | 34121 | 34128 | trnE-UUC_trnT-GGU |
| Paphiopedilum_armeniacum | p2 | (AT)8 | 16 | 34354 | 34369 | trnE-UUC_trnT-GGU |
| Paphiopedilum_armeniacum | p2 | (AT)6 | 12 | 34465 | 34476 | trnE-UUC_trnT-GGU |
| Paphiopedilum_armeniacum | p2 | (TA)4 | 8 | 34944 | 34951 | trnE-UUC_trnT-GGU |
| Paphiopedilum_armeniacum | p2 | (GA)4 | 8 | 38852 | 38859 | trnS-UGA |
| Paphiopedilum_armeniacum | p2 | (CT)4 | 8 | 48191 | 48198 | trnS-GGA |
| Paphiopedilum_armeniacum | p2 | (TA)4 | 8 | 49282 | 49289 | rps4_trnT-UGU |
| Paphiopedilum_armeniacum | p2 | (TA)4 | 8 | 50339 | 50346 | trnT-UGU_trnL-UAA |
| Paphiopedilum_armeniacum | p2 | (AG)4 | 8 | 51113 | 51120 | trnL-UAA intron |
| Paphiopedilum_armeniacum | p2 | (TA)4 | 8 | 58894 | 58901 | atpB_rbcL |
| Paphiopedilum_armeniacum | p2 | (TA)5 | 10 | 58970 | 58979 | atpB_rbcL |
| Paphiopedilum_armeniacum | p2 | (AT)4 | 8 | 61275 | 61282 | rbcL_accD |
| Paphiopedilum_armeniacum | p2 | (TG)4 | 8 | 62606 | 62613 | accD |
| Paphiopedilum_armeniacum | p2 | (TA)7 | 14 | 65165 | 65178 | ycf4_cemA |
| Paphiopedilum_armeniacum | p2 | (TA)5 | 10 | 65296 | 65305 | ycf4_cemA |
| Paphiopedilum_armeniacum | p2 | (TC)4 | 8 | 66059 | 66066 | cemA |
| Paphiopedilum_armeniacum | p2 | (TA)4 | 8 | 68543 | 68550 | petA_psbJ |
| Paphiopedilum_armeniacum | p2 | (TA)4 | 8 | 68568 | 68575 | petA_psbJ |
| Paphiopedilum_armeniacum | p2 | (TA)4 | 8 | 71617 | 71624 | trnP-UGG_psaJ |
| Paphiopedilum_armeniacum | p2 | (TA)7 | 14 | 71635 | 71648 | trnP-UGG_psaJ |
| Paphiopedilum_armeniacum | p2 | (AT)4 | 8 | 71874 | 71881 | trnP-UGG_psaJ |
| Paphiopedilum_armeniacum | p2 | (AT)6 | 12 | 71919 | 71930 | trnP-UGG_psaJ |
| Paphiopedilum_armeniacum | p2 | (TA)5 | 10 | 73231 | 73240 | rpl33_rps18 |
| Paphiopedilum_armeniacum | p2 | (TA)4 | 8 | 73243 | 73250 | rpl33_rps18 |
| Paphiopedilum_armeniacum | p2 | (TA)5 | 10 | 75879 | 75888 | clpP intron 1 |
| Paphiopedilum_armeniacum | p2 | (TA)19 | 38 | 77178 | 77215 | clpP intron 2 |
| Paphiopedilum_armeniacum | p2 | (TA)5 | 10 | 77227 | 77236 | clpP intron 2 |
| Paphiopedilum_armeniacum | p2 | (AT)4 | 8 | 77580 | 77587 | clpP intron 2 |
| Paphiopedilum_armeniacum | p2 | (TA)4 | 8 | 77961 | 77968 | clpP_psbB |
| Paphiopedilum_armeniacum | p2 | (AT)5 | 10 | 77988 | 77997 | clpP_psbB |
| Paphiopedilum_armeniacum | p2 | (TA)4 | 8 | 78060 | 78067 | clpP_psbB |
| Paphiopedilum_armeniacum | p2 | (TA)6 | 12 | 78089 | 78100 | clpP_psbB |
| Paphiopedilum_armeniacum | p2 | (TA)5 | 10 | 78134 | 78143 | clpP_psbB |
| Paphiopedilum_armeniacum | p2 | (TA)4 | 8 | 78149 | 78156 | clpP_psbB |
| Paphiopedilum_armeniacum | p2 | (TA)6 | 12 | 78319 | 78330 | clpP_psbB |
| Paphiopedilum_armeniacum | p2 | (TA)10 | 20 | 78346 | 78365 | clpP_psbB |
| Paphiopedilum_armeniacum | p2 | (TA)6 | 12 | 78367 | 78378 | clpP_psbB |
| Paphiopedilum_armeniacum | p2 | (TA)4 | 8 | 78483 | 78490 | clpP_psbB |
| Paphiopedilum_armeniacum | p2 | (TA)8 | 16 | 80409 | 80424 | psbB_psbT |
| Paphiopedilum_armeniacum | p2 | (TA)10 | 20 | 80761 | 80780 | psbB_psbT |
| Paphiopedilum_armeniacum | p2 | (AT)4 | 8 | 83232 | 83239 | petB_petD |
| Paphiopedilum_armeniacum | p2 | (TA)5 | 10 | 89765 | 89774 | rpl16 intron |
| Paphiopedilum_armeniacum | p2 | (TA)5 | 10 | 89782 | 89791 | rpl16 intron |
| Paphiopedilum_armeniacum | p2 | (TA)8 | 16 | 90019 | 90034 | rpl16 intron |
| Paphiopedilum_armeniacum | p2 | (TA)4 | 8 | 90168 | 90175 | rpl16 intron |
| Paphiopedilum_armeniacum | p2 | (AT)4 | 8 | 91930 | 91937 | rpl22 |
| Paphiopedilum_armeniacum | p2 | (GA)4 | 8 | 94887 | 94894 | ycf2 |
| Paphiopedilum_armeniacum | p2 | (GA)4 | 8 | 95874 | 95881 | ycf2 |
| Paphiopedilum_armeniacum | p2 | (GA)5 | 10 | 98095 | 98104 | ycf2 |
| Paphiopedilum_armeniacum | p2 | (TA)4 | 8 | 101508 | 101515 | ycf2 |
| Paphiopedilum_armeniacum | p2 | (AG)4 | 8 | 103739 | 103746 | ndhB intron |
| Paphiopedilum_armeniacum | p2 | (TA)6 | 12 | 108304 | 108315 | rps12_trnV-GAC |
| Paphiopedilum_armeniacum | p2 | (CT)4 | 8 | 115143 | 115150 | rrn23 |
| Paphiopedilum_armeniacum | p2 | (AT)5 | 10 | 124598 | 124607 | rps15_psaC |
| Paphiopedilum_armeniacum | p2 | (TA)15 | 30 | 125893 | 125922 | rpl32_trnL-UAG |
| Paphiopedilum_armeniacum | p2 | (AT)5 | 10 | 130018 | 130027 | psaC_rps15 |
| Paphiopedilum_armeniacum | p2 | (AG)4 | 8 | 139475 | 139482 | rrn23 |
| Paphiopedilum_armeniacum | p2 | (AT)6 | 12 | 146309 | 146320 | trnV-GAC_rps12 |
| Paphiopedilum_armeniacum | p2 | (CT)4 | 8 | 150879 | 150886 | ndhB intron |
| Paphiopedilum_armeniacum | p2 | (TA)4 | 8 | 153110 | 153117 | ycf2 |
| Paphiopedilum_armeniacum | p2 | (TC)5 | 10 | 156521 | 156530 | ycf2 |
| Paphiopedilum_armeniacum | p2 | (TC)4 | 8 | 158744 | 158751 | ycf2 |
| Paphiopedilum_armeniacum | p2 | (TC)4 | 8 | 159731 | 159738 | ycf2 |
| Paphiopedilum_dianthum | p2 | (TA)4 | 8 | 7481 | 7488 | psbK_psbI |
| Paphiopedilum_dianthum | p2 | (TA)4 | 8 | 7499 | 7506 | psbK_psbI |
| Paphiopedilum_dianthum | p2 | (AT)9 | 18 | 8354 | 8371 | trnS-GCU_trnG-UCC |
| Paphiopedilum_dianthum | p2 | (AT)4 | 8 | 8993 | 9000 | trnS-GCU_trnG-UCC |
| Paphiopedilum_dianthum | p2 | (TA)4 | 8 | 9056 | 9063 | trnS-GCU_trnG-UCC |
| Paphiopedilum_dianthum | p2 | (TA)4 | 8 | 9165 | 9172 | trnS-GCU_trnG-UCC |
| Paphiopedilum_dianthum | p2 | (AT)4 | 8 | 20196 | 20203 | rpoC2 |
| Paphiopedilum_dianthum | p2 | (AT)5 | 10 | 20286 | 20295 | rpoC2 |
| Paphiopedilum_dianthum | p2 | (AT)4 | 8 | 21321 | 21328 | rpoC1 |
| Paphiopedilum_dianthum | p2 | (AT)4 | 8 | 27468 | 27475 | rpoB_trnC-GCA |
| Paphiopedilum_dianthum | p2 | (AT)4 | 8 | 28854 | 28861 | trnC-GCA_petN |
| Paphiopedilum_dianthum | p2 | (AT)5 | 10 | 29414 | 29423 | petN_psbM |
| Paphiopedilum_dianthum | p2 | (TA)4 | 8 | 29428 | 29435 | petN_psbM |
| Paphiopedilum_dianthum | p2 | (TA)4 | 8 | 29631 | 29638 | petN_psbM |
| Paphiopedilum_dianthum | p2 | (AC)4 | 8 | 30412 | 30419 | psbM_trnD-GUC |
| Paphiopedilum_dianthum | p2 | (AT)4 | 8 | 31443 | 31450 | trnE-UUC_trnT-GGU |
| Paphiopedilum_dianthum | p2 | (TA)4 | 8 | 31471 | 31478 | trnE-UUC_trnT-GGU |
| Paphiopedilum_dianthum | p2 | (TA)4 | 8 | 31498 | 31505 | trnE-UUC_trnT-GGU |
| Paphiopedilum_dianthum | p2 | (TA)4 | 8 | 31516 | 31523 | trnE-UUC_trnT-GGU |
| Paphiopedilum_dianthum | p2 | (TA)4 | 8 | 31531 | 31538 | trnE-UUC_trnT-GGU |
| Paphiopedilum_dianthum | p2 | (TA)7 | 14 | 31561 | 31574 | trnE-UUC_trnT-GGU |
| Paphiopedilum_dianthum | p2 | (TA)5 | 10 | 31576 | 31585 | trnE-UUC_trnT-GGU |
| Paphiopedilum_dianthum | p2 | (TA)6 | 12 | 31603 | 31614 | trnE-UUC_trnT-GGU |
| Paphiopedilum_dianthum | p2 | (TA)8 | 16 | 31637 | 31652 | trnE-UUC_trnT-GGU |
| Paphiopedilum_dianthum | p2 | (TA)4 | 8 | 31725 | 31732 | trnE-UUC_trnT-GGU |
| Paphiopedilum_dianthum | p2 | (TA)4 | 8 | 31735 | 31742 | trnE-UUC_trnT-GGU |
| Paphiopedilum_dianthum | p2 | (TA)4 | 8 | 32658 | 32665 | trnE-UUC_trnT-GGU |
| Paphiopedilum_dianthum | p2 | (GA)4 | 8 | 36460 | 36467 | trnS-UGA |
| Paphiopedilum_dianthum | p2 | (CT)4 | 8 | 45792 | 45799 | trnS-GGA |
| Paphiopedilum_dianthum | p2 | (TA)4 | 8 | 46902 | 46909 | rps4_trnT-UGU |
| Paphiopedilum_dianthum | p2 | (AG)4 | 8 | 48592 | 48599 | trnL-UAA intron |
| Paphiopedilum_dianthum | p2 | (TA)4 | 8 | 55030 | 55037 | atpB_rbcL |
| Paphiopedilum_dianthum | p2 | (TA)6 | 12 | 55049 | 55060 | atpB_rbcL |
| Paphiopedilum_dianthum | p2 | (TA)12 | 24 | 55227 | 55250 | atpB_rbcL |
| Paphiopedilum_dianthum | p2 | (AT)4 | 8 | 57568 | 57575 | rbcL_accD |
| Paphiopedilum_dianthum | p2 | (TG)4 | 8 | 58933 | 58940 | accD |
| Paphiopedilum_dianthum | p2 | (AT)4 | 8 | 61515 | 61522 | ycf4_cemA |
| Paphiopedilum_dianthum | p2 | (AT)4 | 8 | 61624 | 61631 | ycf4_cemA |
| Paphiopedilum_dianthum | p2 | (AT)5 | 10 | 67009 | 67018 | trnP-UGG_psaJ |
| Paphiopedilum_dianthum | p2 | (AT)4 | 8 | 67162 | 67169 | trnP-UGG_psaJ |
| Paphiopedilum_dianthum | p2 | (AT)4 | 8 | 67499 | 67506 | trnP-UGG_psaJ |
| Paphiopedilum_dianthum | p2 | (AT)4 | 8 | 67548 | 67555 | trnP-UGG_psaJ |
| Paphiopedilum_dianthum | p2 | (AT)4 | 8 | 67574 | 67581 | trnP-UGG_psaJ |
| Paphiopedilum_dianthum | p2 | (AT)4 | 8 | 67625 | 67632 | trnP-UGG_psaJ |
| Paphiopedilum_dianthum | p2 | (AT)4 | 8 | 68544 | 68551 | psaJ_rpl33 |
| Paphiopedilum_dianthum | p2 | (TA)4 | 8 | 69208 | 69215 | rpl33_rps18 |
| Paphiopedilum_dianthum | p2 | (TA)7 | 14 | 71283 | 71296 | clpP |
| Paphiopedilum_dianthum | p2 | (TA)4 | 8 | 71846 | 71853 | clpP intron 1 |
| Paphiopedilum_dianthum | p2 | (AT)4 | 8 | 73602 | 73609 | clpP intron 2 |
| Paphiopedilum_dianthum | p2 | (TA)5 | 10 | 73992 | 74001 | clpP_psbB |
| Paphiopedilum_dianthum | p2 | (AT)4 | 8 | 74004 | 74011 | clpP_psbB |
| Paphiopedilum_dianthum | p2 | (TA)4 | 8 | 74016 | 74023 | clpP_psbB |
| Paphiopedilum_dianthum | p2 | (TA)4 | 8 | 74029 | 74036 | clpP_psbB |
| Paphiopedilum_dianthum | p2 | (TA)4 | 8 | 74173 | 74180 | clpP_psbB |
| Paphiopedilum_dianthum | p2 | (TA)7 | 14 | 76099 | 76112 | psbB_psbT |
| Paphiopedilum_dianthum | p2 | (AT)4 | 8 | 76114 | 76121 | psbB_psbT |
| Paphiopedilum_dianthum | p2 | (AT)4 | 8 | 78593 | 78600 | petB_petD |
| Paphiopedilum_dianthum | p2 | (TA)4 | 8 | 85115 | 85122 | rpl16 intron |
| Paphiopedilum_dianthum | p2 | (AT)4 | 8 | 86832 | 86839 | rpl22 |
| Paphiopedilum_dianthum | p2 | (GA)4 | 8 | 89782 | 89789 | ycf2 |
| Paphiopedilum_dianthum | p2 | (GA)4 | 8 | 90769 | 90776 | ycf2 |
| Paphiopedilum_dianthum | p2 | (GA)5 | 10 | 92930 | 92939 | ycf2 |
| Paphiopedilum_dianthum | p2 | (TA)4 | 8 | 96328 | 96335 | ycf2 |
| Paphiopedilum_dianthum | p2 | (CT)4 | 8 | 97252 | 97259 | trnL-CAA_ndhB |
| Paphiopedilum_dianthum | p2 | (AG)4 | 8 | 97949 | 97956 | ndhB intron |
| Paphiopedilum_dianthum | p2 | (TA)6 | 12 | 101838 | 101849 | rps12_trnV-GAC |
| Paphiopedilum_dianthum | p2 | (TA)7 | 14 | 101853 | 101866 | rps12_trnV-GAC |
| Paphiopedilum_dianthum | p2 | (TA)9 | 18 | 101883 | 101900 | rps12_trnV-GAC |
| Paphiopedilum_dianthum | p2 | (TA)6 | 12 | 101902 | 101913 | rps12_trnV-GAC |
| Paphiopedilum_dianthum | p2 | (CT)4 | 8 | 109066 | 109073 | rrn23 |
| Paphiopedilum_dianthum | p2 | (AT)7 | 14 | 118439 | 118452 | rps15_psaC |
| Paphiopedilum_dianthum | p2 | (TA)4 | 8 | 119426 | 119433 | trnL-UAG_rpl32 |
| Paphiopedilum_dianthum | p2 | (AT)4 | 8 | 122127 | 122134 | ndhD_psaC |
| Paphiopedilum_dianthum | p2 | (AT)7 | 14 | 123109 | 123122 | psaC_rps15 |
| Paphiopedilum_dianthum | p2 | (AG)4 | 8 | 132488 | 132495 | rrn23 |
| Paphiopedilum_dianthum | p2 | (AT)6 | 12 | 139647 | 139658 | trnV-GAC_rps12 |
| Paphiopedilum_dianthum | p2 | (AT)9 | 18 | 139660 | 139677 | trnV-GAC_rps12 |
| Paphiopedilum_dianthum | p2 | (TA)7 | 14 | 139695 | 139708 | trnV-GAC_rps12 |
| Paphiopedilum_dianthum | p2 | (TA)6 | 12 | 139712 | 139723 | trnV-GAC_rps12 |
| Paphiopedilum_dianthum | p2 | (CT)4 | 8 | 143605 | 143612 | ndhB intron |
| Paphiopedilum_dianthum | p2 | (GA)4 | 8 | 144301 | 144308 | ndhB_trnL-CAA |
| Paphiopedilum_dianthum | p2 | (TA)4 | 8 | 145226 | 145233 | ycf2 |
| Paphiopedilum_dianthum | p2 | (TC)5 | 10 | 148622 | 148631 | ycf2 |
| Paphiopedilum_dianthum | p2 | (TC)4 | 8 | 150785 | 150792 | ycf2 |
| Paphiopedilum_dianthum | p2 | (TC)4 | 8 | 151772 | 151779 | ycf2 |
| Paphiopedilum_violascens | p2 | (TA)4 | 8 | 3694 | 3701 | trnK-UUU intron |
| Paphiopedilum_violascens | p2 | (TA)4 | 8 | 3722 | 3729 | trnK-UUU intron |
| Paphiopedilum_violascens | p2 | (AT)4 | 8 | 4980 | 4987 | matK_rps16 |
| Paphiopedilum_violascens | p2 | (AT)4 | 8 | 8931 | 8938 | trnS-GCU_trnG-UCC |
| Paphiopedilum_violascens | p2 | (TA)4 | 8 | 9043 | 9050 | trnS-GCU_trnG-UCC |
| Paphiopedilum_violascens | p2 | (TA)6 | 12 | 10289 | 10300 | trnR-UCU_atpA |
| Paphiopedilum_violascens | p2 | (AT)4 | 8 | 20017 | 20024 | rpoC2 |
| Paphiopedilum_violascens | p2 | (AT)5 | 10 | 20107 | 20116 | rpoC2 |
| Paphiopedilum_violascens | p2 | (AT)4 | 8 | 21142 | 21149 | rpoC1 |
| Paphiopedilum_violascens | p2 | (AT)4 | 8 | 27282 | 27289 | rpoB_trnC-GCA |
| Paphiopedilum_violascens | p2 | (TA)4 | 8 | 28639 | 28646 | trnC-GCA_petN |
| Paphiopedilum_violascens | p2 | (AT)4 | 8 | 28675 | 28682 | trnC-GCA_petN |
| Paphiopedilum_violascens | p2 | (AT)4 | 8 | 29381 | 29388 | petN_psbM |
| Paphiopedilum_violascens | p2 | (AT)4 | 8 | 29403 | 29410 | petN_psbM |
| Paphiopedilum_violascens | p2 | (TA)7 | 14 | 29431 | 29444 | petN_psbM |
| Paphiopedilum_violascens | p2 | (AT)4 | 8 | 29668 | 29675 | petN_psbM |
| Paphiopedilum_violascens | p2 | (AT)4 | 8 | 29726 | 29733 | petN_psbM |
| Paphiopedilum_violascens | p2 | (AT)4 | 8 | 29798 | 29805 | petN_psbM |
| Paphiopedilum_violascens | p2 | (AT)4 | 8 | 29887 | 29894 | petN_psbM |
| Paphiopedilum_violascens | p2 | (AT)4 | 8 | 29901 | 29908 | petN_psbM |
| Paphiopedilum_violascens | p2 | (AT)4 | 8 | 29926 | 29933 | petN_psbM |
| Paphiopedilum_violascens | p2 | (TA)4 | 8 | 29952 | 29959 | petN_psbM |
| Paphiopedilum_violascens | p2 | (TA)4 | 8 | 29961 | 29968 | petN_psbM |
| Paphiopedilum_violascens | p2 | (AT)4 | 8 | 29972 | 29979 | petN_psbM |
| Paphiopedilum_violascens | p2 | (AT)4 | 8 | 29984 | 29991 | petN_psbM |
| Paphiopedilum_violascens | p2 | (TA)4 | 8 | 30128 | 30135 | petN_psbM |
| Paphiopedilum_violascens | p2 | (TA)4 | 8 | 30348 | 30355 | petN_psbM |
| Paphiopedilum_violascens | p2 | (AC)4 | 8 | 31135 | 31142 | psbM_trnD-GUC |
| Paphiopedilum_violascens | p2 | (AT)4 | 8 | 32337 | 32344 | trnE-UUC_trnT-GGU |
| Paphiopedilum_violascens | p2 | (AT)4 | 8 | 32346 | 32353 | trnE-UUC_trnT-GGU |
| Paphiopedilum_violascens | p2 | (AT)6 | 12 | 32394 | 32405 | trnE-UUC_trnT-GGU |
| Paphiopedilum_violascens | p2 | (AT)5 | 10 | 32457 | 32466 | trnE-UUC_trnT-GGU |
| Paphiopedilum_violascens | p2 | (TA)4 | 8 | 33351 | 33358 | trnE-UUC_trnT-GGU |
| Paphiopedilum_violascens | p2 | (GA)4 | 8 | 37234 | 37241 | trnS-UGA |
| Paphiopedilum_violascens | p2 | (CT)4 | 8 | 46680 | 46687 | trnS-GGA |
| Paphiopedilum_violascens | p2 | (TA)4 | 8 | 47861 | 47868 | rps4_trnT-UGU |
| Paphiopedilum_violascens | p2 | (AG)4 | 8 | 49702 | 49709 | trnL-UAA intron |
| Paphiopedilum_violascens | p2 | (TA)6 | 12 | 56271 | 56282 | atpB_rbcL |
| Paphiopedilum_violascens | p2 | (TA)4 | 8 | 56285 | 56292 | atpB_rbcL |
| Paphiopedilum_violascens | p2 | (TA)4 | 8 | 56302 | 56309 | atpB_rbcL |
| Paphiopedilum_violascens | p2 | (TA)6 | 12 | 56337 | 56348 | atpB_rbcL |
| Paphiopedilum_violascens | p2 | (TA)8 | 16 | 56354 | 56369 | atpB_rbcL |
| Paphiopedilum_violascens | p2 | (AT)4 | 8 | 58328 | 58335 | rbcL_accD |
| Paphiopedilum_violascens | p2 | (AT)4 | 8 | 58710 | 58717 | rbcL_accD |
| Paphiopedilum_violascens | p2 | (TG)4 | 8 | 60022 | 60029 | accD |
| Paphiopedilum_violascens | p2 | (AT)4 | 8 | 62633 | 62640 | ycf4_petA |
| Paphiopedilum_violascens | p2 | (TA)4 | 8 | 64549 | 64556 | petA_psbJ |
| Paphiopedilum_violascens | p2 | (TA)4 | 8 | 64561 | 64568 | petA_psbJ |
| Paphiopedilum_violascens | p2 | (AT)5 | 10 | 67560 | 67569 | trnP-UGG_psaJ |
| Paphiopedilum_violascens | p2 | (AT)4 | 8 | 68019 | 68026 | trnP-UGG_psaJ |
| Paphiopedilum_violascens | p2 | (AT)4 | 8 | 68067 | 68074 | trnP-UGG_psaJ |
| Paphiopedilum_violascens | p2 | (AT)4 | 8 | 68089 | 68096 | trnP-UGG_psaJ |
| Paphiopedilum_violascens | p2 | (AT)4 | 8 | 68927 | 68934 | psaJ_rpl33 |
| Paphiopedilum_violascens | p2 | (TA)4 | 8 | 69535 | 69542 | rpl33_rps18 |
| Paphiopedilum_violascens | p2 | (TA)4 | 8 | 69988 | 69995 | rps18_rpl20 |
| Paphiopedilum_violascens | p2 | (TA)4 | 8 | 72193 | 72200 | clpP intron 1 |
| Paphiopedilum_violascens | p2 | (TA)5 | 10 | 72257 | 72266 | clpP intron 1 |
| Paphiopedilum_violascens | p2 | (TA)4 | 8 | 72285 | 72292 | clpP intron 1 |
| Paphiopedilum_violascens | p2 | (TA)7 | 14 | 73654 | 73667 | clpP intron 2 |
| Paphiopedilum_violascens | p2 | (AT)4 | 8 | 73670 | 73677 | clpP intron 2 |
| Paphiopedilum_violascens | p2 | (AT)4 | 8 | 74115 | 74122 | clpP intron 2 |
| Paphiopedilum_violascens | p2 | (TA)4 | 8 | 74561 | 74568 | clpP_psbB |
| Paphiopedilum_violascens | p2 | (TA)5 | 10 | 74581 | 74590 | clpP_psbB |
| Paphiopedilum_violascens | p2 | (TA)4 | 8 | 74593 | 74600 | clpP_psbB |
| Paphiopedilum_violascens | p2 | (TA)4 | 8 | 74606 | 74613 | clpP_psbB |
| Paphiopedilum_violascens | p2 | (TA)4 | 8 | 74619 | 74626 | clpP_psbB |
| Paphiopedilum_violascens | p2 | (TA)4 | 8 | 74758 | 74765 | clpP_psbB |
| Paphiopedilum_violascens | p2 | (AT)4 | 8 | 74971 | 74978 | clpP_psbB |
| Paphiopedilum_violascens | p2 | (TA)7 | 14 | 76708 | 76721 | psbB_psbT |
| Paphiopedilum_violascens | p2 | (TA)8 | 16 | 76993 | 77008 | psbB_psbT |
| Paphiopedilum_violascens | p2 | (AT)4 | 8 | 79387 | 79394 | petB_petD |
| Paphiopedilum_violascens | p2 | (TA)5 | 10 | 79908 | 79917 | petD intron |
| Paphiopedilum_violascens | p2 | (TA)4 | 8 | 85876 | 85883 | rpl16 intron |
| Paphiopedilum_violascens | p2 | (TA)4 | 8 | 85899 | 85906 | rpl16 intron |
| Paphiopedilum_violascens | p2 | (AT)4 | 8 | 87645 | 87652 | rpl22 |
| Paphiopedilum_violascens | p2 | (GA)4 | 8 | 90602 | 90609 | ycf2 |
| Paphiopedilum_violascens | p2 | (GA)4 | 8 | 91589 | 91596 | ycf2 |
| Paphiopedilum_violascens | p2 | (GA)5 | 10 | 93810 | 93819 | ycf2 |
| Paphiopedilum_violascens | p2 | (TA)4 | 8 | 97214 | 97221 | ycf2 |
| Paphiopedilum_violascens | p2 | (CT)4 | 8 | 97864 | 97871 | trnL-CAA_ndhB |
| Paphiopedilum_violascens | p2 | (AG)4 | 8 | 98562 | 98569 | ndhB |
| Paphiopedilum_violascens | p2 | (CT)4 | 8 | 109661 | 109668 | rrn23 |
| Paphiopedilum_violascens | p2 | (AT)6 | 12 | 119084 | 119095 | rps15_psaC |
| Paphiopedilum_violascens | p2 | (TC)4 | 8 | 120302 | 120309 | ndhD_trnL-UAG |
| Paphiopedilum_violascens | p2 | (TA)4 | 8 | 122074 | 122081 | ndhD_trnL-UAG |
| Paphiopedilum_violascens | p2 | (AG)4 | 8 | 125218 | 125225 | ccsA_ndhD |
| Paphiopedilum_violascens | p2 | (AT)6 | 12 | 126433 | 126444 | psaC_rps15 |
| Paphiopedilum_violascens | p2 | (AG)4 | 8 | 135860 | 135867 | rrn23 |
| Paphiopedilum_violascens | p2 | (CT)4 | 8 | 146959 | 146966 | ndhB |
| Paphiopedilum_violascens | p2 | (GA)4 | 8 | 147656 | 147663 | ndhB_trnL-CAA |
| Paphiopedilum_violascens | p2 | (TA)4 | 8 | 148307 | 148314 | ycf2 |
| Paphiopedilum_violascens | p2 | (TC)5 | 10 | 151709 | 151718 | ycf2 |
| Paphiopedilum_violascens | p2 | (TC)4 | 8 | 153932 | 153939 | ycf2 |
| Paphiopedilum_violascens | p2 | (TC)4 | 8 | 154919 | 154926 | ycf2 |
| Paphiopedilum_niveum | p2 | (AT)4 | 8 | 16 | 23 | rps19_psbA |
| Paphiopedilum_niveum | p2 | (TA)4 | 8 | 3705 | 3712 | trnK-UUU intron |
| Paphiopedilum_niveum | p2 | (AT)4 | 8 | 3732 | 3739 | trnK-UUU intron |
| Paphiopedilum_niveum | p2 | (TA)4 | 8 | 3767 | 3774 | trnK-UUU intron |
| Paphiopedilum_niveum | p2 | (TA)4 | 8 | 3795 | 3802 | trnK-UUU intron |
| Paphiopedilum_niveum | p2 | (TA)4 | 8 | 3823 | 3830 | trnK-UUU intron |
| Paphiopedilum_niveum | p2 | (AT)4 | 8 | 5214 | 5221 | matK_rps16 |
| Paphiopedilum_niveum | p2 | (TA)6 | 12 | 5977 | 5988 | rps16 intron |
| Paphiopedilum_niveum | p2 | (TA)5 | 10 | 6020 | 6029 | rps16 intron |
| Paphiopedilum_niveum | p2 | (AT)6 | 12 | 9351 | 9362 | trnS-GCU_trnG-GCC |
| Paphiopedilum_niveum | p2 | (AT)4 | 8 | 9819 | 9826 | trnS-GCU_trnG-GCC |
| Paphiopedilum_niveum | p2 | (TA)4 | 8 | 9941 | 9948 | trnS-GCU_trnG-GCC |
| Paphiopedilum_niveum | p2 | (TA)5 | 10 | 9951 | 9960 | trnS-GCU_trnG-GCC |
| Paphiopedilum_niveum | p2 | (TA)4 | 8 | 9964 | 9971 | trnS-GCU_trnG-GCC |
| Paphiopedilum_niveum | p2 | (TA)7 | 14 | 11267 | 11280 | trnR-UCU_atpA |
| Paphiopedilum_niveum | p2 | (AT)9 | 18 | 11289 | 11306 | trnR-UCU_atpA |
| Paphiopedilum_niveum | p2 | (AT)4 | 8 | 20939 | 20946 | rpoC2 |
| Paphiopedilum_niveum | p2 | (AT)5 | 10 | 21029 | 21038 | rpoC2 |
| Paphiopedilum_niveum | p2 | (AT)4 | 8 | 22064 | 22071 | rpoC1 |
| Paphiopedilum_niveum | p2 | (AT)4 | 8 | 28208 | 28215 | rpoB_trnC-GCA |
| Paphiopedilum_niveum | p2 | (TA)5 | 10 | 29580 | 29589 | trnC-GCA_petN |
| Paphiopedilum_niveum | p2 | (TA)6 | 12 | 29592 | 29603 | trnC-GCA_petN |
| Paphiopedilum_niveum | p2 | (AT)4 | 8 | 30239 | 30246 | petN_psbM |
| Paphiopedilum_niveum | p2 | (TA)5 | 10 | 30266 | 30275 | petN_psbM |
| Paphiopedilum_niveum | p2 | (AC)4 | 8 | 31068 | 31075 | psbM_trnD-GUC |
| Paphiopedilum_niveum | p2 | (AT)4 | 8 | 32284 | 32291 | trnE-UUC_trnT-GGU |
| Paphiopedilum_niveum | p2 | (TA)4 | 8 | 32303 | 32310 | trnE-UUC_trnT-GGU |
| Paphiopedilum_niveum | p2 | (TA)4 | 8 | 32536 | 32543 | trnE-UUC_trnT-GGU |
| Paphiopedilum_niveum | p2 | (AT)5 | 10 | 32841 | 32850 | trnE-UUC_trnT-GGU |
| Paphiopedilum_niveum | p2 | (TA)4 | 8 | 33356 | 33363 | trnE-UUC_trnT-GGU |
| Paphiopedilum_niveum | p2 | (GA)4 | 8 | 37269 | 37276 | trnS-UGA |
| Paphiopedilum_niveum | p2 | (CT)4 | 8 | 46665 | 46672 | trnS-GGA |
| Paphiopedilum_niveum | p2 | (AG)4 | 8 | 49595 | 49602 | trnL-UAA intron |
| Paphiopedilum_niveum | p2 | (TA)5 | 10 | 57268 | 57277 | atpB_rbcL |
| Paphiopedilum_niveum | p2 | (TA)4 | 8 | 57291 | 57298 | atpB_rbcL |
| Paphiopedilum_niveum | p2 | (TA)7 | 14 | 57300 | 57313 | atpB_rbcL |
| Paphiopedilum_niveum | p2 | (TA)4 | 8 | 57362 | 57369 | atpB_rbcL |
| Paphiopedilum_niveum | p2 | (AT)4 | 8 | 59727 | 59734 | rbcL_accD |
| Paphiopedilum_niveum | p2 | (TG)4 | 8 | 61034 | 61041 | accD |
| Paphiopedilum_niveum | p2 | (TA)4 | 8 | 63645 | 63652 | ycf4_cemA |
| Paphiopedilum_niveum | p2 | (AT)4 | 8 | 63661 | 63668 | ycf4_cemA |
| Paphiopedilum_niveum | p2 | (TC)4 | 8 | 64242 | 64249 | cemA |
| Paphiopedilum_niveum | p2 | (TA)4 | 8 | 66721 | 66728 | petA_psbJ |
| Paphiopedilum_niveum | p2 | (AT)7 | 14 | 69692 | 69705 | trnP-UGG_psaJ |
| Paphiopedilum_niveum | p2 | (AT)4 | 8 | 69977 | 69984 | trnP-UGG_psaJ |
| Paphiopedilum_niveum | p2 | (AT)4 | 8 | 70015 | 70022 | trnP-UGG_psaJ |
| Paphiopedilum_niveum | p2 | (AT)4 | 8 | 70037 | 70044 | trnP-UGG_psaJ |
| Paphiopedilum_niveum | p2 | (TA)4 | 8 | 71440 | 71447 | rpl33_rps18 |
| Paphiopedilum_niveum | p2 | (TA)4 | 8 | 71449 | 71456 | rpl33_rps18 |
| Paphiopedilum_niveum | p2 | (TA)8 | 16 | 71489 | 71504 | rpl33_rps18 |
| Paphiopedilum_niveum | p2 | (TA)4 | 8 | 71532 | 71539 | rpl33_rps18 |
| Paphiopedilum_niveum | p2 | (TA)4 | 8 | 71566 | 71573 | rpl33_rps18 |
| Paphiopedilum_niveum | p2 | (TA)7 | 14 | 74155 | 74168 | clpP intron 1 |
| Paphiopedilum_niveum | p2 | (TA)8 | 16 | 75421 | 75436 | clpP intron 2 |
| Paphiopedilum_niveum | p2 | (AT)5 | 10 | 75467 | 75476 | clpP intron 2 |
| Paphiopedilum_niveum | p2 | (AT)4 | 8 | 75864 | 75871 | clpP intron 2 |
| Paphiopedilum_niveum | p2 | (TA)5 | 10 | 76414 | 76423 | clpP_psbB |
| Paphiopedilum_niveum | p2 | (TA)4 | 8 | 76429 | 76436 | clpP_psbB |
| Paphiopedilum_niveum | p2 | (TA)4 | 8 | 76442 | 76449 | clpP_psbB |
| Paphiopedilum_niveum | p2 | (TA)5 | 10 | 76502 | 76511 | clpP_psbB |
| Paphiopedilum_niveum | p2 | (TA)7 | 14 | 76528 | 76541 | clpP_psbB |
| Paphiopedilum_niveum | p2 | (TA)4 | 8 | 76655 | 76662 | clpP_psbB |
| Paphiopedilum_niveum | p2 | (TA)7 | 14 | 78581 | 78594 | psbB_psbT |
| Paphiopedilum_niveum | p2 | (TA)4 | 8 | 78798 | 78805 | psbB_psbT |
| Paphiopedilum_niveum | p2 | (TA)4 | 8 | 78879 | 78886 | psbB_psbT |
| Paphiopedilum_niveum | p2 | (AT)7 | 14 | 78895 | 78908 | psbB_psbT |
| Paphiopedilum_niveum | p2 | (TA)4 | 8 | 78916 | 78923 | psbB_psbT |
| Paphiopedilum_niveum | p2 | (TA)6 | 12 | 78981 | 78992 | psbB_psbT |
| Paphiopedilum_niveum | p2 | (AT)4 | 8 | 81441 | 81448 | petB_petD |
| Paphiopedilum_niveum | p2 | (AT)4 | 8 | 89926 | 89933 | rpl22 |
| Paphiopedilum_niveum | p2 | (GA)4 | 8 | 92918 | 92925 | ycf2 |
| Paphiopedilum_niveum | p2 | (GA)4 | 8 | 93905 | 93912 | ycf2 |
| Paphiopedilum_niveum | p2 | (GA)5 | 10 | 95982 | 95991 | ycf2 |
| Paphiopedilum_niveum | p2 | (TA)4 | 8 | 99404 | 99411 | ycf2 |
| Paphiopedilum_niveum | p2 | (CT)4 | 8 | 100928 | 100935 | trnL-CAA_ndhB |
| Paphiopedilum_niveum | p2 | (AG)4 | 8 | 101625 | 101632 | ndhB intron |
| Paphiopedilum_niveum | p2 | (TA)6 | 12 | 106174 | 106185 | rps12_trnV-GAC |
| Paphiopedilum_niveum | p2 | (CT)4 | 8 | 113127 | 113134 | rrn23 |
| Paphiopedilum_niveum | p2 | (AG)4 | 8 | 125176 | 125183 | ndhD intron |
| Paphiopedilum_niveum | p2 | (AT)8 | 16 | 126385 | 126400 | psaC_rps15 |
| Paphiopedilum_niveum | p2 | (AG)4 | 8 | 135933 | 135940 | rrn23 |
| Paphiopedilum_niveum | p2 | (AT)6 | 12 | 142881 | 142892 | trnV-GAC_rps12 |
| Paphiopedilum_niveum | p2 | (CT)4 | 8 | 147435 | 147442 | ndhB intron |
| Paphiopedilum_niveum | p2 | (GA)4 | 8 | 148131 | 148138 | ndhB_trnL-CAA |
| Paphiopedilum_niveum | p2 | (TA)4 | 8 | 149656 | 149663 | ycf2 |
| Paphiopedilum_niveum | p2 | (TC)5 | 10 | 153076 | 153085 | ycf2 |
| Paphiopedilum_niveum | p2 | (TC)4 | 8 | 155155 | 155162 | ycf2 |
| Paphiopedilum_niveum | p2 | (TC)4 | 8 | 156142 | 156149 | ycf2 |
| Paphiopedilum_kolopakingii | p2 | (TA)4 | 8 | 3807 | 3814 | matK_rps16 |
| Paphiopedilum_kolopakingii | p2 | (TA)4 | 8 | 5844 | 5851 | rps16 intron |
| Paphiopedilum_kolopakingii | p2 | (AT)7 | 14 | 8841 | 8854 | trnS-GCU_trnG-UCC |
| Paphiopedilum_kolopakingii | p2 | (AT)4 | 8 | 9431 | 9438 | trnS-GCU_trnG-UCC |
| Paphiopedilum_kolopakingii | p2 | (TA)4 | 8 | 9460 | 9467 | trnS-GCU_trnG-UCC |
| Paphiopedilum_kolopakingii | p2 | (TA)5 | 10 | 9494 | 9503 | trnS-GCU_trnG-UCC |
| Paphiopedilum_kolopakingii | p2 | (TA)4 | 8 | 9745 | 9752 | trnS-GCU_trnG-UCC |
| Paphiopedilum_kolopakingii | p2 | (TA)4 | 8 | 9755 | 9762 | trnS-GCU_trnG-UCC |
| Paphiopedilum_kolopakingii | p2 | (AT)4 | 8 | 9852 | 9859 | trnS-GCU_trnG-UCC |
| Paphiopedilum_kolopakingii | p2 | (AT)4 | 8 | 11074 | 11081 | trnR-UCU_atpA |
| Paphiopedilum_kolopakingii | p2 | (AT)4 | 8 | 20784 | 20791 | rpoC2 |
| Paphiopedilum_kolopakingii | p2 | (AT)5 | 10 | 20874 | 20883 | rpoC2 |
| Paphiopedilum_kolopakingii | p2 | (AT)4 | 8 | 21909 | 21916 | rpoC1 |
| Paphiopedilum_kolopakingii | p2 | (AT)4 | 8 | 28046 | 28053 | rpoB_trnC-GCA |
| Paphiopedilum_kolopakingii | p2 | (TA)4 | 8 | 29405 | 29412 | trnC-GCA_petN |
| Paphiopedilum_kolopakingii | p2 | (AT)4 | 8 | 29415 | 29422 | trnC-GCA_petN |
| Paphiopedilum_kolopakingii | p2 | (AT)4 | 8 | 29455 | 29462 | trnC-GCA_petN |
| Paphiopedilum_kolopakingii | p2 | (AT)4 | 8 | 29507 | 29514 | trnC-GCA_petN |
| Paphiopedilum_kolopakingii | p2 | (AT)4 | 8 | 29547 | 29554 | trnC-GCA_petN |
| Paphiopedilum_kolopakingii | p2 | (AT)4 | 8 | 29565 | 29572 | trnC-GCA_petN |
| Paphiopedilum_kolopakingii | p2 | (AT)4 | 8 | 30174 | 30181 | petN_psbM |
| Paphiopedilum_kolopakingii | p2 | (AT)5 | 10 | 30265 | 30274 | petN_psbM |
| Paphiopedilum_kolopakingii | p2 | (TA)4 | 8 | 30279 | 30286 | petN_psbM |
| Paphiopedilum_kolopakingii | p2 | (TA)4 | 8 | 30298 | 30305 | petN_psbM |
| Paphiopedilum_kolopakingii | p2 | (TA)4 | 8 | 30498 | 30505 | petN_psbM |
| Paphiopedilum_kolopakingii | p2 | (AC)4 | 8 | 31284 | 31291 | psbM_trnD-GUC |
| Paphiopedilum_kolopakingii | p2 | (AT)4 | 8 | 32516 | 32523 | trnE-UUC_trnT-GGU |
| Paphiopedilum_kolopakingii | p2 | (TA)4 | 8 | 32543 | 32550 | trnE-UUC_trnT-GGU |
| Paphiopedilum_kolopakingii | p2 | (AT)4 | 8 | 32577 | 32584 | trnE-UUC_trnT-GGU |
| Paphiopedilum_kolopakingii | p2 | (TA)6 | 12 | 33386 | 33397 | trnE-UUC_trnT-GGU |
| Paphiopedilum_kolopakingii | p2 | (GA)4 | 8 | 37194 | 37201 | trnS-UGA |
| Paphiopedilum_kolopakingii | p2 | (CT)4 | 8 | 46639 | 46646 | trnS-GGA |
| Paphiopedilum_kolopakingii | p2 | (TA)4 | 8 | 47746 | 47753 | rps4_trnT-UGU |
| Paphiopedilum_kolopakingii | p2 | (TA)4 | 8 | 48235 | 48242 | trnT-UGU_trnL-UAA |
| Paphiopedilum_kolopakingii | p2 | (AG)4 | 8 | 49606 | 49613 | trnL-UAA intron |
| Paphiopedilum_kolopakingii | p2 | (TA)4 | 8 | 55744 | 55751 | atpB_rbcL |
| Paphiopedilum_kolopakingii | p2 | (TA)4 | 8 | 56184 | 56191 | atpB_rbcL |
| Paphiopedilum_kolopakingii | p2 | (AT)4 | 8 | 56229 | 56236 | atpB_rbcL |
| Paphiopedilum_kolopakingii | p2 | (TA)4 | 8 | 56240 | 56247 | atpB_rbcL |
| Paphiopedilum_kolopakingii | p2 | (TA)6 | 12 | 56321 | 56332 | atpB_rbcL |
| Paphiopedilum_kolopakingii | p2 | (TA)6 | 12 | 56356 | 56367 | atpB_rbcL |
| Paphiopedilum_kolopakingii | p2 | (AT)4 | 8 | 58699 | 58706 | rbcL_accD |
| Paphiopedilum_kolopakingii | p2 | (TG)4 | 8 | 60002 | 60009 | accD |
| Paphiopedilum_kolopakingii | p2 | (AT)4 | 8 | 62617 | 62624 | ycf4_cemA |
| Paphiopedilum_kolopakingii | p2 | (AT)5 | 10 | 68177 | 68186 | trnP-UGG_psaJ |
| Paphiopedilum_kolopakingii | p2 | (AT)5 | 10 | 68402 | 68411 | trnP-UGG_psaJ |
| Paphiopedilum_kolopakingii | p2 | (AT)5 | 10 | 68417 | 68426 | trnP-UGG_psaJ |
| Paphiopedilum_kolopakingii | p2 | (AT)4 | 8 | 68430 | 68437 | trnP-UGG_psaJ |
| Paphiopedilum_kolopakingii | p2 | (AT)4 | 8 | 68569 | 68576 | trnP-UGG_psaJ |
| Paphiopedilum_kolopakingii | p2 | (AT)4 | 8 | 69255 | 69262 | psaJ_rpl33 |
| Paphiopedilum_kolopakingii | p2 | (TA)4 | 8 | 69803 | 69810 | rpl33_rps18 |
| Paphiopedilum_kolopakingii | p2 | (TA)7 | 14 | 71910 | 71923 | clpP |
| Paphiopedilum_kolopakingii | p2 | (TA)5 | 10 | 72477 | 72486 | clpP intron 1 |
| Paphiopedilum_kolopakingii | p2 | (TA)8 | 16 | 73894 | 73909 | clpP intron 2 |
| Paphiopedilum_kolopakingii | p2 | (TA)4 | 8 | 73911 | 73918 | clpP intron 2 |
| Paphiopedilum_kolopakingii | p2 | (TA)4 | 8 | 73920 | 73927 | clpP intron 2 |
| Paphiopedilum_kolopakingii | p2 | (AT)4 | 8 | 74377 | 74384 | clpP intron 2 |
| Paphiopedilum_kolopakingii | p2 | (TA)5 | 10 | 74926 | 74935 | clpP_psbB |
| Paphiopedilum_kolopakingii | p2 | (TA)8 | 16 | 74938 | 74953 | clpP_psbB |
| Paphiopedilum_kolopakingii | p2 | (TA)4 | 8 | 74959 | 74966 | clpP_psbB |
| Paphiopedilum_kolopakingii | p2 | (TA)5 | 10 | 77038 | 77047 | psbB_psbT |
| Paphiopedilum_kolopakingii | p2 | (TA)5 | 10 | 77051 | 77060 | psbB_psbT |
| Paphiopedilum_kolopakingii | p2 | (TA)8 | 16 | 77301 | 77316 | psbB_psbT |
| Paphiopedilum_kolopakingii | p2 | (AT)4 | 8 | 79752 | 79759 | petB_petD |
| Paphiopedilum_kolopakingii | p2 | (AT)4 | 8 | 84481 | 84488 | rps8_rpl14 |
| Paphiopedilum_kolopakingii | p2 | (TA)4 | 8 | 86441 | 86448 | rpl16 intron |
| Paphiopedilum_kolopakingii | p2 | (TA)4 | 8 | 86469 | 86476 | rpl16 intron |
| Paphiopedilum_kolopakingii | p2 | (AT)4 | 8 | 88129 | 88136 | rpl22 |
| Paphiopedilum_kolopakingii | p2 | (GA)4 | 8 | 91086 | 91093 | ycf2 |
| Paphiopedilum_kolopakingii | p2 | (GA)4 | 8 | 92073 | 92080 | ycf2 |
| Paphiopedilum_kolopakingii | p2 | (GA)5 | 10 | 94294 | 94303 | ycf2 |
| Paphiopedilum_kolopakingii | p2 | (TA)4 | 8 | 97698 | 97705 | ycf2 |
| Paphiopedilum_kolopakingii | p2 | (CT)4 | 8 | 99239 | 99246 | trnL-CAA_ndhB |
| Paphiopedilum_kolopakingii | p2 | (AG)4 | 8 | 99936 | 99943 | ndhB |
| Paphiopedilum_kolopakingii | p2 | (TA)4 | 8 | 103802 | 103809 | rps12_trnV-GAC |
| Paphiopedilum_kolopakingii | p2 | (TA)7 | 14 | 103829 | 103842 | rps12_trnV-GAC |
| Paphiopedilum_kolopakingii | p2 | (TA)4 | 8 | 103854 | 103861 | rps12_trnV-GAC |
| Paphiopedilum_kolopakingii | p2 | (TA)5 | 10 | 103865 | 103874 | rps12_trnV-GAC |
| Paphiopedilum_kolopakingii | p2 | (TA)7 | 14 | 103876 | 103889 | rps12_trnV-GAC |
| Paphiopedilum_kolopakingii | p2 | (TA)4 | 8 | 104123 | 104130 | rps12_trnV-GAC |
| Paphiopedilum_kolopakingii | p2 | (CT)4 | 8 | 110998 | 111005 | rrn23 |
| Paphiopedilum_kolopakingii | p2 | (AT)7 | 14 | 120385 | 120398 | rps15_psaC |
| Paphiopedilum_kolopakingii | p2 | (TC)4 | 8 | 121540 | 121547 | ndhD_trnL-UAG |
| Paphiopedilum_kolopakingii | p2 | (TA)4 | 8 | 123213 | 123220 | ndhD_trnL-UAG |
| Paphiopedilum_kolopakingii | p2 | (TA)4 | 8 | 123229 | 123236 | ndhD_trnL-UAG |
| Paphiopedilum_kolopakingii | p2 | (TA)4 | 8 | 123249 | 123256 | ndhD_trnL-UAG |
| Paphiopedilum_kolopakingii | p2 | (AG)4 | 8 | 126459 | 126466 | ccsA_ndhD |
| Paphiopedilum_kolopakingii | p2 | (AT)7 | 14 | 127609 | 127622 | psaC_rps15 |
| Paphiopedilum_kolopakingii | p2 | (AG)4 | 8 | 137002 | 137009 | rrn23 |
| Paphiopedilum_kolopakingii | p2 | (AT)4 | 8 | 143876 | 143883 | trnV-GAC_rps12 |
| Paphiopedilum_kolopakingii | p2 | (AT)5 | 10 | 144105 | 144114 | trnV-GAC_rps12 |
| Paphiopedilum_kolopakingii | p2 | (TA)4 | 8 | 144119 | 144126 | trnV-GAC_rps12 |
| Paphiopedilum_kolopakingii | p2 | (AT)7 | 14 | 144137 | 144150 | trnV-GAC_rps12 |
| Paphiopedilum_kolopakingii | p2 | (AT)4 | 8 | 144170 | 144177 | trnV-GAC_rps12 |
| Paphiopedilum_kolopakingii | p2 | (CT)4 | 8 | 148064 | 148071 | ndhB |
| Paphiopedilum_kolopakingii | p2 | (GA)4 | 8 | 148760 | 148767 | ndhB_trnL-CAA |
| Paphiopedilum_kolopakingii | p2 | (TA)4 | 8 | 150302 | 150309 | ycf2 |
| Paphiopedilum_kolopakingii | p2 | (TC)5 | 10 | 153704 | 153713 | ycf2 |
| Paphiopedilum_kolopakingii | p2 | (TC)4 | 8 | 155927 | 155934 | ycf2 |
| Paphiopedilum_kolopakingii | p2 | (TC)4 | 8 | 156914 | 156921 | ycf2 |
| Paphiopedilum_hirsutissimum | p2 | (TA)4 | 8 | 3716 | 3723 | trnK-UUU intron |
| Paphiopedilum_hirsutissimum | p2 | (TA)4 | 8 | 3733 | 3740 | trnK-UUU intron |
| Paphiopedilum_hirsutissimum | p2 | (AT)4 | 8 | 9269 | 9276 | trnS-GCU_trnG-UCC |
| Paphiopedilum_hirsutissimum | p2 | (TA)4 | 8 | 9349 | 9356 | trnS-GCU_trnG-UCC |
| Paphiopedilum_hirsutissimum | p2 | (TA)4 | 8 | 9403 | 9410 | trnS-GCU_trnG-UCC |
| Paphiopedilum_hirsutissimum | p2 | (TA)4 | 8 | 9415 | 9422 | trnS-GCU_trnG-UCC |
| Paphiopedilum_hirsutissimum | p2 | (TA)4 | 8 | 9432 | 9439 | trnS-GCU_trnG-UCC |
| Paphiopedilum_hirsutissimum | p2 | (TA)4 | 8 | 9450 | 9457 | trnS-GCU_trnG-UCC |
| Paphiopedilum_hirsutissimum | p2 | (TA)7 | 14 | 10774 | 10787 | trnR-UCU_atpA |
| Paphiopedilum_hirsutissimum | p2 | (AT)4 | 8 | 20352 | 20359 | rpoC2 |
| Paphiopedilum_hirsutissimum | p2 | (AT)5 | 10 | 20442 | 20451 | rpoC2 |
| Paphiopedilum_hirsutissimum | p2 | (AT)4 | 8 | 21477 | 21484 | rpoC1 |
| Paphiopedilum_hirsutissimum | p2 | (AT)4 | 8 | 27618 | 27625 | rpoB_trnC-GCA |
| Paphiopedilum_hirsutissimum | p2 | (TA)6 | 12 | 28998 | 29009 | trnC-GCA_petN |
| Paphiopedilum_hirsutissimum | p2 | (AT)4 | 8 | 29041 | 29048 | trnC-GCA_petN |
| Paphiopedilum_hirsutissimum | p2 | (TA)4 | 8 | 29715 | 29722 | petN_psbM |
| Paphiopedilum_hirsutissimum | p2 | (AC)4 | 8 | 30496 | 30503 | psbM_trnD-GUC |
| Paphiopedilum_hirsutissimum | p2 | (AT)4 | 8 | 31690 | 31697 | trnE-UUC_trnT-GGU |
| Paphiopedilum_hirsutissimum | p2 | (TA)4 | 8 | 31744 | 31751 | trnE-UUC_trnT-GGU |
| Paphiopedilum_hirsutissimum | p2 | (GA)4 | 8 | 35745 | 35752 | trnS-UGA |
| Paphiopedilum_hirsutissimum | p2 | (CT)4 | 8 | 45212 | 45219 | trnS-GGA |
| Paphiopedilum_hirsutissimum | p2 | (TA)4 | 8 | 46321 | 46328 | rps4_trnT-UGU |
| Paphiopedilum_hirsutissimum | p2 | (AG)4 | 8 | 47682 | 47689 | trnL-UAA intron |
| Paphiopedilum_hirsutissimum | p2 | (TA)4 | 8 | 53498 | 53505 | atpB_rbcL |
| Paphiopedilum_hirsutissimum | p2 | (TA)4 | 8 | 53543 | 53550 | atpB_rbcL |
| Paphiopedilum_hirsutissimum | p2 | (TA)6 | 12 | 53611 | 53622 | atpB_rbcL |
| Paphiopedilum_hirsutissimum | p2 | (TA)4 | 8 | 53624 | 53631 | atpB_rbcL |
| Paphiopedilum_hirsutissimum | p2 | (AT)4 | 8 | 55562 | 55569 | rbcL_accD |
| Paphiopedilum_hirsutissimum | p2 | (AT)4 | 8 | 55980 | 55987 | rbcL_accD |
| Paphiopedilum_hirsutissimum | p2 | (TG)4 | 8 | 57287 | 57294 | accD |
| Paphiopedilum_hirsutissimum | p2 | (TC)4 | 8 | 59908 | 59915 | ycf4_cemA |
| Paphiopedilum_hirsutissimum | p2 | (AT)5 | 10 | 65334 | 65343 | trnP-UGG_psaJ |
| Paphiopedilum_hirsutissimum | p2 | (TA)9 | 18 | 65469 | 65486 | trnP-UGG_psaJ |
| Paphiopedilum_hirsutissimum | p2 | (AT)4 | 8 | 66376 | 66383 | psaJ_rpl33 |
| Paphiopedilum_hirsutissimum | p2 | (TA)4 | 8 | 66887 | 66894 | rpl33_rps18 |
| Paphiopedilum_hirsutissimum | p2 | (TA)6 | 12 | 69547 | 69558 | clpP intron 1 |
| Paphiopedilum_hirsutissimum | p2 | (AT)4 | 8 | 70885 | 70892 | clpP intron 2 |
| Paphiopedilum_hirsutissimum | p2 | (AT)4 | 8 | 71228 | 71235 | clpP intron 2 |
| Paphiopedilum_hirsutissimum | p2 | (TA)4 | 8 | 71500 | 71507 | clpP_psbB |
| Paphiopedilum_hirsutissimum | p2 | (AT)8 | 16 | 73439 | 73454 | psbB_psbT |
| Paphiopedilum_hirsutissimum | p2 | (TA)6 | 12 | 73580 | 73591 | psbB_psbT |
| Paphiopedilum_hirsutissimum | p2 | (AT)4 | 8 | 76066 | 76073 | petB_petD |
| Paphiopedilum_hirsutissimum | p2 | (AT)4 | 8 | 84338 | 84345 | rpl22 |
| Paphiopedilum_hirsutissimum | p2 | (GA)4 | 8 | 87337 | 87344 | ycf2 |
| Paphiopedilum_hirsutissimum | p2 | (GA)4 | 8 | 88324 | 88331 | ycf2 |
| Paphiopedilum_hirsutissimum | p2 | (GA)5 | 10 | 90407 | 90416 | ycf2 |
| Paphiopedilum_hirsutissimum | p2 | (TA)4 | 8 | 93847 | 93854 | ycf2 |
| Paphiopedilum_hirsutissimum | p2 | (CT)4 | 8 | 94497 | 94504 | trnL-CAA_ndhB |
| Paphiopedilum_hirsutissimum | p2 | (AG)4 | 8 | 95206 | 95213 | ndhB |
| Paphiopedilum_hirsutissimum | p2 | (TA)7 | 14 | 99806 | 99819 | rps12_ycf15 |
| Paphiopedilum_hirsutissimum | p2 | (CT)4 | 8 | 106673 | 106680 | rrn23 |
| Paphiopedilum_hirsutissimum | p2 | (TC)4 | 8 | 117300 | 117307 | ndhD intron |
| Paphiopedilum_hirsutissimum | p2 | (AG)4 | 8 | 122655 | 122662 | ndhD intron |
| Paphiopedilum_hirsutissimum | p2 | (AG)4 | 8 | 133289 | 133296 | rrn23 |
| Paphiopedilum_hirsutissimum | p2 | (AT)7 | 14 | 140149 | 140162 | ycf15_rps12 |
| Paphiopedilum_hirsutissimum | p2 | (CT)4 | 8 | 144756 | 144763 | ndhB |
| Paphiopedilum_hirsutissimum | p2 | (GA)4 | 8 | 145464 | 145471 | ndhB_trnL-CAA |
| Paphiopedilum_hirsutissimum | p2 | (TA)4 | 8 | 146115 | 146122 | ycf2 |
| Paphiopedilum_hirsutissimum | p2 | (TC)5 | 10 | 149553 | 149562 | ycf2 |
| Paphiopedilum_hirsutissimum | p2 | (TC)4 | 8 | 151638 | 151645 | ycf2 |
| Paphiopedilum_hirsutissimum | p2 | (TC)4 | 8 | 152625 | 152632 | ycf2 |
| Paphiopedilum_philippinense | p2 | (TA)4 | 8 | 3816 | 3823 | trnK-UUU intron |
| Paphiopedilum_philippinense | p2 | (AT)9 | 18 | 8734 | 8751 | trnS-GCU_trnG-UCC |
| Paphiopedilum_philippinense | p2 | (AT)4 | 8 | 9302 | 9309 | trnS-GCU_trnG-UCC |
| Paphiopedilum_philippinense | p2 | (AT)4 | 8 | 9312 | 9319 | trnS-GCU_trnG-UCC |
| Paphiopedilum_philippinense | p2 | (TA)4 | 8 | 9375 | 9382 | trnS-GCU_trnG-UCC |
| Paphiopedilum_philippinense | p2 | (TA)6 | 12 | 9406 | 9417 | trnS-GCU_trnG-UCC |
| Paphiopedilum_philippinense | p2 | (TA)6 | 12 | 9457 | 9468 | trnS-GCU_trnG-UCC |
| Paphiopedilum_philippinense | p2 | (TA)7 | 14 | 9509 | 9522 | trnS-GCU_trnG-UCC |
| Paphiopedilum_philippinense | p2 | (AT)4 | 8 | 10834 | 10841 | trnR-UCU_atpA |
| Paphiopedilum_philippinense | p2 | (AT)4 | 8 | 20539 | 20546 | rpoC2 |
| Paphiopedilum_philippinense | p2 | (AT)5 | 10 | 20629 | 20638 | rpoC2 |
| Paphiopedilum_philippinense | p2 | (AT)4 | 8 | 21664 | 21671 | rpoC1 |
| Paphiopedilum_philippinense | p2 | (AT)4 | 8 | 27803 | 27810 | rpoB_trnC-GCA |
| Paphiopedilum_philippinense | p2 | (TA)5 | 10 | 29037 | 29046 | trnC-GCA_petN |
| Paphiopedilum_philippinense | p2 | (AT)4 | 8 | 29058 | 29065 | trnC-GCA_petN |
| Paphiopedilum_philippinense | p2 | (AT)4 | 8 | 29103 | 29110 | trnC-GCA_petN |
| Paphiopedilum_philippinense | p2 | (AT)4 | 8 | 29487 | 29494 | petN_psbM |
| Paphiopedilum_philippinense | p2 | (AT)4 | 8 | 29568 | 29575 | petN_psbM |
| Paphiopedilum_philippinense | p2 | (TA)4 | 8 | 29685 | 29692 | petN_psbM |
| Paphiopedilum_philippinense | p2 | (TA)4 | 8 | 29766 | 29773 | petN_psbM |
| Paphiopedilum_philippinense | p2 | (AC)4 | 8 | 30566 | 30573 | psbM_trnD-GUC |
| Paphiopedilum_philippinense | p2 | (AT)5 | 10 | 31605 | 31614 | trnE-UUC_trnT-GGU |
| Paphiopedilum_philippinense | p2 | (AT)4 | 8 | 31776 | 31783 | trnE-UUC_trnT-GGU |
| Paphiopedilum_philippinense | p2 | (TA)7 | 14 | 31811 | 31824 | trnE-UUC_trnT-GGU |
| Paphiopedilum_philippinense | p2 | (AT)5 | 10 | 31861 | 31870 | trnE-UUC_trnT-GGU |
| Paphiopedilum_philippinense | p2 | (TA)5 | 10 | 31896 | 31905 | trnE-UUC_trnT-GGU |
| Paphiopedilum_philippinense | p2 | (TA)7 | 14 | 32535 | 32548 | trnE-UUC_trnT-GGU |
| Paphiopedilum_philippinense | p2 | (GA)4 | 8 | 36349 | 36356 | trnS-UGA |
| Paphiopedilum_philippinense | p2 | (CT)4 | 8 | 45811 | 45818 | trnS-GGA |
| Paphiopedilum_philippinense | p2 | (TA)4 | 8 | 46919 | 46926 | rps4_trnT-UGU |
| Paphiopedilum_philippinense | p2 | (TA)4 | 8 | 47378 | 47385 | trnT-UGU_trnL-UAA |
| Paphiopedilum_philippinense | p2 | (AG)4 | 8 | 48765 | 48772 | trnL-UAA intron |
| Paphiopedilum_philippinense | p2 | (TA)4 | 8 | 54852 | 54859 | atpB_rbcL |
| Paphiopedilum_philippinense | p2 | (TA)4 | 8 | 55060 | 55067 | atpB_rbcL |
| Paphiopedilum_philippinense | p2 | (TA)5 | 10 | 55111 | 55120 | atpB_rbcL |
| Paphiopedilum_philippinense | p2 | (TA)5 | 10 | 55129 | 55138 | atpB_rbcL |
| Paphiopedilum_philippinense | p2 | (AT)4 | 8 | 57442 | 57449 | rbcL_accD |
| Paphiopedilum_philippinense | p2 | (TG)4 | 8 | 58760 | 58767 | accD |
| Paphiopedilum_philippinense | p2 | (AT)4 | 8 | 61388 | 61395 | ycf4_cemA |
| Paphiopedilum_philippinense | p2 | (AT)4 | 8 | 61403 | 61410 | ycf4_cemA |
| Paphiopedilum_philippinense | p2 | (AT)9 | 18 | 66815 | 66832 | trnP-UGG_psaJ |
| Paphiopedilum_philippinense | p2 | (AT)5 | 10 | 66988 | 66997 | trnP-UGG_psaJ |
| Paphiopedilum_philippinense | p2 | (AT)4 | 8 | 67001 | 67008 | trnP-UGG_psaJ |
| Paphiopedilum_philippinense | p2 | (AT)4 | 8 | 67012 | 67019 | trnP-UGG_psaJ |
| Paphiopedilum_philippinense | p2 | (AT)4 | 8 | 67025 | 67032 | trnP-UGG_psaJ |
| Paphiopedilum_philippinense | p2 | (TA)4 | 8 | 67131 | 67138 | trnP-UGG_psaJ |
| Paphiopedilum_philippinense | p2 | (AT)4 | 8 | 67838 | 67845 | psaJ_rpl33 |
| Paphiopedilum_philippinense | p2 | (TA)4 | 8 | 68423 | 68430 | rpl33_rps18 |
| Paphiopedilum_philippinense | p2 | (TA)7 | 14 | 70510 | 70523 | clpP |
| Paphiopedilum_philippinense | p2 | (TA)5 | 10 | 71077 | 71086 | clpP intron 1 |
| Paphiopedilum_philippinense | p2 | (TA)5 | 10 | 71092 | 71101 | clpP intron 1 |
| Paphiopedilum_philippinense | p2 | (TA)5 | 10 | 71105 | 71114 | clpP intron 1 |
| Paphiopedilum_philippinense | p2 | (AT)4 | 8 | 71179 | 71186 | clpP intron 1 |
| Paphiopedilum_philippinense | p2 | (TA)4 | 8 | 72530 | 72537 | clpP intron 2 |
| Paphiopedilum_philippinense | p2 | (TA)4 | 8 | 72551 | 72558 | clpP intron 2 |
| Paphiopedilum_philippinense | p2 | (TA)4 | 8 | 72625 | 72632 | clpP intron 2 |
| Paphiopedilum_philippinense | p2 | (TA)4 | 8 | 72636 | 72643 | clpP intron 2 |
| Paphiopedilum_philippinense | p2 | (AT)4 | 8 | 73032 | 73039 | clpP intron 2 |
| Paphiopedilum_philippinense | p2 | (TA)4 | 8 | 73408 | 73415 | clpP_psbB |
| Paphiopedilum_philippinense | p2 | (TA)5 | 10 | 73446 | 73455 | clpP_psbB |
| Paphiopedilum_philippinense | p2 | (TA)15 | 30 | 73458 | 73487 | clpP_psbB |
| Paphiopedilum_philippinense | p2 | (TA)4 | 8 | 73493 | 73500 | clpP_psbB |
| Paphiopedilum_philippinense | p2 | (TA)4 | 8 | 73518 | 73525 | clpP_psbB |
| Paphiopedilum_philippinense | p2 | (AT)4 | 8 | 73590 | 73597 | clpP_psbB |
| Paphiopedilum_philippinense | p2 | (TA)4 | 8 | 73679 | 73686 | clpP_psbB |
| Paphiopedilum_philippinense | p2 | (TA)7 | 14 | 75605 | 75618 | psbB_psbT |
| Paphiopedilum_philippinense | p2 | (TA)4 | 8 | 75630 | 75637 | psbB_psbT |
| Paphiopedilum_philippinense | p2 | (TA)10 | 20 | 75887 | 75906 | psbB_psbT |
| Paphiopedilum_philippinense | p2 | (AT)4 | 8 | 75974 | 75981 | psbB_psbT |
| Paphiopedilum_philippinense | p2 | (AT)4 | 8 | 78339 | 78346 | petB_petD |
| Paphiopedilum_philippinense | p2 | (AT)4 | 8 | 83164 | 83171 | rps8_rpl14 |
| Paphiopedilum_philippinense | p2 | (TA)6 | 12 | 85008 | 85019 | rpl16 intron |
| Paphiopedilum_philippinense | p2 | (TA)4 | 8 | 85021 | 85028 | rpl16 intron |
| Paphiopedilum_philippinense | p2 | (AT)4 | 8 | 86806 | 86813 | rpl22 |
| Paphiopedilum_philippinense | p2 | (GA)4 | 8 | 89763 | 89770 | ycf2 |
| Paphiopedilum_philippinense | p2 | (GA)4 | 8 | 90750 | 90757 | ycf2 |
| Paphiopedilum_philippinense | p2 | (GA)5 | 10 | 92971 | 92980 | ycf2 |
| Paphiopedilum_philippinense | p2 | (TA)4 | 8 | 96393 | 96400 | ycf2 |
| Paphiopedilum_philippinense | p2 | (CT)4 | 8 | 97946 | 97953 | trnL-CAA_ndhB |
| Paphiopedilum_philippinense | p2 | (AG)4 | 8 | 98643 | 98650 | ndhB |
| Paphiopedilum_philippinense | p2 | (TA)4 | 8 | 102743 | 102750 | rps12_trnV-GAC |
| Paphiopedilum_philippinense | p2 | (CT)4 | 8 | 109595 | 109602 | rrn23 |
| Paphiopedilum_philippinense | p2 | (AT)7 | 14 | 119055 | 119068 | rps15_psaC |
| Paphiopedilum_philippinense | p2 | (TC)4 | 8 | 120210 | 120217 | ndhD_trnL-UAG |
| Paphiopedilum_philippinense | p2 | (TA)4 | 8 | 121935 | 121942 | ndhD_trnL-UAG |
| Paphiopedilum_philippinense | p2 | (TA)4 | 8 | 122498 | 122505 | trnL-UAG_rpl32 |
| Paphiopedilum_philippinense | p2 | (TA)7 | 14 | 122524 | 122537 | trnL-UAG_rpl32 |
| Paphiopedilum_philippinense | p2 | (AG)4 | 8 | 125273 | 125280 | ccsA_ndhD |
| Paphiopedilum_philippinense | p2 | (AT)7 | 14 | 126423 | 126436 | psaC_rps15 |
| Paphiopedilum_philippinense | p2 | (AG)4 | 8 | 135889 | 135896 | rrn23 |
| Paphiopedilum_philippinense | p2 | (AT)4 | 8 | 142730 | 142737 | trnV-GAC_rps12 |
| Paphiopedilum_philippinense | p2 | (CT)4 | 8 | 146841 | 146848 | ndhB |
| Paphiopedilum_philippinense | p2 | (GA)4 | 8 | 147537 | 147544 | ndhB_trnL-CAA |
| Paphiopedilum_philippinense | p2 | (TA)4 | 8 | 149091 | 149098 | ycf2 |
| Paphiopedilum_philippinense | p2 | (TC)5 | 10 | 152511 | 152520 | ycf2 |
| Paphiopedilum_philippinense | p2 | (TC)4 | 8 | 154734 | 154741 | ycf2 |
| Paphiopedilum_philippinense | p2 | (TC)4 | 8 | 155721 | 155728 | ycf2 |
| Paphiopedilum_emersonii | p3 | (TTA)4 | 12 | 3623 | 3634 | trnK-UUU intron |
| Paphiopedilum_emersonii | p3 | (ATT)4 | 12 | 9383 | 9394 | trnS-GCU_trnG-UCC |
| Paphiopedilum_emersonii | p3 | (TCT)4 | 12 | 15767 | 15778 | atpI_rps2 |
| Paphiopedilum_emersonii | p3 | (ATA)4 | 12 | 28609 | 28620 | trnC-GCA_petN |
| Paphiopedilum_emersonii | p3 | (TAT)4 | 12 | 29186 | 29197 | petN_psbM |
| Paphiopedilum_emersonii | p3 | (TAT)4 | 12 | 30001 | 30012 | petN_psbM |
| Paphiopedilum_emersonii | p3 | (ATA)4 | 12 | 53968 | 53979 | atpB_rbcL |
| Paphiopedilum_emersonii | p3 | (TAA)4 | 12 | 68165 | 68176 | psaJ_rpl33 |
| Paphiopedilum_emersonii | p3 | (ATT)4 | 12 | 68692 | 68703 | rpl33_rps18 |
| Paphiopedilum_emersonii | p3 | (TAT)4 | 12 | 73588 | 73599 | clpP_psbB |
| Paphiopedilum_emersonii | p3 | (TTA)4 | 12 | 75809 | 75820 | psbB_psbT |
| Paphiopedilum_emersonii | p3 | (ATA)5 | 15 | 83186 | 83200 | rps8_rpl14 |
| Paphiopedilum_emersonii | p3 | (ATT)4 | 12 | 84768 | 84779 | rpl16 intron |
| Paphiopedilum_emersonii | p3 | (ATA)4 | 12 | 111394 | 111405 | trnN-GUU_ycf1 |
| Paphiopedilum_emersonii | p3 | (AGA)4 | 12 | 113297 | 113308 | ycf1 |
| Paphiopedilum_emersonii | p3 | (AAT)4 | 12 | 121774 | 121785 | rpl32_ccsA |
| Paphiopedilum_emersonii | p3 | (TCT)4 | 12 | 129647 | 129658 | ycf1 |
| Paphiopedilum_emersonii | p3 | (ATT)4 | 12 | 131548 | 131559 | ycf1_trnN-GUU |
| Paphiopedilum_victoria-mariae | p3 | (TTA)4 | 12 | 3644 | 3655 | trnK-UUU intron |
| Paphiopedilum_victoria-mariae | p3 | (TAT)4 | 12 | 9495 | 9506 | trnS-GCU_trnG-UCC |
| Paphiopedilum_victoria-mariae | p3 | (ATT)4 | 12 | 9790 | 9801 | trnS-GCU_trnG-UCC |
| Paphiopedilum_victoria-mariae | p3 | (TCT)4 | 12 | 16606 | 16617 | atpI_rps2 |
| Paphiopedilum_victoria-mariae | p3 | (ATA)4 | 12 | 29520 | 29531 | trnC-GCA_petN |
| Paphiopedilum_victoria-mariae | p3 | (ATA)4 | 12 | 29537 | 29548 | trnC-GCA_petN |
| Paphiopedilum_victoria-mariae | p3 | (TAT)4 | 12 | 31252 | 31263 | petN_psbM |
| Paphiopedilum_victoria-mariae | p3 | (TAT)6 | 18 | 31272 | 31289 | petN_psbM |
| Paphiopedilum_victoria-mariae | p3 | (ATA)4 | 12 | 56615 | 56626 | atpB_rbcL |
| Paphiopedilum_victoria-mariae | p3 | (ATA)4 | 12 | 69968 | 69979 | trnP-UGG_psaJ |
| Paphiopedilum_victoria-mariae | p3 | (ATA)4 | 12 | 69990 | 70001 | trnP-UGG_psaJ |
| Paphiopedilum_victoria-mariae | p3 | (TAA)4 | 12 | 70657 | 70668 | psaJ_rpl33 |
| Paphiopedilum_victoria-mariae | p3 | (TAT)4 | 12 | 76192 | 76203 | clpP_psbB |
| Paphiopedilum_victoria-mariae | p3 | (TAT)4 | 12 | 76209 | 76220 | clpP_psbB |
| Paphiopedilum_victoria-mariae | p3 | (TAT)5 | 15 | 78382 | 78396 | psbB_psbT |
| Paphiopedilum_victoria-mariae | p3 | (AAT)4 | 12 | 85739 | 85750 | rps8_rpl14 |
| Paphiopedilum_victoria-mariae | p3 | (ATT)4 | 12 | 87438 | 87449 | rpl16 intron |
| Paphiopedilum_victoria-mariae | p3 | (ATA)5 | 15 | 87581 | 87595 | rpl16 intron |
| Paphiopedilum_victoria-mariae | p3 | (AGA)4 | 12 | 116782 | 116793 | ycf1 |
| Paphiopedilum_victoria-mariae | p3 | (ATA)4 | 12 | 124808 | 124819 | ndhD_trnL-UAG |
| Paphiopedilum_victoria-mariae | p3 | (TCT)4 | 12 | 133871 | 133882 | ycf1 |
| Paphiopedilum_armeniacum | p3 | (ATT)4 | 12 | 9973 | 9984 | trnS-GCU_trnG-UCC |
| Paphiopedilum_armeniacum | p3 | (TCT)4 | 12 | 16692 | 16703 | atpI_rps2 |
| Paphiopedilum_armeniacum | p3 | (TTA)4 | 12 | 30054 | 30065 | trnC-GCA_petN |
| Paphiopedilum_armeniacum | p3 | (TAT)4 | 12 | 30549 | 30560 | petN_psbM |
| Paphiopedilum_armeniacum | p3 | (TAT)4 | 12 | 30726 | 30737 | petN_psbM |
| Paphiopedilum_armeniacum | p3 | (ATA)4 | 12 | 58048 | 58059 | atpB_rbcL |
| Paphiopedilum_armeniacum | p3 | (TAA)5 | 15 | 72685 | 72699 | psaJ_rpl33 |
| Paphiopedilum_armeniacum | p3 | (AAT)4 | 12 | 87978 | 87989 | rps8_rpl14 |
| Paphiopedilum_armeniacum | p3 | (AAT)4 | 12 | 87993 | 88004 | rps8_rpl14 |
| Paphiopedilum_armeniacum | p3 | (ATT)4 | 12 | 89807 | 89818 | rpl16 intron |
| Paphiopedilum_armeniacum | p3 | (TAT)4 | 12 | 89970 | 89981 | rpl16 intron |
| Paphiopedilum_armeniacum | p3 | (AGA)4 | 12 | 119776 | 119787 | ycf1 |
| Paphiopedilum_armeniacum | p3 | (TTA)4 | 12 | 126775 | 126786 | trnL-UAG_ccsA |
| Paphiopedilum_armeniacum | p3 | (TAT)4 | 12 | 126802 | 126813 | trnL-UAG_ccsA |
| Paphiopedilum_armeniacum | p3 | (TCT)4 | 12 | 134838 | 134849 | ycf1 |
| Paphiopedilum_dianthum | p3 | (ATT)4 | 12 | 9207 | 9218 | trnS-GCU_trnG-UCC |
| Paphiopedilum_dianthum | p3 | (TCT)4 | 12 | 15935 | 15946 | atpI_rps2 |
| Paphiopedilum_dianthum | p3 | (TAT)4 | 12 | 29359 | 29370 | petN_psbM |
| Paphiopedilum_dianthum | p3 | (ATT)4 | 12 | 29471 | 29482 | petN_psbM |
| Paphiopedilum_dianthum | p3 | (ATA)4 | 12 | 54244 | 54255 | atpB_rbcL |
| Paphiopedilum_dianthum | p3 | (TAT)4 | 12 | 55049 | 55060 | atpB_rbcL |
| Paphiopedilum_dianthum | p3 | (ATA)4 | 12 | 67145 | 67156 | trnP-UGG_psaJ |
| Paphiopedilum_dianthum | p3 | (TAA)4 | 12 | 67717 | 67728 | trnP-UGG_psaJ |
| Paphiopedilum_dianthum | p3 | (TAA)5 | 15 | 68500 | 68514 | psaJ_rpl33 |
| Paphiopedilum_dianthum | p3 | (ATA)4 | 12 | 69176 | 69187 | rpl33_rps18 |
| Paphiopedilum_dianthum | p3 | (TTA)4 | 12 | 74070 | 74081 | clpP_psbB |
| Paphiopedilum_dianthum | p3 | (AAT)4 | 12 | 83337 | 83348 | rps8_rpl14 |
| Paphiopedilum_dianthum | p3 | (ATT)4 | 12 | 85023 | 85034 | rpl16 intron |
| Paphiopedilum_dianthum | p3 | (AGA)4 | 12 | 113840 | 113851 | ycf1 |
| Paphiopedilum_dianthum | p3 | (TCT)4 | 12 | 127710 | 127721 | ycf1 |
| Paphiopedilum_violascens | p3 | (TAT)4 | 12 | 4960 | 4971 | matK_rps16 |
| Paphiopedilum_violascens | p3 | (ATT)4 | 12 | 12973 | 12984 | atpF intron |
| Paphiopedilum_violascens | p3 | (TCT)4 | 12 | 15754 | 15765 | atpI_rps2 |
| Paphiopedilum_violascens | p3 | (ATA)4 | 12 | 28675 | 28686 | trnC-GCA_petN |
| Paphiopedilum_violascens | p3 | (TTA)6 | 18 | 29317 | 29334 | petN_psbM |
| Paphiopedilum_violascens | p3 | (TAT)4 | 12 | 30038 | 30049 | petN_psbM |
| Paphiopedilum_violascens | p3 | (TAT)4 | 12 | 30182 | 30193 | petN_psbM |
| Paphiopedilum_violascens | p3 | (ATT)4 | 12 | 30197 | 30208 | petN_psbM |
| Paphiopedilum_violascens | p3 | (ATT)7 | 21 | 32560 | 32580 | trnE-UUC_trnT-GGU |
| Paphiopedilum_violascens | p3 | (ATA)4 | 12 | 55513 | 55524 | atpB_rbcL |
| Paphiopedilum_violascens | p3 | (TAT)4 | 12 | 67864 | 67875 | trnP-UGG_psaJ |
| Paphiopedilum_violascens | p3 | (TAT)4 | 12 | 67883 | 67894 | trnP-UGG_psaJ |
| Paphiopedilum_violascens | p3 | (ATA)4 | 12 | 68237 | 68248 | trnP-UGG_psaJ |
| Paphiopedilum_violascens | p3 | (ATA)6 | 18 | 68313 | 68330 | trnP-UGG_psaJ |
| Paphiopedilum_violascens | p3 | (TAA)4 | 12 | 68886 | 68897 | psaJ_rpl33 |
| Paphiopedilum_violascens | p3 | (ATA)6 | 18 | 73670 | 73687 | clpP intron 2 |
| Paphiopedilum_violascens | p3 | (TTA)4 | 12 | 74526 | 74537 | clpP_psbB |
| Paphiopedilum_violascens | p3 | (ATT)4 | 12 | 85736 | 85747 | rpl16 intron |
| Paphiopedilum_violascens | p3 | (AGA)4 | 12 | 114390 | 114401 | ycf1 |
| Paphiopedilum_violascens | p3 | (ATA)4 | 12 | 122074 | 122085 | ndhD_trnL-UAG |
| Paphiopedilum_violascens | p3 | (TCT)4 | 12 | 131127 | 131138 | ycf1 |
| Paphiopedilum_niveum | p3 | (TAT)4 | 12 | 5070 | 5081 | matK_rps16 |
| Paphiopedilum_niveum | p3 | (TCT)4 | 12 | 16685 | 16696 | atpI_rps2 |
| Paphiopedilum_niveum | p3 | (ATA)4 | 12 | 29612 | 29623 | trnC-GCA_petN |
| Paphiopedilum_niveum | p3 | (ATT)5 | 15 | 32379 | 32393 | trnE-UUC_trnT-GGU |
| Paphiopedilum_niveum | p3 | (ATA)5 | 15 | 63627 | 63641 | ycf4_cemA |
| Paphiopedilum_niveum | p3 | (AAT)4 | 12 | 69734 | 69745 | trnP-UGG_psaJ |
| Paphiopedilum_niveum | p3 | (ATA)4 | 12 | 70037 | 70048 | trnP-UGG_psaJ |
| Paphiopedilum_niveum | p3 | (ATA)5 | 15 | 70067 | 70081 | trnP-UGG_psaJ |
| Paphiopedilum_niveum | p3 | (AAT)4 | 12 | 70139 | 70150 | trnP-UGG_psaJ |
| Paphiopedilum_niveum | p3 | (TAA)4 | 12 | 70893 | 70904 | psaJ_rpl33 |
| Paphiopedilum_niveum | p3 | (ATA)4 | 12 | 75467 | 75478 | clpP intron 2 |
| Paphiopedilum_niveum | p3 | (TAT)4 | 12 | 76345 | 76356 | clpP_psbB |
| Paphiopedilum_niveum | p3 | (TAT)4 | 12 | 78657 | 78668 | psbB_psbT |
| Paphiopedilum_niveum | p3 | (AAT)4 | 12 | 86224 | 86235 | rps8_rpl14 |
| Paphiopedilum_niveum | p3 | (ATT)4 | 12 | 87957 | 87968 | rpl16 intron |
| Paphiopedilum_niveum | p3 | (AGA)4 | 12 | 117912 | 117923 | ycf1 |
| Paphiopedilum_niveum | p3 | (TCT)4 | 12 | 131144 | 131155 | ycf1 |
| Paphiopedilum_kolopakingii | p3 | (TTA)4 | 12 | 3634 | 3645 | matK_rps16 |
| Paphiopedilum_kolopakingii | p3 | (TTA)4 | 12 | 3652 | 3663 | matK_rps16 |
| Paphiopedilum_kolopakingii | p3 | (TAT)5 | 15 | 3685 | 3699 | matK_rps16 |
| Paphiopedilum_kolopakingii | p3 | (TAT)4 | 12 | 5860 | 5871 | rps16 intron |
| Paphiopedilum_kolopakingii | p3 | (ATT)4 | 12 | 9805 | 9816 | trnS-GCU_trnG-UCC |
| Paphiopedilum_kolopakingii | p3 | (ATA)4 | 12 | 11120 | 11131 | trnR-UCU_atpA |
| Paphiopedilum_kolopakingii | p3 | (TCT)4 | 12 | 16525 | 16536 | atpI_rps2 |
| Paphiopedilum_kolopakingii | p3 | (TAT)4 | 12 | 30233 | 30244 | petN_psbM |
| Paphiopedilum_kolopakingii | p3 | (TAT)4 | 12 | 30349 | 30360 | petN_psbM |
| Paphiopedilum_kolopakingii | p3 | (ATA)4 | 12 | 55292 | 55303 | atpB_rbcL |
| Paphiopedilum_kolopakingii | p3 | (TAA)4 | 12 | 69198 | 69209 | psaJ_rpl33 |
| Paphiopedilum_kolopakingii | p3 | (TAT)4 | 12 | 74890 | 74901 | clpP_psbB |
| Paphiopedilum_kolopakingii | p3 | (TAT)5 | 15 | 77085 | 77099 | psbB_psbT |
| Paphiopedilum_kolopakingii | p3 | (AAT)4 | 12 | 84511 | 84522 | rps8_rpl14 |
| Paphiopedilum_kolopakingii | p3 | (ATT)4 | 12 | 86194 | 86205 | rpl16 intron |
| Paphiopedilum_kolopakingii | p3 | (AAT)4 | 12 | 86425 | 86436 | rpl16 intron |
| Paphiopedilum_kolopakingii | p3 | (AAT)4 | 12 | 86453 | 86464 | rpl16 intron |
| Paphiopedilum_kolopakingii | p3 | (AGA)4 | 12 | 115795 | 115806 | ycf1 |
| Paphiopedilum_kolopakingii | p3 | (TCT)4 | 12 | 132201 | 132212 | ycf1 |
| Paphiopedilum_hirsutissimum | p3 | (TTA)4 | 12 | 3574 | 3585 | trnK-UUU intron |
| Paphiopedilum_hirsutissimum | p3 | (TTA)4 | 12 | 3590 | 3601 | trnK-UUU intron |
| Paphiopedilum_hirsutissimum | p3 | (AAT)4 | 12 | 4565 | 4576 | matK_rps16 |
| Paphiopedilum_hirsutissimum | p3 | (ATA)4 | 12 | 4954 | 4965 | matK_rps16 |
| Paphiopedilum_hirsutissimum | p3 | (ATT)4 | 12 | 9490 | 9501 | trnS-GCU_trnG-UCC |
| Paphiopedilum_hirsutissimum | p3 | (TCT)4 | 12 | 16088 | 16099 | atpI_rps2 |
| Paphiopedilum_hirsutissimum | p3 | (ATA)4 | 12 | 29041 | 29052 | trnC-GCA_petN |
| Paphiopedilum_hirsutissimum | p3 | (ATT)5 | 15 | 31831 | 31845 | trnE-UUC_trnT-GGU |
| Paphiopedilum_hirsutissimum | p3 | (ATT)4 | 12 | 35719 | 35730 | psbC_trnS-UGA |
| Paphiopedilum_hirsutissimum | p3 | (ATA)4 | 12 | 52773 | 52784 | atpB_rbcL |
| Paphiopedilum_hirsutissimum | p3 | (TAA)4 | 12 | 66335 | 66346 | psaJ_rpl33 |
| Paphiopedilum_hirsutissimum | p3 | (AAT)4 | 12 | 80832 | 80843 | rps8_rpl14 |
| Paphiopedilum_hirsutissimum | p3 | (ATT)4 | 12 | 82509 | 82520 | rpl16 intron |
| Paphiopedilum_hirsutissimum | p3 | (AGA)4 | 12 | 111423 | 111434 | ycf1 |
| Paphiopedilum_hirsutissimum | p3 | (TCT)4 | 12 | 128535 | 128546 | ycf1 |
| Paphiopedilum_philippinense | p3 | (TTA)4 | 12 | 3603 | 3614 | trnK-UUU intron |
| Paphiopedilum_philippinense | p3 | (ATA)4 | 12 | 8775 | 8786 | trnS-GCU_trnG-UCC |
| Paphiopedilum_philippinense | p3 | (TTA)4 | 12 | 9580 | 9591 | trnS-GCU_trnG-UCC |
| Paphiopedilum_philippinense | p3 | (TCT)4 | 12 | 16281 | 16292 | atpI_rps2 |
| Paphiopedilum_philippinense | p3 | (TAT)5 | 15 | 29525 | 29539 | petN_psbM |
| Paphiopedilum_philippinense | p3 | (TAT)4 | 12 | 29631 | 29642 | petN_psbM |
| Paphiopedilum_philippinense | p3 | (ATA)4 | 12 | 54415 | 54426 | atpB_rbcL |
| Paphiopedilum_philippinense | p3 | (TAA)4 | 12 | 67797 | 67808 | psaJ_rpl33 |
| Paphiopedilum_philippinense | p3 | (AAT)4 | 12 | 83086 | 83097 | rps8_rpl14 |
| Paphiopedilum_philippinense | p3 | (ATT)4 | 12 | 84818 | 84829 | rpl16 intron |
| Paphiopedilum_philippinense | p3 | (TAT)4 | 12 | 84921 | 84932 | rpl16 intron |
| Paphiopedilum_philippinense | p3 | (AAT)4 | 12 | 84969 | 84980 | rpl16 intron |
| Paphiopedilum_philippinense | p3 | (ATA)5 | 15 | 84982 | 84996 | rpl16 intron |
| Paphiopedilum_philippinense | p3 | (AGA)4 | 12 | 114456 | 114467 | ycf1 |
| Paphiopedilum_philippinense | p3 | (TAT)4 | 12 | 122851 | 122862 | trnL-UAG_rpl32 |
| Paphiopedilum_philippinense | p3 | (TCT)4 | 12 | 131024 | 131035 | ycf1 |
| Paphiopedilum_emersonii | p4 | (ATTT)3 | 12 | 3644 | 3655 | trnK-UUU intron |
| Paphiopedilum_emersonii | p4 | (TAAA)3 | 12 | 4787 | 4798 | matK_rps16 |
| Paphiopedilum_emersonii | p4 | (GTCT)3 | 12 | 11844 | 11855 | atpA |
| Paphiopedilum_emersonii | p4 | (TAAA)3 | 12 | 47421 | 47432 | trnT-UGU_trnL-UAA |
| Paphiopedilum_emersonii | p4 | (TTAT)3 | 12 | 54512 | 54523 | atpB_rbcL |
| Paphiopedilum_emersonii | p4 | (CTTT)3 | 12 | 57240 | 57251 | rbcL_accD |
| Paphiopedilum_emersonii | p4 | (AATG)3 | 12 | 62056 | 62067 | cemA |
| Paphiopedilum_emersonii | p4 | (GATA)4 | 16 | 75870 | 75885 | psbB_psbT |
| Paphiopedilum_emersonii | p4 | (TAAT)4 | 16 | 83161 | 83176 | rps8_rpl14 |
| Paphiopedilum_emersonii | p4 | (ACAT)3 | 12 | 86430 | 86441 | rpl22 |
| Paphiopedilum_emersonii | p4 | (TCTA)3 | 12 | 111352 | 111363 | trnN-GUU_ycf1 |
| Paphiopedilum_emersonii | p4 | (ATTC)3 | 12 | 115445 | 115456 | ycf1 |
| Paphiopedilum_emersonii | p4 | (AAAT)3 | 12 | 116300 | 116311 | ycf1 |
| Paphiopedilum_emersonii | p4 | (TTTA)3 | 12 | 121434 | 121445 | trnL-UAG_rpl32 |
| Paphiopedilum_emersonii | p4 | (TATT)3 | 12 | 126635 | 126646 | ycf1 |
| Paphiopedilum_emersonii | p4 | (GAAT)3 | 12 | 127499 | 127510 | ycf1 |
| Paphiopedilum_emersonii | p4 | (ATAG)3 | 12 | 131591 | 131602 | ycf1_trnN-GUU |
| Paphiopedilum_victoria-mariae | p4 | (TAAA)3 | 12 | 5061 | 5072 | matK_rps16 |
| Paphiopedilum_victoria-mariae | p4 | (GTCT)3 | 12 | 12333 | 12344 | atpA |
| Paphiopedilum_victoria-mariae | p4 | (TTAT)3 | 12 | 57138 | 57149 | atpB_rbcL |
| Paphiopedilum_victoria-mariae | p4 | (CTTT)3 | 12 | 59910 | 59921 | rbcL_accD |
| Paphiopedilum_victoria-mariae | p4 | (AATG)3 | 12 | 64630 | 64641 | cemA |
| Paphiopedilum_victoria-mariae | p4 | (AAAT)3 | 12 | 70143 | 70154 | trnP-UGG_psaJ |
| Paphiopedilum_victoria-mariae | p4 | (GATA)3 | 12 | 78486 | 78497 | psbB_psbT |
| Paphiopedilum_victoria-mariae | p4 | (TTAT)3 | 12 | 87383 | 87394 | rpl16 intron |
| Paphiopedilum_victoria-mariae | p4 | (ACAT)3 | 12 | 89334 | 89345 | rpl22 |
| Paphiopedilum_victoria-mariae | p4 | (TTAT)4 | 16 | 105194 | 105209 | rps12_trnV-GAC |
| Paphiopedilum_victoria-mariae | p4 | (TCTA)3 | 12 | 114735 | 114746 | trnN-GUU_ycf1 |
| Paphiopedilum_victoria-mariae | p4 | (ATTC)3 | 12 | 118828 | 118839 | ycf1 |
| Paphiopedilum_victoria-mariae | p4 | (TTTA)3 | 12 | 125455 | 125466 | trnL-UAG_rpl32 |
| Paphiopedilum_victoria-mariae | p4 | (TTAT)4 | 16 | 125863 | 125878 | rpl32_ccsA |
| Paphiopedilum_victoria-mariae | p4 | (GAAT)3 | 12 | 131825 | 131836 | ycf1 |
| Paphiopedilum_victoria-mariae | p4 | (ATAG)3 | 12 | 135917 | 135928 | ycf1_trnN-GUU |
| Paphiopedilum_victoria-mariae | p4 | (AATA)4 | 16 | 145454 | 145469 | trnV-GAC_rps12 |
| Paphiopedilum_armeniacum | p4 | (AATA)4 | 16 | 4981 | 4996 | matK_rps16 |
| Paphiopedilum_armeniacum | p4 | (AAAT)3 | 12 | 5296 | 5307 | matK_rps16 |
| Paphiopedilum_armeniacum | p4 | (TTAT)3 | 12 | 9973 | 9984 | trnS-GCU_trnG-UCC |
| Paphiopedilum_armeniacum | p4 | (GTCT)3 | 12 | 12651 | 12662 | atpA |
| Paphiopedilum_armeniacum | p4 | (TTAT)3 | 12 | 58614 | 58625 | atpB_rbcL |
| Paphiopedilum_armeniacum | p4 | (TATT)3 | 12 | 58861 | 58872 | atpB_rbcL |
| Paphiopedilum_armeniacum | p4 | (CTTT)3 | 12 | 61329 | 61340 | rbcL_accD |
| Paphiopedilum_armeniacum | p4 | (AATG)3 | 12 | 66673 | 66684 | cemA |
| Paphiopedilum_armeniacum | p4 | (AAAT)3 | 12 | 72119 | 72130 | trnP-UGG_psaJ |
| Paphiopedilum_armeniacum | p4 | (TATT)4 | 16 | 80727 | 80742 | psbB_psbT |
| Paphiopedilum_armeniacum | p4 | (TTAT)3 | 12 | 89724 | 89735 | rpl16 intron |
| Paphiopedilum_armeniacum | p4 | (AATA)3 | 12 | 91754 | 91765 | rpl22 |
| Paphiopedilum_armeniacum | p4 | (ACAT)3 | 12 | 91930 | 91941 | rpl22 |
| Paphiopedilum_armeniacum | p4 | (TCTA)3 | 12 | 117729 | 117740 | trnN-GUU_ycf1 |
| Paphiopedilum_armeniacum | p4 | (ATTC)3 | 12 | 121807 | 121818 | ycf1 |
| Paphiopedilum_armeniacum | p4 | (TTAT)3 | 12 | 124249 | 124260 | rps15_psaC |
| Paphiopedilum_armeniacum | p4 | (AAAT)3 | 12 | 130356 | 130367 | psaC_rps15 |
| Paphiopedilum_armeniacum | p4 | (GAAT)3 | 12 | 132807 | 132818 | ycf1 |
| Paphiopedilum_armeniacum | p4 | (ATAG)3 | 12 | 136884 | 136895 | ycf1_trnN-GUU |
| Paphiopedilum_dianthum | p4 | (GTCT)3 | 12 | 11674 | 11685 | atpA |
| Paphiopedilum_dianthum | p4 | (ATTT)4 | 16 | 29452 | 29467 | petN_psbM |
| Paphiopedilum_dianthum | p4 | (CTTT)3 | 12 | 57614 | 57625 | rbcL_accD |
| Paphiopedilum_dianthum | p4 | (AATG)3 | 12 | 62329 | 62340 | cemA intron |
| Paphiopedilum_dianthum | p4 | (AAAT)3 | 12 | 67981 | 67992 | trnP-UGG_psaJ |
| Paphiopedilum_dianthum | p4 | (TAGA)3 | 12 | 76114 | 76125 | psbB_psbT |
| Paphiopedilum_dianthum | p4 | (AATA)3 | 12 | 83337 | 83348 | rps8_rpl14 |
| Paphiopedilum_dianthum | p4 | (TTAT)3 | 12 | 84968 | 84979 | rpl16 intron |
| Paphiopedilum_dianthum | p4 | (ACAT)3 | 12 | 86832 | 86843 | rpl22 |
| Paphiopedilum_dianthum | p4 | (TTAT)4 | 16 | 101734 | 101749 | rps12_trnV-GAC |
| Paphiopedilum_dianthum | p4 | (TCTA)3 | 12 | 111880 | 111891 | trnN-GUU_ycf1 |
| Paphiopedilum_dianthum | p4 | (ATTC)3 | 12 | 115892 | 115903 | ycf1 |
| Paphiopedilum_dianthum | p4 | (TAAT)3 | 12 | 119412 | 119423 | trnL-UAG_rpl32 |
| Paphiopedilum_dianthum | p4 | (ATTA)3 | 12 | 122138 | 122149 | ndhD_psaC |
| Paphiopedilum_dianthum | p4 | (GAAT)3 | 12 | 125658 | 125669 | ycf1 |
| Paphiopedilum_dianthum | p4 | (ATAG)3 | 12 | 129669 | 129680 | ycf1_trnN-GUU |
| Paphiopedilum_dianthum | p4 | (AATA)4 | 16 | 139811 | 139826 | trnV-GAC_rps12 |
| Paphiopedilum_violascens | p4 | (TAAA)4 | 16 | 4960 | 4975 | matK_rps16 |
| Paphiopedilum_violascens | p4 | (GTCT)3 | 12 | 11481 | 11492 | atpA |
| Paphiopedilum_violascens | p4 | (TTAT)3 | 12 | 32560 | 32571 | trnE-UUC_trnT-GGU |
| Paphiopedilum_violascens | p4 | (AAAT)3 | 12 | 32806 | 32817 | trnE-UUC_trnT-GGU |
| Paphiopedilum_violascens | p4 | (AAAT)3 | 12 | 48829 | 48840 | trnT-UGU_trnL-UAA |
| Paphiopedilum_violascens | p4 | (TTAT)3 | 12 | 56059 | 56070 | atpB_rbcL |
| Paphiopedilum_violascens | p4 | (CTTT)3 | 12 | 58764 | 58775 | rbcL_accD |
| Paphiopedilum_violascens | p4 | (AATA)4 | 16 | 68261 | 68276 | trnP-UGG_psaJ |
| Paphiopedilum_violascens | p4 | (AAAT)3 | 12 | 68366 | 68377 | trnP-UGG_psaJ |
| Paphiopedilum_violascens | p4 | (TTAT)4 | 16 | 72225 | 72240 | clpP intron 1 |
| Paphiopedilum_violascens | p4 | (TTAT)3 | 12 | 72257 | 72268 | clpP intron 1 |
| Paphiopedilum_violascens | p4 | (TTAT)3 | 12 | 85674 | 85685 | rpl16 intron |
| Paphiopedilum_violascens | p4 | (ACAT)3 | 12 | 87645 | 87656 | rpl22 |
| Paphiopedilum_violascens | p4 | (TTAT)4 | 16 | 102852 | 102867 | rps12_trnV-GAC |
| Paphiopedilum_violascens | p4 | (TCTA)3 | 12 | 112307 | 112318 | trnN-GUU_ycf1 |
| Paphiopedilum_violascens | p4 | (ATTC)3 | 12 | 116418 | 116429 | ycf1 |
| Paphiopedilum_violascens | p4 | (TTTA)3 | 12 | 122800 | 122811 | trnL-UAG_rpl32 |
| Paphiopedilum_violascens | p4 | (GAAT)3 | 12 | 129099 | 129110 | ycf1 |
| Paphiopedilum_violascens | p4 | (ATAG)3 | 12 | 133209 | 133220 | ycf1_trnN-GUU |
| Paphiopedilum_violascens | p4 | (AATA)4 | 16 | 142660 | 142675 | trnV-GAC_rps12 |
| Paphiopedilum_niveum | p4 | (TATG)3 | 12 | 16 | 27 | rps19_psbA |
| Paphiopedilum_niveum | p4 | (TATT)3 | 12 | 3628 | 3639 | trnK-UUU intron |
| Paphiopedilum_niveum | p4 | (GTCT)3 | 12 | 12434 | 12445 | atpA |
| Paphiopedilum_niveum | p4 | (TTAT)3 | 12 | 32444 | 32455 | trnE-UUC_trnT-GGU |
| Paphiopedilum_niveum | p4 | (TTAT)3 | 12 | 57046 | 57057 | atpB_rbcL |
| Paphiopedilum_niveum | p4 | (CTTT)3 | 12 | 59781 | 59792 | rbcL_accD |
| Paphiopedilum_niveum | p4 | (AATG)3 | 12 | 64856 | 64867 | cemA |
| Paphiopedilum_niveum | p4 | (AAAT)3 | 12 | 70355 | 70366 | trnP-UGG_psaJ |
| Paphiopedilum_niveum | p4 | (TAAT)4 | 16 | 71510 | 71525 | rpl33_rps18 |
| Paphiopedilum_niveum | p4 | (ATAG)3 | 12 | 78943 | 78954 | psbB_psbT |
| Paphiopedilum_niveum | p4 | (ATAG)3 | 12 | 78994 | 79005 | psbB_psbT |
| Paphiopedilum_niveum | p4 | (TAAT)4 | 16 | 86258 | 86273 | rps8_rpl14 |
| Paphiopedilum_niveum | p4 | (TTAT)3 | 12 | 87902 | 87913 | rpl16 intron |
| Paphiopedilum_niveum | p4 | (ACAT)3 | 12 | 89926 | 89937 | rpl22 |
| Paphiopedilum_niveum | p4 | (TCTA)3 | 12 | 115856 | 115867 | trnN-GUU_ycf1 |
| Paphiopedilum_niveum | p4 | (ATTC)3 | 12 | 119937 | 119948 | ycf1 |
| Paphiopedilum_niveum | p4 | (TTAT)3 | 12 | 122649 | 122660 | trnL-UAG_rpl32 |
| Paphiopedilum_niveum | p4 | (TTTA)3 | 12 | 122715 | 122726 | trnL-UAG_rpl32 |
| Paphiopedilum_niveum | p4 | (GAAT)3 | 12 | 129119 | 129130 | ycf1 |
| Paphiopedilum_niveum | p4 | (ATAG)3 | 12 | 133199 | 133210 | ycf1_trnN-GUU |
| Paphiopedilum_kolopakingii | p4 | (TAAA)3 | 12 | 5021 | 5032 | matK_rps16 |
| Paphiopedilum_kolopakingii | p4 | (ATTA)4 | 16 | 5072 | 5087 | matK_rps16 |
| Paphiopedilum_kolopakingii | p4 | (TTAT)3 | 12 | 9829 | 9840 | trnS-GCU_trnG-UCC |
| Paphiopedilum_kolopakingii | p4 | (GTCT)3 | 12 | 12274 | 12285 | atpA |
| Paphiopedilum_kolopakingii | p4 | (TTAT)4 | 16 | 30349 | 30364 | petN_psbM |
| Paphiopedilum_kolopakingii | p4 | (CTTT)3 | 12 | 58753 | 58764 | rbcL_accD |
| Paphiopedilum_kolopakingii | p4 | (AATG)3 | 12 | 63341 | 63352 | cemA_petA |
| Paphiopedilum_kolopakingii | p4 | (ATAA)3 | 12 | 68516 | 68527 | trnP-UGG_psaJ |
| Paphiopedilum_kolopakingii | p4 | (AATA)3 | 12 | 68533 | 68544 | trnP-UGG_psaJ |
| Paphiopedilum_kolopakingii | p4 | (AAAT)3 | 12 | 68666 | 68677 | trnP-UGG_psaJ |
| Paphiopedilum_kolopakingii | p4 | (GATA)3 | 12 | 77301 | 77312 | psbB_psbT |
| Paphiopedilum_kolopakingii | p4 | (TTAT)3 | 12 | 86146 | 86157 | rpl16 intron |
| Paphiopedilum_kolopakingii | p4 | (TTAT)3 | 12 | 86363 | 86374 | rpl16 intron |
| Paphiopedilum_kolopakingii | p4 | (ACAT)3 | 12 | 88129 | 88140 | rpl22 |
| Paphiopedilum_kolopakingii | p4 | (TTAT)3 | 12 | 103741 | 103752 | rps12_trnV-GAC |
| Paphiopedilum_kolopakingii | p4 | (TCTA)3 | 12 | 113748 | 113759 | trnN-GUU_ycf1 |
| Paphiopedilum_kolopakingii | p4 | (ATTC)3 | 12 | 117841 | 117852 | ycf1 |
| Paphiopedilum_kolopakingii | p4 | (GAAT)3 | 12 | 130155 | 130166 | ycf1 |
| Paphiopedilum_kolopakingii | p4 | (ATAG)3 | 12 | 134247 | 134258 | ycf1_trnN-GUU |
| Paphiopedilum_kolopakingii | p4 | (AATA)3 | 12 | 144227 | 144238 | trnV-GAC_rps12 |
| Paphiopedilum_hirsutissimum | p4 | (TAAA)3 | 12 | 4954 | 4965 | matK_rps16 |
| Paphiopedilum_hirsutissimum | p4 | (GTCT)3 | 12 | 11981 | 11992 | atpA |
| Paphiopedilum_hirsutissimum | p4 | (TTAT)3 | 12 | 31831 | 31842 | trnE-UUC_trnT-GGU |
| Paphiopedilum_hirsutissimum | p4 | (TTAT)3 | 12 | 53298 | 53309 | atpB_rbcL |
| Paphiopedilum_hirsutissimum | p4 | (CTTT)3 | 12 | 56034 | 56045 | rbcL_accD |
| Paphiopedilum_hirsutissimum | p4 | (AAAT)4 | 16 | 65824 | 65839 | trnP-UGG_psaJ |
| Paphiopedilum_hirsutissimum | p4 | (TATT)3 | 12 | 69525 | 69536 | clpP intron 1 |
| Paphiopedilum_hirsutissimum | p4 | (AAAT)3 | 12 | 70937 | 70948 | clpP intron 2 |
| Paphiopedilum_hirsutissimum | p4 | (AATT)3 | 12 | 80867 | 80878 | rps8_rpl14 |
| Paphiopedilum_hirsutissimum | p4 | (TAAT)3 | 12 | 80880 | 80891 | rps8_rpl14 |
| Paphiopedilum_hirsutissimum | p4 | (ACAT)3 | 12 | 84338 | 84349 | rpl22 |
| Paphiopedilum_hirsutissimum | p4 | (TTAT)4 | 16 | 99734 | 99749 | rps12_ycf15 |
| Paphiopedilum_hirsutissimum | p4 | (TCTA)3 | 12 | 109349 | 109360 | trnN-GUU_ycf1 |
| Paphiopedilum_hirsutissimum | p4 | (ATTC)3 | 12 | 113559 | 113570 | ycf1 |
| Paphiopedilum_hirsutissimum | p4 | (AAAT)3 | 12 | 119702 | 119713 | rpl32_trnL-UAG |
| Paphiopedilum_hirsutissimum | p4 | (TTTA)3 | 12 | 120247 | 120258 | trnL-UAG_rpl32 |
| Paphiopedilum_hirsutissimum | p4 | (GAAT)3 | 12 | 126393 | 126404 | ycf1 |
| Paphiopedilum_hirsutissimum | p4 | (ATAG)3 | 12 | 130608 | 130619 | ycf1_trnN-GUU |
| Paphiopedilum_hirsutissimum | p4 | (AATA)4 | 16 | 140219 | 140234 | ycf15_rps12 |
| Paphiopedilum_philippinense | p4 | (TTAT)3 | 12 | 3648 | 3659 | trnK-UUU intron |
| Paphiopedilum_philippinense | p4 | (TAAA)3 | 12 | 5039 | 5050 | matK_rps16 |
| Paphiopedilum_philippinense | p4 | (GTCT)3 | 12 | 12008 | 12019 | atpA |
| Paphiopedilum_philippinense | p4 | (ATTT)3 | 12 | 29631 | 29642 | petN_psbM |
| Paphiopedilum_philippinense | p4 | (CTTT)3 | 12 | 57496 | 57507 | rbcL_accD |
| Paphiopedilum_philippinense | p4 | (AATG)3 | 12 | 62142 | 62153 | cemA_petA |
| Paphiopedilum_philippinense | p4 | (ATAA)3 | 12 | 67117 | 67128 | trnP-UGG_psaJ |
| Paphiopedilum_philippinense | p4 | (ATAA)3 | 12 | 67145 | 67156 | trnP-UGG_psaJ |
| Paphiopedilum_philippinense | p4 | (ATAA)3 | 12 | 67165 | 67176 | trnP-UGG_psaJ |
| Paphiopedilum_philippinense | p4 | (AAAT)3 | 12 | 67278 | 67289 | trnP-UGG_psaJ |
| Paphiopedilum_philippinense | p4 | (GATA)3 | 12 | 75887 | 75898 | psbB_psbT |
| Paphiopedilum_philippinense | p4 | (TTAT)3 | 12 | 84770 | 84781 | rpl16 intron |
| Paphiopedilum_philippinense | p4 | (ACAT)3 | 12 | 86806 | 86817 | rpl22 |
| Paphiopedilum_philippinense | p4 | (TTAT)6 | 24 | 102440 | 102463 | rps12_trnV-GAC |
| Paphiopedilum_philippinense | p4 | (TCTA)3 | 12 | 112409 | 112420 | trnN-GUU_ycf1 |
| Paphiopedilum_philippinense | p4 | (ATTC)3 | 12 | 116508 | 116519 | ycf1 |
| Paphiopedilum_philippinense | p4 | (GAAT)3 | 12 | 128972 | 128983 | ycf1 |
| Paphiopedilum_philippinense | p4 | (ATAG)3 | 12 | 133070 | 133081 | ycf1_trnN-GUU |
| Paphiopedilum_philippinense | p4 | (AATA)6 | 24 | 143027 | 143050 | trnV-GAC_rps12 |
| Paphiopedilum_emersonii | p5 | (TTATA)3 | 15 | 3694 | 3708 | trnK-UUU intron |
| Paphiopedilum_emersonii | p5 | (ATAAT)3 | 15 | 28609 | 28623 | trnC-GCA_petN |
| Paphiopedilum_emersonii | p5 | (TTATA)3 | 15 | 29186 | 29200 | petN_psbM |
| Paphiopedilum_emersonii | p5 | (TATTG)3 | 15 | 54768 | 54782 | atpB_rbcL |
| Paphiopedilum_emersonii | p5 | (ATATA)3 | 15 | 60666 | 60680 | ycf4_cemA |
| Paphiopedilum_victoria-mariae | p5 | (TTATA)3 | 15 | 9582 | 9596 | trnS-GCU_trnG-UCC |
| Paphiopedilum_victoria-mariae | p5 | (ATTAT)3 | 15 | 30155 | 30169 | petN_psbM |
| Paphiopedilum_victoria-mariae | p5 | (TATAT)3 | 15 | 30382 | 30396 | petN_psbM |
| Paphiopedilum_victoria-mariae | p5 | (TATAA)3 | 15 | 63815 | 63829 | ycf4_cemA |
| Paphiopedilum_victoria-mariae | p5 | (TATAT)5 | 25 | 124520 | 124544 | ndhD_trnL-UAG |
| Paphiopedilum_armeniacum | p5 | (TATAT)3 | 15 | 31291 | 31305 | petN_psbM |
| Paphiopedilum_armeniacum | p5 | (TATAC)3 | 15 | 58614 | 58628 | atpB_rbcL |
| Paphiopedilum_armeniacum | p5 | (ATTTA)3 | 15 | 89807 | 89821 | rpl16 intron |
| Paphiopedilum_armeniacum | p5 | (AAAAT)3 | 15 | 92109 | 92123 | rpl22_rps19 |
| Paphiopedilum_armeniacum | p5 | (TATTT)3 | 15 | 162501 | 162515 | rps19_ |
| Paphiopedilum_dianthum | p5 | (TATAT)3 | 15 | 55049 | 55063 | atpB_rbcL |
| Paphiopedilum_dianthum | p5 | (TTATA)3 | 15 | 61597 | 61611 | ycf4_cemA |
| Paphiopedilum_violascens | p5 | (TATAC)3 | 15 | 3457 | 3471 | trnK-UUU intron |
| Paphiopedilum_violascens | p5 | (AAATA)3 | 15 | 4960 | 4974 | matK_rps16 |
| Paphiopedilum_violascens | p5 | (AATAT)4 | 20 | 27941 | 27960 | rpoB_trnC-GCA |
| Paphiopedilum_violascens | p5 | (ATATA)3 | 15 | 68284 | 68298 | trnP-UGG_psaJ |
| Paphiopedilum_violascens | p5 | (ATTAT)3 | 15 | 73670 | 73684 | clpP intron 2 |
| Paphiopedilum_violascens | p5 | (TTATA)3 | 15 | 73687 | 73701 | clpP intron 2 |
| Paphiopedilum_violascens | p5 | (ATATT)3 | 15 | 76946 | 76960 | psbB_psbT |
| Paphiopedilum_violascens | p5 | (TATAT)3 | 15 | 85843 | 85857 | rpl16 intron |
| Paphiopedilum_violascens | p5 | (ATATT)3 | 15 | 86014 | 86028 | rpl16 intron |
| Paphiopedilum_niveum | p5 | (ATAAT)3 | 15 | 5139 | 5153 | matK_rps16 |
| Paphiopedilum_niveum | p5 | (ATAAT)3 | 15 | 5171 | 5185 | matK_rps16 |
| Paphiopedilum_niveum | p5 | (TATTT)3 | 15 | 10080 | 10094 | trnS-GCU_trnG-GCC |
| Paphiopedilum_niveum | p5 | (TATAC)3 | 15 | 57098 | 57112 | atpB_rbcL |
| Paphiopedilum_niveum | p5 | (TATTG)3 | 15 | 57291 | 57305 | atpB_rbcL |
| Paphiopedilum_niveum | p5 | (TAAAA)3 | 15 | 69781 | 69795 | trnP-UGG_psaJ |
| Paphiopedilum_niveum | p5 | (ATATT)3 | 15 | 88121 | 88135 | rpl16 intron |
| Paphiopedilum_niveum | p5 | (GGTCT)3 | 15 | 101119 | 101133 | trnL-CAA_ndhB |
| Paphiopedilum_niveum | p5 | (CCAGA)3 | 15 | 147932 | 147946 | ndhB_trnL-CAA |
| Paphiopedilum_kolopakingii | p5 | (ATATA)3 | 15 | 32577 | 32591 | trnE-UUC_trnT-GGU |
| Paphiopedilum_kolopakingii | p5 | (AAATA)3 | 15 | 33897 | 33911 | trnT-GGU_psbD |
| Paphiopedilum_kolopakingii | p5 | (TTTTA)3 | 15 | 65272 | 65286 | petA_psbJ |
| Paphiopedilum_kolopakingii | p5 | (ATCAT)3 | 15 | 73860 | 73874 | clpP intron 2 |
| Paphiopedilum_kolopakingii | p5 | (ATATT)3 | 15 | 103876 | 103890 | rps12_trnV-GAC |
| Paphiopedilum_kolopakingii | p5 | (ATATT)3 | 15 | 103926 | 103940 | rps12_trnV-GAC |
| Paphiopedilum_kolopakingii | p5 | (TAATA)3 | 15 | 144038 | 144052 | trnV-GAC_rps12 |
| Paphiopedilum_kolopakingii | p5 | (TAATA)3 | 15 | 144088 | 144102 | trnV-GAC_rps12 |
| Paphiopedilum_kolopakingii | p5 | (TAATA)3 | 15 | 144105 | 144119 | trnV-GAC_rps12 |
| Paphiopedilum_hirsutissimum | p5 | (TATAC)3 | 15 | 3408 | 3422 | trnK-UUU intron |
| Paphiopedilum_hirsutissimum | p5 | (AAATA)3 | 15 | 4954 | 4968 | matK_rps16 |
| Paphiopedilum_hirsutissimum | p5 | (ATAAT)3 | 15 | 4970 | 4984 | matK_rps16 |
| Paphiopedilum_hirsutissimum | p5 | (ATTTT)3 | 15 | 44886 | 44900 | ycf3_trnS-GGA |
| Paphiopedilum_hirsutissimum | p5 | (TTATA)4 | 20 | 53559 | 53578 | atpB_rbcL |
| Paphiopedilum_hirsutissimum | p5 | (TTATA)4 | 20 | 53582 | 53601 | atpB_rbcL |
| Paphiopedilum_hirsutissimum | p5 | (ATATT)3 | 15 | 82637 | 82651 | rpl16 intron |
| Paphiopedilum_philippinense | p5 | (TTATA)3 | 15 | 3776 | 3790 | trnK-UUU intron |
| Paphiopedilum_philippinense | p5 | (TATTG)3 | 15 | 5098 | 5112 | matK_rps16 |
| Paphiopedilum_philippinense | p5 | (TTTTA)3 | 15 | 64003 | 64017 | petA_psbJ |
| Paphiopedilum_philippinense | p5 | (ATATA)3 | 15 | 71159 | 71173 | clpP intron 1 |
| Paphiopedilum_philippinense | p5 | (ATATA)3 | 15 | 71179 | 71193 | clpP intron 1 |
| Paphiopedilum_philippinense | p5 | (TATAT)3 | 15 | 102570 | 102584 | rps12_trnV-GAC |
| Paphiopedilum_philippinense | p5 | (TATAT)3 | 15 | 102743 | 102757 | rps12_trnV-GAC |
| Paphiopedilum_philippinense | p5 | (ATATA)3 | 15 | 142730 | 142744 | trnV-GAC_rps12 |
| Paphiopedilum_philippinense | p5 | (ATAAT)3 | 15 | 142904 | 142918 | trnV-GAC_rps12 |
| Paphiopedilum_emersonii | p6 | (CTATAG)3 | 18 | 77206 | 77223 | petB intron |
| Paphiopedilum_emersonii | p6 | (TAGAAG)3 | 18 | 95394 | 95411 | ycf2 |
| Paphiopedilum_emersonii | p6 | (GGAAGA)4 | 24 | 113429 | 113452 | ycf1 |
| Paphiopedilum_emersonii | p6 | (AAGAGG)4 | 24 | 113545 | 113568 | ycf1 |
| Paphiopedilum_emersonii | p6 | (CCTCTT)4 | 24 | 129387 | 129410 | ycf1 |
| Paphiopedilum_emersonii | p6 | (CTCTTC)4 | 24 | 129502 | 129525 | ycf1 |
| Paphiopedilum_emersonii | p6 | (CTTCTA)3 | 18 | 147544 | 147561 | ycf2 |
| Paphiopedilum_victoria-mariae | p6 | (TAGAAG)3 | 18 | 98242 | 98259 | ycf2 |
| Paphiopedilum_victoria-mariae | p6 | (GGAAGA)6 | 36 | 116914 | 116949 | ycf1 |
| Paphiopedilum_victoria-mariae | p6 | (CCTCTT)6 | 36 | 133713 | 133748 | ycf1 |
| Paphiopedilum_victoria-mariae | p6 | (CTTCTA)3 | 18 | 152405 | 152422 | ycf2 |
| Paphiopedilum_armeniacum | p6 | (ATATAA)3 | 18 | 65260 | 65277 | ycf4_cemA |
| Paphiopedilum_armeniacum | p6 | (TAGAAG)3 | 18 | 100889 | 100906 | ycf2 |
| Paphiopedilum_armeniacum | p6 | (CTATTA)3 | 18 | 108662 | 108679 | rps12_trnV-GAC |
| Paphiopedilum_armeniacum | p6 | (AATAGT)3 | 18 | 145941 | 145958 | trnV-GAC_rps12 |
| Paphiopedilum_armeniacum | p6 | (CTTCTA)3 | 18 | 153719 | 153736 | ycf2 |
| Paphiopedilum_dianthum | p6 | (TTTATA)3 | 18 | 31768 | 31785 | trnE-UUC_trnT-GGU |
| Paphiopedilum_dianthum | p6 | (TAGAAG)3 | 18 | 95709 | 95726 | ycf2 |
| Paphiopedilum_dianthum | p6 | (GGAAGA)7 | 42 | 113972 | 114013 | ycf1 |
| Paphiopedilum_dianthum | p6 | (CCTCTT)7 | 42 | 127546 | 127587 | ycf1 |
| Paphiopedilum_dianthum | p6 | (CTTCTA)3 | 18 | 145835 | 145852 | ycf2 |
| Paphiopedilum_violascens | p6 | (ATATTA)3 | 18 | 56285 | 56302 | atpB_rbcL |
| Paphiopedilum_violascens | p6 | (CAAATA)3 | 18 | 62522 | 62539 | ycf4_petA |
| Paphiopedilum_violascens | p6 | (TAGAAG)3 | 18 | 96595 | 96612 | ycf2 |
| Paphiopedilum_violascens | p6 | (GGAAGA)3 | 18 | 114522 | 114539 | ycf1 |
| Paphiopedilum_violascens | p6 | (CCTCTT)3 | 18 | 130987 | 131004 | ycf1 |
| Paphiopedilum_violascens | p6 | (CTTCTA)3 | 18 | 148916 | 148933 | ycf2 |
| Paphiopedilum_niveum | p6 | (ATTTAT)3 | 18 | 9838 | 9855 | trnS-GCU_trnG-GCC |
| Paphiopedilum_niveum | p6 | (TAGAAG)3 | 18 | 98785 | 98802 | ycf2 |
| Paphiopedilum_niveum | p6 | (TTTTAA)3 | 18 | 120576 | 120593 | ycf1 |
| Paphiopedilum_niveum | p6 | (ATTAAA)3 | 18 | 128473 | 128490 | ycf1 |
| Paphiopedilum_niveum | p6 | (CTTCTA)3 | 18 | 150265 | 150282 | ycf2 |
| Paphiopedilum_kolopakingii | p6 | (TATTAG)3 | 18 | 30125 | 30142 | petN_psbM |
| Paphiopedilum_kolopakingii | p6 | (TAGAAG)3 | 18 | 97079 | 97096 | ycf2 |
| Paphiopedilum_kolopakingii | p6 | (GGAAGA)6 | 36 | 115927 | 115962 | ycf1 |
| Paphiopedilum_kolopakingii | p6 | (CCTCTT)6 | 36 | 132043 | 132078 | ycf1 |
| Paphiopedilum_kolopakingii | p6 | (CTTCTA)3 | 18 | 150911 | 150928 | ycf2 |
| Paphiopedilum_hirsutissimum | p6 | (TAGAAG)3 | 18 | 93228 | 93245 | ycf2 |
| Paphiopedilum_hirsutissimum | p6 | (GGAAGA)21 | 126 | 111555 | 111680 | ycf1 |
| Paphiopedilum_hirsutissimum | p6 | (CCTCTT)22 | 132 | 128281 | 128412 | ycf1 |
| Paphiopedilum_hirsutissimum | p6 | (CTTCTA)3 | 18 | 146724 | 146741 | ycf2 |
| Paphiopedilum_philippinense | p6 | (TAAATC)3 | 18 | 47721 | 47738 | trnT-UGU_trnL-UAA |
| Paphiopedilum_philippinense | p6 | (AAGAAT)3 | 18 | 69909 | 69926 | rpl20_rps12 |
| Paphiopedilum_philippinense | p6 | (TAGAAG)3 | 18 | 95774 | 95791 | ycf2 |
| Paphiopedilum_philippinense | p6 | (GGAAGA)7 | 42 | 114588 | 114629 | ycf1 |
| Paphiopedilum_philippinense | p6 | (CCTCTT)7 | 42 | 130860 | 130901 | ycf1 |
| Paphiopedilum_philippinense | p6 | (CTTCTA)3 | 18 | 149700 | 149717 | ycf2 |

**Table S6.** Variability of homologous coding regions across the 9 *Paphiopedilum* cp genomes.

| No. | Genes | Length (bp) | Aligned length (bp) | Variable positions | Nucleotide substitutions | Number of indels | Total length of indels | Variability percentage |
| --- | --- | --- | --- | --- | --- | --- | --- | --- |
| 1 | *psbA* | 1062-1107 | 1107 | 59 | 14 | 1 | 45 | 1.41% |
| 2 | *matK* | 1533-1563 | 1569 | 110 | 68 | 1 | 42 | 4.52% |
| 3 | *rps16* | 237 | 237 | 7 | 7 | 0 | 0 | 2.95% |
| 4 | *psbK* | 186-195 | 195 | 14 | 5 | 1 | 9 | 3.21% |
| 5 | *psbI* | 108-111 | 111 | 9 | 6 | 1 | 3 | 6.42% |
| 6 | *atpA* | 1524 | 1524 | 26 | 26 | 0 | 0 | 1.71% |
| 7 | *atpF* | 525-555 | 555 | 52 | 19 | 1 | 33 | 3.82% |
| 8 | *atpH* | 246 | 246 | 1 | 1 | 0 | 0 | 0.41% |
| 9 | *atpI* | 744 | 744 | 11 | 11 | 0 | 0 | 1.48% |
| 10 | *rps2* | 711-804 | 804 | 106 | 13 | 1 | 93 | 1.97% |
| 11 | *rpoC2* | 4086-4101 | 4107 | 139 | 118 | 1 | 21 | 2.91% |
| 12 | *rpoC1* | 2040-2085 | 2085 | 84 | 39 | 1 | 45 | 1.96% |
| 13 | *rpoB* | 3213-3225 | 3225 | 88 | 76 | 1 | 12 | 2.40% |
| 14 | *petN* | 90-93 | 96 | 10 | 1 | 1 | 9 | 2.27% |
| 15 | *psbM* | 105-120 | 121 | 19 | 2 | 1 | 17 | 2.86% |
| 16 | *psbD* | 1062 | 1062 | 22 | 22 | 0 | 0 | 2.07% |
| 17 | *psbC* | 1422 | 1422 | 32 | 32 | 0 | 0 | 2.25% |
| 18 | *psbZ* | 189 | 189 | 0 | 0 | 0 | 0 | 0.00% |
| 19 | *rps14* | 303 | 303 | 7 | 7 | 0 | 0 | 2.31% |
| 20 | *psaB* | 2205 | 2205 | 53 | 53 | 0 | 0 | 2.40% |
| 21 | *psaA* | 2253 | 2253 | 51 | 51 | 0 | 0 | 2.26% |
| 22 | *ycf3* | 510-513 | 513 | 10 | 7 | 1 | 3 | 1.57% |
| 23 | *rps4* | 606 | 606 | 18 | 18 | 0 | 0 | 2.97% |
| 24 | *ndhJ* | 291-507 | 508 | 276 | 0 | 1 | 508 | 100% |
| 25 | *ndhK* | 117-686 | 723 | 651 | 0 | 1 | 723 | 100% |
| 26 | *atpE* | 405 | 405 | 5 | 5 | 0 | 0 | 1.23% |
| 27 | *atpB* | 1506 | 1506 | 23 | 23 | 0 | 0 | 1.53% |
| 28 | *rbcL* | 1464 | 1464 | 11 | 11 | 0 | 0 | 0.75% |
| 29 | *accD* | 1461-1554 | 1632 | 225 | 54 | 1 | 171 | 3.76% |
| 30 | *psaI* | 111 | 111 | 2 | 2 | 0 | 0 | 1.80% |
| 31 | *ycf4* | 549 | 549 | 13 | 13 | 0 | 0 | 2.37% |
| 32 | *cemA* | 225-750 | 773 | 641 | 0 | 1 | 773 | 100% |
| 33 | *petA* | 963 | 963 | 20 | 20 | 0 | 0 | 2.08% |
| 34 | *psbJ* | 123 | 123 | 2 | 2 | 0 | 0 | 1.63% |
| 35 | *psbL* | 117 | 117 | 1 | 1 | 0 | 0 | 0.85% |
| 36 | *psbF* | 120 | 120 | 1 | 1 | 0 | 0 | 0.83% |
| 37 | *psbE* | 252 | 252 | 5 | 5 | 0 | 0 | 1.98% |
| 38 | *petL* | 96 | 96 | 1 | 1 | 0 | 0 | 1.04% |
| 39 | *petG* | 114 | 114 | 2 | 2 | 0 | 0 | 1.75% |
| 40 | *psaJ* | 129-135 | 135 | 8 | 2 | 1 | 6 | 2.31% |
| 41 | *rpl33* | 201 | 201 | 7 | 7 | 0 | 0 | 3.48% |
| 42 | *rps18* | 306-348 | 348 | 54 | 12 | 1 | 42 | 4.23% |
| 43 | *rpl20* | 387-426 | 426 | 56 | 16 | 1 | 40 | 4.39% |
| 44 | *rps12* | 372-622 | 632 | 277 | 7 | 1 | 270 | 2.20% |
| 45 | *clpP* | 615-624 | 624 | 45 | 33 | 1 | 12 | 5.55% |
| 46 | *psbB* | 1527 | 1527 | 21 | 21 | 0 | 0 | 1.38% |
| 47 | *psbT* | 108-114 | 117 | 13 | 1 | 1 | 12 | 1.89% |
| 48 | *psbN* | 132 | 132 | 3 | 3 | 0 | 0 | 2.27% |
| 49 | *psbH* | 222-309 | 309 | 93 | 6 | 1 | 87 | 3.14% |
| 50 | *petB* | 648-663 | 663 | 26 | 11 | 1 | 15 | 1.85% |
| 51 | *petD* | 492 | 492 | 9 | 9 | 0 | 0 | 1.83% |
| 52 | *rpoA* | 1014-1056 | 1056 | 79 | 37 | 1 | 42 | 3.74% |
| 53 | *rps11* | 417 | 417 | 6 | 6 | 0 | 0 | 1.44% |
| 54 | *rpl36* | 114 | 114 | 4 | 4 | 0 | 0 | 3.51% |
| 55 | *infA* | 234 | 234 | 7 | 0 | 1 | 234 | 100% |
| 56 | *rps8* | 396 | 396 | 15 | 15 | 0 | 0 | 3.79% |
| 57 | *rpl14* | 369 | 369 | 12 | 12 | 0 | 0 | 3.25% |
| 58 | *rpl16* | 414-417 | 417 | 10 | 7 | 1 | 3 | 1.93% |
| 59 | *rps3* | 657 | 657 | 21 | 21 | 0 | 0 | 3.20% |
| 60 | *rpl22* | 363-372 | 372 | 28 | 19 | 1 | 9 | 5.49% |
| 61 | *rps19* | 276-279 | 279 | 3 | 0 | 1 | 3 | 0.36% |
| 62 | *rpl2* | 816-921 | 927 | 125 | 11 | 1 | 114 | 1.47% |
| 63 | *rpl23* | 282 | 282 | 2 | 2 | 0 | 0 | 0.71% |
| 64 | *ycf2* | 6801-6945 | 7002 | 595 | 67 | 1 | 528 | 1.05% |
| 65 | *ndhB* | 753-2234 | 2243 | 1583 | 11 | 1 | 1572 | 1.79% |
| 66 | *rps7* | 468 | 468 | 5 | 5 | 0 | 0 | 1.07% |
| 67 | *ycf15* | 117 | 117 | 0 | 0 | 1 | 117 | 100% |
| 68 | *ycf1* | 5580-5802 | 5925 | 622 | 184 | 1 | 438 | 3.37% |
| 69 | *rps15* | 273 | 273 | 7 | 7 | 0 | 0 | 2.56% |
| 70 | *psaC* | 246 | 246 | 3 | 3 | 0 | 0 | 1.22% |
| 71 | *rpl32* | 165-177 | 177 | 28 | 14 | 1 | 14 | 9.15% |
| 72 | *ccsA* | 975-984 | 984 | 47 | 38 | 1 | 9 | 4.00% |
|  | Total |  | **63296** | **6621** | **1327** | **36** | **6074** | **2.38%** |

**Table S7.** Variability of homologous non-coding regions across the 9 *Paphiopedilum* cp genomes.

| No. | Fragments | Length (bp) | Aligned length (bp) | Variable positions | Nucleotide substitutions | Number of indels | Total length of indels | Variability percentage |
| --- | --- | --- | --- | --- | --- | --- | --- | --- |
| 1 | psbA_trnK-UUU | 220-257 | 266 | 94 | 18 | 1 | 76 | 9.95% |
| 2 | trnK-UUU_intron | 1559-3146 | 3341 | 1878 | 73 | 1 | 1805 | 4.81% |
| 3 | matK_rps16 | 1765-2287 | 2583 | 1229 | 151 | 1 | 1078 | 10.09% |
| 4 | rps16_intron | 833-949 | 972 | 228 | 57 | 1 | 171 | 7.23% |
| 5 | rps16_trnQ-UUG | 330-940 | 1002 | 724 | 17 | 1 | 707 | 6.08% |
| 6 | trnQ-UUG_psbK | 321-382 | 405 | 124 | 22 | 1 | 102 | 7.57% |
| 7 | psbK_psbI | 470-552 | 640 | 326 | 90 | 1 | 236 | 22.47% |
| 8 | psbI_trnS-GCU | 157-160 | 160 | 11 | 8 | 1 | 3 | 5.70% |
| 9 | trnS-GCU_trnG-UCC | 1040-1966 | 2284 | 1507 | 0 | 1 | 2284 | 100% |
| 10 | trnG-UCC_intron | 58-685 | 698 | 650 | 0 | 1 | 698 | 100% |
| 11 | trnG-UCC_trnR-UCU | 85-138 | 138 | 60 | 0 | 1 | 138 | 100% |
| 12 | trnR-UCU_atpA | 203-341 | 367 | 208 | 37 | 1 | 171 | 19.29% |
| 13 | atpA_atpF | 70-72 | 72 | 7 | 5 | 1 | 2 | 8.45% |
| 14 | atpF_intron | 816-859 | 900 | 162 | 33 | 1 | 129 | 4.40% |
| 15 | atpF_atpH | 100-334 | 343 | 258 | 14 | 1 | 244 | 15.00% |
| 16 | atpH_atpI | 606-747 | 786 | 228 | 30 | 1 | 198 | 5.26% |
| 17 | atpI_rps2 | 257-292 | 293 | 48 | 10 | 1 | 38 | 4.30% |
| 18 | rps2_rpoC2 | 191-289 | 293 | 110 | 8 | 1 | 102 | 4.69% |
| 19 | rpoC2_rpoC1 | 183 | 183 | 11 | 11 | 0 | 0 | 6.01% |
| 20 | rpoC1_intron | 767-792 | 808 | 68 | 24 | 1 | 44 | 3.27% |
| 21 | rpoC1_rpoB | 5-26 | 26 | 21 | 0 | 1 | 21 | 16.67% |
| 22 | rpoB_trnC-GCA | 773-1366 | 1411 | 718 | 30 | 1 | 688 | 4.28% |
| 23 | trnC-GCA_petN | 706-1021 | 1063 | 540 | 52 | 1 | 488 | 9.20% |
| 24 | petN_psbM | 434-1636 | 1868 | 1599 | 51 | 1 | 1548 | 16.20% |
| 25 | psbM_trnD-GUC | 843-863 | 877 | 97 | 56 | 1 | 41 | 6.81% |
| 26 | trnD-GUC_trnY-GUA | 149-341 | 354 | 222 | 13 | 1 | 209 | 9.59% |
| 27 | trnY-GUA_trnE-UUC | 59 | 59 | 4 | 4 | 0 | 0 | 6.78% |
| 28 | trnE-UUC_trnT-GGU | 492-1725 | 2185 | 2019 | 13 | 1 | 2006 | 7.78% |
| 29 | trnT-GGU_psbD | 1019-1056 | 1071 | 133 | 71 | 1 | 62 | 7.13% |
| 30 | psbC_trnS-UGA | 145-151 | 152 | 23 | 16 | 1 | 7 | 11.64% |
| 31 | trnS-UGA_psbZ | 225-230 | 231 | 11 | 5 | 1 | 6 | 2.65% |
| 32 | psbZ_trnG-GCC | 247-271 | 287 | 49 | 0 | 1 | 287 | 100% |
| 33 | trnG-GCC_trnfM-CAU | 170-188 | 189 | 23 | 0 | 1 | 189 | 100% |
| 34 | trnfM-CAU_rps14 | 152-181 | 182 | 37 | 4 | 1 | 33 | 3.33% |
| 35 | rps14_psaB | 162-173 | 193 | 41 | 4 | 1 | 37 | 3.18% |
| 36 | psaB_psaA | 25 | 25 | 0 | 0 | 0 | 0 | 0.00% |
| 37 | psaA_ycf3 | 444-651 | 651 | 225 | 18 | 1 | 207 | 4.27% |
| 38 | ycf3_intron1 | 716-720 | 725 | 24 | 12 | 1 | 12 | 1.82% |
| 39 | ycf3_intron2 | 710-779 | 799 | 108 | 18 | 1 | 90 | 2.68% |
| 40 | ycf3_trnS-GGA | 552-617 | 637 | 119 | 29 | 1 | 90 | 5.47% |
| 41 | trnS-GGA_rps4 | 270-280 | 283 | 23 | 9 | 1 | 14 | 3.70% |
| 42 | rps4_trnT-UGU | 378-458 | 502 | 164 | 24 | 1 | 140 | 6.89% |
| 43 | trnT-UGU_trnL-UAA | 574-1099 | 1184 | 688 | 27 | 1 | 661 | 5.34% |
| 44 | trnL-UAA_intron | 541-569 | 574 | 76 | 24 | 1 | 52 | 4.78% |
| 45 | trnL-UAA_trnF-GAA | 358-410 | 416 | 74 | 9 | 1 | 65 | 2.84% |
| 46 | trnF-GAA_ndhJ | 582-610 | 642 | 97 | 0 | 1 | 642 | 100% |
| 47 | ndhJ_ndhK | 26-347 | 349 | 329 | 0 | 1 | 349 | 100% |
| 48 | ndhK_trnV-UAC | 713-1397 | 1454 | 816 | 0 | 1 | 1454 | 100% |
| 49 | trnV-UAC_intron | 577-588 | 588 | 24 | 12 | 1 | 12 | 2.25% |
| 50 | trnV-UAC_trnM-CAU | 173-174 | 175 | 12 | 10 | 1 | 2 | 6.32% |
| 51 | trnM-CAU_atpE | 202-209 | 209 | 15 | 8 | 1 | 7 | 4.43% |
| 52 | atpB_rbcL | 1038-1390 | 1525 | 657 | 64 | 1 | 593 | 6.97% |
| 53 | rbcL_accD | 710-809 | 865 | 224 | 34 | 1 | 190 | 5.18% |
| 54 | accD_psaI | 489-937 | 1011 | 596 | 32 | 1 | 564 | 7.37% |
| 55 | psaI_ycf4 | 385-392 | 396 | 34 | 23 | 1 | 11 | 6.22% |
| 56 | ycf4_cemA | 443-1052 | 1344 | 1283 | 0 | 1 | 1344 | 100% |
| 57 | cemA_petA | 238-379 | 380 | 152 | 0 | 1 | 380 | 100% |
| 58 | petA_psbJ | 983-1060 | 1112 | 201 | 62 | 1 | 139 | 6.47% |
| 59 | psbJ_psbL | 123-153 | 153 | 40 | 10 | 1 | 30 | 8.87% |
| 60 | psbL_psbF | 22 | 22 | 1 | 1 | 0 | 0 | 4.55% |
| 61 | psbF_psbE | 9-10 | 10 | 1 | 0 | 1 | 1 | 10.00% |
| 62 | psbE_petL | 766-880 | 896 | 252 | 31 | 1 | 221 | 4.73% |
| 63 | petL_petG | 185-193 | 195 | 24 | 14 | 1 | 10 | 8.06% |
| 64 | petG_trnW-CCA | 125-128 | 128 | 20 | 17 | 1 | 3 | 14.29% |
| 65 | trnW-CCA_trnP-UGG | 154-162 | 163 | 15 | 5 | 1 | 10 | 3.90% |
| 66 | trnP-UGG_psaJ | 598-1112 | 1284 | 1072 | 144 | 1 | 928 | 40.62% |
| 67 | psaJ_rpl33 | 474-549 | 576 | 133 | 26 | 1 | 107 | 5.74% |
| 68 | rpl33_rps18 | 260-426 | 448 | 241 | 24 | 1 | 217 | 10.78% |
| 69 | rps18_rpl20 | 210-274 | 279 | 130 | 19 | 1 | 111 | 11.83% |
| 70 | rpl20_rps12 | 794-811 | 827 | 75 | 38 | 1 | 37 | 4.93% |
| 71 | clpP_intron1 | 1005-1210 | 1340 | 563 | 89 | 1 | 474 | 10.38% |
| 72 | clpP_intron2 | 710-902 | 963 | 312 | 43 | 1 | 269 | 6.33% |
| 73 | clpP_psbB | 483-1114 | 1228 | 872 | 42 | 1 | 830 | 10.78% |
| 74 | psbB_psbT | 271-636 | 786 | 643 | 29 | 1 | 614 | 17.34% |
| 75 | psbT_psbN | 65-69 | 69 | 9 | 4 | 1 | 5 | 7.69% |
| 76 | psbN_psbH | 23-110 | 110 | 95 | 2 | 1 | 93 | 16.67% |
| 77 | psbH_petB | 167-200 | 216 | 59 | 9 | 1 | 50 | 5.99% |
| 78 | petB_intron | 743-770 | 779 | 71 | 24 | 1 | 47 | 3.41% |
| 79 | petB_petD | 183-192 | 198 | 24 | 9 | 1 | 15 | 5.43% |
| 80 | petD_intron | 856-904 | 1045 | 366 | 36 | 1 | 330 | 5.17% |
| 81 | petD_rpoA | 193-205 | 211 | 30 | 11 | 1 | 19 | 6.22% |
| 82 | rpoA_rps11 | 41-83 | 83 | 47 | 5 | 1 | 42 | 14.29% |
| 83 | rps11_rpl36 | 169-189 | 189 | 31 | 8 | 1 | 23 | 5.39% |
| 84 | rpl36_infA | 101-109 | 110 | 11 | 0 | 1 | 110 | 100% |
| 85 | infA_rps8 | 121-129 | 136 | 18 | 0 | 1 | 136 | 100% |
| 86 | rps8_rpl14 | 423-563 | 768 | 503 | 85 | 1 | 418 | 24.50% |
| 87 | rpl14_rpl16 | 124-144 | 144 | 32 | 12 | 1 | 20 | 10.40% |
| 88 | rpl16_intron | 1071-1584 | 1697 | 733 | 56 | 1 | 677 | 5.58% |
| 89 | rpl16_rps3 | 121-170 | 179 | 64 | 6 | 1 | 58 | 5.74% |
| 90 | rps3_rpl22 | 70-105 | 115 | 63 | 7 | 1 | 56 | 13.33% |
| 91 | rpl22_rps19 | 224-266 | 269 | 52 | 7 | 1 | 45 | 3.56% |
| 92 | rps19_trnH-GUG | 144-145 | 145 | 5 | 4 | 1 | 1 | 3.45% |
| 93 | trnH-GUG_rpl2 | 43-46 | 46 | 8 | 5 | 1 | 3 | 13.64% |
| 94 | rpl2_intron | 559-664 | 664 | 108 | 3 | 1 | 105 | 0.71% |
| 95 | rpl2_rpl23 | 18-24 | 24 | 6 | 0 | 1 | 6 | 5.26% |
| 96 | rpl23_trnI-CAU | 159 | 159 | 3 | 3 | 0 | 0 | 1.89% |
| 97 | trnI-CAU_ycf2 | 61-68 | 68 | 10 | 3 | 1 | 7 | 6.45% |
| 98 | ycf2_trnL-CAA | 94-997 | 1028 | 976 | 0 | 1 | 976 | 1.89% |
| 99 | trnL-CAA_ndhB | 540-740 | 745 | 211 | 6 | 1 | 205 | 1.29% |
| 100 | ndhB_intron | 328-703 | 703 | 375 | 0 | 1 | 703 | 100% |
| 101 | ndhB_rps7 | 297-617 | 635 | 344 | 6 | 1 | 338 | 2.35% |
| 102 | rps12_intron | 547-548 | 552 | 22 | 13 | 1 | 9 | 2.57% |
| 103 | rps12_ycf15 | 1044-1176 | 1176 | 134 | 0 | 1 | 1176 | 100% |
| 104 | ycf15_trnV-GAC | 770-777 | 777 | 7 | 0 | 1 | 777 | 100% |
| 105 | trnV-GAC_rrn16 | 229 | 229 | 5 | 5 | 0 | 0 | 2.18% |
| 106 | rrn16_trnI-GAU | 302 | 302 | 3 | 3 | 0 | 0 | 0.99% |
| 107 | trnI-GAU_intron | 930-942 | 949 | 30 | 6 | 1 | 24 | 0.76% |
| 108 | trnI-GAU_trnA-UGC | 64 | 64 | 0 | 0 | 0 | 0 | 0.00% |
| 109 | trnA-UGC_intron | 787-801 | 802 | 19 | 4 | 1 | 15 | 0.63% |
| 110 | trnA-UGC_rrn23 | 145 | 145 | 3 | 3 | 0 | 0 | 2.07% |
| 111 | rrn23_rrn4.5 | 98-100 | 100 | 3 | 1 | 1 | 2 | 2.02% |
| 112 | rrn4.5_rrn5 | 220 | 220 | 1 | 1 | 0 | 0 | 0.45% |
| 113 | rrn5_trnR-ACG | 166-251 | 251 | 101 | 10 | 1 | 91 | 6.83% |
| 114 | trnR-ACG_trnN-GUU | 287-509 | 509 | 366 | 6 | 1 | 360 | 4.67% |
| 115 | trnN-GUU_ycf1 | 279-355 | 366 | 100 | 7 | 1 | 93 | 2.92% |
| 116 | ycf1_rps15 | 349-445 | 451 | 126 | 0 | 1 | 451 | 100% |
| 117 | rps15_psaC | 460-696 | 747 | 457 | 0 | 1 | 747 | 100% |
| 118 | psaC_trnL-UAG | 339-2964 | 3022 | 2748 | 0 | 1 | 3022 | 100% |
| 119 | trnL-UAG_rpl32 | 302-510 | 594 | 426 | 0 | 1 | 594 | 100% |
| 120 | rpl32_ccsA | 137-183 | 194 | 109 | 0 | 1 | 194 | 100% |
| 121 | ccsA_psaC | 1492-1637 | 1670 | 517 | 224 | 1 | 293 | 16.33% |
|  | Total |  | **75481** | **33234** | **2468** | **111** | **37064** | **6.69%** |
